# Affinity-Based Profiling of the Flavin Mononucleotide Riboswitch

**DOI:** 10.1021/jacs.2c02685

**Published:** 2022-06-06

**Authors:** Stefan Crielaard, Rick Maassen, Tess Vosman, Ivy Rempkens, Willem A. Velema

**Affiliations:** Institute for Molecules and Materials, Radboud University Nijmegen, Heyendaalseweg 135, 6525 AJ Nijmegen, The Netherlands

## Abstract

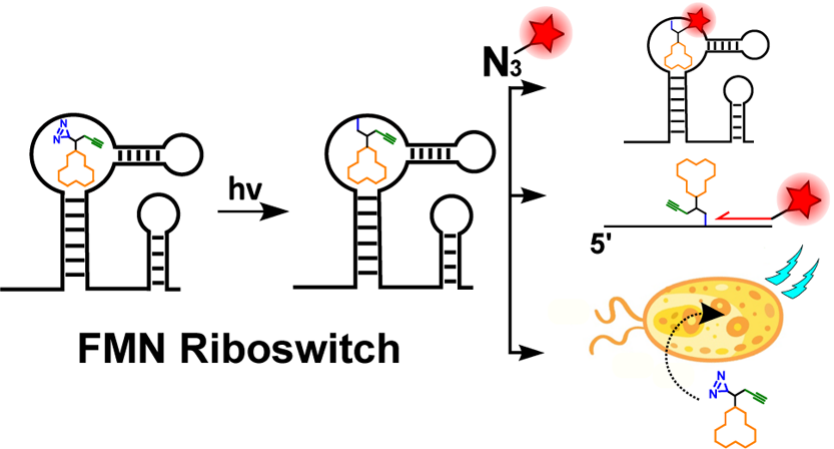

Riboswitches are structural RNA elements that control gene expression. These naturally occurring RNA sensors are of continued interest as antibiotic targets, molecular sensors, and functional elements of synthetic circuits. Here, we describe affinity-based profiling of the flavin mononucleotide (FMN) riboswitch to characterize ligand binding and structural folding. We designed and synthesized photoreactive ligands and used them for photoaffinity labeling. We showed selective labeling of the FMN riboswitch and used this covalent interaction to quantitatively measure ligand binding, which we demonstrate with the naturally occurring antibiotic roseoflavin. We measured conditional riboswitch folding as a function of temperature and cation concentration. Furthermore, combining photoaffinity labeling with reverse transcription revealed ligand binding sites within the aptamer domain with single-nucleotide resolution. The photoaffinity probe was applied to cellular extracts of *Bacillus subtilis* to demonstrate conditional folding of the endogenous low-abundant *ribD* FMN riboswitch in biologically derived samples using quantitative PCR. Lastly, binding of the riboswitch-targeting antibiotic roseoflavin to the FMN riboswitch was measured in live bacteria using the photoaffinity probe.

## Introduction

RNA is a multifaceted biomolecule that exhibits many crucial cellular functions ranging from architectural to catalytic.^1−3^ These functions go far beyond the initially proposed role of RNA as information carrier. Recent discoveries have revealed the role of RNA in chromatin regulation,^4^ post-transcriptional regulation,^5^ gene silencing^6^ and enhancing,^7^ and transcription and translational control through riboswitches,^8−14^ among other functions. The newly appreciated importance of RNA in pathological processes has sparked interest in the development of selective small-molecule drugs that can modulate cellular RNA activity.^15−18^ To study the diverse functions, structure, and druggability of RNA, there is a need for new chemical tools.^3,19−23^

**Figure 1:**
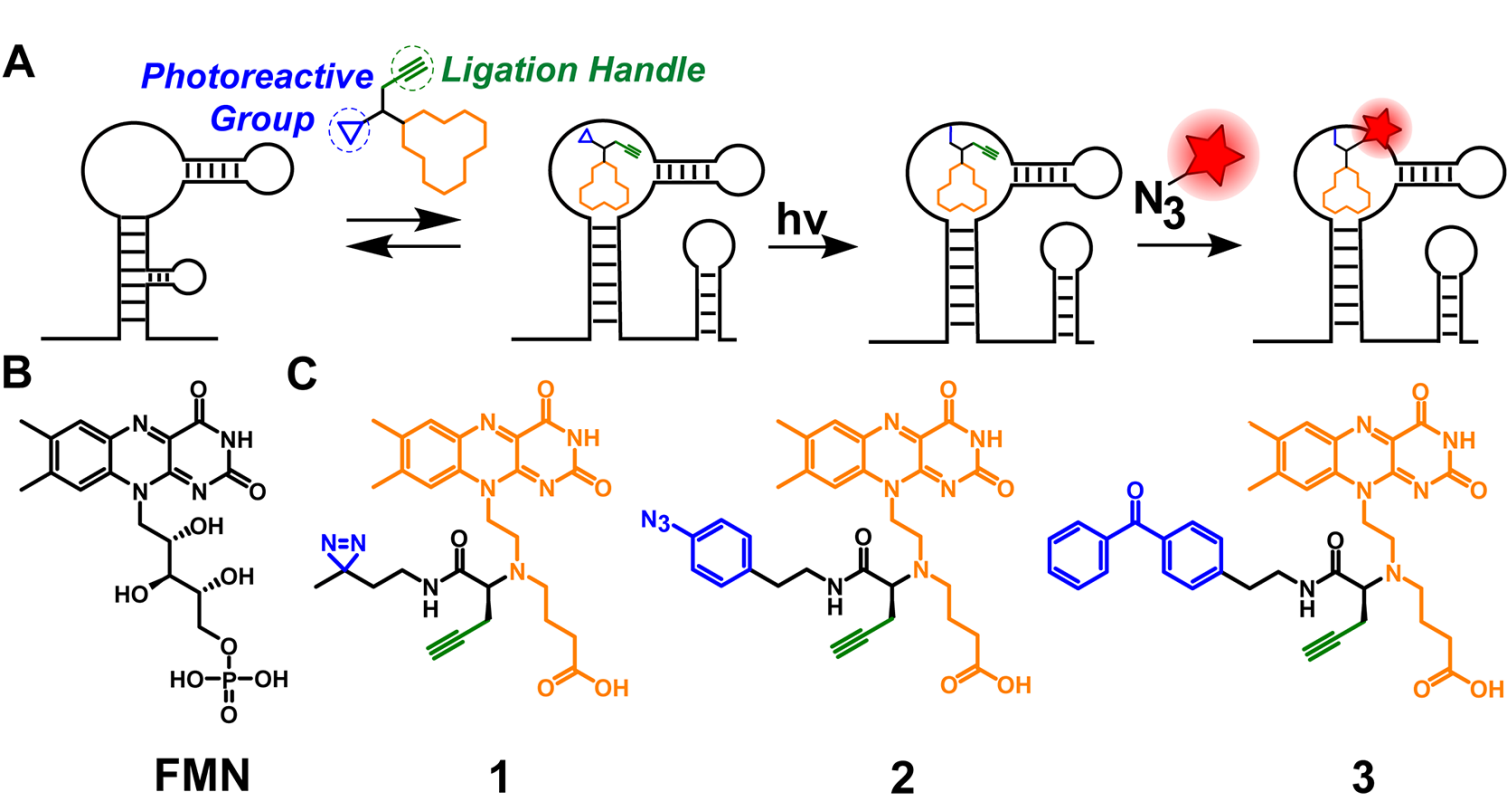
Schematic overview of affinity-based profiling of riboswitches. (A) Schematic illustration of the photoaffinity labeling workflow. Cross-linking is achieved through light activation of the photoreactive group, and the alkyne ligation handle enables downstream analysis. (B) Molecular structure of the natural ligand flavin mononucleotide (FMN). (C) Structures of the three designed and synthesized bifunctional photoaffinity probes **1**, **2**, and **3**. The orange parts represent the RNA-binding moiety, the blue parts the photoactivatable groups, and the green parts the attached alkyne ligation handle.

Early pioneering studies demonstrated the broad potential of (photo)chemical methods to investigate nucleic acid structure and function.^24−28^ An emerging approach to study RNA is applying small chemical probes that bind RNA with high affinity and that form covalent bonds between the probe and RNA of interest. The covalently bound small-molecule probe can be subsequently modified for analysis. Recent reports have employed this concept to investigate small-molecule binding to pre-mRNAs^29^ and microRNAs^30,31^ (CHEM-Clip) and artificial synthetic aptamers (PEARL-seq).^32^ Here, we explore if we can apply similar principles to characterize ligand binding and structural folding of bacterial riboswitches (Figure 1A).

Riboswitches are *cis*-regulatory structural RNA elements that are found in the 5′ untranslated region of mRNAs. They control gene expression in several organisms and are broadly distributed across bacteria.^10,11,33,34^ They consist of an aptamer domain and an expression platform. Binding of the cognate ligand to the aptamer induces a conformational change in the expression platform, thereby altering the expression of the downstream gene.^10,35^ The aptamer is highly conserved and serves as a sensor for its target metabolite. These sensors must be sufficiently dynamic to respond and adapt precisely to specific signals, to rapidly produce the intended effect for cell survival.

One widely distributed class of RNA regulatory elements in bacteria is the flavin mononucleotide (FMN) riboswitch with FMN (Figure 1B) as primary regulatory ligand.^8,36^ This riboswitch controls expression of genes required for biosynthesis and transport of riboflavin, an important vitamin for both bacteria and humans.^37^ FMN regulates the expression of these downstream genes upon binding to the aptamer.^8^

Mechanistic data of riboswitches are often obtained with synthetic RNA, using a combination of physical methods, such as nuclear magnetic resonance (NMR) studies,^38−40^ X-ray diffraction crystallography,^36,41^ fluorescence spectroscopy,^42,43^*in vitro* profiling methods,^8,41,44,45^ and genetic approaches.^46,47^ Though biologically vital, the structural features of riboswitches can prove challenging to study and remain of continued interest.^11,33^

Chemical methods that covalently capture the interaction between a riboswitch and its ligand could further assist in elucidating riboswitch properties and druggability.

Here, we set out to develop chemical photoaffinity probes to study the interactions between the FMN riboswitch and its ligands on purified RNA, as well as in bacterial extracts and live bacteria. Inspired by activity-based protein profiling^48,49^ and recent reports of photoaffinity labeling of RNA,^29,32,50−52^ we designed three photoreactive FMN ligands (Figure 1C). Labeling of the FMN riboswitch was observed for two of three synthesized probes. Competition experiments with its cognate ligand FMN and the naturally occurring antibiotic roseoflavin demonstrate the potential use of photoaffinity probes to screen for riboswitch inhibitors. Conditional riboswitch folding, controlled by temperature and cation concentration, was analyzed with the probe. Furthermore, photo-cross-linking sites could be identified with single-nucleotide resolution, potentially helping to pinpoint the binding site within the riboswitch aptamer domain. Finally, quantification of probe binding in bacterial extracts and live bacteria was achieved using bead enrichment and RT-qPCR, demonstrating the potential use of photoaffinity labeling to measure riboswitch inhibitor binding *in vivo*.

## Results and Discussion

### Design and Synthesis of FMN Riboswitch Photoaffinity Probes

To measure the interactions between the FMN riboswitch and its ligand, we designed and synthesized photoreactive derivatives of FMN that can covalently label RNA when bound in the aptamer region of the riboswitch and exposed to UV light (Figure 1A and C). Seminal work by Vicens, Batey, and co-workers^53^ provided a set of principles to ensure specific and productive binding of FMN analogues to the FMN riboswitch that we followed.^53^ Importantly, they showed that the ribityl-phosphate chain attached to position 10 of the isoalloxazine ring of FMN (Figure 1B) can be modified without perturbing the interactions between the FMN riboswitch and its ligand. Additionally, it was found that the hydroxyl groups of the ribityl chain could be removed without losing affinity for the riboswitch.^53^ Lastly, it was shown that the terminal phosphate group can be replaced with a carboxylic anion moiety.^53^ On the basis of these structural constraints, we designed probes **1**, **2**, and **3**, which all contain the isoalloxazine core that is crucial for binding (Figure 1C).^53^ The ribityl chain was replaced with a linker containing a terminal carboxylate and tertiary amine to which a photoreactive group and alkyne are appended. The tertiary amine was also found to be beneficial for enhanced affinity for the FMN riboswitch and bacterial uptake.^53^ Three photoaffinity probes were designed and synthesized, containing the frequently used diazirine, aryl azide, and benzophenone photoreactive groups (Figure 1C).^54^ When the probes bind in the FMN riboswitch aptamer domain, activation of the photoreactive groups can induce the formation of a covalent bond with the FMN RNA. For convenient downstream analysis of these probe–RNA complexes, an alkyne ligation handle was included in the designs of probes **1**, **2**, and **3** (Figure 1C).

### Performance of Photoaffinity Probes

**Figure 2:**
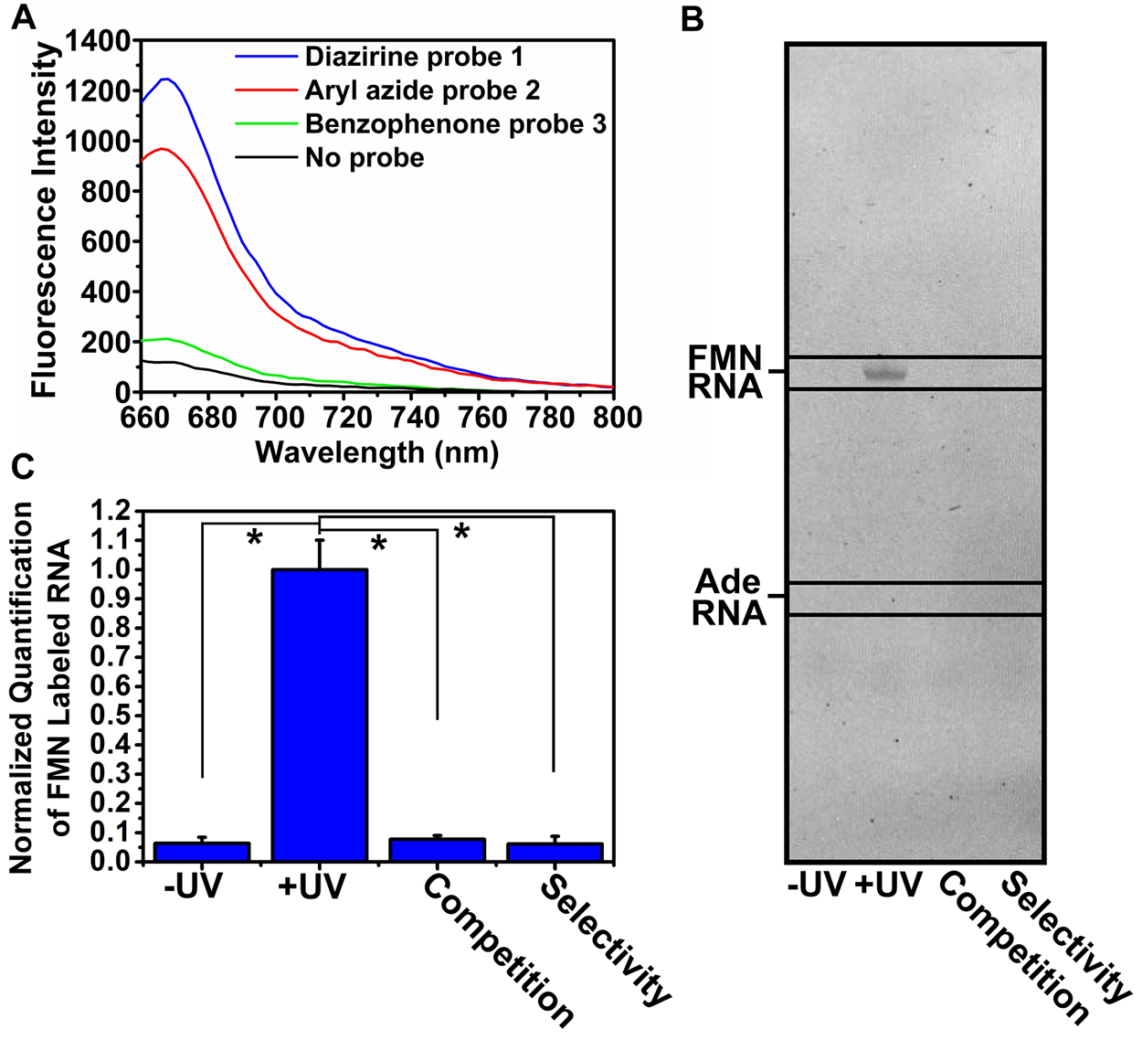
Efficiency and selectivity of FMN probes **1**, **2**, and **3**. (A) Fluorescence intensity measurements after labeling FMN riboswitch aptamer with 10 μM **1**, **2**, or **3** and subsequent “click” reaction with Cy5-azide. (B) Denaturing RNA gel showing selective binding of probe **1** to the FMN RNA aptamer. (C). Quantification of band intensities of the PAGE experiment in B in triplicate. “Competition” is labeling in the presence of 10 μM FMN natural ligand. “Selectivity” is labeling on the adenine riboswitch. Error bars represent standard deviations based on three technical replicates. Statistical significance was calculated using an unpaired two-tailed Student’s *t* test (*p < 0.05).

The performance of photoaffinity probes **1**, **2**, and **3** was examined by measuring probe binding to an *in vitro* transcribed FMN riboswitch (see the SI for details) upon UV irradiation. After ligating a Cy5 fluorophore to the probe–RNA complexes using copper(I)-catalyzed azide–alkyne cycloaddition, the fluorescence intensity was measured to examine probe binding at concentrations ranging 2–100 μM (Figures 2A and S1). At a concentration of 10 μM, diazirine probe **1** efficiently labeled the riboswitch, as was apparent from a significant fluorescent signal (Figures 2A and S1).

Aryl azide probe **2** displayed efficient labeling as well, whereas benzophenone probe **3** showed only minor fluorescence. A potential explanation is that the size of the photo-cross-linking group is crucial for optimal probe binding, with the bulky benzophenone group being too sterically hindered to effectuate labeling.^54^ Moreover, it is possible that the “click” reaction works less efficiently for ligated probe **3**, which would result in lower fluorescence. Because of the superior performance of probe **1** compared to probe **3** and slightly better performance than probe **2**, we decided to use probe **1** for all further experiments.

To test the selectivity of probe **1** for the FMN riboswitch, labeling was analyzed with denaturing polyacrylamide gel electrophoresis (PAGE) (Figures 2B,C and S2). Both UV irradiation and fluorophore ligation were required to observe labeling of the FMN riboswitch by probe **1**. To show that probe **1** selectively bound to the FMN riboswitch, 10 μM FMN natural ligand was incubated as a competitor for the probe to bind to the riboswitch aptamer. The observed labeling signal disappeared, indicating that the probe was competed out of the aptamer domain (Figures 2B,C and S2). This might imply a selective interaction between probe **1** and the FMN riboswitch. To further demonstrate selectivity, probe **1** was incubated with the adenine riboswitch, instead of the FMN riboswitch. No labeling of the adenine riboswitch was detected, underlining the selectivity of probe **1** for the FMN riboswitch aptamer (Figures 2B,C and S2).

### Structure Probing of the Riboswitch–Probe Interaction

**Figure 3:**
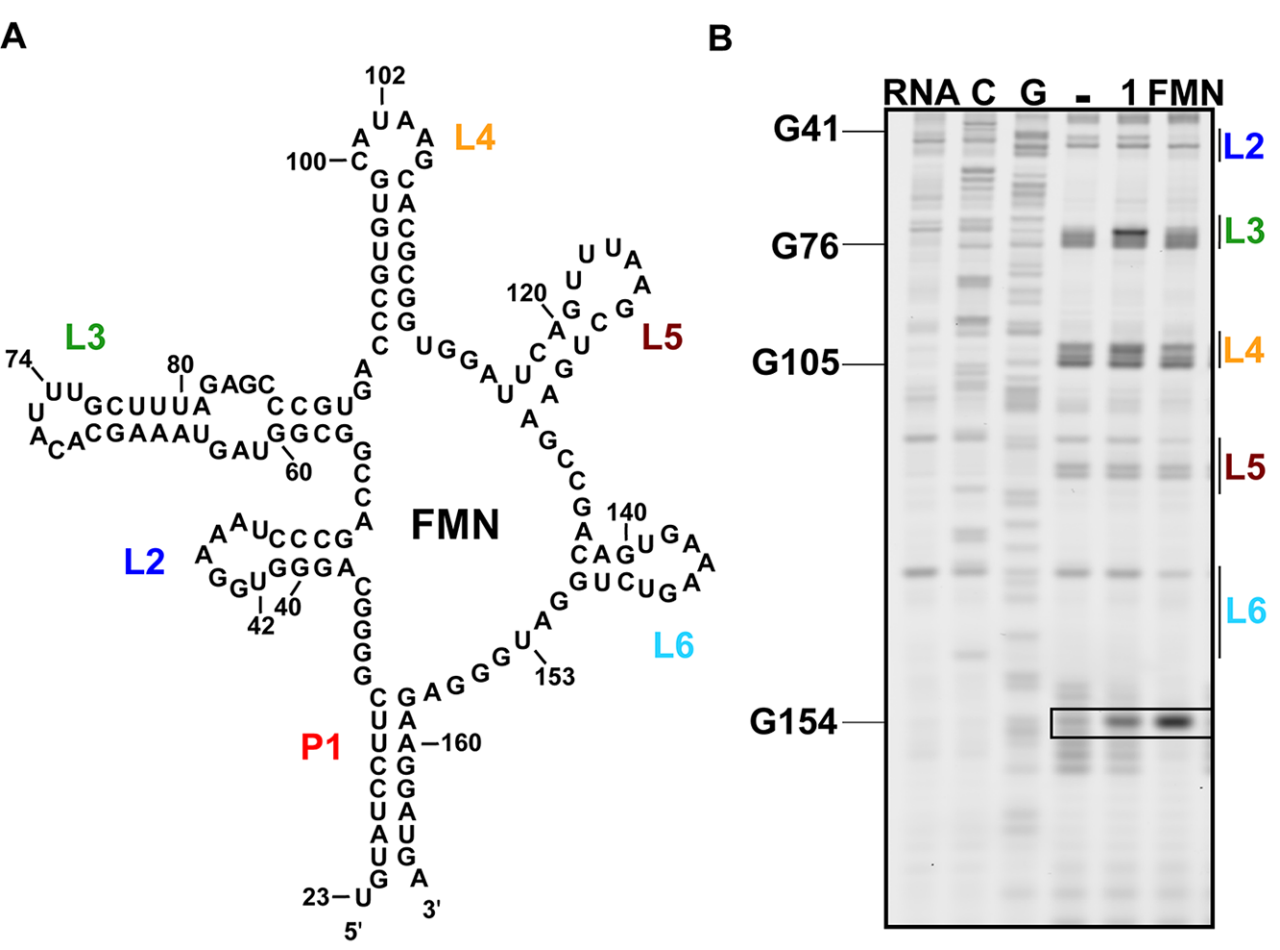
SHAPE analysis of interaction of probe **1** with the FMN riboswitch. (A) Secondary structure of the FMN riboswitch from *Bacillus subtilis*, with loop regions annotated in color. (B) PAGE of the SHAPE experiment with probe **1** and the FMN ligand. The boxed area shows the area of the gel where characteristic changes in the SHAPE profile are observed for ligand binding. For triplicate experiments and quantification of band intensities see Figure S3.

To study the binding interaction between probe **1** and the FMN riboswitch, SHAPE analysis^3,55,56^ was performed (see the SI for details). For SHAPE, FMN and probe **1** were separately incubated at 100 μM with the riboswitch at 37 °C for 30 min, after which 50 mM 2-methylnicotinic acid imidazolide (NAI)^57^ was added to each sample. The samples were incubated at 37 °C for 10 min, and RNA was isolated using precipitation. Reverse transcription (RT) was performed and analyzed using PAGE (Figures 3 and S3). Increased reactivity at U153 was observed for both FMN and probe **1**. This is believed to be typical for ligand binding to the aptamer and caused by an altered conformation in which U153 is bulged out and exposed to solvent;^44^ it might indicate that probe **1** displays similarity in binding to the aptamer as compared to FMN. Slight differences in SHAPE profile were observed as well at nucleotides U42, U74, and U102, which are located in L2, L3, and L4, respectively, and may be caused by differences in structure of the side-chain. Increased reactivity in these loop regions was observed previously for FMN structural analogues on which probe **1** was based.^53^ This was further analyzed by molecular docking of probe **1** to the FMN aptamer (Figure S4), which showed similar binding of the isoalloxazine core to FMN and no noticeable differences at nucleotide U42.

Together, these results imply that the isoalloxazine core of photoreactive probe **1** likely interacts with the FMN riboswitch in a similar manner to the natural ligand and that the diazirine group in the side-chain might cause slight changes in structure.

### Competitive Photoaffinity Labeling of the FMN Riboswitch

**Figure 4:**
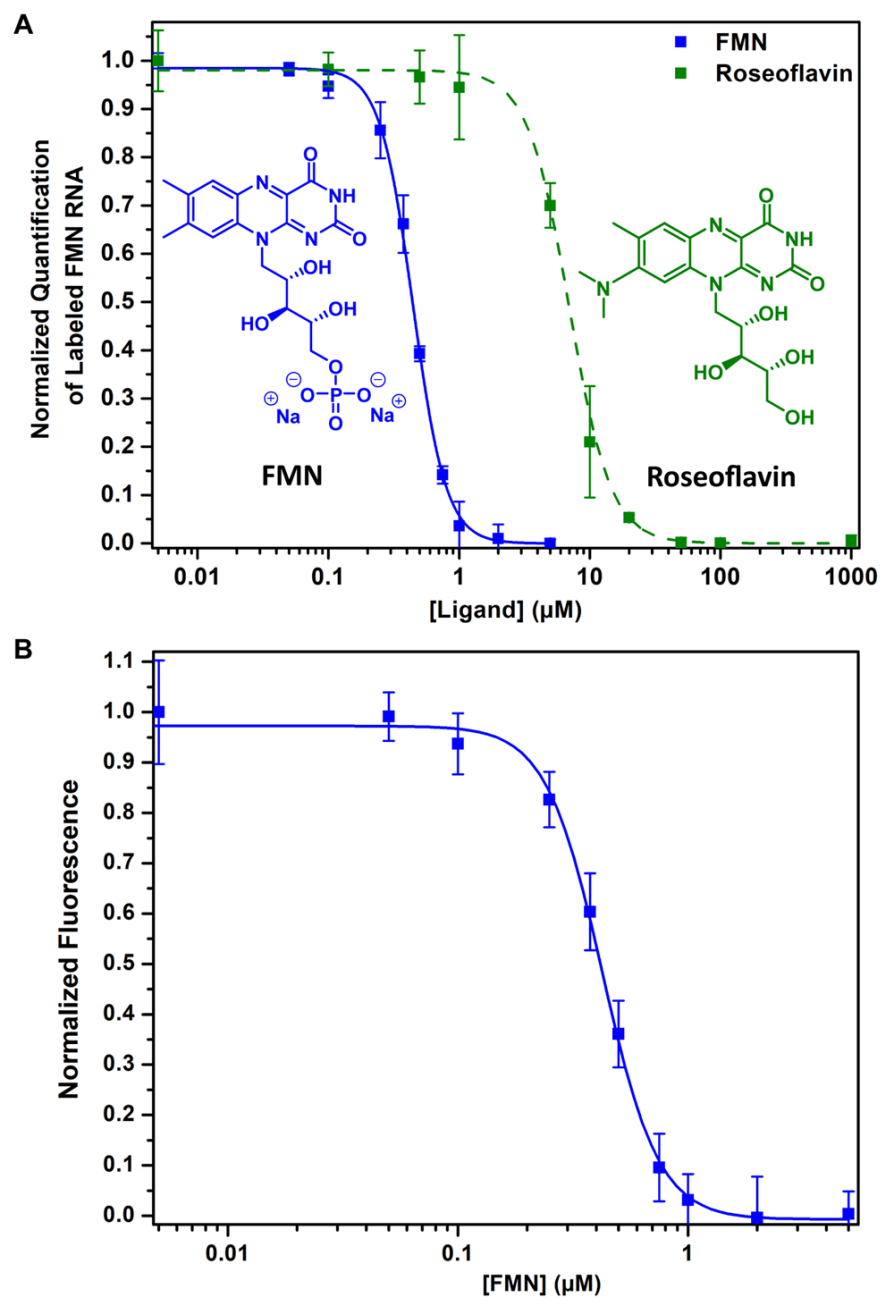
Competitive photoaffinity labeling. (A) Dose-dependent inhibition of labeling with the FMN ligand and roseoflavin. After photolabeling, the probe is modified with a fluorescein fluorophore and binding is determined by PAGE and subsequently quantified by measuring band intensity in triplicates (see Figures S5 and S6). (B) Competitive photoaffinity labeling with the FMN ligand quantified using a microplate reader. Error bars represent standard deviations based on three technical replicates.

To show that the photoaffinity labeling approach can be used to study the interactions of small molecules with a riboswitch aptamer, a competition experiment was performed. To this end, a series of concentrations of the natural ligand FMN and the FMN riboswitch targeting bacterial antibiotic roseoflavin^58^ were preincubated with the FMN riboswitch. Subsequently, probe **1** (10 μM) was added, cross-linked, modified with fluorescein-azide, and finally analyzed using PAGE, and band intensities were quantified to determine the dose-dependent inhibition of labeling. IC_50_ values were calculated using a sigmoidal fit (see the SI for details). Both FMN and roseoflavin were capable of fully outcompeting probe **1**, with an apparent IC_50_ of 0.4 ± 0.01 and 7.0 ± 0.18 μM, respectively (Figures 4A, S5, and S6). This observed ∼20-fold difference is in accordance with earlier observed dissociation constants (*K*_D_) of FMN and roseoflavin for the *ribD* riboswitch from *Bacillus subtilis* (*B. subtilis*).^58^

To further improve the utility of this approach, a fluorescently labeled FMN riboswitch was quantified on a microplate reader, yielding similar results with an apparent IC_50_ of 0.4 ± 0.01 μM for FMN and avoiding the need for PAGE (Figure 4B). We expect that this feature will be found useful for screening for new small-molecule riboswitch binders.^59,60^

### Conditional Aptamer Folding

Riboswitch conformations are conditional and depend on temperature and ion concentrations.^34^ When the riboswitch unfolds at increased temperature or low cation concentration, the interaction between RNA and ligand is lost.^61^ Bacteria are suggested to exploit these phenomena to fine-tune their gene regulation.^14^

**Figure 5:**
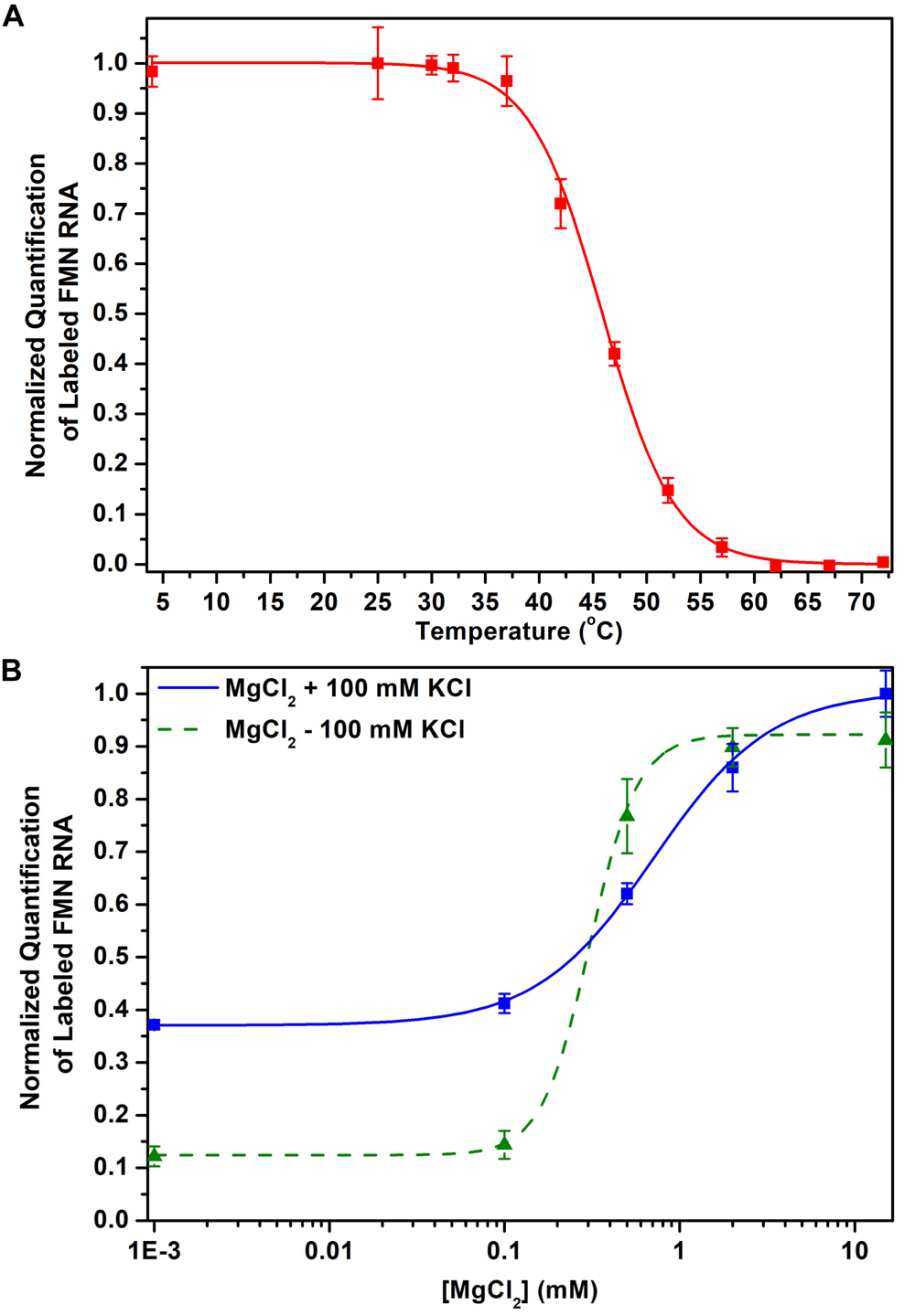
Measurements of FMN riboswitch folding with probe **1**. (A) A decrease in FMN riboswitch labeling is observed at increased temperatures. (B) FMN riboswitch labeling is highly dependent on cation concentration, which is attributed to potential (un)folding of the aptamer domain. Error bars represent standard deviations based on three technical replicates.

Since probe **1** exclusively reports on the riboswitch bound state, we aimed to exploit this feature to study folding of the riboswitch aptamer. To examine if probe **1** can measure changes in riboswitch conformations, we applied the probe to the FMN riboswitch under varied temperature and cation conditions. Probe **1** was incubated with the FMN riboswitch at temperatures in the range 4–72 °C and exposed to UV light to initiate photo-cross-linking. The amount of captured riboswitch was quantified using a click reaction with fluorescein azide and subsequent PAGE analysis (Figure S7 and see the SI for details). At increased temperature the amount of captured riboswitch decreased, which might be attributed to unfolding of the aptamer domain, with the corresponding midpoint at 46 ± 0.4 °C, using a sigmoidal fit (see the SI for details) (Figure 5A). The interaction between RNA and probe decreased above 37 °C (Figure 5A), and no significant interaction between the probe and riboswitch was observed above 62 °C, which might imply that the riboswitch was fully unfolded at this temperature (Figure 5A).^61^ These observations are in line with previously reported data.^44^ These experiments are conducted well below the reported activation temperature of diazirine photoreactive groups,^62^ yet slight differences in photochemistry cannot be excluded and should be taken into consideration.

To further explore structural changes with probe **1**, we focused on the influence of cation concentrations, which is known to affect riboswitch conformations.^61^ First, the MgCl_2_ concentration was reduced from 15 mM to 0 mM, resulting in an apparent decrease of fluorescently labeled riboswitch as observed by PAGE (Figures 5B and S8). A sharp decrease in labeled riboswitch was observed below 2 mM of MgCl_2_, with a [Mg^2+^]_1/2_ of 0.7 ± 0.06 mM calculated using a sigmoidal fit, which is in accordance with the literature value of 0.8 ± 0.1 mM.^44^ The presence of monovalent cations such as potassium can partially compensate for a lack of magnesium.^63^ To study this, we repeated the prior experiment in the presence of 100 mM KCl. We found increased riboswitch labeling compared to low magnesium concentrations with a [Mg^2+^]_1/2_ of 0.3 ± 0.02 mM, which could signal that potassium can partially assist riboswitch folding in the absence of magnesium (Figures 5B and S8). These experiments demonstrate the potential utility of directly measuring conditional riboswitch folding using photoaffinity probes.^44^

### Primer Extension of Labeled Transcripts

**Figure 6:**
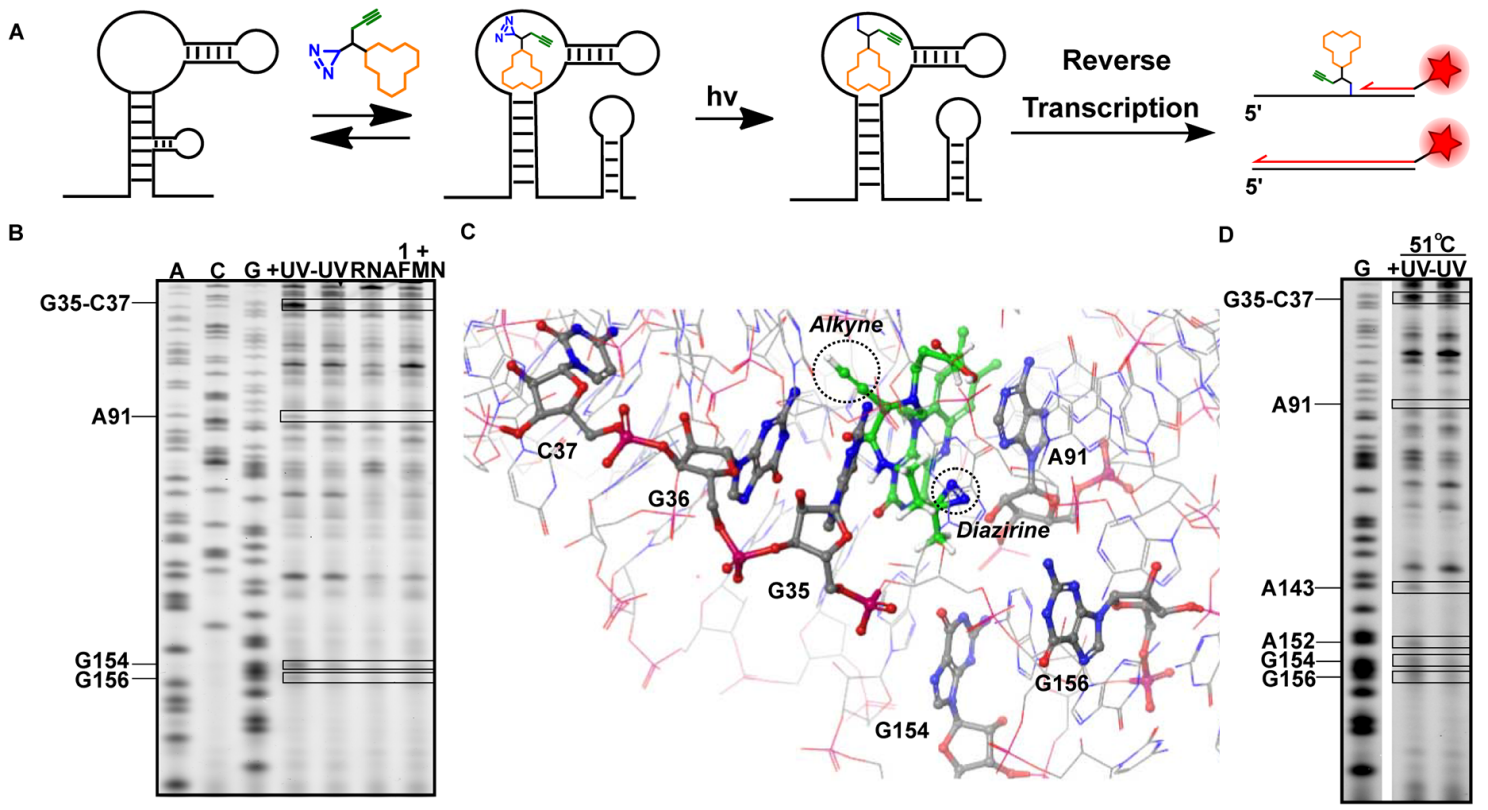
Structural insights in binding of probe **1** to the FMN riboswitch aptamer domain. (A). Schematic illustration of the reverse transcription (RT) termination assay. (B) PAGE gel after performing the RT termination assay. Boxed bands show termination sites only observed with probe treatment. (C) Docking of probe **1** in the FMN riboswitch aptamer of *Fusobacterium nucleatum* (PDB: 3F2Q)^36^ with observed labeled nucleotides annotated according to *B. subtilis* numbering. (D) PAGE analysis of the RT termination assay at increased temperature. Sequencing lanes were obtained by incorporating dideoxynucleotides. The “+UV” lane shows results with photo-cross-linking and “–UV” shows the control without photo-cross-linking. “RNA” is the control with untreated RNA. The “**1** + FMN” lane shows results of the termination assay in the presence of the competing FMN ligand. For triplicate experiments and quantification of band intensities see Figure S9.

We postulated that transforming transient binding interactions into covalent interactions could enable the identification of the binding site of the probe within the riboswitch with single-nucleotide resolution. After photo-cross-linking, primer extension can be performed with reverse transcriptase and a fluorescently labeled primer, which stops at the covalent bond of the probe to the riboswitch (Figure 6A).^55^ The exact nucleotide to which the probe is photo-cross-linked can then be deduced by performing sequence analysis using electrophoresis. To this end, 10 μM of probe **1** was incubated with 2 μM FMN riboswitch RNA in folding buffer at 37 °C for 30 min, after which UV irradiation was performed to photo-cross-link the probe to RNA. The RNA was purified and reverse transcription was performed (see the SI for further details).

New stops were observed at G35-C37, A91, G154, and G156 after photo-cross-linking of the probe to the FMN riboswitch (Figures 6B,C and S9). These stops were only observed after UV irradiation and are located in the aptamer domain of the FMN riboswitch.^36^ When the experiment was repeated in the presence of competing concentrations of natural ligand FMN, the RT stops disappeared, indicating that the observed interactions are specific (Figure 6B).

Nucleotides G35 and G36 are known to interact with the ribityl chain of FMN^36^ and are therefore expected to be in close proximity to the photoreactive diazirine group, explaining stops observed at G35-C37. Nucleotides G154 and G156 are positioned around the isoalloxazine ring of FMN in the ligand-bound state.^36^ The flexibility of the diazirine linker could potentially explain the appearance of stops at these positions. Interestingly, nucleotide A91 is reported to base-stack with A115, and the isoalloxazine ring of FMN intercalates between these two nucleotides.^36^ The stop observed at A91 is possibly a result of this interaction. Molecular docking of probe **1** into the FMN riboswitch showed that the probe is centered within a cluster of nucleotides at which the RT stops are observed (Figures 6C and S10).

To elucidate temperature-dependent changes in conformation, we repeated the experiment at 51 °C. Two additional stops were observed at A143 and A152 (Figures 6D and S9C). The uracil-like edge of the isoalloxazine ring system of the ligand forms specific Watson–Crick hydrogen bonds with the highly conserved nucleotide A152.^36^ No stop was observed at this position at 37 °C, which could mean that the elevated temperature is weakening this base-pair interaction. Interestingly, slight differences in SHAPE reactivity at this nucleotide have been previously observed at elevated temperature, but not below 65 °C.^44^ The additional stops observed at positions A143 and A152 could be the result of differences in flexibility of the ligand and FMN as well, positioning probe **1** and L6 (Figure 3A) of the riboswitch in closer proximity at elevated temperatures.

### Photoaffinity Labeling in Bacterial Extracts and Live Bacteria

**Figure 7:**
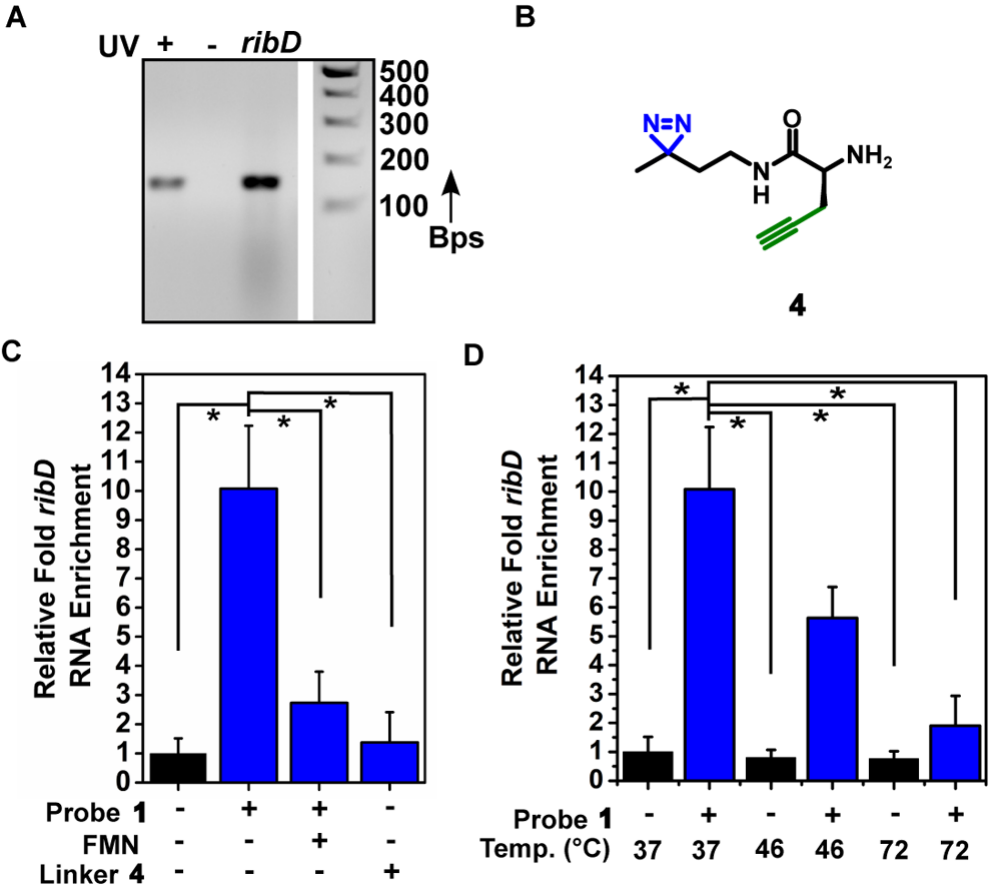
Photoaffinity labeling of *ribD* FMN riboswitch in *B. subtilis* extracts. (A) Agarose gel analysis of RT-PCR of enriched *ribD* mRNA using probe **1**. Amplification is observed only when probe **1** is activated by UV (+) and not without UV (−). (B) Molecular structure of control diazirine linker **4**. (C) Relative enrichment of *ribD* mRNA compared to the control (DMSO), in the presence of competing FMN, and with control diazirine linker **4**. (D) Enrichment of *ribD* mRNA at increasing temperatures. Error bars represent standard errors based on three biological replicates, each consisting of two technical replicates. Statistical significance was calculated using an unpaired two-tailed Student’s *t* test (*p < 0.05).

To show that we can determine ligand binding to the FMN riboswitch in endogenous samples, we first performed photoaffinity labeling on cell extracts from *B. subtilis* (see the SI for details). Extracts were incubated at 37 °C with 100 μM probe **1** and exposed to 365 nm light to initiate covalent labeling. Samples were incubated with biotin azide and a click mixture. Biotinylated RNA was isolated with Streptavidin magnetic beads and reverse transcribed. Using PCR with gene-specific primers for the *ribD* gene that is under control of the FMN riboswitch,^64^ successful labeling was demonstrated (Figure 7A). The amount of captured RNA was quantified using qPCR. Employing probe **1**, we found a 10-fold enrichment compared to a DMSO control, showing that we can selectively capture the *ribD* FMN riboswitch in bacterial extracts. When using control linker **4** (Figure 7B and C) instead of probe **1**, significantly lower amounts of *ribD* were enriched, indicating a high degree of selectivity provided by the flavin scaffold of probe **1**. To further demonstrate the selectivity and show the potential to measure binding of RNA modulators in cell extracts, we repeated the experiment in the presence of 100 μM competing FMN ligand. As expected, a significant decrease in enrichment compared to probe **1** alone was observed (Figure 7C). To investigate if we can potentially measure conditional folding of the FMN riboswitch in endogenous samples, we repeated the experiment at 46 and 72 °C. A temperature-dependent decrease in enriched *ribD* RNA when using probe **1** was observed compared to a negative control of DMSO only, which might potentially be explained by partial unfolding of the FMN riboswitch at higher temperatures (Figure 7D).

To assess the potential utility of photoaffinity labeling for measuring riboswitch binding *in vivo*, probe **1** was incubated with live *Escherichia coli* (*E. coli*) CS1562 containing a plasmid carrying the *E. coli ribB* riboswitch (pTXTL-sroGp2eGFP, see Figure S11 and the SI for details). This strain is efflux impaired to secure a sufficient intracellular probe concentration (Figure S12).^46^

**Figure 8:**
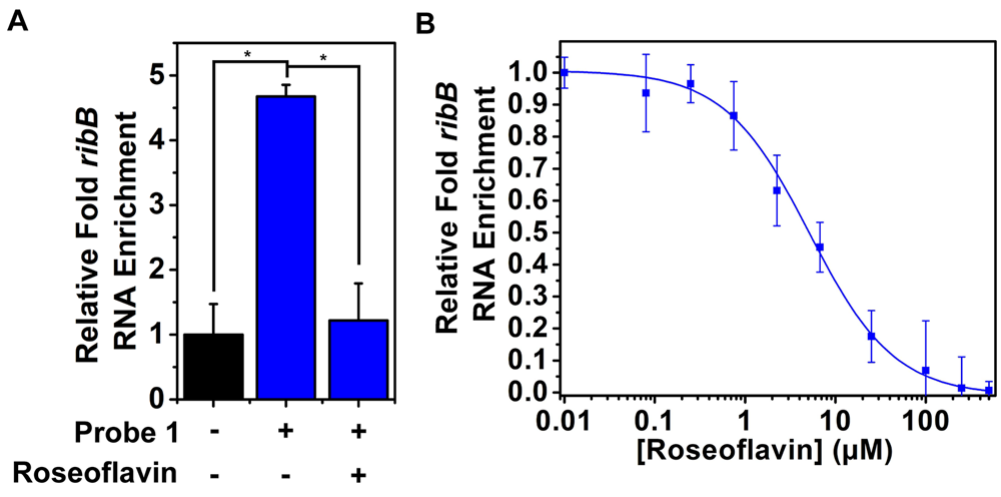
Photoaffinity labeling of *ribB* riboswitch in live *E. coli* CS1562 pTXTL-sroGp2eGFP. (A) Relative enrichment of *ribB* riboswitch RNA with probe **1** compared to the control (DMSO) and in the presence of competing roseoflavin (100 μM). (B) Dose-dependent inhibition of enrichment with roseoflavin in live *E. coli* CS1562 pTXTL-sroGp2eGFP. Error bars represent standard deviations based on three biological replicates, each consisting of two technical replicates. Statistical significance was calculated using an unpaired two-tailed Student’s *t* test (*p < 0.05).

In an initial experiment, 25 μM probe **1** was incubated for 30 min with bacteria and then irradiated to initiate cross-linking. Bacteria were lysed, and extracted RNA was exposed to biotin azide and a click mixture. After Streptavidin pulldown, enriched *ribB* riboswitch RNA was quantified by qPCR. A ∼5-fold enrichment compared to a DMSO control was observed (Figure 8A). To further analyze the selectivity of labeling and potential of measuring inhibitor binding *in vivo* with photoaffinity labeling, competition experiments with roseoflavin were performed. Bacteria were preincubated with increasing concentrations of roseoflavin, after which photoaffinity labeling was performed with probe **1**. A dose-dependent effect on enrichment was observed with an apparent IC_50_ of 5 ± 0.64 μM (Figure 8B). No decrease in enriched housekeeping gene (*cysG*) was observed at high concentrations of roseoflavin (Table. S1), supporting that the decrease in enrichment might be attributed to competitive binding to the riboswitch aptamer.

Together, these experiments demonstrate the possibility to measure riboswitch binding in biologically derived samples and could further assist in elucidating riboswitch properties and druggability.

## Conclusion

In summary, we have prepared photoaffinity probes to study the interaction between small-molecule ligands and riboswitches. We have applied our photoaffinity probe **1** to the FMN riboswitch and were able to selectively label the aptamer region of the riboswitch. The utility of the photoaffinity probe was demonstrated by quantitatively measuring the interaction between the riboswitch and small-molecule ligands, which we think might find applicability in drug discovery, as riboswitches make attractive antibiotic targets.^46,59,60^ By measuring riboswitch–ligand interactions at varied temperatures and cation concentrations, we showed the potential use of photoaffinity labeling for determining conditional riboswitch folding. Photoaffinity labeling yields a new addition to the riboswitch profiling toolbox, as it directly measures the interaction between ligand and RNA, unlike other profiling methods that indirectly observe altered RNA reactivity.^3,33,65^ Using reverse transcription and gel electrophoresis, we were able to determine the RNA binding site with single-nucleotide resolution. Interestingly, by conducting the experiment under elevated temperature conditions we observed altered ligand binding interactions that, to the best of our knowledge, have not been reported before using traditional RNA profiling methods. Lastly, by applying the photoaffinity probe to bacterial extracts and live bacteria, we were able to competitively measure ligand binding using bead enrichment and qPCR. We believe these experiments demonstrate the applicability of photoaffinity labeling to investigate riboswitch properties *in vitro* and in complex biological samples.^66^

## Supporting Information Available

Further experimental details, including PCR, *in vitro* transcription, affinity-based profiling, SHAPE assay, RT termination assay, bacterial growth, RNA extraction, qPCR, bacterial transformations, synthetic procedures, and spectral data.(PDF)
