# Supplementary material for: Affinity-Based Profiling of the Flavin Mononucleotide Riboswitch
Source: J Am Chem Soc. 2022 Jun 6;144(23):10462–70. doi: 10.1021/jacs.2c02685 (PMC9204756; doi:10.1021/jacs.2c02685)
Supplement: Supplementary file 1 — ja2c02685_si_001.pdf [file ja2c02685_si_001.pdf]

Supporting Information

Affinity-Based Profiling of the Flavin Mononucleotide Riboswitch

Stefan Crielaard, Rick Maassen, Tess Vosman, Ivy Rempkens and Willem A. Velema*
Institute for Molecules and Materials, Radboud University Nijmegen, Nijmegen, The Netherlands
*To whom correspondence should be addressed: willem.velema@ru.nl

TABLE OF CONTENTS

Supporting Figures S1-S13……………………………………p2
Supporting Table S1……………………………………p15
Experimental Section……………………………………p16
Biochemical Experiments……………………………………p16
Polymerase Chain Reaction……………………………………p16
In Vitro Transcription……………………………………p16
Affinity-Based Profiling of the FMN Riboswitch *In Vitro*……………………………………p17
Selective 2’ Hydroxyl Acylation analyzed by Primer Extension……………………………………p18
Reverse Transcription Termination Assay……………………………………p18
Molecular Docking of Probe **1** in the FMN Riboswitch……………………………………p19
Bacterial Growing Conditions……………………………………p19
Affinity-Based Profiling of the FMN Riboswitch from Bacterial Extracts……………………………………p19
Construction of pTXTL-sroGp2eGFP……………………………………p20
Transformation of pTXTL-sroGp2eGFP Plasmid in *E. Coli* CS1562……………………………………p22
Bacterial Growth Curves to Examine Cell Entrance of Flavin-Containing Compounds……………………………………p23
Affinity-Based Profiling of the FMN Riboswitch in Live Bacteria……………………………………p23
Synthesis, Purification and Analysis of Compounds……………………………………p24
Spectra……………………………………p36
References……………………………………p103

SUPPORTING FIGURES

Supporting figure S1A-C: Various probe binding concentrations

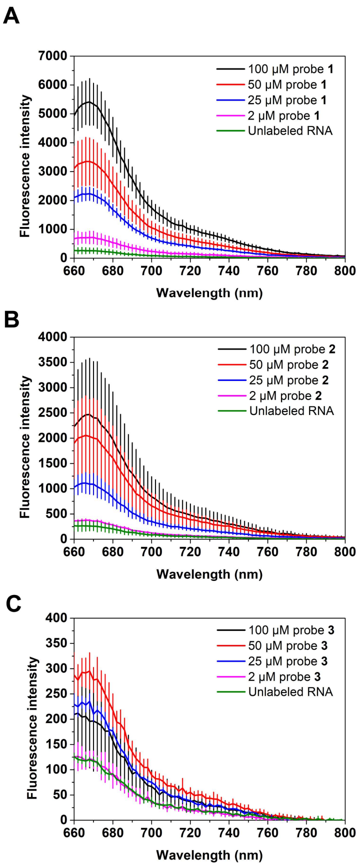

Supporting figure S1. Concentration-dependent labeling of FMN riboswitch RNA by photoreactive probes **1**, **2**, and **3**. Fluorescence intensity measurements of FMN RNA aptamer labeled with various concentrations of probe **1** (A), probe **2** (B) and probe **3** (C) and sequential modification with Cy5-azide. Error bars represent standard deviations based on three technical replicates.

Supporting figure S2A-B: Selective fluorescent labeling of the FMN riboswitch using photoaffinity labeling with probe **1**

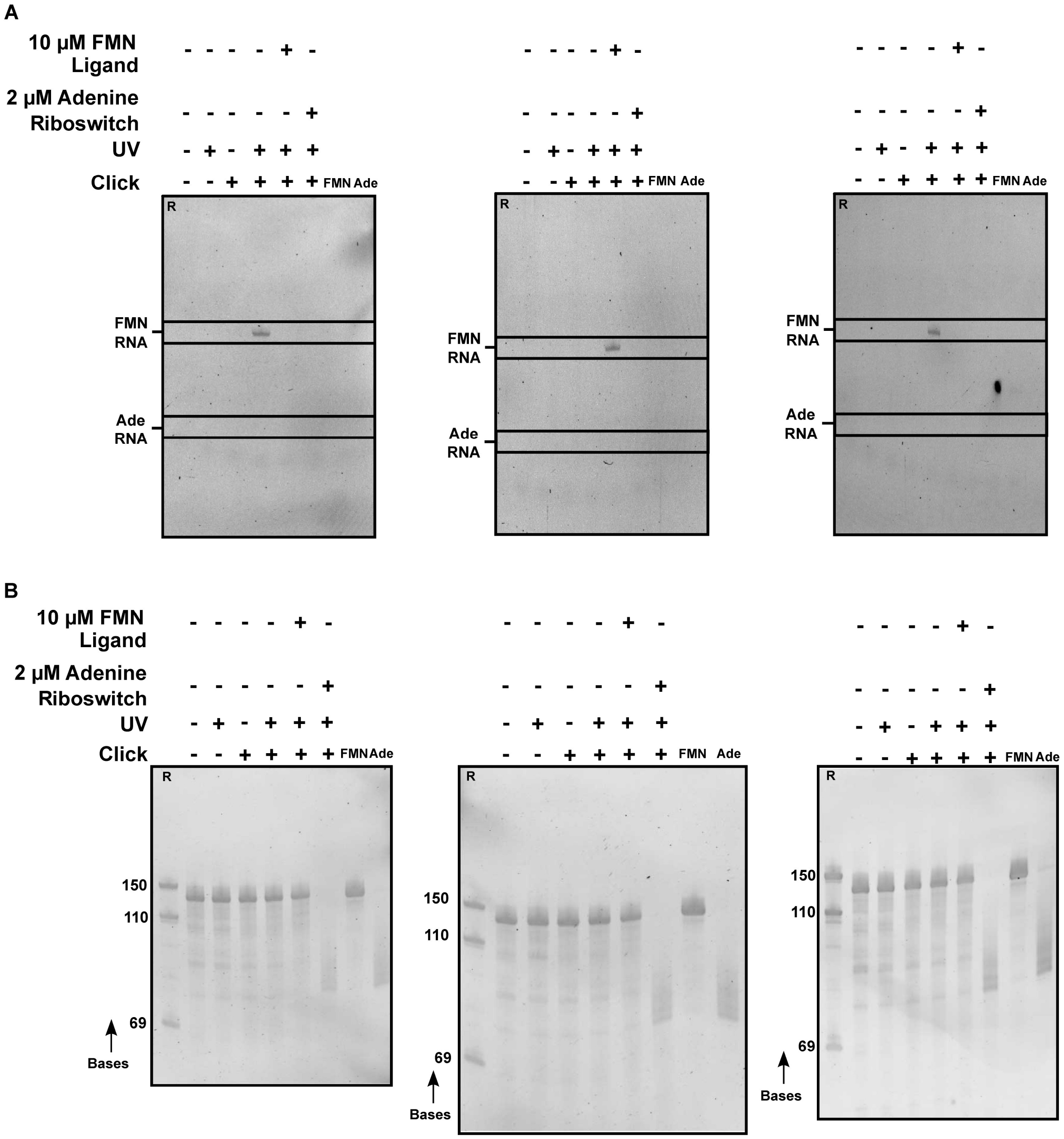

Supporting figure S2. The efficiency and selectivity of photoreactive probe **1**. (A) Fluorescence imaging of three denaturing RNA gels showing selective binding of probe **1** to the FMN RNA aptamer. (B) The same denaturing RNA gels after SYBR Gold staining to visualize all loaded RNA. 'R' is the reference ladder, which consists of three RNA sequences with known lengths. 'FMN' is untreated FMN RNA and 'Ade' stands for adenine riboswitch RNA.

Supporting figure S3A-B: SHAPE assay triplicate measurements

A

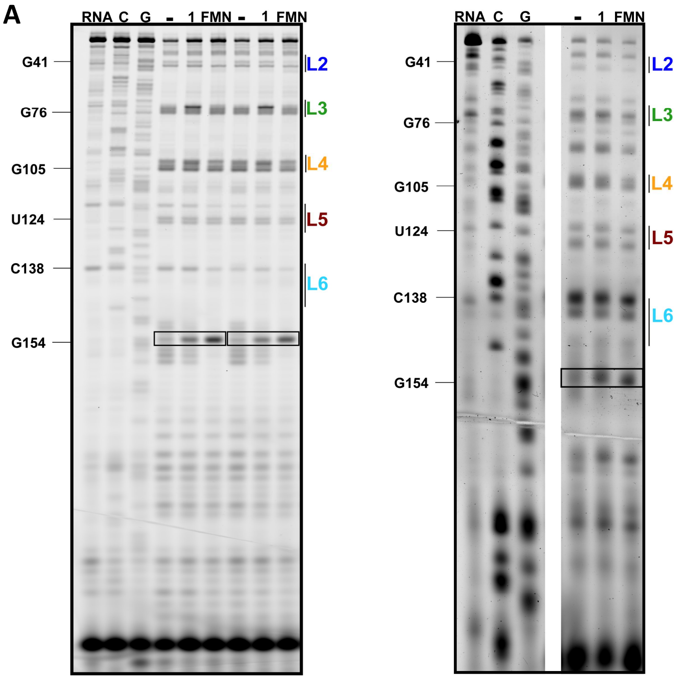

B

	Gel I		
**Nucleotides**	**Relative lane intensity '-'**	**Relative lane intensity '1'**	**Relative lane intensity 'FMN'**
**U42**	1.0	1.2	0.5
**U74**	1.0	1.9	1.1
**U102**	1.0	1.3	0.9
**U153**	1.0	2.0	3.1
**U124**	1.0	1.0	1.0
**C138**	1.0	1.0	0.8
	**Gel II**		
**Nucleotides**	**Relative lane intensity '-'**	**Relative lane intensity '1'**	**Relative lane intensity 'FMN'**
**U42**	1.0	0.9	0.5
**U74**	1.0	1.9	0.9
**U102**	1.0	1.1	0.8
**U153**	1.0	2.1	2.4
**U124**	1.0	1.0	0.9
**C138**	1.0	1.0	0.8
	**Gel III**		
**Nucleotides**	**Relative lane intensity '-'**	**Relative lane intensity '1'**	**Relative lane intensity 'FMN'**
**U42**	1.0	0.9	0.5
**U74**	1.0	1.0	0.6
**U102**	1.0	0.9	0.7
**U153**	1.0	1.7	1.9
**U124**	1.0	1.2	1.2
**C138**	1.0	0.9	0.8

Supporting figure S3. SHAPE assay of photoreactive probe **1**, natural ligand FMN and without ligand (indicated by '-'). (A) Fluorescence imaging of denaturing RNA sequencing gels *in triplo* scanned on a Typhoon Amersham at 488 nm excitation. The observed increased reactivity at U153 is highlighted as it is characteristic for ligand binding to the FMN riboswitch. The colored areas on the right side of the gel indicate for the different loops visualized in **figure 3A**. (B) Relative quantification of gel lanes that show variation between probe **1** and FMN (U42, U74, U102 and U153). The signals observed for U124 and C138 were also quantified relatively to correct for differences during gel loading before running the gel. On the left side of the gels are sequencing lanes included, obtained by incorporating dideoxynucleotides. Details about the quantification of the lanes can be found in section 'Selective 2' Hydroxyl Acylation analyzed by Primer Extension'.

Supporting figure S4A-D: Molecular docking of probe **1** and FMN in the FMN riboswitch

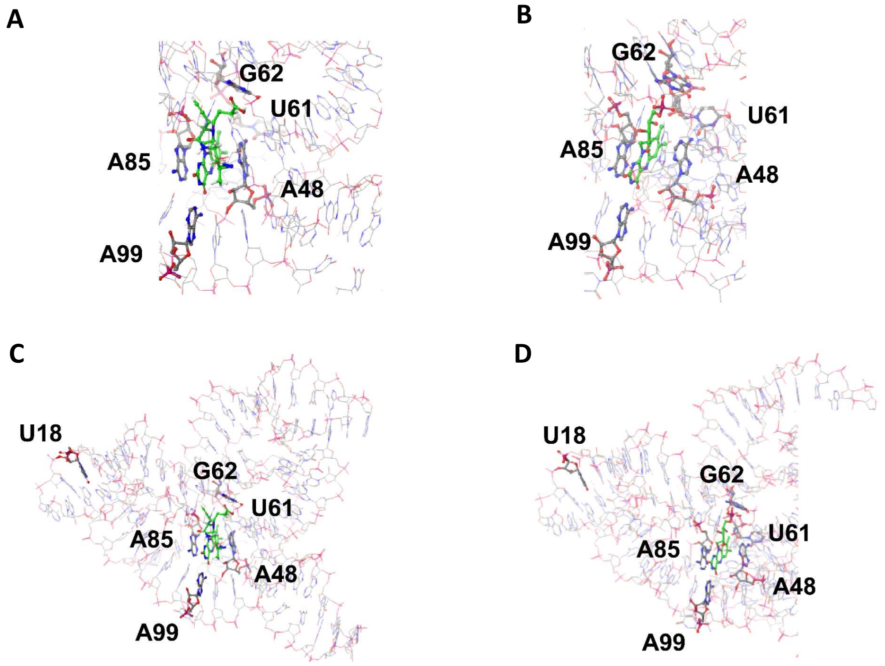

Supporting figure S4. Molecular docking of photoreactive probe **1** in the FMN riboswitch of *F. Nucleatum* (PDB ID: 3F2Q)[5]. (A) Molecular docking shows the positioning of probe **1** in the FMN riboswitch. The highlighted nucleotides are nucleotides commonly known to significantly interact with the FMN riboswitch.[5,7] (B) Binding of FMN in the FMN riboswitch as was determined by crystallography by Serganov *et al.* (2009)[5]. (C) Molecular docking shows the structure of the whole FMN riboswitch with U18 highlighted, in addition to the nucleotides highlighted in (A) and (B), which showed increased reactivity for acylation compared to FMN. (D) Full structure of the FMN riboswitch upon binding FMN with U18 highlighted, in addition to the nucleotides highlighted in (A) and (B).[5] U18 of the FMN riboswitch of *F. Nucleatum* is a conserved nucleotide and similar to U42 in the FMN riboswitch of *Bacillus Subtilis.*[7]

Supporting figure S5A-B: Gels FMN competition experiment

A

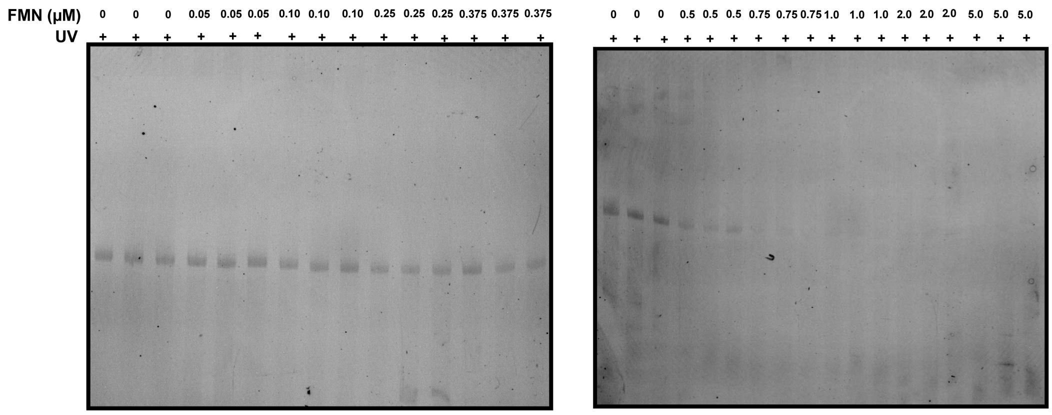

B

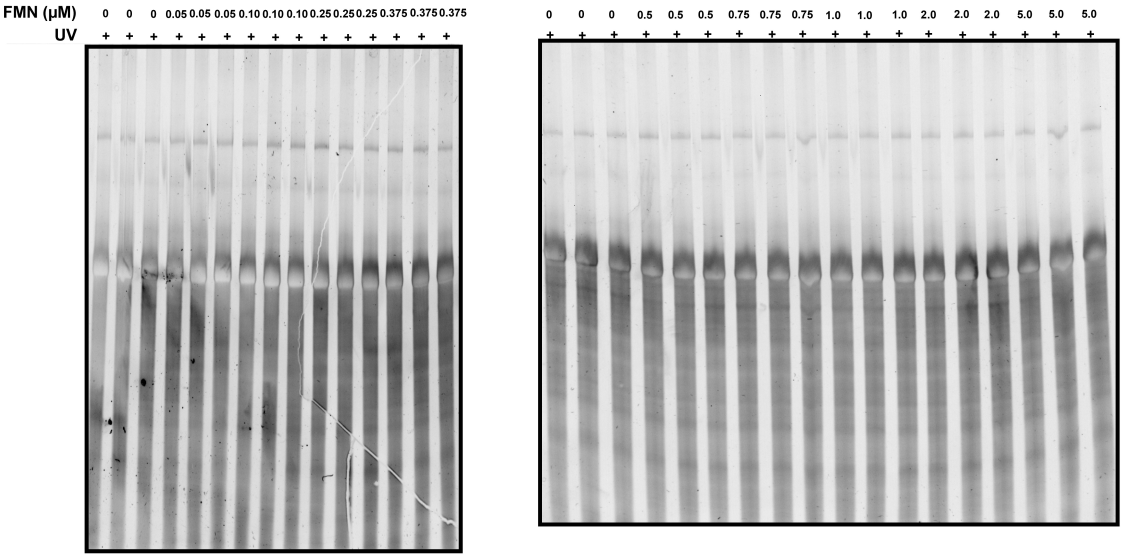

Supporting figure S5. Denaturing PAGE gels of labeling of the FMN riboswitch with probe **1** in the presence of competing concentrations of FMN. (A) Fluorescence imaging of the denaturing gels using fluorescein settings. (B) Fluorescence imaging of the denaturing gels after staining all loaded RNA in the gel with SYBR Gold.

Supporting figure S6A-B: Gels roseoflavin competition experiment

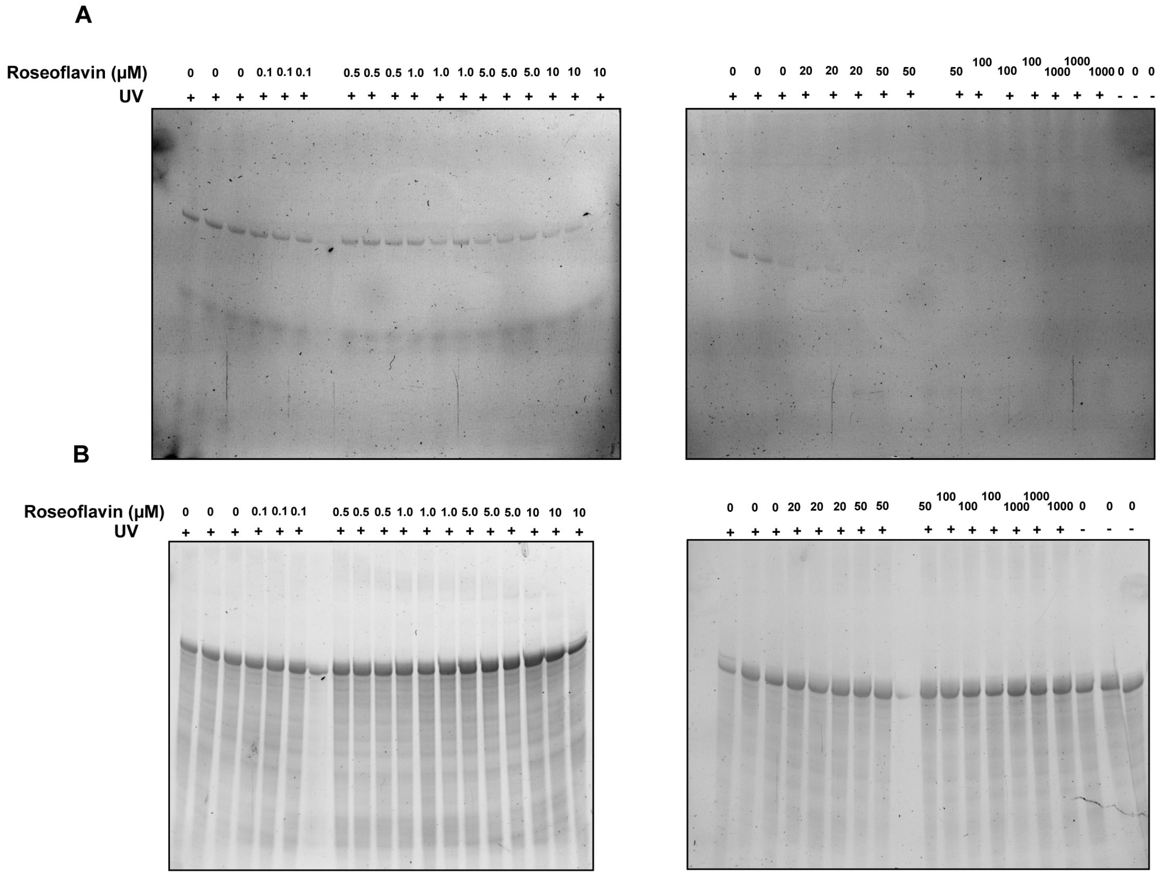

Supporting figure S6. Denaturing PAGE gels of labeling of the FMN riboswitch with probe **1** in the presence of competing concentrations of roseoflavin. (A) Fluorescence imaging of the denaturing gels using fluorescein settings. (B) Fluorescence imaging of the denaturing gels after staining all loaded RNA in the gel with SYBR Gold.

Supporting figure S7A-B: Gels temperature-dependent probe binding

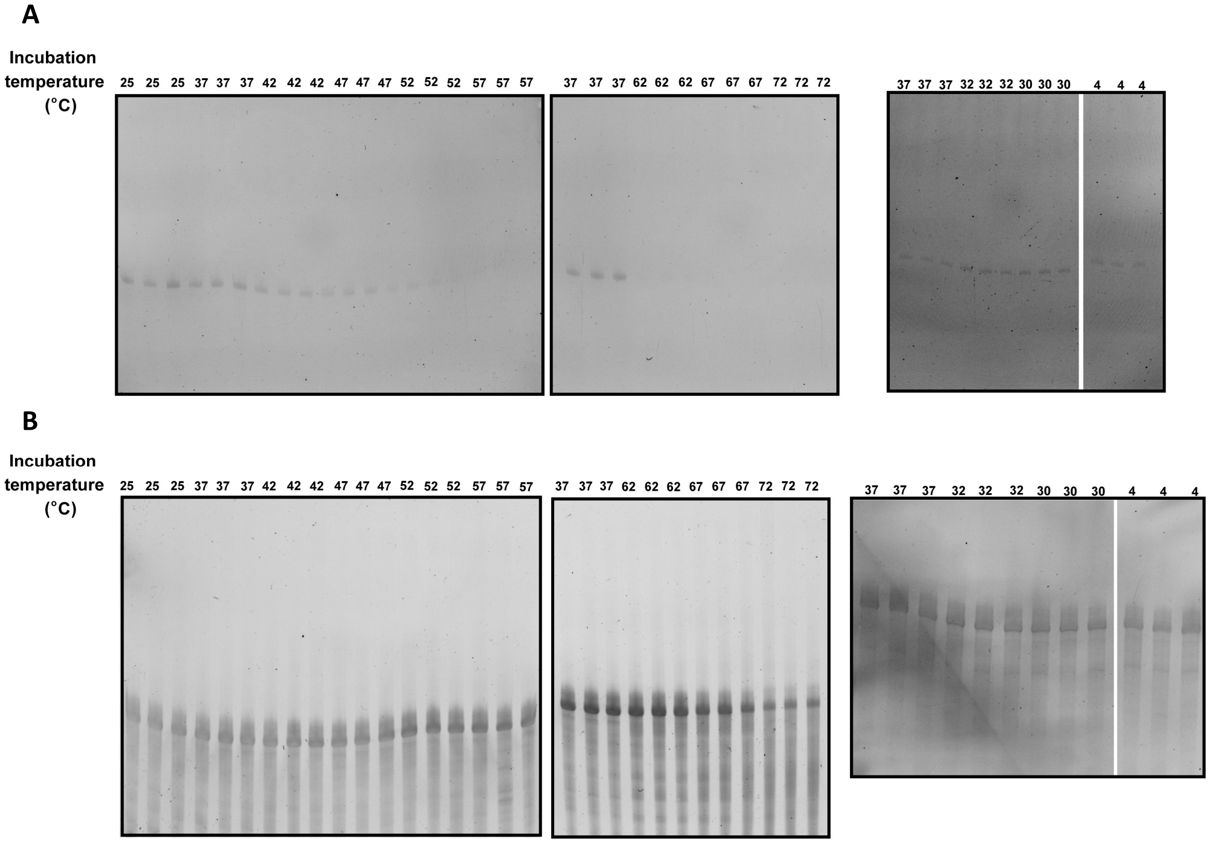

Supporting figure S7. Denaturing PAGE gels of the temperature-dependent labeling of probe **1** to the FMN riboswitch. (A) Fluorescence imaging of denaturing gels using fluorescein settings. (B) Fluorescence imaging of denaturing gels after staining all loaded RNA in the gel with SYBR Gold.

Supporting figure S8A-D: Gels $MgCl_2$-dependent probe binding

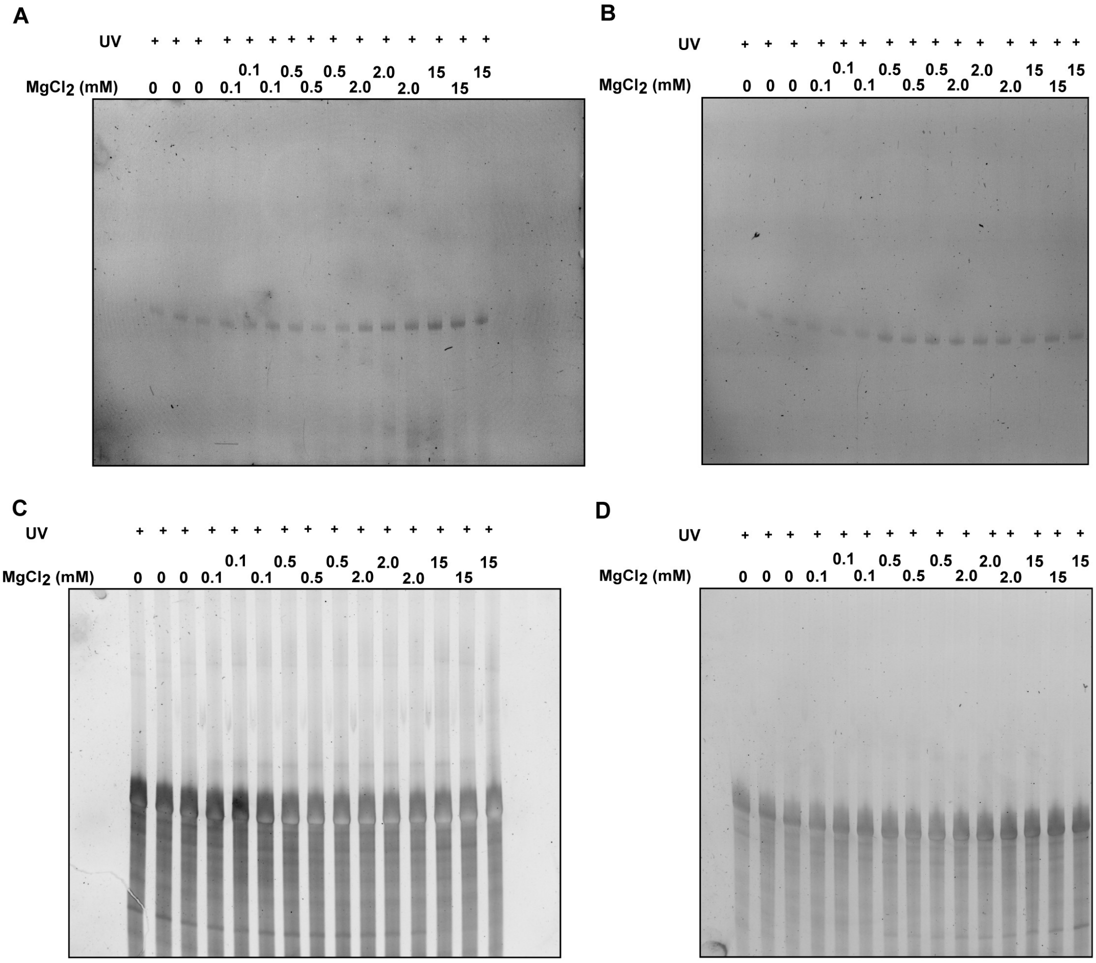

Supporting figure S8. Denaturing PAGE gels of the cation concentration-dependent labeling of probe **1** to the FMN riboswitch. (A) Fluorescence imaging of denaturing RNA gel of the magnesium-dependent labeling of probe **1** in the presence of 100 mM KCl using fluorescein settings. (B) Fluorescence imaging of the denaturing RNA gel of magnesium-dependent binding of probe **1** in the absence of KCl using fluorescein settings. (C) Fluorescence imaging of denaturing RNA gel of the magnesium-dependent binding of probe **1** in the presence of 100 mM KCl after RNA staining using SYBR Gold. (D) Fluorescence imaging of denaturing RNA gel of the magnesium-dependent binding of probe **1** in the absence of KCl after RNA staining using SYBR Gold.

Supporting figure S9A-C: Reverse transcription termination assay triplicate measurements

A

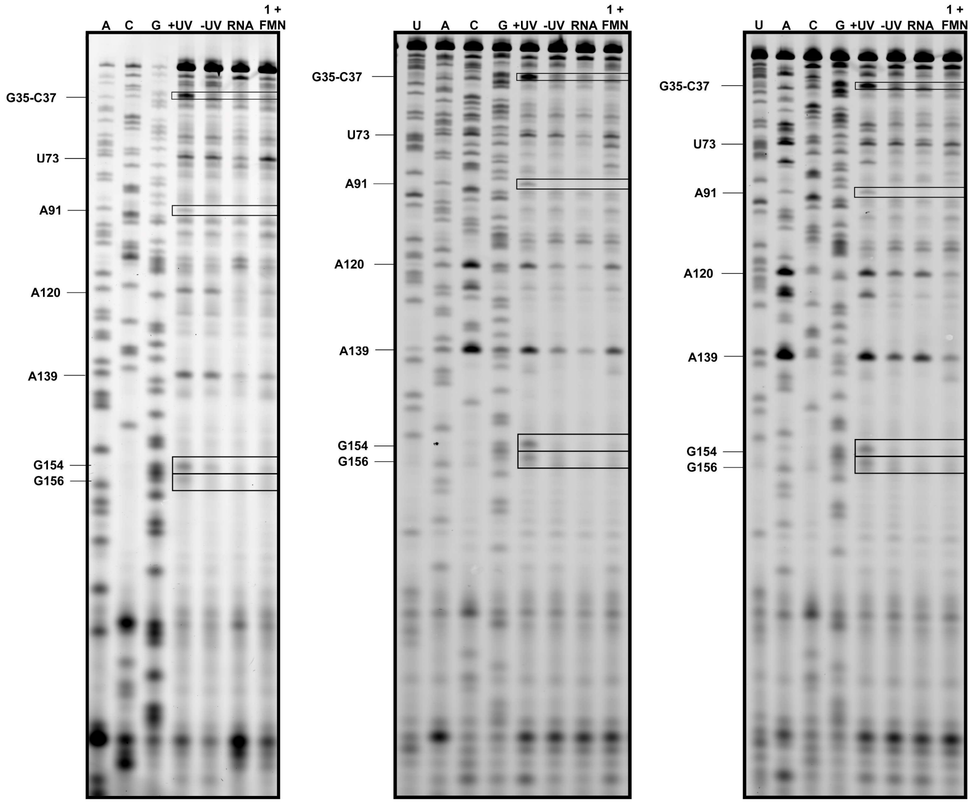

B

	Relative Lane Intensity Gel I		Relative Lane Intensity Gel II		Relative Lane Intensity Gel III	
**Nucleotides**	**+UV**	**-UV**	**+UV**	**-UV**	**+UV**	**-UV**
**G35-C37**	2.2	1.0	3.2	1.0	3.0	1.0
**A91**	2.3	1.0	4.0	1.0	3.5	1.0
**G154**	2.4	1.0	3.9	1.0	3.6	1.0
**G156**	1.7	1.0	2.0	1.0	2.9	1.0
**U73**	1.1	1.0	1.3	1.0	1.2	1.0
**A120**	1.0	1.0	1.9	1.0	1.7	1.0
**A139**	1.0	1.0	2.0	1.0	1.7	1.0

C

	Relative Lane Intensity Gel Figure 6D	
**Nucleotides**	**+UV**	**-UV**
**G35-C37**	1.5	1.0
**A91**	1.7	1.0
**A143**	2.0	1.0
**A152**	1.9	1.0
**G154**	1.6	1.0
**G156**	1.7	1.0
**U73**	0.8	1.0
**A120**	0.8	1.0
**A139**	0.8	1.0

Supporting figure S9. Reverse transcription termination assay to localize binding of probe **1** in the FMN riboswitch. (A) Fluorescence imaging of denaturing RNA sequencing gels *in triplo* scanned on a Typhoon Amersham at 488 nm excitation. Additional stops in the '+UV' samples compared to the control samples are highlighted and indicate for probe photocrosslinking sites to the FMN riboswitch. On the left side of the gels are sequencing lanes included, obtained by incorporating dideoxynucleotides. '+UV' lane shows results with photocrosslinking and '-UV' shows the control without photocrosslinking. 'RNA' is control with untreated RNA. '**1** + FMN' lane shows results of termination assay in presence of competing FMN ligand. (B) and (C) Relative quantification of the highlighted gel lanes that show enhanced signal in the '+UV' lanes in supporting figure S9A and figure 6D respectively. The signals observed for U73, A120 and A139 were also included to correct for differences during gel loading before running the gel. Details about the quantification of the lanes can be found in section 'Reverse Transcription Termination Assay'.

Supporting figure S10A-B: Highlighting the photocrosslinking sites of probe **1** in the FMN riboswitch using molecular docking

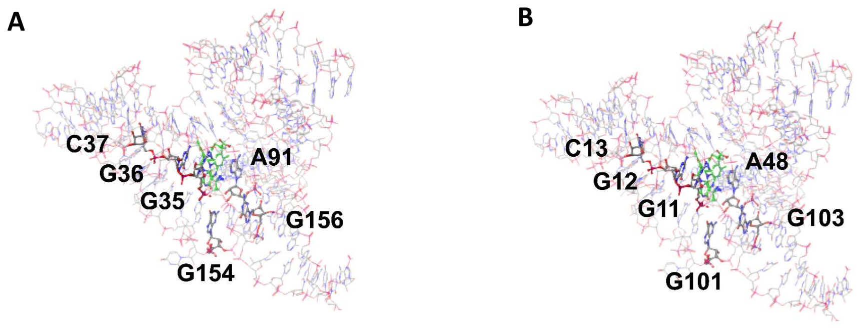

Supporting figure S10. Molecular docking of photoreactive probe **1** in the FMN riboswitch of *F. Nucleatum* (PDB ID: 3F2Q)[5] with the photocrosslinked nucleotides highlighted. (A) Molecular docking shows the structure of the full FMN riboswitch structure upon interacting with probe **1**. The highlighted nucleotides are the nucleotides that are photocrosslinked by the photoreactive probe during the reverse transcription termination assay. The highlighted nucleotides are numbered using the *Bacillus Subtilis* FMN riboswitch sequence.[7] (B) Same as in (A), but the highlighted nucleotides are numbered using the *F. Nucleatum* FMN riboswitch sequence. The photocrosslinked nucleotides are all positioned in the conserved binding region of the aptamer domain.[7]

Supporting figure S11A-C: Transformation of pTXTL-sroGp2eGFP in *Escherichia Coli* CS1562

A

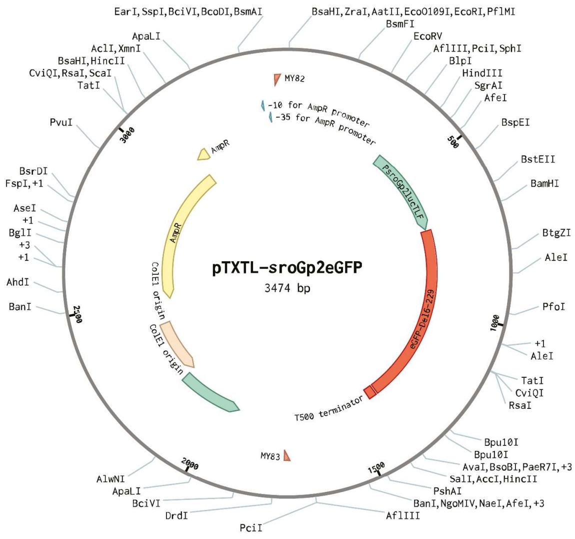

B

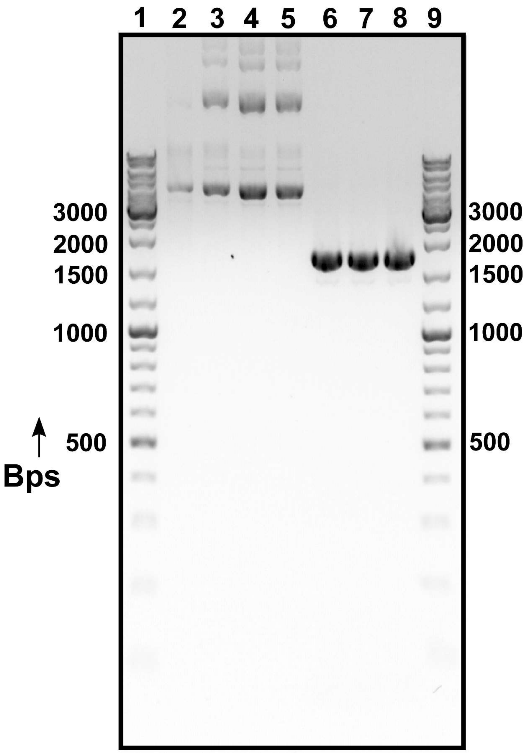

C

5’ GGGGGGCWACGATATCCGCCTGATGCGTGAACGTGACGGACGTAACCACCGCGACATGTGTGTGCTGTTCCGCTGGGCAT GCCAGGACAACTTCTGGTCCGGTAACGTGCTGAGCGGCGTTGCTGCCGCTAATCATTAGCGTTATAGTGAATCCGCTTAAAGCT TGCTTATTCTCAGGGCGGGGCGAAATTCCCCACCGGCGGTAAATCAACTCAGTTGAAAGCCCGCGAGCGCTTTGGGTGCGAAC TCAAAGGACAGCAGATCCGGTGTAATTCCGGGGCCGACGGTTAGAGTCCGGATGGGAGAGAGTAACGATTCTGTCGGGCATG GACCCGCTCACGTTATTTTGGCTATATGCCGCCACTCCTAAGACTGCCCTGATTCTGGTAACCATAATTTTAGTGAGGTTTTTTA CCATGAATCAGACGCTACTTTGGATCCAAGCAGAGTTGGAGCTTTTCACTGGCGTTGTTCCCATCCTGGTCGAGCTGGACGGCG ACGTAAACGGCCACAAGTTCAGCGTGTCCGGCGAGGGCGAGGGCGATGCCACCTACGGCAAGCTGACCCTGAAGTTCATCTG CACCACCGGCAAGCTGCCCGTGCCCTGGCCCACCCTCGTGACCACCCTGACCTACGGCGTGCAGTGCTTCAGCCGCTACCCCGA CCACATGAAGCAGCACGACTTCTTCAAGTCCGCCATGCCCGAAGGCTACGTCCAGGAGCGCACCATCTTCTTCAAGGACGACG GCAACTACAAGACCCGCGCCGAGGTGAAGTTCGAGGGCGACACCCTGGTGAACCGCATCGAGCTGAAGGGCATCGACTTCAA GGAGGACGGCAACATCCTGGGGCACAAGCTGGAGTACAACTACAACAGCCACAACGTCTATATCATGGCCGACAAGCAGAAG AACGGCATCAAGGTGAACTTCAAGATCCGCCACAACATCGAGGACGGCAGCGTGCAGCTCGCCGACCACTACCAGCAGAACA CCCCCATCGGCGACGGCCCCGTGCTGCTGCCCGACAACCACTACCTGAGCACCCAGTCCGCCCTGAGCAAGACCCCAACGAGA AGCGCGATCACATGGTCCTGCTG 3’

Supporting figure S11. Transformation efficiency of pTXTL-sroGp2eGFP in *E. Coli* CS1562. (A) Construct of plasmid pTXTL-sroGp2eGFP. (B) 2% agarose gel of the transformed plasmid and the PCR product of the plasmid to verify the plasmid sequence. The gels were stained with 50 µg ethidium bromide. **1** Reference ladder with basepair sizes. **2** Result after DNA isolation after the first transformation in *E. Coli* strain TOP10F (expected size of the plasmid: 3474 bps). **3**-**5** DNA isolation of three different bacterial cultures after the second transformation in *E. Coli* CS1562 (expected size of the plasmid: 3474 bps). **6**-**8** PCR of the obtained plasmids of the three different bacterial cultures (expected size of the amplicons: 1782 bps). **9** Reference ladder with basepair sizes. (C) Sanger sequencing result of part of the plasmid including the FMN riboswitch with similar sequence as the FMN riboswitch encoded in *ribB.* This sequence was obtained after both performed transformations.

Supporting Figure S12. Bacterial growth curves showing cell entrance of flavin-containing compounds in *E. Coli* CS1562 pTXTL-sroGp2eGFP

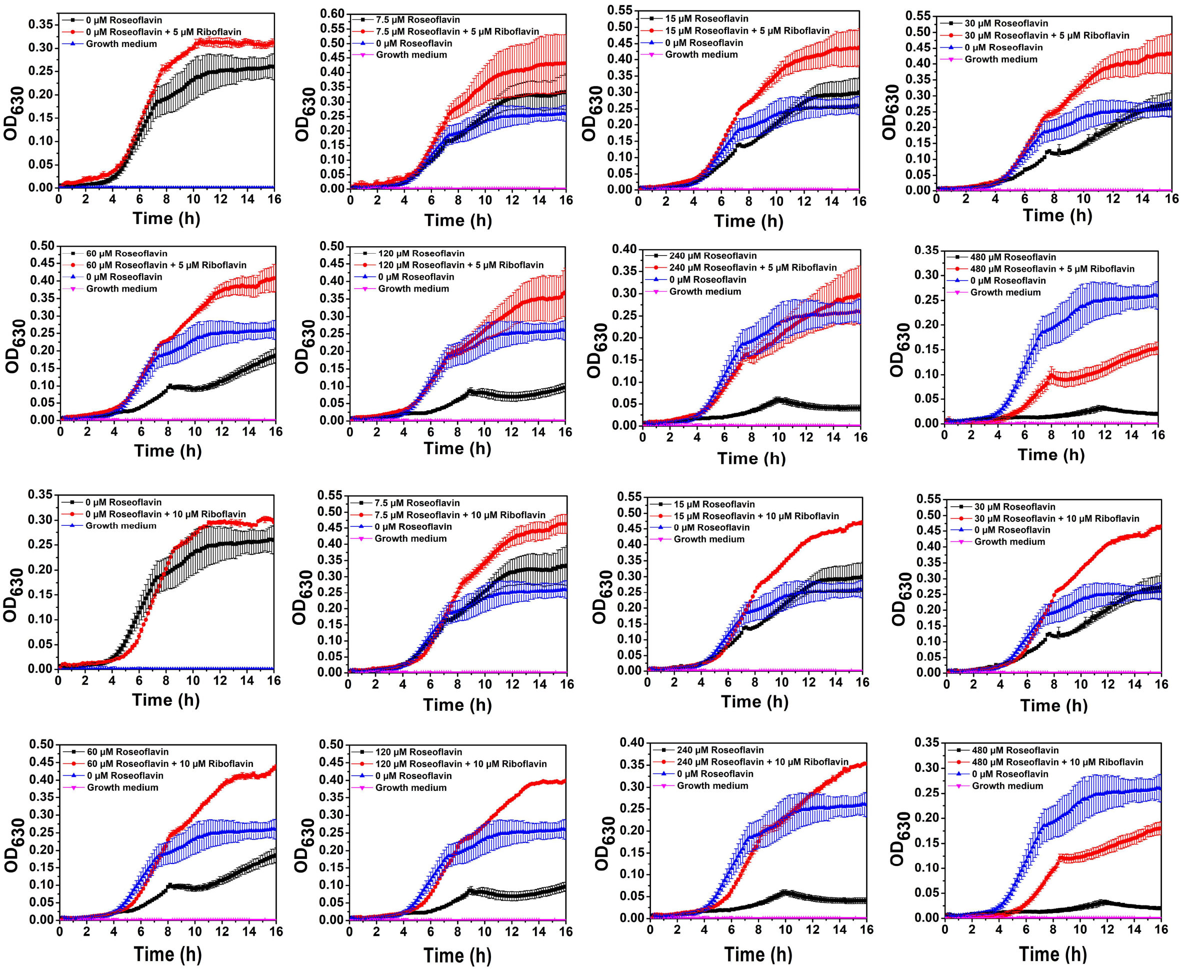

Supporting Figure S12. To demonstrate that flavin-containing compounds can enter the bacterial strain *E. coli* CS1562 pTXTL-sroGp2eGFP, a series of bacterial growth experiments was conducted as described earlier.[15] Bacteria were grown in the presence of the flavin containing antibiotic roseoflavin. Bacterial growth was significantly slowed down at concentrations of 30 μM and higher. Roseoflavin is reported to interact with the FMN riboswitch and interfere with riboflavin biosynthesis. Supplying riboflavin to the bacteria can therefore counteract roseoflavin's effect.[15] We conducted similar experiments and indeed observed that the growth inhibitory effect was attenuated in the presence of 5 μM riboflavin. Importantly, this implies that flavin-containing compounds can enter *E. coli* CS1562 pTXTL-sroGp2eGFP at low micromolar concentrations as is also observed in our photoaffinity labeling experiments in live bacteria. Error bars represent standard deviations based on three technical replicates.

Supporting figure S13A-B: Synthesis of the FMN riboswitch RNA

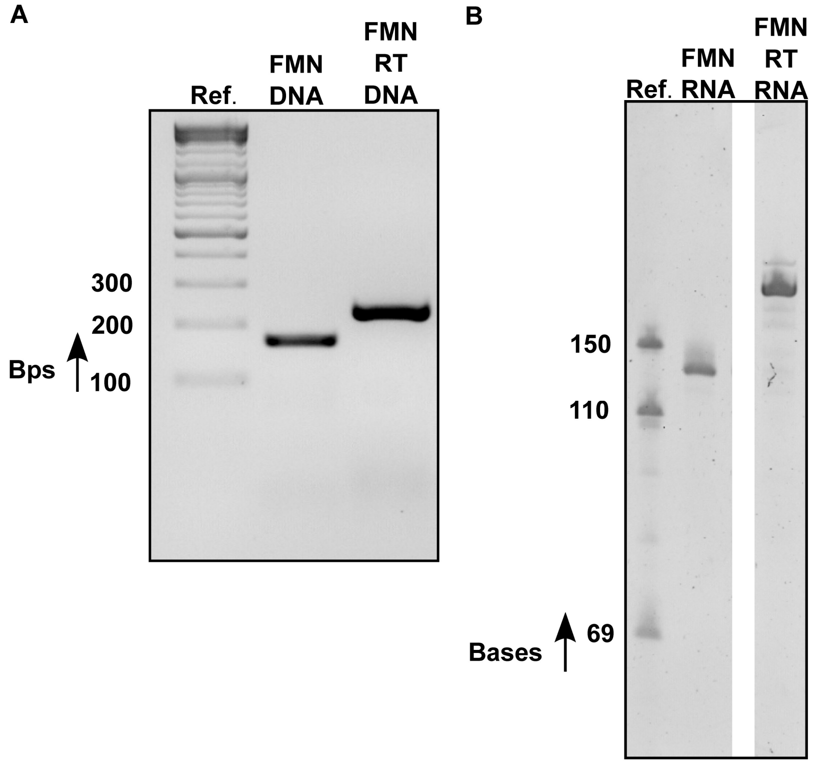

Supporting figure S13. Synthesis of the FMN riboswitch aptamer. (A) 2% agarose gel of the PCR product to check the purity of the amplified DNA sequences. The gels were stained with 50 µg ethidium bromide. 'FMN DNA' is expected to be 167 nucleotides. 'FMN RT DNA' is expected to be 228 nucleotides. (B) Urea-PAGE gel to check the purity of the obtained RNA after *in vitro* transcription of the amplified DNA sequences. 'FMN RNA' is expected to be 147 nucleotides and 'FMN RT RNA' is expected to be 202 nucleotides. 'FMN RT DNA' and 'FMN RT RNA' encode for the FMN riboswitch sequence inserted in a structure cassette for SHAPE mapping and RT pausing assays. 'Ref' stands for reference ladder. 'Bps' stands for basepairs.

Supporting table S1: Quantitative PCR cycle threshold data of the relative fold enrichment of *ribB* in the presence of different concentrations of roseoflavin

Sample	House Keeping Gene (*cysG*)				Gene of Interest (*ribB*)			
	**Ct1**	**Ct2**	**Mean Ct**	**St. dev.**	**Ct1**	**Ct2**	**Mean Ct**	**St. dev.**
RNA extract	13.5	13.4	13.4	0.0	6.1	6.1	6.1	0.0
RNA extract	13.4	13.7	13.5	0.0	6.0	6.1	6.0	0.0
RNA extract	13.8	13.5	13.6	0.1	6.1	6.0	6.1	0.0
25 µM **1**	30.5	29.8	30.2	0.2	16.3	16.1	16.2	0.0
25 µM **1**	31.0	31.1	31.1	0.0	17.1	17.1	17.1	0.0
25 µM **1**	29.9	30.6	30.3	0.3	16.1	16.3	16.2	0.0
25 µM **1** + 0.08 µM roseoflavin	30.1	30.5	30.3	0.1	16.5	16.1	16.3	0.1
25 µM **1** + 0.08 µM roseoflavin	31.0	29.7	30.3	0.8	16.3	16.2	16.3	0.0
25 µM **1** + 0.08 µM roseoflavin	30.3	29.7	30.0	0.1	16.2	16.2	16.2	0.0
25 µM **1** + 0.25 µM roseoflavin	30.1	30.2	30.2	0.0	16.2	16.0	16.1	0.0
25 µM **1** + 0.25 µM roseoflavin	30.3	29.7	30.0	0.2	16.1	16.1	16.1	0.0
25 µM **1** + 0.25 µM roseoflavin	30.6	30.5	30.5	0.0	16.6	16.6	16.6	0.0
25 µM **1** + 0.75 µM roseoflavin	30.3	30.2	30.3	0.0	16.5	16.7	16.6	0.0
25 µM **1** + 0.75 µM roseoflavin	29.5	29.4	29.4	0.0	15.5	15.6	15.5	0.0
25 µM **1** + 0.75 µM roseoflavin	30.3	30.4	30.3	0.0	16.3	16.4	16.4	0.0
25 µM **1** + 2.25 µM roseoflavin	29.3	30.0	29.6	0.2	16.2	16.4	16.3	0.0
25 µM **1** + 2.25 µM roseoflavin	30.0	29.7	29.9	0.1	16.3	16.1	16.2	0.0
25 µM **1** + 2.25 µM roseoflavin	29.7	30.0	29.9	0.1	16.5	16.2	16.3	0.1
25 µM **1** + 6.75 µM roseoflavin	29.4	29.3	29.4	0.0	16.1	16.1	16.1	0.0
25 µM **1** + 6.75 µM roseoflavin	29.7	29.3	29.5	0.1	16.3	16.1	16.2	0.0
25 µM **1** + 6.75 µM roseoflavin	30.0	29.2	29.6	0.3	16.9	16.3	16.6	0.2
25 µM **1** + 25 µM roseoflavin	29.2	29.7	29.4	0.1	16.9	16.5	16.7	0.1
25 µM **1** + 25 µM roseoflavin	29.1	28.7	28.9	0.1	16.8	16.6	16.7	0.0
25 µM **1** + 25 µM roseoflavin	29.3	29.4	29.4	0.0	16.9	16.7	16.8	0.0
25 µM **1** + 100 µM roseoflavin	31.0	30.6	30.8	0.1	18.1	18.5	18.3	0.1
25 µM **1** + 100 µM roseoflavin	30.6	30.6	30.6	0.0	18.5	18.3	18.4	0.0
25 µM **1** + 100 µM roseoflavin	30.6	30.9	30.7	0.0	19.4	19.0	19.2	0.1
25 µM **1** + 250 µM roseoflavin	31.5	31.0	31.2	0.1	18.6	18.9	18.8	0.0
25 µM **1** + 250 µM roseoflavin	30.1	30.0	30.1	0.0	18.5	18.1	18.3	0.1
25 µM **1** + 250 µM roseoflavin	29.1	29.2	29.2	0.0	18.1	18.5	18.3	0.1
25 µM **1** + 500 µM roseoflavin	30.4	30.5	30.4	0.0	18.5	18.3	18.4	0.0
25 µM **1** + 500 µM roseoflavin	30.7	29.4	30.1	0.8	18.3	18.4	18.4	0.0
25 µM **1** + 500 µM roseoflavin	30.4	29.6	30.0	0.3	18.4	18.2	18.3	0.0
DMSO	28.4	28.3	28.4	0.0	16.6	17.5	17.0	0.4
DMSO	29.1	28.6	28.8	0.1	17.5	17.6	17.5	0.0
DMSO	29.9	29.8	29.8	0.0	17.1	17.7	17.4	0.2

Supporting table S1. Quantitative PCR cycle threshold data of the relative fold enrichment of *ribB* in the presence of different concentrations of roseoflavin. Biological triplicates were performed, each consisting of internal duplicate measurements.

EXPERIMENTAL SECTION

BIOCHEMICAL EXPERIMENTS

All reagents described below were received from commercial suppliers and were used according to manufacturer's instructions. Oligonucleotides were purchased from Integrated DNA Technology (IDT). *Bacillus Subtilis subspecies subtilis* strain 168 (BGSCID: 1A1) was obtained from *Bacillus Subtilis* Stock Center (Ohio State University). *Escherichia Coli* CS1562 (CGSC#: 6911) was a gift from Prof. Arnold Driessen, University of Groningen.

Polymerase Chain Reaction

The DNA templates to produce the corresponding RNA were amplified using polymerase chain reaction (PCR) (**Supporting Table S2**). The FMN riboswitch was inserted in a structure cassette for reverse transcription experiments[1]. Both strands contain a T7 promotor sequence for RNA synthesis purposes. For PCR, 200 µL reaction mixes were prepared, each containing: Phusion HF buffer (1x, Thermo Fisher Scientifc); dNTPs (0.2 mM, Jena Bioscience); forward and reverse primer (both 1.0 µM); DNA template (10 ng); Phusion DNA polymerase (4 units, prepared in house). Conditions for the PCR amplification were as follows: 95 °C for 120 s (1 cycle); 95 °C for 30 s, 50 °C for 30 s, 72 °C for 120 s (18 cycles); 72 °C for 300 s (1 cycle) in a TProfessional thermal cycler (Biometra). The PCR efficiency was checked using agarose gel electrophoresis (2%) and ethidium bromide staining (50 µg). The gels were scanned using a GelDoc (BioRad) on ethidium bromide settings. The DNA was purified using the QIAquick PCR Purification Kit (Qiagen) (**Supporting figure S13A**). The obtained DNA concentrations were determined using NanoDrop.

**Indicated DNA strand**	**Sequence**
FMN aptamer DNA strand	5' CGG TAA TAC GAC TCA CTA TAG GGT GTA TCC TTC GGG GCA GGG TGG AAA TCC CGA CCG GCG GTA GTA AAG CAC ATT TGC TTT AGA GCC CGT GAC CCG TGT GCA TAA GCA CGC GGT GGA TTC AGT TTA AGC TGA AGC CGA CAG TGA AAG TCT GGA TGG GAG AAG GAT GA 3'
Forward primer FMN DNA strand	5' CGG TAA TAC GAC TCA CTA TAG GGT 3'
Reverse primer FMN DNA strand	5' TCA TCC TTC TCC CAT CCA GAC TTT 3'
FMN RT DNA strand	5' GCG GAA TTC TAA TAC GAC TCA CTA TAG GGC CTT CGG GCC AAT GTA TCC TTC GGG GCA GGG TGG AAA TCC CGA CCG GCG GTA GTA AAG CAC ATT TGC TTT AGA GCC CGT GAC CCG TGT GCA TAA GCA CGC GGT GGA TTC AGT TTA AGC TGA AGC CGA CAG TGA AAG TCT GGA TGG GAG AAG GAT GAT CGA TCC GGT TCG CCG GAT CCA AAT CGG GCT TCG GTC CGG TTC 3'
Forward primer FMN RT DNA strand	5' GCG GAA TTC TAA TAC GAC TCA CTA TA 3'
Reverse primer FMN RT DNA strand	5' GAA CCG GAC CGA AGC CCG 3'

Supporting table S2. DNA sequences and the corresponding forward and reverse primer used during PCR.

In Vitro Transcription

Transcription proceeded in 0.5-2.0 mL reaction mixes for four hours containing: Tris.HCl (40 mM, pH 8.1); magnesium chloride (25 mM); spermidine (1 mM); dithiotreitol (5 mM); rNTPs (4 mM, Jena Bioscience); Guanosine-5'-monophosphate (5 mM); T7 RNA polymerase reaction mixture (20% (v/v), prepared in house); DNA template (25 nM). After this reaction, DNA was removed by adding DNAse I (2 U/µg DNA, New England Biolabs) and incubating at 37 °C for 30 minutes. RNA purification was

performed on ice. Ethylenediaminetetraacetic acid (30 mM) was added to quench the enzymes. Q Sepharose high performance resin (GE healthcare) was used to purify the RNA. Beads were washed with Tris-EDTA buffer (25 mM Tris.HCl, pH 8.1 + 1 mM EDTA). RNA was incubated with the beads at 37 °C for 10 minutes. Consequently, the beads were pelleted (RT, 17.0 g, 5 min) and washed with Tris-EDTA buffer (three times). RNA was eluted twice by adding NaCl (1.5 M, 200 µL) and using RNase-free purification column filters. The RNA was precipitated by performing an isopropanol precipitation overnight and stored in nuclease-free water at -20 °C. RNA purity was checked using urea-PAGE (8% (v/v)) and concentrations were determined using NanoDrop (**Supporting figure S13B**).

Affinity-Based Profiling of the FMN Riboswitch *In Vitro*

RNA (20 µM) containing residues 23-166 of the *ribD* mRNA was incubated at 95 °C for 5 minutes and afterwards stored on ice for at least 5 minutes. Denatured RNA (2 µM) was incubated with the indicated probe (2-100 µM) in folding buffer (15 mM $MgCl_2$, 100 mM KCl and 50 mM HEPES (pH 8.0)) in the dark at 37 °C for 30 minutes. When required, photocrosslinking was performed with 365 nm UV light in a UV Stratalinker for 15 minutes. The RNA was isolated using an ethanol precipitation following a standard procedure described in Habibian *et al*. (2019)[2] and consequently dissolved in nuclease-free water (9 µL). For competition experiments, FMN (0-5 µM) or roseoflavin (0-1.0 mM) was incubated with RNA at 37 °C for 30 minutes, after which probe **1** (10 µM) was added and the samples were incubated at 37 °C for 30 minutes. To observe temperature-dependent probe binding, the incubation temperature during probe binding and UV irradiation was varied from 4-72 °C. To measure the influence of cation concentrations, the magnesium chloride (0-15 mM) was varied in the presence and absence of potassium chloride.

The RNA was incubated at 95 °C for 5 minutes, after which it was stored on ice for the click reaction. A ten-fold concentrated click mixture (1 µL) in water was added to each RNA sample to obtain final concentrations of 0.5 mM $CuSO_4$, 5.0 mM sodium ascorbate, 2.5 mM Tris-(3-hydroxypropyltriazolylmethyl)amine and 0.475 mM fluorescein azide or Cy5 azide. Cy5-azide was used for fluorescence intensity measurements, whereas fluorescein azide was used for downstream urea-PAGE analysis. The click reactions were incubated at ambient temperature for 60 minutes. All reagents were removed by performing an ethanol precipitation in a similar way as described earlier[2]. For fluorescence intensity measurements three additional precipitations were performed to ensure removal of unbound probe. The RNA was dissolved in nuclease-free water (10 µL), transferred onto a Greiner 384 well plate and fluorescence intensity measurements were performed using an excitation wavelength of 620 nm with bandwidth 20 and emission from 658-800 nm (Tecan Spark M10). For the FMN competition experiment using the Tecan Spark M10 microplate reader, the fluorescence measurements were performed with an excitation wavelength of 620 ± 20 nm and emission wavelength of 680 ± 20 nm. For downstream urea-PAGE analysis, the RNA pellets were dissolved in urea (8M), denatured for 3 minutes at 95 °C, and loaded on a denaturing 8% polyacrylamide gel. Products were separated in a gel in 1x TBE (pH 8.3), 500 V, ~1-1.5 hours. The gels were visualized by fluorescence imaging (GelDoc, BioRad) set on fluorescein. To visualize all RNA in the gels, the gels were afterwards stained using SYBR Gold and visualized by fluorescence imaging set on SYBR Gold (GelDoc, BioRad).

Relative intensities of the gel lanes were compared using Fiji[3]. Three biological replicates were run on the same gel per indicated condition. The observed FMN RNA bands in the gel were selected, after which the area under the peaks were calculated. To correct for differences between gels, a triplicate of standard labeling experiments was performed on every gel. The areas were corrected for background fluorescence intensity using empty gel lanes and normalized over the highest obtained

value. The normalized values were plotted using Origin version 8.5[4] using a logistic non-linear curve fitting with equation (**1**).

$$y = \mathrm{A2} + \frac{(\mathrm{A1}-\mathrm{A2})}{(1+\left(\frac{x}{x0}\right)^{p})} \qquad (\mathbf{1})$$

A1 stands for initial value. A2 stands for final value. x0 stands for center. p stands for power. x can be any value from the x-axis. y can be any value from the y-axis.

For the FMN competition experiment with fluorescence intensities, the values of triplicates were averaged, corrected using unlabeled RNA in nuclease-free water and normalized over the highest obtained value. The normalized values were plotted using Origin version 8.5[4] using a logistic non-linear curve fitting with equation (**1**).

An unpaired two-tailed Student's *t* test was performed to show significant labeling of FMN RNA after gel quantification in **Fig. 2B** (**Fig. 2C**). Error bars represent standard deviations based on three technical replicates.

Selective 2' Hydroxyl Acylation analyzed by Primer Extension

FMN RT RNA (20 µM) was heat denatured at 95 °C for 5 minutes and afterwards stored on ice for at least 5 minutes. RNA (2 µM) was incubated with FMN (100 µM), probe **1** (100 µM) or in absence of ligand in folding buffer (15 mM $MgCl_2$, 100 mM KCl and 50 mM HEPES (pH 8.0)) in the dark at 37 °C for 30 minutes. A 1M NAI solution was freshly prepared by mixing 2-methyl nicotinic acid (NA) (2M) with carbonyldiimidazole (CDI) (2M) in in dry DMSO. After ten minutes, the NAI solution was further diluted to the indicated final NAI concentration (0.5 mM) using dry DMSO. The prepared final NAI solution (0.5 mM) was added to each sample to obtain a 50 mM NAI concentration. The samples were incubated at 37°C in the dark for ten minutes. The reactions were quenched by performing an ethanol precipitation in a similar way as described earlier[2]. The RNA pellets were dissolved in 10 µL NF-water. Reverse transcription was performed using 4 pmol untreated or NAI treated RNA and 6 pmol fluorescein primer (5'- /56-FAM/GAA CCG GAC CGA AGC CCG -3'). To create sequencing lanes, reverse transcription was performed with a dNTP mix containing a 10:1 ratio for one of four selected ddNTP:dNTP (all NTPS were purchased at Jena Bioscience), whereas the other three dNTPs have a 1:1 ratio with the selected ddNTP. The reverse transcription reactions were incubated at 55 °C for 60 minutes. Superscript III (200 U) was added to the reactions after at least one minute preheating of the reactions at 55 °C.[1] Each reaction was treated with NaOH (1 M, 10 µL) and subsequently heated at 95 °C for 10 minutes. The reactions were neutralized using HCl (1 M, 10 µL). The cDNA was purified by performing an ethanol precipitation in a similar way as described earlier[2]. The pellets were dissolved in urea (8M) and loaded on a denaturing 8% polyacrylamide gel. Products were separated in a gel in 1x TBE (pH 8.3); 450 V for 120 minutes and 200 V for 20 hours. The gels were visualized by fluorescence imaging (Amersham Typhoon). The highlighted gel lanes and gel lanes used to correct for gel loading differences were quantified using Fiji[3]. The measured signal in the '-' samples were set on a value of one, after which the relative increased or decreased signals were calculated (**Supporting figure S3B**).

Reverse Transcription Termination Assay

FMN RT RNA (20 µM) was heat denatured at 95 °C for 5 minutes and afterwards stored on ice for at least 5 minutes. Denatured RNA (2 µM) was incubated with probe **1** (10 µM) or DMSO in folding buffer (15 mM $MgCl_2$, 100 mM KCl and 50 mM HEPES (pH 8.0)) in the dark at 37 °C or 51 °C for 30 minutes. For competition experiments, FMN (0-5 µM) was incubated with the RNA at 37 °C for 30 minutes, after which probe **1** (10 µM) was added and the samples were incubated at 37 °C for 30 minutes. When required, photocrosslinking was performed with 365 nm UV light in a UV Stratalinker for 15 minutes. The RNA was isolated using an ethanol precipitation in a similar way as described earlier[2] and dissolved

in nuclease-free water. Reverse transcription and downstream denaturing gel analysis was performed in the same way as described in the 'Selective 2' Hydroxyl Acylation analyzed by Primer Extension' section. The lanes that gave a different signal in samples '+UV' compared to '-UV' and the gel lanes that were used to correct for gel loading differences were quantified using Fiji[3]. The measured signals in the '-UV' samples were set on a value of one, after which the relative increased or decreased signals were calculated (**Supporting figure S9B** and **C**).

Molecular Docking of Probe **1** in the FMN Riboswitch

Using the experimental data published by several FMN related studies[5–8], molecular docking of probe **1** in the FMN riboswitch was carried out using the Maestro 12.9 software package (Schrödinger, LLC)[9]. A computational model of probe **1** bound to the FMN riboswitch was created using the 'crystal structure of the FMN riboswitch bound to FMN' (PDB ID: 3F2Q), which was published by Serganov *et al.* (2009)[5] as starting structure. The structure of the FMN ligand was modified to the structure of probe **1** and the obtained structure was refined using the OPLS4 force field (Schrödinger, LLC)[9,10]. Only conserved nucleotides found in both the FMN riboswitch of *Bacillus Subtilis* wildtype and *Fusobacterium Nucleatum* were discussed and highlighted in the shown structures[7].

Bacterial Growing Conditions

Bacillus Subtilis subspecies subtilis strain 168 were grown on sterile Luria Broth (LB)-low salt (5 g/L yeast extract, 10 g/L tryptone and 5 g/L sodium chloride) agar plates supplemented with magnesium sulfate (5 mM) and glucose (20 mM) at 37 °C. Colonies were picked and grown in liquid LB-low salt medium, containing magnesium sulfate (5 mM) and glucose (20 mM) at 37 °C. Colonies were stored on agar plates at 4 °C for one week maximum. Glycerol stocks (20% (v/v)) of confluent bacteria were created and stored at -80 °C.

Escherichia Coli CS1562 (*tolC6:tn10*) were grown on sterile LB (5 g/L yeast, 10 g/L tryptone and 10 g/L sodium chloride) agar plates supplemented with tetracycline (12 µg/mL) at 37 °C. Colonies were picked and grown in liquid LB medium supplemented with tetracycline (12 µg/mL) at 37 °C. Colonies were stored on agar plates at 4 °C for one week maximum. Glycerol stocks (20% (v/v)) of confluent bacteria were created and stored at -80 °C.

Escherichia Coli CS1562 bacteria containing pTXTL-sroGp2eGFP were grown similar as *Escherichia Coli* CS1562, but supplemented with tetracycline (12 µg/mL) and ampicillin (100 µg/mL).

Affinity-Based Profiling of the FMN Riboswitch from Bacterial Extracts

Bacillus Subtilis bacteria were grown to OD_{600} of 0.8. Total RNA was extracted from the bacteria using TRIzol LS reagent following the method described in Toni *et al.* (2018)[11]. The concentration and purity of the RNA was measured using NanoDrop. RNA extracts were considered sufficiently pure when both A_{260}/A_{230} and A_{260}/A_{280} ratios were above 2.0. Approximately 30 µg of total RNA extract was incubated at 95 °C for 5 minutes and afterwards stored on ice for at least 5 minutes. Denatured RNA was incubated with probe **1** (100 µM), DMSO or **4** (100 µM) in folding buffer (15 mM $MgCl_2$, 100 mM KCl and 50 mM HEPES (pH 8.0)) in a total volume of 30 µL in the dark at 37 °C for 30 minutes. For competition experiments, FMN (100 µM) was incubated with the RNA at 37 °C for 30 minutes, after which probe **1** (100 µM) was added and the samples were incubated at 37 °C for 30 minutes. For temperature-dependent binding experiments, the indicated incubation temperatures were used, whereas other conditions were kept the same. When required photocrosslinking was performed with 365 nm UV light in a UV Stratalinker for 15 minutes. RNA was isolated using an ethanol precipitation as described earlier[2] and subsequently dissolved in nuclease-free water (9 µL). The RNA was incubated at 95 °C for 5 minutes, after which it was stored on ice for the click reaction. A ten-fold concentrated click mixture (1 µL) in water was added to each RNA sample to obtain final concentrations of 0.5 mM

$CuSO_4$, 5.0 mM sodium ascorbate, 2.5 mM Tris-(3-hydroxypropyltriazolylmethyl)amine and 0.475 mM disulfide biotin azide (Click Chemistry Tools). The click reactions were incubated at 37 °C for 90 minutes. All reagents were removed by performing an ethanol precipitation in a similar way as described earlier[2]. RNA pellets were dissolved in 100 µL binding buffer (0.5M NaCl, 20 mM Tris-HCl (pH 7.5), 1 mM EDTA) and Streptavidin magnetic beads (25 µL, NEB) were added for pulldown of biotinylated RNA. After incubating at 37 °C for 90 minutes, the supernatant was removed and the beads were washed three times with washing buffer (0.5M NaCl, 20 mM Tris-HCl (pH 7.5), 1 mM EDTA, 0.2% (v/v) Tween-20) and low-salt buffer (0.15M NaCl, 20 mM Tris-HCl (pH 7.5), 1 mM EDTA). RNA was eluted from the beads using DTT (20 µL, 50 mM). Eluted RNA (2 µL) was used for RT-qPCR using prescribed protocols for Superscript III (Thermo Scientific) and 4x CAPITAL qPCR Green Master Mix (Biotechrabbit). The RT method was adapted for the DTT concentration used during RNA elution and the samples were treated with RNase H (5U, NEB) after reverse transcription. The cDNA material was three-fold concentrated by performing an ethanol precipitation in a similar way as described earlier[2] and dissolving the pellets in nuclease-free water, and 10% (v/v) RT material was used for qPCR. Biological triplicates were measured, each consisting of two technical replicates. The levels of *gyrase B (gyrB)* were monitored as housekeeping gene for relative quantification. The qPCR samples were analyzed using agarose gel electrophoresis (2%) to validate the amplification of the desired amplicons. The used annealing temperature during qPCR was 58 °C and 45 amplification cycles were run. The used primers are provided in **Supporting table S3**. The relative fold enrichment of *ribD* was calculated using equation (**2**):

$$relative\ fold\ enrichment = 2^{-(\Delta\text{Ct before pulldown} - \Delta\text{Ct after pulldown})} \quad \textbf{(2)}$$

where $\Delta Ct^{\text{before pulldown}}$ is the difference between the Ct values for the RNA of interest and a housekeeping gene (*gyrB*) in total RNA from cells and $\Delta Ct^{\text{after pulldown}}$ is the difference between the Ct values for the RNA of interest and the same housekeeping gene in RNA after pulldown. The values were normalized over the background signal observed in the DMSO control samples. The error bars represent standard errors based on three biological replicates, each consisting of two technical replicates. Statistical significance was calculated using an unpaired two-tailed Student's *t* test.

**Indicated DNA strand**	**Sequence**
Reverse transcription primer *ribD*	5' CCA TTT GTT TCC CTC CCC TCT TTT GTT ATT TAC C 3'
Forward primer qPCR *ribD*	5' TGA GCC GCT ATG CAA AAT GTT T 3'
Reverse primer qPCR *ribD*	5' CAT TTG TTT CCC TCC CCT CTT TTG 3'
Reverse transcription primer *gyrB*	5' TAC CGC GGC CAT TAT CTA CAA CC 3'
Forward primer qPCR *gyrB*	5' AAA CAG CAA AGG CCT TCA CCA CT 3'
Reverse primer qPCR *gyrB*	5' TAC CGC GGC CAT TAT CTA CAA CC 3'

Supporting table S3. DNA sequences of the used primers during RT-qPCR for selected amplicons of the *ribD* and *gyrB* genes[12].

Construction of pTXTL-sroGp2eGFP

The design of the plasmid pTXTL-sroGp2eGFP was based on a similar plasmid constructed by Pedrolli *et al.* (2015)[13], in which the *E. Coli ribB* FMN riboswitch was placed upstream a luciferase expressing gene (**Supporting figure S11A**). All fragments were obtained in-house, except for fragment psroGp2 (synthetic gBlock, Integrated DNA Technologies) (**Supporting table S4**).[13] The vector fragment (100 ng) was combined with the other indicated fragments in a 1:3 molar ratio in 15 µL master mixes including T4 ligase buffer (1x) and 1 µL of Esp3I and T4 ligase. The reactions are incubated using the following program: 37 °C for 2 minutes, 20 °C for 3 minutes (25 cycles); 37 °C for 5 minutes (1 cycle); 55 °C for 10 minutes (1 cycle); 80 °C for 10 minutes (1 cycle).

Fragment	Sequence
**Vector**	CTCCTAGGCTCCGCCCCCCTGACGAGCATCACAAAAATCGACGCTCAAGTCAGAGGTGGC GAAACCCGACAGGACTATAAAGATACCAGGCGTTTCCCCCTGGAAGCTCCCTCGTGCGCT CTCCTGTTCCGACCCTGCCGCTTACCGGATACCTGTCCGCCTTTCTCCCTTCGGGAAGCGTG GCGCTTTCTCATAGCTCACGCTGTAGGTATCTCAGTTCGGTGTAGGTCGTTCGCTCCAAGC TGGGCTGTGTGCACGAACCCCCCGTTCAGCCCGACCGCTGCGCCTTATCCGGTAACTATCG TCTTGAGTCCAACCCGGTAAGACACGACTTATCGCCACTGGCAGCAGCCACTGGTAACAGG ATTAGCAGAGCGAGGTATGTAGGCGGTGCTACAGAGTTCTTGAAGTGGTGGCCTAACTACG GCTACACTAGAAGAACAGTATTTGGTATCTGCGCTCTGCTGAAGCCAGTTACCTTCGGAAAA AGAGTTGGTAGCTCTTGATCCGGCAAACAAACCACCGCTGGTAGCGGTGGTTTTTTTGTTTG CAAGCAGCAGATTACGCGCAGAAAAAAAGGATCTCAAGAAGATCCTTTGATCTTTTCTACGG GGTCTGACGCTCAGTGGAACGAAAACTCACGTTAAGGGATTTTGGTCATGAGATTATCAAAA AGGATCTTCACCTAGATCCTTTTAAATTAAAAATGAAGTTTTAAATCAATCTAAAGTATATATG AGTAAACTTGGTCTGACAGTTACCAATGCTTAATCAGTGAGGCACCTATCTCAGCGATCTGTC TATTTCGTTCATCCATAGTTGCCTGACTCCCCGTCGTGTAGATAACTACGATACGGGAGGGCT TACCATCTGGCCCCAGTGCTGCAATGATACCGCGAGACCCACGCTCACCGGCTCCAGATTTAT CAGCAATAAACCAGCCAGCCGGAAGGGCCGAGCGCAGAAGTGGTCCTGCAACTTTATCCGCC TCCATCCAGTCTATTAATTGTTGCCGGGAAGCTAGAGTAAGTAGTTCGCCAGTTAATAGTTTGC GCAACGTTGTTGCCATTGCTACAGGCATCGTGGTGTCACGCTCGTCGTTTGGTATGGCTTCATT CAGCTCCGGTTCCCAACGATCAAGGCGAGTTACATGATCCCCCATGTTGTGCAAAAAAGCGGT TAGCTCCTTCGGTCCTCCGATCGTTGTCAGAAGTAAGTTGGCCGCAGTGTTATCACTCATGGTT ATGGCAGCACTGCATAATTCTCTTACTGTCATGCCATCCGTAAGATGCTTTTCTGTGACTGGTG AGTACTCAACCAAGTCATTCTGAGAATAGTGTATGCGGCGACCGAGTTGCTCTTGCCCGGCGT CAACACGGGATAATACCGCGCCACATAGCAGAACTTTAAAAGTGCTCATCATTGGAAAACGTT CTTCGGGGCGAAAACTCTCAAGGATCTTACCGCTGTTGAGATCCAGTTCGATGTAACCCACTC GTGCACCCAACTGATCTTCAGCATCTTTTACTTTCACCAGCGTTTCTGGGTGAGCAAAAACAGG AAGGCAAAATGCCGCAAAAAAGGGAATAAGGGCGACACGGAAATGTTGAATACTCATACTCT TCCTTTTTCAATATTATTGAAGCATTTATCAGGGTTATTGTCTCATGAGCGGATACATATTTGAA TGTATTTAGAAAAATAAACAAATAGGGGTTCCGCGCACATTTCCGGAGACGGGAGAA
**5’**	TTCTCCCGTCTCCTTCCCCGAAAAGTGCCACCTGACGTCTAAGAAACCATTATTATCATGACA TTAACCTATAAAAATAGGCGTATCACGAGGCCCTTTCGTCTTCAAGAATTCTGGCGAATCCT CTGACCAGCCAGAAAACGACCTTTCTGTGGTGAAACCGGATGCTGCAATTCAGAGCGCCAG CAAGTGGGGGACAGCAGAAGACCTGACCGCCGCAGAGTGGATGTTTGACATGGTGAAGAC TATCGCACCATCAGCCAGAAAACCGAATTTTGCTGGGTGGGCTAACGATATCCGCCTGATGC GTGAACGTGACGGACGTAACCACCGCGACATGTGTGTGCTGTTCCGCTGGGCATGCCAGGA CAACTTCTGGTCCGGTAACGTGCTGAGCGGAGACGGGAGAA
**sroGp2**[13]	TTCTCCCGTCTCCGAGCGGCGTTGCTGCCGCTAATCATTAGCGTtatagtGAATCCgCTTAaagctt GCttattctcaGGGCGGggcgaaattCCCCACCggcggtaaatCAACTCAGttgaaagcccgcgAGCGCTTT GGGTGCGaactcaaaggacaGCAGATCCggtgtaattCCGGGGCCGACggttagagtccGGATGGGAG AGAgtaacgatTCTGTCGGGCATggacccgctcacGTTATTTTGGCTATATgccgccactcctaagactgcc ctgattCTGGTAACCATAATTTTagtgaggTTTTTTaccATGAATCAGACGCTACTTtGGATCCaaGC AGAGTTGGGAGACGCCTCA
**eGFP**	TTCTCCCGTCTCCAATGGAGCTTTTCACTGGCGTTGTTCCCATCCTGGTCGAGCTGGACGGCGA CGTAAACGGCCACAAGTTCAGCGTGTCCGGCGAGGGCGAGGGCGATGCCACCTACGGCAAGC TGACCCTGAAGTTCATCTGCACCACCGGCAAGCTGCCCGTGCCCTGGCCCACCCTCGTGACCAC CCTGACCTACGGCGTGCAGTGCTTCAGCCGCTACCCCGACCACATGAAGCAGCACGACTTCTTC AAGTCCGCCATGCCCGAAGGCTACGTCCAGGAGCGCACCATCTTCTTCAAGGACGACGGCAAC

	TACAAGACCCGCGCCGAGGTGAAGTTCGAGGGCGACACCCTGGTGAACCGCATCGAGCTGAAGGGCATCGACTTCAAGGAGGACGGCAACATCCTGGGGCACAAGCTGGAGTACAACTACAACAGCCACAACGTCTATATCATGGCCGACAAGCAGAAGAACGGCATCAAGGTGAACTTCAAGATCCGCCACAACATCGAGGACGGCAGCGTGCAGCTCGCCGACCACTACCAGCAGAACACCCCCATCGGCGACGGCCCCGTGCTGCTGCCCGACAACCACTACCTGAGCACCCAGTCCGCCCTGAGCAAAGACCCCAACGAGAAGCGCGATCACATGGTCCTGCTGGAGTTCGTGACCGCCGCCGGGATCTAACTCGAGGGAGACGGGAGAA
**T500-3’**	TTCTCCCGTCTCCCGAGCAAAGCCCGCCGAAAGGCGGGCTTTTCTGTGTCGACCGATGCCCTTGAGAGCCTTCAACCCAGTCAGCTCCTTCCGGTGGGCGCGGGGCATGACTATCGTCGCCGCACTTATGACTGTCTTCTTTATCATGCAACTCGTAGGACAGGTGCCGGCAGCGCTCTTCCGCTTCCTCGCTCACTGACTCGCTGCGCTCGGTCGTTCGGCTGCGGCGAGCGGTATCAGCTCACTCAAAGGCGGTAATACGGTTATCCACAGAATCAGGGGATAACGCAGGAAAGAACATGTGAGCAAAAGGCCAGCAAAAGGCCAGGAACCGTAAAAAGGCCGCGTTGCTGGCGTTTTTCCATAGGGGAGACGGGAGAA

Supporting table S4. Sequences of all the used fragments required to construct plasmid pTXTL-sroGp2eGFP.[13]

The ligated DNA (1 µL) was diluted three-fold, after which 2 µL ligated DNA was mixed with 200 µL competent *Escherichia Coli* TOP10F cells. The samples were incubated on ice for 30 minutes, after which a heat shock was performed at 42 °C for exactly 45 seconds. The bacteria were incubated on ice for 2 minutes, after which 800 µL LB liquid was added. The cells were recovered at 37 °C for 20 minutes before 100 µL cells were spread on LB-agar plates containing 50 µg/mL ampicillin. The plates were incubated at 37 °C overnight. Colonies were picked and grown in liquid LB medium containing 50 µg/mL ampicillin until they reached OD_{600} of 0.9. The DNA was extracted using QIAprep Spin Miniprep Kit (QIAGEN). The size of the plasmid was analyzed using agarose gel electrophoresis (2%) and the sequence of the obtained plasmid was verified using Sanger sequencing (Baseclear) (**Supporting figure S11A-C**).

Transformation of pTXTL-sroGp2eGFP Plasmid in *E. Coli* CS1562

Escherichia Coli CS1562 cells were rendered competent using calcium chloride following a procedure described by Chang *et al.* (2017)[14]. The competent *Escherichia Coli* CS1562 cells (200 µL) were combined with the isolated plasmid DNA solution (400 ng). The tubes were incubated on ice for 30 minutes, after which a heat shock was performed at 42 °C for exactly 45 seconds. The bacteria were incubated on ice for 2 minutes, after which 800 µL sterile LB liquid was added. The cells were recovered at 37 °C for 20 minutes before 100 µL cells were spread on LB-agar plates supplemented with 12 µg/mL tetracycline and 100 µg/mL ampicillin. The plates were incubated at 37 °C overnight. Colonies were picked and grown in liquid LB medium supplemented with 12 µg/mL tetracycline and 100 µg/mL ampicillin until they reached OD_{600} of 0.9. The DNA was extracted using QIAprep Spin Miniprep Kit (QIAGEN). Presence of the plasmid checked using agarose gel electrophoresis (2%) and ethidium bromide (50 µg). The gels were scanned using a GelDoc (BioRad) on ethidium bromide settings. Additionally, PCR was performed to check the sequence. For PCR, 200 µL reaction mixes were prepared, each containing: Phusion HF buffer (1x, Thermo Fisher Scientific); dNTPs (0.2 mM, Jena Bioscience); forward primer (1.0 µM) (ATAGGGGTTCCGCGCAC) and reverse primer (1.0 µM) (GGGGCGGAGCCTATGGA); DNA template (10 ng); Phusion DNA polymerase (4 units, prepared in house). Conditions for the PCR amplification were as follows: 98 °C for 30 s (1 cycle); 98 °C for 10 s, 60 °C for 20 s, 72 °C for 90 s (30 cycles); 72 °C for 600 s (1 cycle) in a TProfessional thermal cycler (Biometra). The PCR efficiency was checked using agarose gel electrophoresis (2%) and ethidium bromide (50 µg). The gels were scanned using a GelDoc (BioRad) on ethidium bromide settings. The

sequence of the obtained plasmid was verified using Sanger sequencing (Baseclear) (**Supporting figure S11A-C**)

Bacterial Growth Curves to Examine Cell Ent0rance of Flavin-Containing Compounds

These experiments were based on Howe *et al*. (2015)[15] using the method described earlier by Velema *et al.* (2013)[16]. Briefly, overnight cultures of *Escherichia Coli* CS1562 containing pTXTL-sroGp2eGFP were diluted to an optical density at 600 nm (OD_{600}) of 0.1 in the presence or absence of indicated concentration of riboflavin, and 100 µL of this cell suspension was added to 100 µL medium containing antibiotics and riboflavin if indicated at given concentrations. Cells were grown in transparent 96-well plates at 37 ˚C and the cell density (OD_{630}) was measured every 10 minutes for 16 h with a 10 s linear shaking step before each read in a microplate reader (Synergy SH1M, BioTek). Graphs were background-corrected by subtracting the OD_{630} at time 0. Error bars represent standard deviations based on three technical replicates.

Affinity-Based Profiling of the FMN Riboswitch in Live Bacteria

E. Coli CS1562 bacteria containing pTXTL-sroGp2eGFP were grown to OD_{600} of 0.8. The bacteria were transferred to fresh LB medium containing tetracycline (12 µg/mL) and ampicillin (100 µg/mL) and treated with 25 µM **1** after pretreating the bacteria with an indicated concentration of roseoflavin (0-500 µM in a maximum of 1% (v/v) DMSO) in the dark for 30 minutes. The cells were irradiated with 365 nm UV light in a UV Stratalinker for 15 minutes using transparent 6-well plates, each well containing a maximum of 2 mL bacterial solution. Total RNA was extracted from the bacteria using TRIzol LS reagent following the method described in Toni *et al*. (2018)[11]. The concentration and purity of the RNA was measured using NanoDrop. RNA extracts were considered sufficiently pure when both A_{260}/A_{230} and A_{260}/A_{280} ratios were above 2.0. Approximately 30 µg of total RNA extract was diluted to a total volume of 27 µL nuclease-free water, incubated at 95 °C for 5 minutes and afterwards stored on ice for at least 5 minutes. A ten-fold concentrated click mixture (1 µL) in nuclease-free water was added to each RNA sample to obtain final concentrations of 0.5 mM $CuSO_4$, 5.0 mM sodium ascorbate, 2.5 mM Tris-(3-hydroxypropyltriazolylmethyl)amine and 0.2 mM disulfide biotin azide (Click Chemistry Tools). The click reactions were incubated at 37 °C for 90 minutes. All reagents were removed by performing an ethanol precipitation in a similar way as described earlier[2]. The pulldown and reverse transcription procedures are similar as described in the section 'Affinity-based Profiling of the FMN riboswitch from Bacterial Extracts'. After RNase H treatment, qPCR was performed using the 4X CAPITAL^{TM} qPCR Probe Master Mix using 2 µL of RT material and following prescribed instructions using the primers provided in **Supporting table S5**. The levels of *cysG* were monitored as housekeeping gene for relative quantification. The qPCR samples were analyzed using agarose gel electrophoresis (2%) to validate the amplification of the desired amplicons. The used annealing temperature during qPCR was 56 °C and 45 amplification cycles were run. The relative fold enrichment of *ribB* FMN riboswitch was measured using eq (**2**), with *cysG* as housekeeping gene. The values were normalized over background signal observed in the DMSO control samples. For the roseoflavin competition experiment, the values were corrected for the fold enrichment observed in the DMSO control samples and normalized over the highest signal. The normalized values were plotted using Origin version 8.5.0[4] using a logistic non-linear curve fitting with equation (**1**). The error bars represent standard deviations based on three biological replicates, each consisting of two technical replicates. Statistical significance was calculated using an unpaired two-tailed Student's *t* test.

Indicated DNA strand	Sequence
Reverse transcription primer *ribB*	5’ AAT TAT GGT TAC CAG AAT CAG GGC AGT CT 3’
Forward primer qPCR *ribB*	5’ GTA ACG ATT CTG TCG GGC AT 3’
Reverse primer qPCR *ribB*	5’ TAC CAG AAT CAG GGC AGT CT 3’
ribB Taqman probe	5’ /56-FAM/ACC CGC TCA CGT TAT TTT GGC TAT AT/3BHQ_1/ 3’
Forward primer qPCR *cysG*	5’ TTG TCG GCG GTG GTG ATG TC 3’
Reverse primer qPCR *cysG*	5’ ATG CGG TGA ACT GTG GAA TAA ACG 3’
Taqman probe *cysG*	5’ /56-FAM/ACG CAA AGC AAG GTT GCT GTT AGA CGC A/3BHQ_1/ 3’

Supporting table S5. DNA sequences of the used primers during RT-qPCR for selected amplicons of the *ribB* and *cysG* genes[12].

SYNTHESIS, PURIFICATION AND ANALYSIS OF COMPOUNDS

All reagents described below were purchased from commercial suppliers and used as received. Temperatures are given in degrees Celsius (°C). Unless otherwise stated, reactions were carried out at room temperature (18-25 °C). Progress of reactions and RF values were determined using thin-layer-chromatography (TLC) silica gel-coated plates (Merck 60 F254) using the described eluent. Column chromatography was performed with VWR chemicals silica gel (0.040-0.063 mm, pore size 60 Å). The separation was visualized with UV light and/or by potassium permanganate or ninhydrin staining. Low-resolution electron-spray ionisation mass spectra (MS ESI) were recorded on a Thermo Finnigan LCQ Advantage Max Ion Trap mass spectrometer. NMR spectra were recorded in the indicated solvent at 25 °C on a Bruker 500 MHz Avance III spectrometer equipped with a Prodigy BB cryoprobe, a Bruker 400 MHz Avance III HD nanobay spectrometer equipped with a BBFO probe, or an Agilent 400 MHz INOVA spectrometer equipped with a dual-channel inverse probe. Chemical shifts are displayed in parts per million (ppm) with respect to TMS (δ = 0.00 ppm), $CDCl_3$ (δ = 7.26 ppm) or other indicated deuterated solvent as internal reference for ^1H NMR; and $CDCl_3$ (δ = 77.16 ppm) or other indicated deuterated solvent as internal reference for ^{13}C NMR. Coupling constants are reported in hertz (Hz) as *J* values. The multiplicity is described by the number of coupling constants, the number of peaks, and the pattern in the signal (s=singlet, d=doublet, t=triplet, q=quartet, dd= doublet of doublets, dt=doublet of triplets, ddd=doublet of doublet of doublets, m=multiplet, br=broad). The peak assignments in ^1H and ^{13}C spectra are based on 2D COSY, HSQC and HMBC spectra. Each stated ^1H NMR, ^{13}C NMR and Mass spectra result is depicted in the section ‘SPECTRA’.

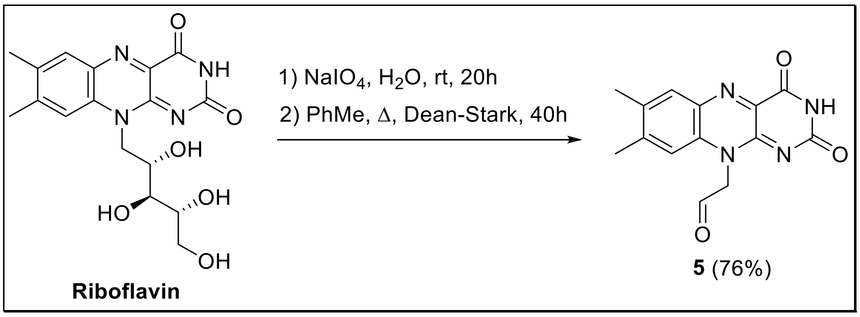

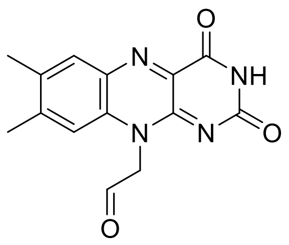

2-(7,8-dimethyl-2,4-dioxo-3,4-dihydrobenzo[g]pteridin-10(2H)-yl)acetalde-hyde (**5**)[17]

Riboflavin (684.6 mg, 1.82 mmol) and sodium *m*-Periodate (849.2 mg, 3.97 mmol) were suspended in water (25 mL) and stirred at room temperature overnight. Subsequently, the suspension was filtered and the residue was washed with cold water, methanol: diethyl ether (1:1) and diethyl ether to afford the crude diol (ca. 600 mg) as a yellow/orange solid. ^{1}H NMR (400 MHz, dimethyl sulfoxide-d$_6$) δ 11.33 (s, 1H), 7.89 – 7.87 (m, 1H), 7.86 (s, 1H), 6.64 (d, *J* = 6.9 Hz, 1H), 4.99 (q, *J* = 5.7 Hz, 1H), 4.70 – 4.55 (m, 2H), 2.49 (s, 3H), 2.40 (s, 3H). After the remaining solvent was evaporated, the crude was dissolved in toluene and refluxed under Dean-Stark conditions for 5 hrs. Subsequently, the suspension was filtered off, washed with diethyl ether and the residue was obtained to afford **5** (417.4 mg, 76% yield) as an orange solid. ^{1}H NMR (400 MHz, dimethyl sulfoxide-d$_6$) δ 11.37 (s, 1H), 9.74 (s, 1H), 7.94 (d, *J* = 1.1 Hz, 1H), 7.70 (s, 1H), 5.64 (s, 2H), 2.46 (s, 3H), 2.42 – 2.38 (m, 3H). ^{13}C NMR (101 MHz, dimethyl sulfoxide-d$_6$) δ 195.71, 171.65, 160.17, 155.73, 155.36, 155.28, 150.59, 147.37, 136.54, 134.00, 131.44, 116.76, 54.22, 21.00, 19.22.

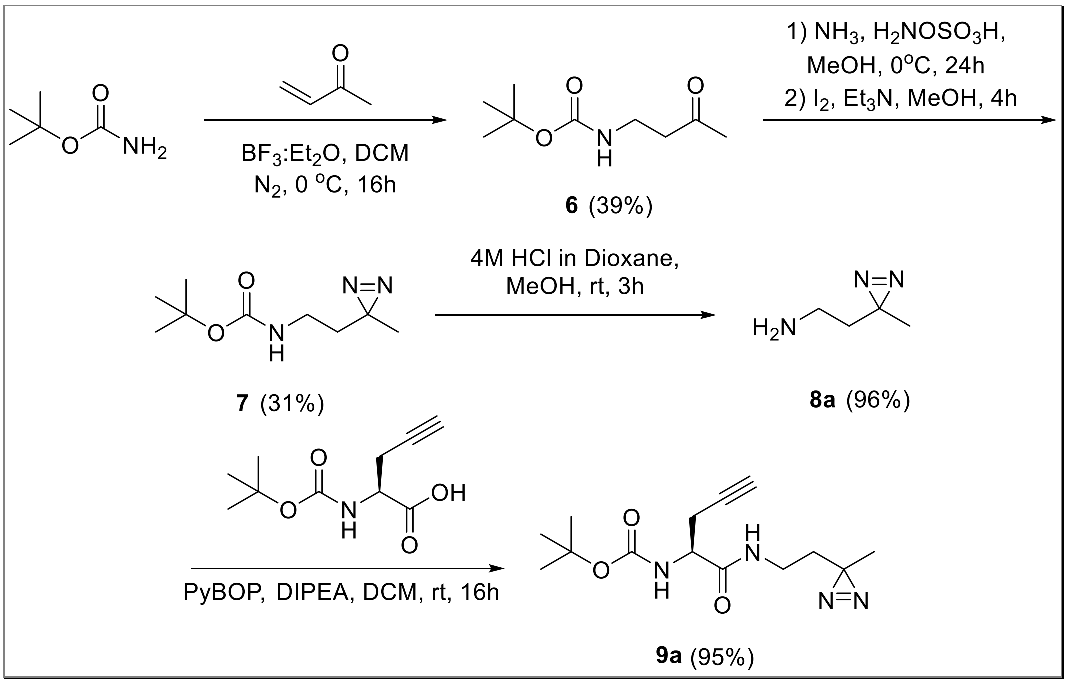

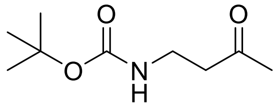

tert-Butyl (3-oxobutyl)carbamate (**6**)

Methyl vinyl ketone (1.7 ml, 24.60 mmol) and boron trifluoride etherate (0.25 mL, 2.026 mmol) were dissolved in dry dichloromethane (20 mL) and cooled to 0 °C under inert atmosphere. Subsequently, a solution of *tert*-butyl carbamate (2.0 g, 17.07 mmol) in dry dichloromethane (20 mL) was added dropwise to the mixture and was allowed to reach room temperature while stirring overnight. Afterwards, the reaction was quenched with water, partitioned with dichloromethane (three times), washed with water (two times) and brine (two times), dried over magnesium sulfate, filtered, concentrated *in vacuo,* and dissolved in a mixture of n-heptane and toluene (4:1), filtered and the filtrate was concentrated *in vacuo* to yield an orange solid. Further purification was achieved by column chromatography (n-heptane:ethyl acetate 3:2) to afford **6** (1.235 g, 39% yield) as a yellow oil. Rf = 0.40 (n-heptane:ethyl acetate 1:1). ^{1}H NMR (400 MHz, chloroform-d) δ 4.99 (s, 1H), 3.35 (q, *J* = 6.0 Hz, 2H), 2.67 (t, *J* = 5.8 Hz, 2H), 2.16 (s, 3H), 1.43 (s, 9H). ^{13}C NMR (101 MHz, chloroform-d) δ 208.23, 155.90, 79.29, 43.53, 35.16, 30.18, 28.40.

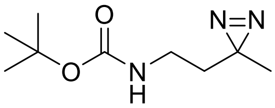

tert-Butyl (2-(3-methyl-3H-diazirin-3-yl)ethyl)carbamate (7)

Compound **6** (1.24 g, 6.62 mmol) was cooled to 0 °C under N_2. Subsequently, 7M NH_3 in methanol (10.5 mL) was added dropwise to stir for 3 h. Subsequently, a solution of hydroxylamine-*O*-sulfonic acid (830.4 mg, 7.34 mmol) in dry methanol (8 mL) was added dropwise to the mixture at 0°C and the resulting mixture was allowed to reach room temperature while stirring overnight. Afterwards, residual solvent was evaporated *in vacuo*, and the residue was resuspended in dry methanol, filtered, washed with dry methanol and the filtrate was concentrated under reduced pressure to afford an orange oil. The crude oil was re-dissolved in dry methanol (10 ml) and cooled to 0°C. Subsequently, triethylamine (4.0 mL) was added, followed by iodine (1.7 g, 6.7 mmol) in small portions over 4 h until a dark brown color persisted in the solution for more than 10 minutes. Afterwards, the reaction was quenched with brine, partitioned with ethyl acetate (three times), washed with 1M hydrogen chloride, and saturated aqueous sodium thiosulfate, dried over magnesium sulfate and concentrated *in vacuo* to yield an orange oil. Further purification was achieved with column chromatography (n-heptane:ethyl acetate = 2:1) to afford **7** (407.2 mg, 31% yield) as a pale-yellow oil. Rf = 0.67 (n-heptane:ethyl acetate 2:1). ^{1}H NMR (400 MHz, chloroform-d) δ 4.56 (s, 1H), 3.05 (q, *J* = 6.7 Hz, 2H), 1.56 (t, *J* = 6.9 Hz, 2H), 1.45 (s, 9H), 1.05 (s, 3H). ^{13}C NMR (101 MHz, chloroform-d) δ 155.74, 79.51, 35.71, 34.66, 28.40, 19.87.

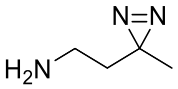

2-(3-methyl-3H-diazirin-3-yl)ethan-1-amine (8a)

Compound **7** (565.5 mg, 2.84 mmol) was dissolved in methanol (2 mL). Subsequently, 4M hydrogen chloride in dioxane (2 mL) was added to the solution and stirred at room temperature for 3 h. Afterwards, the reaction mixture was concentrated *in vacuo* to afford **8a** (369.7 mg, 96% yield) as a pale green solid. ^{1}H NMR (400 MHz, chloroform-d) δ 8.20 (s, 3H), 2.99 (h, *J* = 6.0 Hz, 2H), 1.90 (dd, *J* = 8.6, 7.0 Hz, 2H), 1.13 (s, 3H).

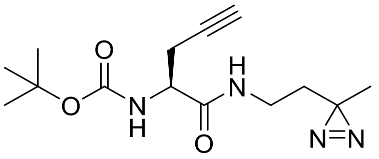

tert-Butyl (*S*)-(1-((2-(3-methyl-3H-diazirin-3-yl)ethyl)amino)-1-oxopent-4-yn-2-yl)carbamate (9a)

2-((tert-butoxycarbonyl)amino)pent-4-ynoic acid (221 mg, 1.63 mmol), compound **8a** (190 mg, 1.4 mmol), benzotriazole-1-yloxytripyrrolidinophosphonium hexafluorophosphate (730 mg, 1.4 mmol) and *N,N*-diisopropylethylamine (0.9 mL, 5.2 mmol) were dissolved in dichloromethane (5 mL) and the reaction mixture was stirred overnight. The mixture was concentrated *in vacuo*, quenched with brine, partitioned with ethyl acetate (three times), washed with 1M hydrogen chloride, and saturated aqueous sodium thiosulfate and concentrated *in vacuo* to yield a brown oil. Further purification was achieved with column chromatography (n-heptane:ethyl acetate 3:1) to afford **9a** (456.9 mg, 95% yield) as a pale-yellow foam. Rf-value = 0.45 (n-heptane:ethyl acetate 3:1). ^{1}H NMR (400 MHz, chloroform-d) δ 6.42 (s, 1H), 5.28 (s, 1H), 4.26 (d, *J* = 7.1 Hz, 1H), 3.28 – 3.14 (m, 2H), 2.82 (ddd, *J* = 17.0, 5.5, 2.6 Hz, 1H), 2.61 (ddd, *J* = 16.9, 6.7, 2.6 Hz, 1H), 2.09 (t, *J* = 2.6 Hz, 1H), 1.61 (t, *J* = 6.9 Hz, 2H), 1.47 (s, 9H), 1.05 (s, 3H). ^{13}C NMR (101 MHz, chloroform-d) δ 170.19, 80.67, 79.43, 71.70, 60.40, 34.79, 34.08, 28.29, 24.33, 22.30, 19.79.

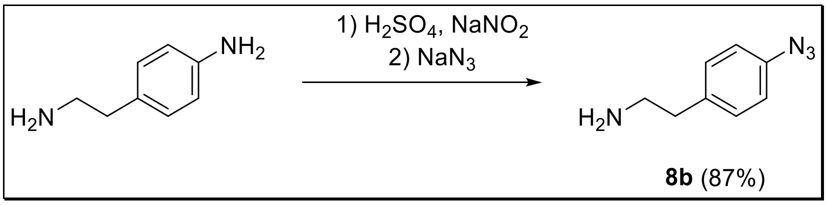

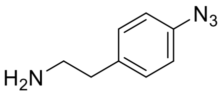

2-(4-azidephenyl)ethan-1-amine (**8b**)

4-(2-aminoethyl)aniline (522 mg, 3.84 mmol) was suspended in water and cooled on ice to 0 °C. Concentrated sulfuric acid (4 mL) and $NaNO_2$ (278 mg, 4.03 mmol) were added and the reaction was stirred at 0 °C for 20 minutes. A solution of NaN_3 (262 mg, 4.03 mmol) dissolved in water was added dropwise at 0 °C. After stirring for 10 min at 0 °C, 25% aqueous sodium hydroxide solution was added to adjust the mixture to basic conditions. EtOAc was added, the layers were separated and the aqueous layer extracted with ethyl acetate (5 × 120 mL). The combined organic layers were washed with water and brine, dried over magnesium sulfate, filtered and concentrated under reduced pressure to obtain **8b** (542 mg, 87%) as a red oil. ^{1}H NMR (400 MHz, chloroform-d) δ 7.18 (d, *J* = 8.3 Hz, 1H), 6.96 (d, *J* = 8.4 Hz, 1H), 2.94 (t, *J* = 6.9 Hz, 1H), 2.72 (t, *J* = 6.9 Hz, 1H).

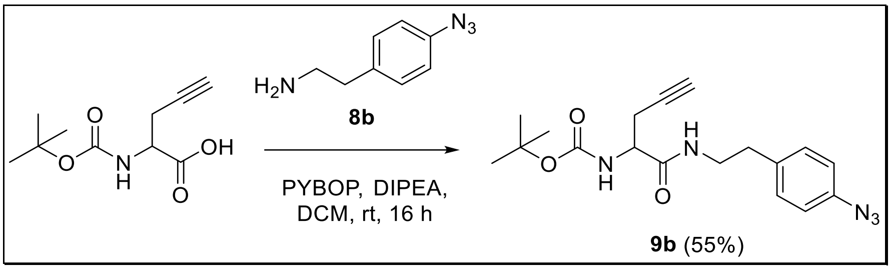

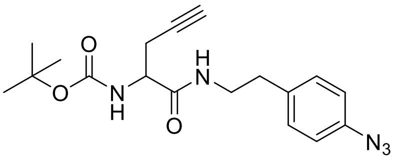

(*S*)-(1-((4-azidophenethyl)amino)-1-oxopent-4-yn-2-yl)-carbamate (**9b**)

(*S*)-2-((*tert*-butoxycarbonyl)amino)pent-4-ynoic acid (1.5 g, 7.0 mmol), N,N-diisopropylethylamine (3.65 mL, 21 mmol), benzotriazol-1-yl-oxytripyrrolidinophosphonium hexafluoro-phosphate (4.0 g, 7.7 mmol), and compound **8b** (1.25 g, 7.7 mmol) were dissolved in dichloromethane (45.0 mL) and stirred at room temperature overnight. The mixture was concentrated *in vacuo*, after which further purification was achieved with column chromatography (n-heptane:ethyl acetate 3:1) to afford **9b** (1.4 g, 55%) as white solid. ^{1}H NMR (400 MHz, chloroform-d) δ 7.12 (d, *J* = 8.3 Hz, 2H), 6.89 (d, *J* = 8.4 Hz, 2H), 6.77 (s, 1H), 4.30 – 4.19 (m, 1H), 3.43 (ddt, *J* = 35.1, 13.5, 6.6 Hz, 2H), 2.74 (t, *J* = 7.2 Hz, 2H), 2.71 – 2.47 (m, 2H), 2.00 (t, *J* = 2.6 Hz, 1H), 1.38 (s, 9H). ^{13}C NMR (101 MHz, $CDCl_3$) δ 170.49, 155.63, 138.41, 135.69, 130.33, 119.35, 80.50, 79.76, 71.74, 53.05, 41.04, 35.22, 28.49, 22.90. ESI-MS [M+Na]: Calculated: 380.4; Observed: 380.

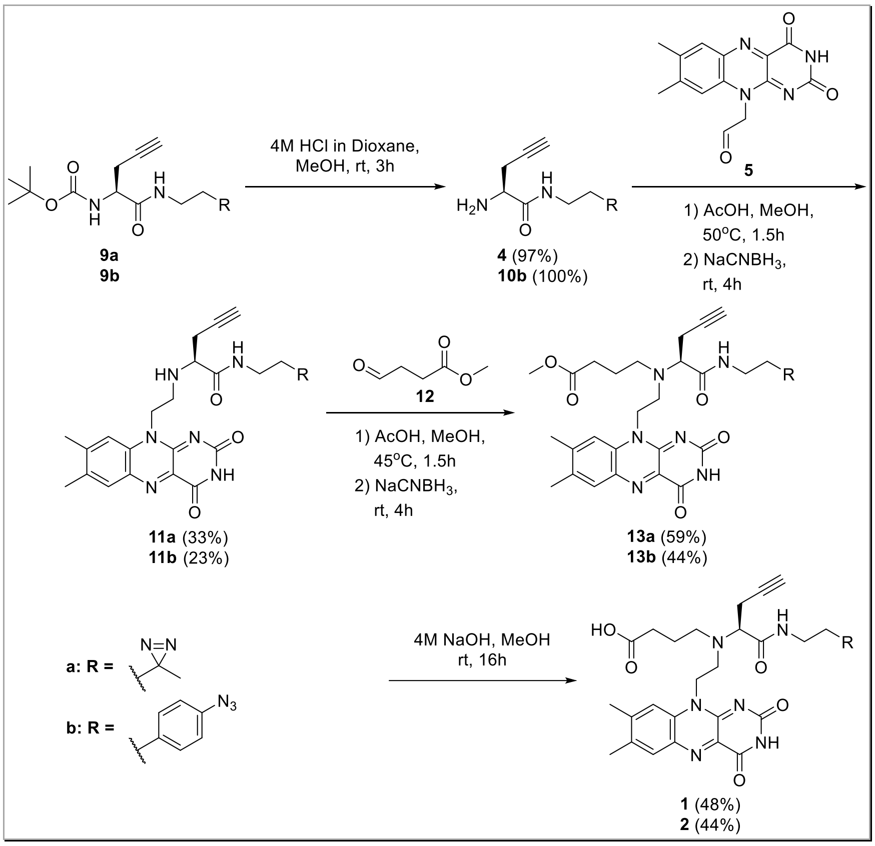

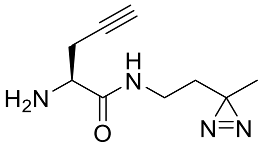

(*S*)-2-Amino-N-(2-(3-methyl-3H-diazirin-3-yl)ethyl)pent-4-ynamide (4)

Compound **9a** (456.9 mg, 1.55 mmol) was dissolved in methanol (2 mL). Subsequently, 4M hydrogen chloride in dioxane (2 mL) was added to the solution and stirred at room temperature for 3h. Afterwards, the mixture was concentrated *in vacuo* to afford **4** (348.6 mg, 97%) as a pale-yellow foam. ^{1}H NMR (400 MHz, dimethyl sulfoxide-d_6) δ 3.89 (t, *J* = 6.0 Hz, 1H), 3.20 – 3.10 (m, 2H), 3.07 – 2.97 (m, 1H), 2.84 – 2.70 (m, 2H), 1.57 – 1.44 (m, 2H), 1.04 (s, 3H). ^{13}C NMR (101 MHz, chloroform-d) δ 172.87, 80.46, 71.20, 53.54, 34.30 (d, *J* = 15.7 Hz), 25.09, 24.45, 19.74. ESI-MS [M+H]: Calculated: 195.2; Observed: 195.19.

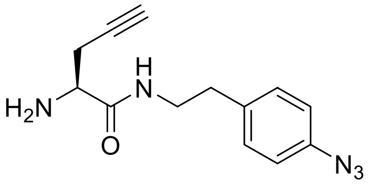

(*S*)-2-Amino-N-(4-azidophenethyl)pent-4-ynamide (10b)

Compound **9b** (501.4 mg, 1.4 mmol) was dissolved in methanol (3 mL). Subsequently, 4M hydrogen chloride in dioxane (2 mL) was added and the mixture was stirred at room temperature for 4 h. Additional 4M hydrogen chloride in dioxane (0.5 ml) was added to the mixture and stirred overnight. Afterwards, the mixture was concentrated *in vacuo* to afford **10b** (455.4 mg, 100%) as a brown foam. Rf-value = 0.30 (dichloromethane: methanol 19:1). ^{1}H NMR (400 MHz, methanol-d_4) δ 7.30 (d, *J* = 8.2 Hz, 2H), 7.00 (dd, *J* = 8.3, 1.6 Hz, 2H), 4.03 (td, *J* = 6.2, 2.5 Hz, 1H), 3.57 (dt, *J* = 14.1, 7.3 Hz, 1H), 3.46 – 3.39 (m, 1H), 2.88 – 2.79 (m, 4H), 2.67 (dt, *J* = 3.3, 1.6 Hz, 1H). ^{13}C NMR (101 MHz, methanol-d_4) δ 167.03, 138.21, 135.84, 130.07, 118.75, 75.87, 73.75, 51.40, 48.11, 47.90, 47.68, 47.47, 47.46, 47.26, 47.25, 47.04, 40.74, 34.29, 21.13. ESI-MS [M+H]: Calculated: 258.3; Observed: 258.0.

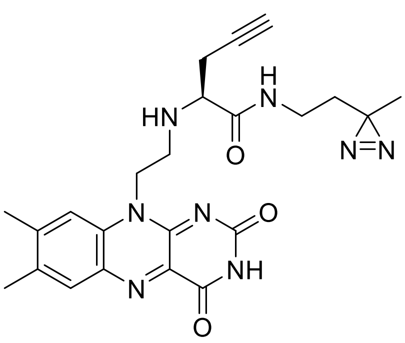

(*S*)-2-((2-(7,8-dimethyl-2,4-dioxo-3,4-dihydrobenzo[g]pteridin-10-(2H)-yl)ethyl)amino)-N-(2-(3-methyl-3H-diazirin-3-yl)ethyl)-pent-4-ynamide (**11a**)

Compound **4** (348.6 mg, 1.51 mmol), compound **5** (476.3 mg, 1.68 mmol) and acetic acid (2.0 mL) were suspended in dry methanol (8.0 mL) and stirred at 50 °C for 1.5 h. Subsequently, sodium cyanoborohydride (478.8 mg, 7.62 mmol) suspended in dry methanol (2.0 mL) was added to the mixture and stirred at room temperature for 1.5 h. Additional acetic acid (1.0 mL) was added to the mixture to stir overnight. Afterwards, the reaction was quenched with saturated aqueous sodium bicarbonate partitioned with ethyl acetate (five times), washed with brine (three times), dried with magnesium sulfate, filtered, and concentrated *in vacuo* to yield a brown oil. Further purification was achieved with column chromatography (dichloromethane:methanol 99:1 to 24:1) to afford **11a** (229.5 mg, 33% yield) as an orange solid. Rf = 0.35 (dichloromethane:methanol 19:1). ^{1}H NMR (400 MHz, chloroform-d) δ 9.14 (s, 1H), 8.06 (d, *J* = 1.0 Hz, 1H), 7.50 (s, 1H), 7.36 (s, 1H), 4.96 – 4.78 (m, 2H), 3.58 (dd, *J* = 7.5, 5.3 Hz, 1H), 3.49 (s, 3H), 3.28 – 3.10 (m, 4H), 2.67 (ddd, *J* = 16.9, 5.3, 2.7 Hz, 1H), 2.62 – 2.51 (m, 4H), 2.48 – 2.44 (m, 3H), 2.04 (d, *J* = 5.2 Hz, 1H), 1.54 (td, *J* = 6.9, 2.4 Hz, 2H), 1.01 (s, 3H). ^{13}C NMR (101 MHz, chloroform-d) δ 172.41, 159.57, 155.69, 150.36, 148.74, 137.46, 136.12, 135.01, 132.89, 131.08, 115.18, 80.16, 71.23, 60.07, 50.80, 44.62, 44.54, 34.65, 34.05, 24.66, 22.87, 21.68, 19.54, 19.49. ESI-MS [M+H]: Calculated: 463.5; Observed: 463.1.

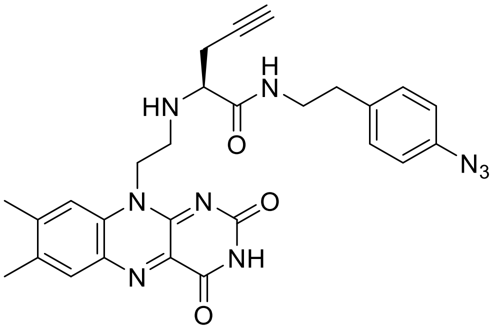

(*S*)-N-(4-azidophenethyl)-2-((2-(7,8-dimethyl-2,4-dioxo-3,4-dihydrobenzo[g]pteridin-10(2H)-yl)ethyl)amino)pent-4-ynamide (**11b**)

Compound **5** (574.5 mg, 2.02 mmol), compound **10b** (412 mg, 1.40 mmol) and acetic acid (1.0 ml) were suspended in methanol (3.0 mL) and stirred at 45 °C for 2.25 h. Subsequently, sodium cyanoborohydride (432.6 mg, 6.9 mmol) dissolved in methanol (0.5 mL) was added to the mixture and stirred at room temperature for 40 h. Afterwards, the reaction was quenched with saturated aqueous sodium bicarbonate and partitioned with ethyl acetate (five times), washed with brine (three times), dried with magnesium sulfate, filtered, and concentrated *in vacuo* to yield a brown oil. Further purification was achieved with column chromatography (dichloromethane:methanol 24:1) to afford **11b** (169.6 mg, 23% yield) as an orange solid. Rf-value = 0.22 (dichloromethane:methanol 24:1). ^{1}H NMR (400 MHz, chloroform-d) δ 10.21 (s, 1H), 7.98 (s, 1H), 7.61 (t, *J* = 6.0 Hz, 1H), 7.49 (s, 1H), 7.13 (d, *J* = 8.0 Hz, 2H), 6.74 (d, *J* = 8.0 Hz, 2H), 4.81 – 4.64 (m, 2H), 3.64 – 3.54 (m, 2H), 3.48 – 3.40 (m, 1H), 3.17 – 3.08 (m, 1H), 3.01 – 2.93 (m, 1H), 2.82 – 2.75 (m, 2H), 2.61 (ddd, *J* = 17.0, 5.7, 2.6 Hz, 2H), 2.55 (s, 3H), 2.54 – 2.49 (m, 1H), 2.42 (s, 3H), 2.05 – 2.01 (m, 1H). ^{13}C NMR (101 MHz, chloroform-d) δ 172.72, 159.82, 156.35, 149.97, 148.78, 137.81, 137.49, 135.91, 135.81, 134.89, 132.54, 130.95, 130.14, 118.76, 115.30, 80.41, 71.10, 59.97, 50.53, 44.56, 44.13, 40.27, 34.69, 23.06, 21.60, 19.49.ESI-MS [M+H]: Calculated: 526.6; Observed: 526.1.

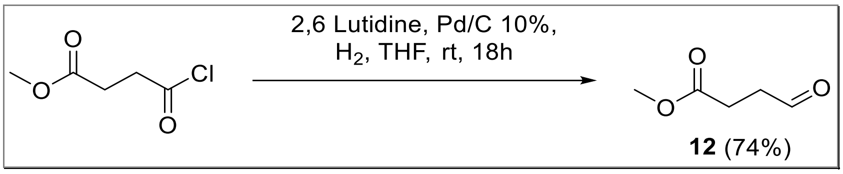

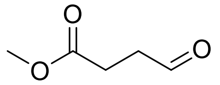

Methyl 4-oxobutanoate (12)

Methyl 4-chloro-4-oxobutyrate (0.6 ml, 4.34 mmol) was dissolved in tetrahydrofuran (10 mL) and degassed with nitrogen gas for 10 minutes. Subsequently, 2,6-lutidine (0.57 mL, 4.38 mmol) and 10% palladium on carbon (50 mg) were added to the reaction mixture and stirred overnight under hydrogen gas. Afterwards, the reaction mixture was filtered through a Celite pad and concentrated *in vacuo* to afford **12** (405 mg, 74% yield) as a pale-yellow liquid. Rf = 0.48 (n-heptane:ethyl acetate 1:1). ^{1}H NMR (400 MHz, chloroform-d) δ 9.82 (t, *J* = 0.7 Hz, 1H), 3.70 (s, 3H), 2.82 – 2.80 (m, 2H), 2.70 – 2.66 (m, 2H).

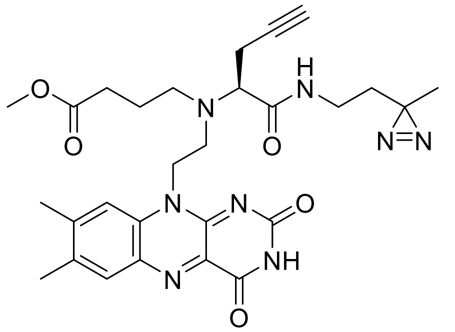

Methyl (*S*)-4-((2-(7,8-dimethyl-2,4-dioxo-3,4-dihydrobenzo[g]-pteridin-10(2H)-yl)ethyl)(1-((2-(3-methyl-3H-diazirin-3-yl)-ethyl)amino)-1-oxopent-4-yn-2-yl)amino)butanoate (13a)

Compound **11a** (229.3 mg, 0.50 mmol), compound **12** (242.3 mg, 2.09 mmol), and acetic acid (1 mL) were dissolved in methanol (4.0 mL) and stirred at 45 °C for 1.5 h. Subsequently, sodium cyanoborohydride (53.6 mg, 0.85 mmol) in methanol (200 µL) was added to the mixture and stirred at room temperature for 1 h, followed by acetic acid (200 µL). This step was carried out twice more to add a total amount of 5 eq. sodium cyanoborohydride (156.1 mg, 2.48 mmol) and stirred for an extra hour to a total of 4h. Afterwards, the reaction was concentrated *in vacuo*, dissolved in ethyl acetate, quenched with saturated aqueous sodium bicarbonate, partitioned with ethyl acetate (three times), washed with brine (three times), dried with magnesium sulfate, filtered, and concentrated *in vacuo* to afford an orange solid. Further purification was achieved with column chromatography (ethyl acetate:methanol 199:1) to afford **13a** (176.1 mg, 63% yield) as an orange solid. ^{1}H NMR (400 MHz, chloroform-d) δ 9.12 (s, 1H), 8.07 (s, 1H), 7.82 (s, 1H), 7.36 (t, *J* = 5.9 Hz, 1H), 4.87 (t, *J* = 9.9 Hz, 1H), 4.68 (s, 1H), 3.79 (dd, *J* = 8.2, 6.3 Hz, 1H), 3.69 (s, 3H), 3.33 – 3.22 (m, 2H), 3.21 – 3.16 (m, 1H), 3.07 (ddd, *J* = 13.5, 9.2, 4.7 Hz, 1H), 2.99 – 2.89 (m, 1H), 2.78 – 2.71 (m, 1H), 2.61 (s, 3H), 2.55 (dd, *J* = 8.1, 2.5 Hz, 1H), 2.53 – 2.49 (m, 1H), 2.48 – 2.46 (m, 3H), 2.43 – 2.38 (m, 2H), 1.99 (t, *J* = 2.6 Hz, 1H), 1.89 (dd, *J* = 14.5, 6.9 Hz, 1H), 1.73 (td, *J* = 7.3, 5.0 Hz, 1H), 1.58 (dd, *J* = 6.9, 1.2 Hz, 2H), 1.01 (s, 3H). ^{13}C NMR (101 MHz, chloroform-d) δ 174.74, 170.22, 159.60, 155.69, 150.07, 149.19, 137.58, 135.90, 135.15, 132.68, 131.22, 115.62, 81.58, 70.39, 62.49, 51.64, 49.71, 47.20, 44.00, 34.77, 34.24, 31.41, 24.69, 23.03, 21.52, 19.56, 19.46, 18.82. ESI-MS [M+Na]: Calculated: 585.6; Observed: 585.1.

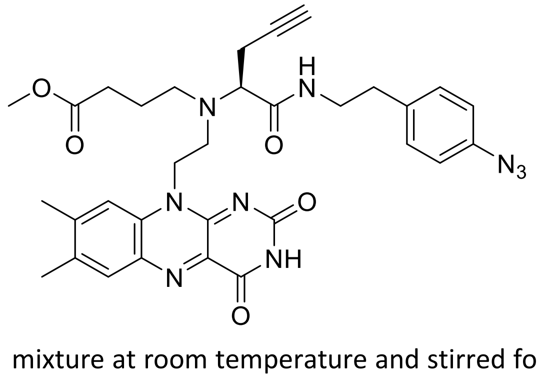

Methyl (*S*)-4-((2-(7,8-dimethyl-2,4-dioxo-3,4-dihydro-benzo-[g]pteridin-10(2H)-yl)ethyl)(1-((2-(3-methyl-3H-diazirin-3-yl)ethyl)amino)-1-oxopent-4-yn-2-yl)amino)-butan-oate (13b)

Compound **11b** (169.0 mg, 0.32 mmol), compound **12** (177.7 mg, 1.53 mmol) and acetic acid (0.5 mL) were dissolved in methanol (2.5 mL) and stirred at 45°C for 1 hour. Subsequently, sodium cyanoborohydride (101.5 mg, 1.61 mmol) in methanol (200 µL) was added to the mixture at room temperature and stirred for 3.5 hours. Afterwards, the reaction was quenched with saturated aqueous sodium bicarbonate, partitioned with ethyl acetate (four times), washed with brine (three times), dried with magnesium sulfate, filtered, and concentrated *in vacuo* to yield a brown oil. Further purification was achieved with column chromatography (ethyl acetate:methanol 99:1) to afford **13b** (87.6 mg, 44% yield) as an orange solid. Rf-value = 0.36 (dichloromethane:methanol 19:1). ^{1}H NMR (400 MHz, chloroform-d) δ 9.50 (s, 1H), 8.08 (s, 1H), 7.81 (s, 1H), 7.41 (t, *J* = 6.0 Hz, 1H), 7.16 – 7.07 (m, 2H), 6.73 – 6.65 (m, 2H), 4.70 (t, *J* = 10.3 Hz, 1H), 4.58 – 4.39 (m, 1H), 3.75 – 3.71 (m, 1H),

3.70 (s, 3H), 3.69 – 3.65 (m, 1H), 3.51 – 3.41 (m, 1H), 3.05 (ddd, *J* = 13.0, 10.5, 6.5 Hz, 1H), 2.92 (ddd, *J* = 13.6, 9.0, 6.8 Hz, 1H), 2.82 (td, *J* = 7.0, 5.0 Hz, 2H), 2.79 – 2.73 (m, 1H), 2.69 (ddd, *J* = 17.1, 6.4, 2.7 Hz, 1H), 2.62 (s, 3H), 2.53 (ddd, *J* = 11.6, 6.1, 2.6 Hz, 1H), 2.47 (s, 4H), 2.46 – 2.42 (m, 1H), 2.41 – 2.35 (m, 2H), 1.97 (t, *J* = 2.6 Hz, 1H), 1.94 – 1.83 (m, 1H), 1.74 – 1.64 (m, 1H). ^{13}C NMR (101 MHz, chloroform-d) δ 174.81, 169.74, 159.61, 155.74, 149.70, 149.28, 137.81, 137.64, 136.15, 135.71, 135.07, 132.59, 131.06, 130.42, 118.57, 115.54, 81.53, 70.28, 62.25, 51.62, 49.49, 46.91, 43.72, 40.39, 34.79, 31.43, 22.95, 21.51, 19.55, 19.05. ESI-MS [M+H]: Calculated: 626.7; Observed: 626.1.

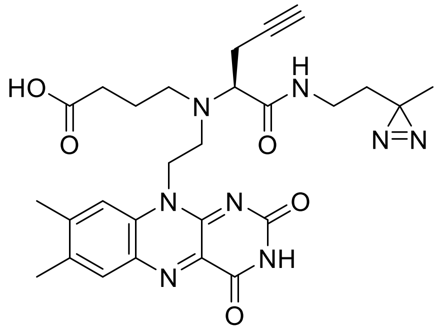

(*S*)-4-((2-(7,8-dimethyl-2,4-dioxo-3,4-dihydrobenzo[g]pteridin-10(2H)-yl)ethyl)(1-((2-(3-methyl-3H-diazirin-3-yl)ethyl)amino)-1-oxopent-4-yn-2-yl)amino)butanoic acid (1)

Compound **13a** (35.4 mg, 0.063 mmol) and 4M sodium hydroxide (1.5 ml) were dissolved in methanol (2.5 ml) and stirred at room temperature overnight. Afterwards, the reaction was neutralized by adding 6M hydrogen chloride (ca. 1 ml), concentrated, resuspended in methanol, filtered, and concentrated to yield a brown solid. Further purification was achieved by column chromatography (dichloromethane:methanol 39:1 to 3:1) to afford **1** (49.4 mg, 48% yield) as an orange solid. Rf-value = 0.49 (dichloromethane:methanol 17:3). ^{1}H NMR (500 MHz, dimethyl sulfoxide-d$_6$) δ 11.34 (s, 1H), 8.11 (t, *J* = 5.8 Hz, 1H), 7.86 (s, 1H), 7.82 (s, 1H), 4.73 – 4.64 (m, 1H), 4.63 – 4.55 (m, 1H), 3.51 (t, *J* = 7.2 Hz, 1H), 3.05 – 3.01 (m, 1H), 3.00 – 2.95 (m, 2H), 2.92 – 2.84 (m, 1H), 2.77 – 2.71 (m, 1H), 2.69 (t, *J* = 2.6 Hz, 1H), 2.58 (dq, *J* = 8.4, 4.4, 3.4 Hz, 1H), 2.52 (s, 3H), 2.50 – 2.45 (m, 1H), 2.40 (s, 3H), 2.26 (ddd, *J* = 16.8, 6.7, 2.6 Hz, 1H), 2.07 – 2.01 (m, 2H), 1.67 – 1.45 (m, 3H), 1.43 (td, *J* = 7.2, 1.4 Hz, 2H), 1.00 (s, 3H). ^{13}C NMR (126 MHz, dimethyl sulfoxide-d$_6$) δ 170.71, 160.36, 156.14, 150.54, 147.07, 137.38, 136.30, 134.26, 131.43, 116.70, 82.54, 72.80, 62.67, 51.73, 49.05, 47.46, 43.99, 34.31, 34.26, 34.22, 25.22, 24.58, 21.11, 19.72, 19.25, 18.28. ESI-MS [M+H]: Calculated: 549.6; Observed: 549.1.

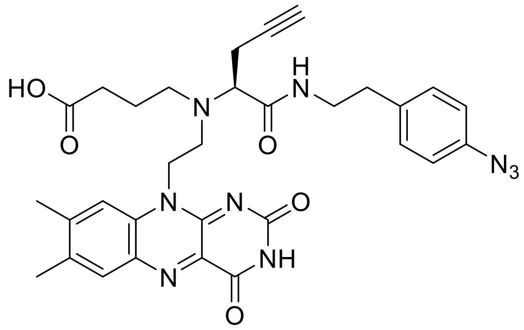

(*S*)-4-((1-((4-azidophenethyl)amino)-1-oxopent-4-yn-2-yl)(2-(7,8-dimethyl-2,4-dioxo-3,4-dihydrobenzo[g]-pteridin-10(2H)-yl)ethyl)amino)butanoic acid (2)

Compound **13b** (87.6 mg, 0.14 mmol) and 2M sodium hydroxide (1.5 ml) were dissolved in methanol (4.0 ml) and stirred at room temperature for 5 h. Additional 4M sodium hydroxide (2.5 ml) was added to the mixture and stirred overnight. Afterwards, the reaction was neutralized with 6M hydrogen chloride (ca. 2 ml), concentrated *in vacuo*, resuspended in chloroform, filtered, and concentrated *in vacuo* to yield a brown solid. Further purification was achieved by column chromatography (dichloromethane:methanol 39:1 to 9:1) to afford **2** (37.8 mg, 44% yield) as an orange solid. ^{1}H NMR (400 MHz, chloroform-d) δ 10.18 (s, 1H), 7.95 (s, 1H), 7.64 (s, 1H), 7.53 (s, 1H), 7.12 (d, *J* = 8.0 Hz, 2H), 6.72 (d, *J* = 7.8 Hz, 2H), 4.83 – 4.42 (m, 2H), 3.71 – 3.63 (m, 1H), 3.63 – 3.52 (m, 1H), 3.48 – 3.42 (m, 1H), 3.15 – 3.00 (m, 1H), 2.96 – 2.83 (m, 2H), 2.83 – 2.78 (m, 2H), 2.78 – 2.72 (m, 1H), 2.57 (s, 3H), 2.55 – 2.44 (m, 2H), 2.42 (s, 3H), 2.39 – 2.30 (m, 2H), 1.99 (s, 1H), 1.87 – 1.69 (m, 2H). ^{13}C NMR (101 MHz, chloroform-d) δ 170.64, 159.84, 156.97, 149.63, 149.48, 137.92, 137.84, 135.98, 135.54, 135.08, 132.40, 130.87, 130.28, 118.74, 115.61, 81.73, 70.77, 62.70, 50.62, 47.00, 43.83, 40.56, 34.75, 32.08, 23.15, 21.57, 19.53, 17.85. ESI-MS [M+Na]: Calculated: 634.7; Observed: 634.1.

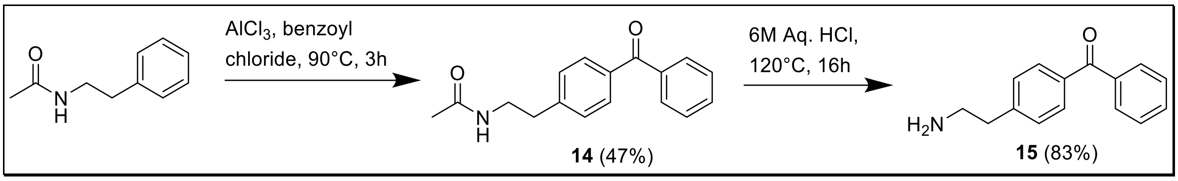

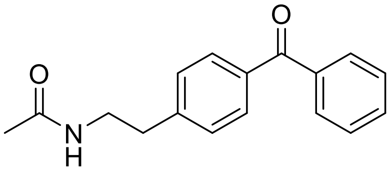

N-(4-benzoylphenethyl)acetamide (14)

N-phenethylacetamide (3.02 g, 18.5 mmol) was dissolved in benzoyl chloride (3.5 mL, 30.3 mmol), aluminium chloride (3.5 g, 26.2 mmol) was added and the reaction mixture was stirred at 90°C for 3h. A total of 3.16 g (23.7 mmol) aluminium chloride and 2 mL (17.3 mmol) benzoyl chloride were added after 1 hour and 2 hour reaction times respectively. After 3 hours the reaction was diluted with saturated aqueous sodium bicarbonate and extracted with ethyl acetate (three times). The combined organic fractions were washed with brine and dried with magnesium sulfate. Further purification was achieved by column chromatography (EtOAc:Heptane 1:1 to EtOAc) to afford **14** (2.32 g, 47% yield) as yellow oil. Rf-value = 0.24 (Heptane:EtOAc 2:1). ^{1}H NMR (400 MHz, chloroform-d) δ 7.81 – 7.70 (m, 4H), 7.64 – 7.55 (m, 1H), 7.53 – 7.44 (m, 2H), 7.36 – 7.29 (m, 2H), 6.55 (s, 1H), 3.55 (td, J = 7.2, 5.9 Hz, 2H), 2.93 (t, J = 7.2 Hz, 2H), 1.97 (s, 3H).

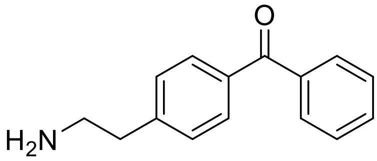

(4-(2-aminoethyl)phenyl)(phenyl)methanone (15)

Compound **14** (357 mg, 1.34 mmol) was dissolved in 6M aqueous hydrochloric acid (12 mL) and stirred at 120°C for 16h. The reaction was diluted with 6M aqueous hydrochloric acid and washed with diethyl ether (two times). Afterwards, the water layer was basified (pH>11) using 6M aqueous potassium hydroxide and extracted with diethyl ether (three times). These three diethyl ether layers were combined, washed with brine and dried with magnesium sulfate. Concentrating *in vacuo* resulted in compound **15** (251 mg, 83%) as yellow oil. Rf-value = 0 (EtOAc). ^{1}H NMR (400 MHz, chloroform-d) δ 7.81 – 7.70 (m, 4H), 7.59 – 7.51 (m, 1H), 7.49 – 7.40 (m, 2H), 7.29 (d, J = 8.0 Hz, 2H), 3.00 (t, J = 7.0 Hz, 2H), 2.81 (t, J = 6.9 Hz, 2H), 1.39 (s, 3H).

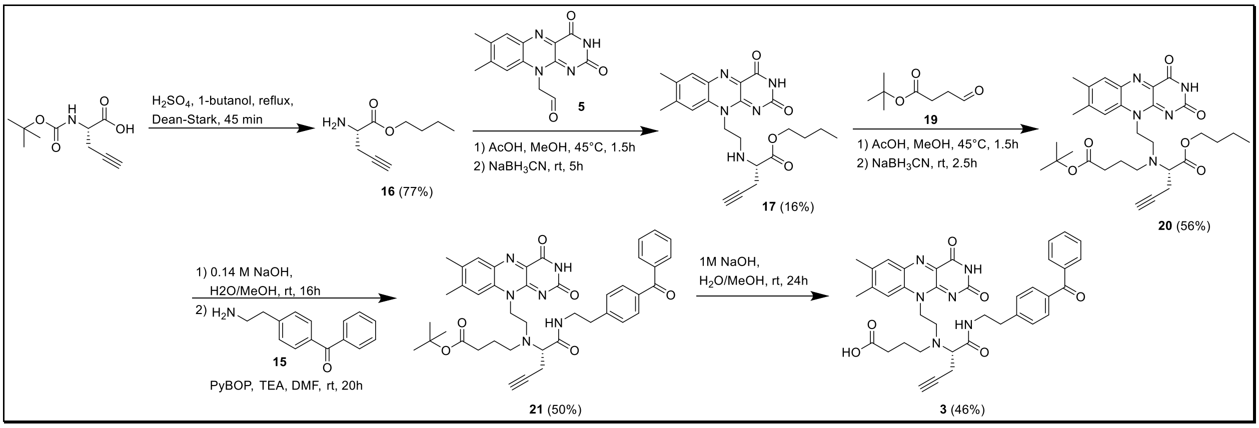

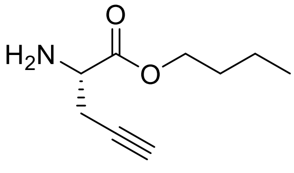

Butyl (*S*)-2-aminopent-4-ynoate (16)

Boc-L-propargylglycine (416 mg, 1.95 mmol) was dissolved in 1-butanol (45 mL). 98% Sulfuric acid (0.18 mL) was added to the reaction and the reaction was heated at reflux in a Dean-Stark set-up for 45 minutes. The reaction was cooled on ice, diluted with ethyl acetate and washed with saturated aqueous sodium bicarbonate. The water layer was extracted with ethyl acetate (three times) and the combined organic fractions were washed with water and brine, dried with magnesium sulfate and carefully concentrated *in vacuo* to afford **16** (254 mg, 77% yield) as slightly yellow oil. Rf-value = 0.26 (97:3 EtOAc:TEA). ^{1}H NMR (400 MHz, chloroform-d) δ 4.08 (qt, J = 10.8, 6.6 Hz, 2H), 3.55 (dd, J = 6.5, 4.7 Hz, 1H), 2.63 – 2.48 (m, 2H), 2.00 (t, J = 2.7 Hz, 1H), 1.98 (s, 2H), 1.62 – 1.50 (m, 2H), 1.39 – 1.25 (m, 2H), 0.85 (t, J = 7.3 Hz, 4H). ^{13}C NMR (101 MHz, chloroform-d) δ 173.70, 79.44, 71.24, 65.11, 53.04, 30.54, 24.70, 19.00, 13.57. ESI-MS [M+H]: Calculated: 170.11; Observed: 170.23.

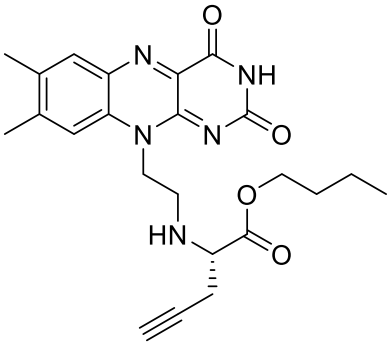

Butyl 2-((2-(7,8-dimethyl-2,4-dioxo-3,4-dihydrobenzo[g]pteridin-10(2H)-yl)ethyl)amino)pent-4-ynoate (17)

Compound **5** (3.024 g, 10.6 mmol), compound **16** (1.498 g, 8.85 mmol) and glacial acetic acid (25 mL) were suspended in dry methanol (50 mL). The resulting suspension was stirred for 1.5h at 45 °C under inert atmosphere. Next, sodium cyanoborohydride (1.597 g, 25.4 mmol) dissolved in dry methanol (3 mL) and glacial acetic acid (2 mL) were added to the reaction mixture after which the reaction was stirred at room temperature. Over the next 5 hours, a total of 1.880 g (29.9 mmol) sodium cyanoborohydride was added over 3 portions, each time dissolved in dry methanol (2 mL) and accompanied by glacial acetic acid (1 mL). Afterwards, the reaction was filtered in order to remove some of the residual compound **5** to ease the following extraction. The filtrate was diluted with saturated aqueous sodium bicarbonate and extracted with ethyl acetate (four times). The combined organic fractions were washed with brine and dried with magnesium sulfate. Further purification was achieved by column chromatography (DCM: MeOH 98:2 to DCM:MeOH 95:5) to obtain **17** (628 mg, 16% yield) as orange solid. Rf-value = 0.56 (DCM:MeOH 95:5). ^{1}H NMR (500 MHz, chloroform-d) δ 8.52 (s, 1H), 8.05 (s, 1H), 7.58 (s, 1H), 4.85 (q, J = 7.0 Hz, 2H), 4.20 – 4.06 (m, 2H), 3.51 (t, J = 5.8 Hz, 1H), 3.30 – 3.23 (m, 1H), 3.14 – 3.05 (m, 1H), 2.56 (s, 3H), 2.58 – 2.53 (m, 2H), 2.45 (d, J = 0.9 Hz, 3H), 1.97 (t, J = 2.6 Hz, 1H), 1.67 – 1.56 (m, 2H), 1.42 – 1.31 (m, 2H), 0.92 (t, J = 7.4 Hz, 3H). ^{13}C NMR (126 MHz, chloroform-d) δ 172.72, 159.55, 155.04, 150.52, 148.30, 137.08, 136.07, 134.98, 132.74, 131.46, 115.78, 79.16, 71.28, 65.21, 59.36, 45.11, 44.53, 30.59, 23.23, 21.57, 19.51, 19.07, 13.63. ESI-MS [M+H]: Calculated: 438.21; Observed: 438.08.

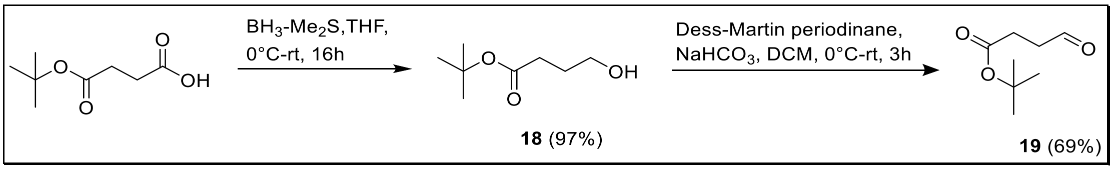

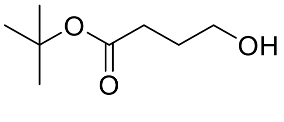

tert-Butyl 4-hydroxybutanoate (18)

Synthesis as previously described[18]. Borane dimethyl sulfide complex (1.03 mL, 10.8 mmol) was mixed with dry THF (5 mL) under inert atmosphere. In a separate flask, mono-*tert*-butyl succinate (878 mg, 5.0 mmol) was dissolved in dry THF (8 mL). The

resulting solution was stirred at 0 °C under inert atmosphere and the previously prepared borane dimethyl sulfide in THF solution (3.03 mL) was added dropwise. The reaction was stirred at room temperature for 16 hours. The reaction was diluted with ethyl acetate. The organic fraction was washed with water (two times), washed with brine, dried with magnesium sulfate and concentrated *in vacuo* to yield **18** (788 mg, 97% yield) as colorless liquid. Rf-value = 0.27 (2:1 Heptane: EtOAc). ^{1}H NMR (400 MHz, chloroform-d) δ 3.66 (t, J = 6.2 Hz, 2H), 2.34 (t, J = 7.1 Hz, 2H), 1.89 – 1.78 (m, 2H), 1.44 (s, 9H).

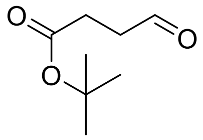

tert-Butyl 4-oxobutanoate (19)

Compound **18** (711 mg, 4.44 mmol) and sodium bicarbonate (747 mg, 8.89 mmol) were suspended in dry DCM (70 mL). The resulting suspension was stirred at 0 °C under inert atmosphere while Dess-Martin Periodinane (DMP) (3.46 g, 8.16 mmol) suspended in anhydrous DCM (40 mL) was added dropwise. Next, the reaction was stirred at room temperature for 3 hours. Afterwards, a 1:1 thiosulfate:saturated aqueous sodium bicarbonate solution (100 mL) was added to the reaction mixture and stirred for 30 minutes until the mixture was a clear colorless solution. The mixture was extracted with ethyl acetate (four times) and the combined organic fractions were washed with water and brine, dried with magnesium sulfate and concentrated *in vacuo* to yield **19** (481 mg, 69%) as colorless liquid. ^{1}H NMR (400 MHz, chloroform-d) δ 9.75 (t, J = 0.9 Hz, 1H), 2.72 – 2.64 (m, 2H), 2.54 – 2.46 (m, 2H), 1.39 (s, 9H).

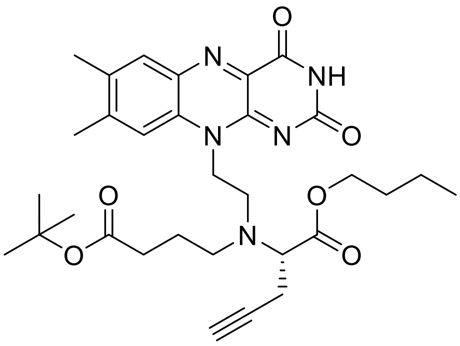

Butyl 2-((4-(tert-butoxy)-4-oxobutyl)(2-(7,8-dimethyl-2,4-dioxo-3,4-dihydrobenzo[g]pteridin-10(2H)-yl)ethyl)amino)pent-4-ynoate (20)

Compound **17** (133 mg, 0.304 mmol), compound **19** (314 mg, 1.98 mmol) and glacial acetic acid (2.5 mL) were dissolved in dry methanol (5 mL). The resulting mixture was stirred for 1.5 hours at 45°C under inert atmosphere. Sodium cyanoborohydride (141 mg, 2.24 mmol) dissolved in anhydrous methanol (1 mL) was added and the reaction was stirred at room temperature. After 30 minutes, glacial acetic acid (1.5 mL) was added to the reaction mixture and after an additional 60 minutes, sodium cyanoborohydride (161 mg, 2.56 mol) dissolved in dry methanol (1 mL) was added to the reaction mixture. After 60 minutes, the reaction was diluted with saturated aqueous sodium bicarbonate and extracted with ethyl acetate (five times). The combined organic fractions were washed with brine, dried with magnesium sulfate and concentrated *in vacuo*. Further purification was achieved by column chromatography (DCM:MeOH 98:2 to DCM:MeOH 95:5) to obtain **20** (96 mg, 56% yield) as orange solid. Rf-value = 0.59 (DCM:MeOH 95:5). ^{1}H NMR (500 MHz, chloroform-d) δ 8.68 (s, 1H), 8.04 (s, 1H), 7.67 (s, 1H), 4.85 – 4.80 (m, 1H), 4.78 – 4.73 (m, 1H), 4.13 – 4.03 (m, 2H), 3.70 (dd, J = 8.8, 6.0 Hz, 1H), 3.20 – 3.05 (m, 2H), 2.87 – 2.72 (m, 2H), 2.64 – 2.52 (m, 1H), 2.58 (s, 4H), 2.49 – 2.42 (m, 1H), 2.45 (s, 3H), 2.26 (td, J = 7.2, 2.4 Hz, 2H), 2.10 (s, 1H), 1.92 (t, J = 2.6 Hz, 1H), 1.79 – 1.63 (m, 2H), 1.63 – 1.56 (m, 2H), 1.44 (d, J = 8.0 Hz, 2H), 1.44 (s, 9H), 1.40 – 1.31 (m, 2H), 0.91 (t, J = 7.4 Hz, 3H). ^{13}C NMR (126 MHz, chloroform-d) δ 172.82, 171.45, 159.64, 155.10, 150.28, 148.46, 137.06, 136.10, 134.99, 132.69, 131.42, 115.75, 80.76, 80.29, 70.54, 64.84, 61.98, 51.49, 47.56, 44.43, 32.43, 30.63, 28.13, 23.68, 21.42, 20.19, 19.50, 19.16, 13.64. ESI-MS [M+H]: Calculated: 580.31; Observed: 580.26

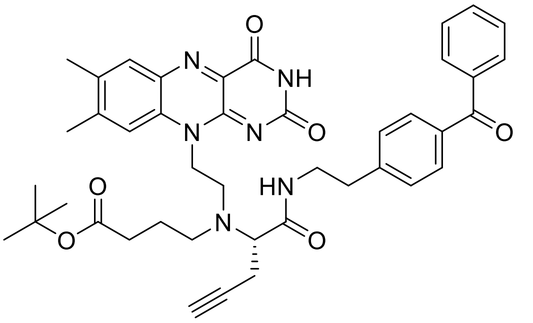

tert-Butyl 4-((1-((4-benzoylphenethyl)amino)-1-oxopent-4-yn-2-yl)(2-(7,8-dimethyl-2,4-dioxo-3,4-dihydrobenzo[g]pteridin-10(2H)-yl)ethyl)amino)butanoate (**21**)

To a solution of compound **20** (150 mg 0.259 mmol) in methanol (3 mL) was added a mixture of methanol (2.25 mL) and 0.46M aqueous sodium hydroxide (2.25 mL). The resulting solution was stirred for 16h. When TLC showed full conversion, the reaction was diluted with methanol and neutralized using a 0.1M hydrogen chloride in methanol solution. The reaction was concentrated *in vacuo* and the product was used without further purification or characterization. Rf-value = 0 (DCM:MeOH 95:5).

The product was dissolved in dry DMF (4 mL) and benzotriazol-1-yloxytripyrrolidinophosphonium hexafluorophosphate (PyBOP) (164 mg, 0.315 mmol), triethylamine (0.11 mL, 0.792 mmol) and compound **15** (87 mg, 0.386 mmol) in dry DMF (1 mL) was added. The resulting solution was stirred for 20h. Next, the reaction was diluted with water and extracted with ethyl acetate (three times). The combined organic fractions were washed with brine, dried with magnesium sulfate and concentrated *in vacuo*. Further purification was achieved by column chromatography (DCM:MeOH 98:2 to 96:4) to yield **21** (95 mg, 50% yield) as orange oil. Rf-value = 0.64 (DCM:MeOH 95:5). ^{1}H NMR (500 MHz, chloroform-d) δ 8.69 (s, 1H), 8.06 (s, 1H), 7.79 (s, 1H), 7.69 – 7.64 (m, 2H), 7.59 – 7.57 (m, 2H), 7.57 – 7.52 (m, 1H), 7.45 – 7.41 (m, 2H), 7.31 – 7.28 (m, 2H), 7.25 (s, 0H), 4.66 – 4.62 (m, 1H), 4.54 – 4.51 (m, 1H), 3.77 – 3.64 (m, 2H), 3.64 – 3.53 (m, 1H), 3.09 – 2.99 (m, 1H), 3.01 – 2.83 (m, 3H), 2.82 – 2.74 (m, 1H), 2.70 (ddd, J = 17.1, 6.4, 2.8 Hz, 1H), 2.60 (s, 3H), 2.52 (s, 0H), 2.56 – 2.41 (m, 5H), 2.34 – 2.25 (m, 2H), 1.96 – 1.94 (m, 1H), 1.88 – 1.78 (m, 1H), 1.70 – 1.64 (m, 1H), 1.45 (s, 9H). ^{13}C NMR (126 MHz, chloroform-d) δ 195.96, 173.65, 170.03, 159.43, 155.23, 149.86, 149.21, 144.38, 137.51, 135.70, 135.57, 135.15, 133.11, 132.67, 132.26, 130.14, 129.86, 128.92, 128.19, 127.87, 115.57, 81.53, 80.31, 70.34, 62.44, 49.79, 46.94, 43.99, 40.08, 35.49, 32.66, 28.29, 28.24, 28.13, 22.80, 21.44, 19.55, 19.10. ESI-MS [M+H]: Calculated: 731.35; Observed: 731.28

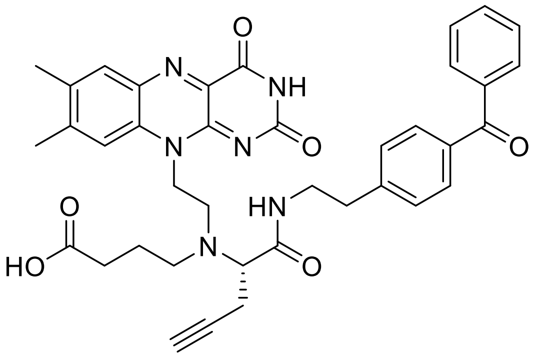

4-((1-((4-benzoylphenethyl)amino)-1-oxopent-4-yn-2-yl)(2-(7,8-dimethyl-2,4-dioxo-3,4-dihydrobenzo[g]pteridin-10(2H)-yl)ethyl)amino)butanoic acid (**3**)

To a solution of compound **21** (25 mg, 0.0342 mmol) in methanol (0.5 mL) was added a mixture of methanol (0.5 mL) and 3M aqueous sodium hydroxide (0.5 mL). The resulting solution was stirred for 24h. The reaction was diluted with methanol, neutralized with 0.1M hydrogen chloride in methanol and concentrated *in vacuo*. Further purification was achieved by column chromatography (DCM:MeOH 95:5 to 82:18) to afford **3** (10.6 mg, 46% yield) as orange solid. Rf-value = 0 (DCM:MeOH 95:5). ^{1}H NMR (500 MHz, methanol-d$_4$) δ 7.80 (s, 1H), 7.70 (s, 1H), 7.56 (d, J = 7.6 Hz, 2H), 7.53 – 7.48 (m, 3H), 7.43 (dd, J = 8.4, 7.1 Hz, 2H), 7.33 (d, J = 8.0 Hz, 2H), 4.61 – 4.50 (m, 2H), 3.69 – 3.59 (m, 2H), 3.48 – 3.38 (m, 1H), 3.03 – 2.91 (m, 2H), 2.90 – 2.84 (m, 1H), 2.83 – 2.78 (m, 1H), 2.78 – 2.70 (m, 1H), 2.62 – 2.56 (m, 2H), 2.55 (s, 3H), 2.46 – 2.40 (m, 1H), 2.40 (s, 3H), 2.29 – 2.21 (m, 3H), 1.73 – 1.60 (m, 2H). ^{13}C NMR (126 MHz, methanol-d$_4$) δ 196.37, 171.56, 160.70, 157.04, 150.00, 148.63, 144.75, 137.44, 137.16, 135.81, 135.26, 134.66, 132.26, 131.25, 131.16, 129.87, 129.45, 128.83, 128.01, 116.04, 80.77, 70.28, 62.69, 59.38, 50.26, 46.86, 43.42, 39.56, 34.83, 31.36, 23.38, 21.14, 20.00, 18.02. ESI-MS [M+H]: Calculated: 675.29; Observed: 675.24.

SPECTRA

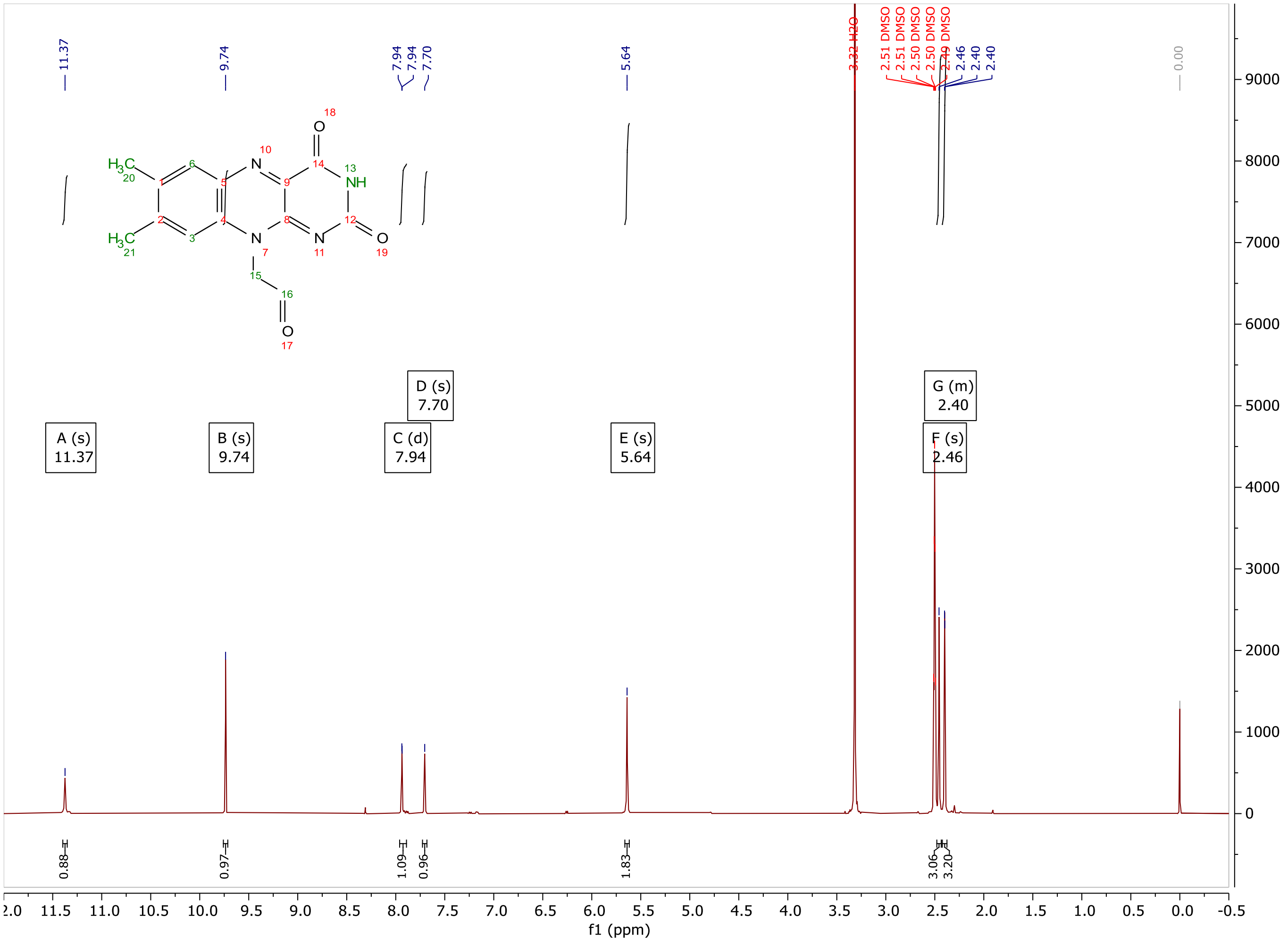

11.37
9.74
7.94
7.94
7.70
5.64
3.32 H2O
2.51 DMSO
2.51 DMSO
2.50 DMSO
2.50 DMSO
2.49 DMSO
2.46
2.40
2.40
0.00
A (s) 11.37
B (s) 9.74
C (d) 7.94
D (s) 7.70
E (s) 5.64
F (s) 2.46
G (m) 2.40
0.88
0.97
1.09
0.96
1.83
3.06
3.20
f1 (ppm)

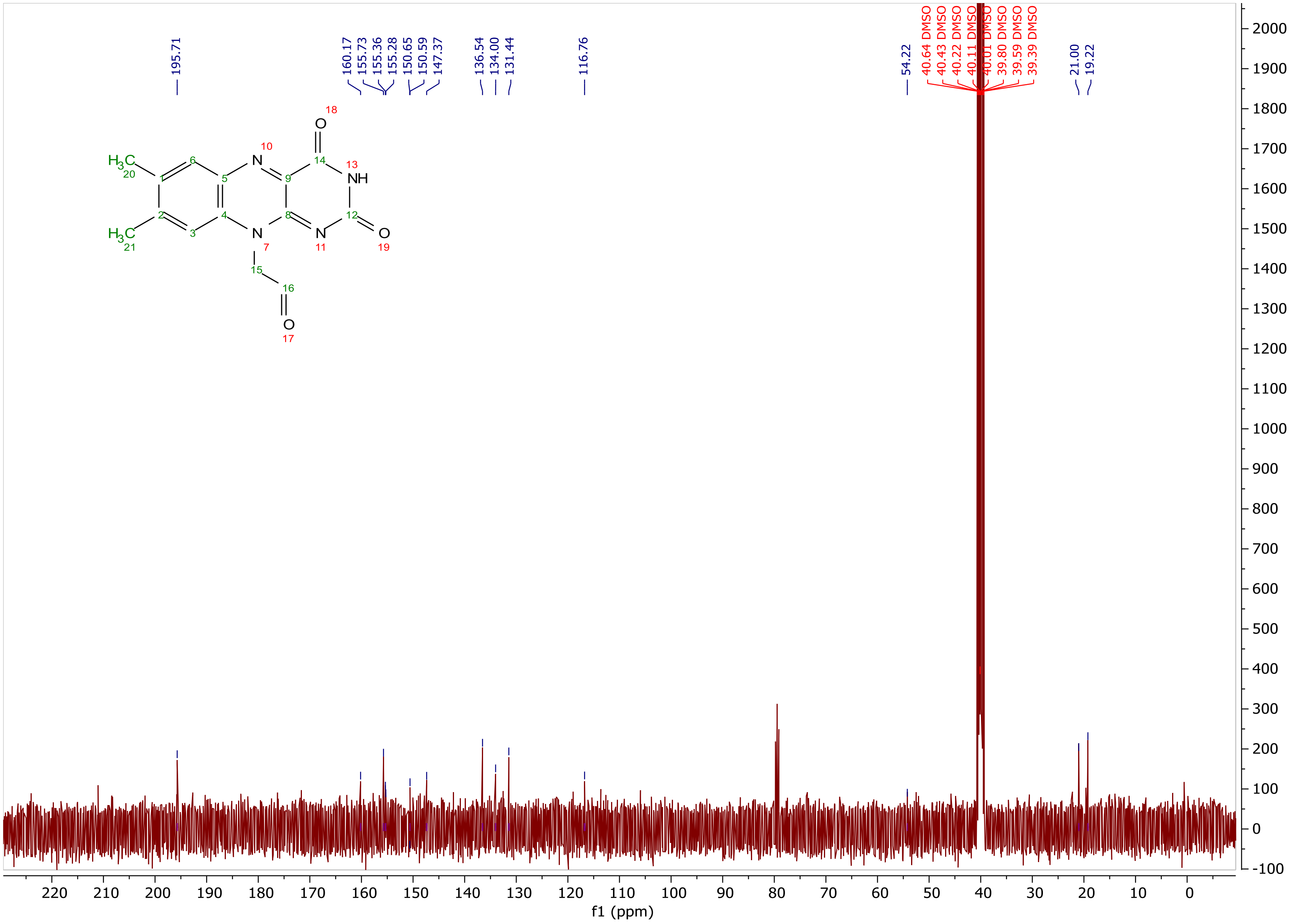

195.71
160.17
155.73
155.36
155.28
150.65
150.59
147.37
136.54
134.00
131.44
116.76
54.22
40.64 DMSO
40.43 DMSO
40.22 DMSO
40.11 DMSO
40.01 DMSO
39.80 DMSO
39.59 DMSO
39.39 DMSO
21.00
19.22
f1 (ppm)

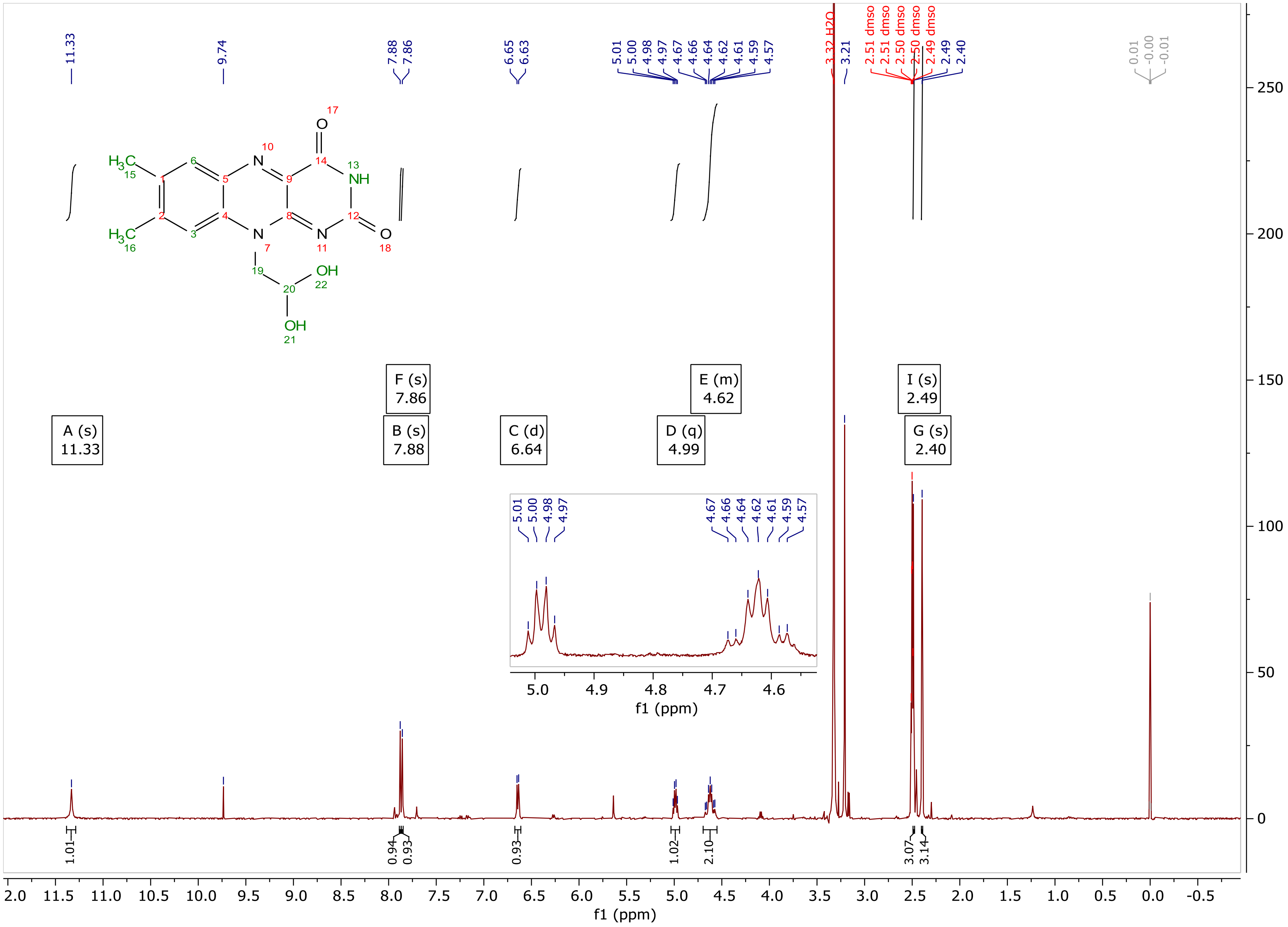

A (s) 11.33
B (s) 7.88
C (d) 6.64
D (q) 4.99
E (m) 4.62
F (s) 7.86
G (s) 2.40
I (s) 2.49
3.32 H2O
2.51 dmso
2.50 dmso
2.49 dmso
f1 (ppm)

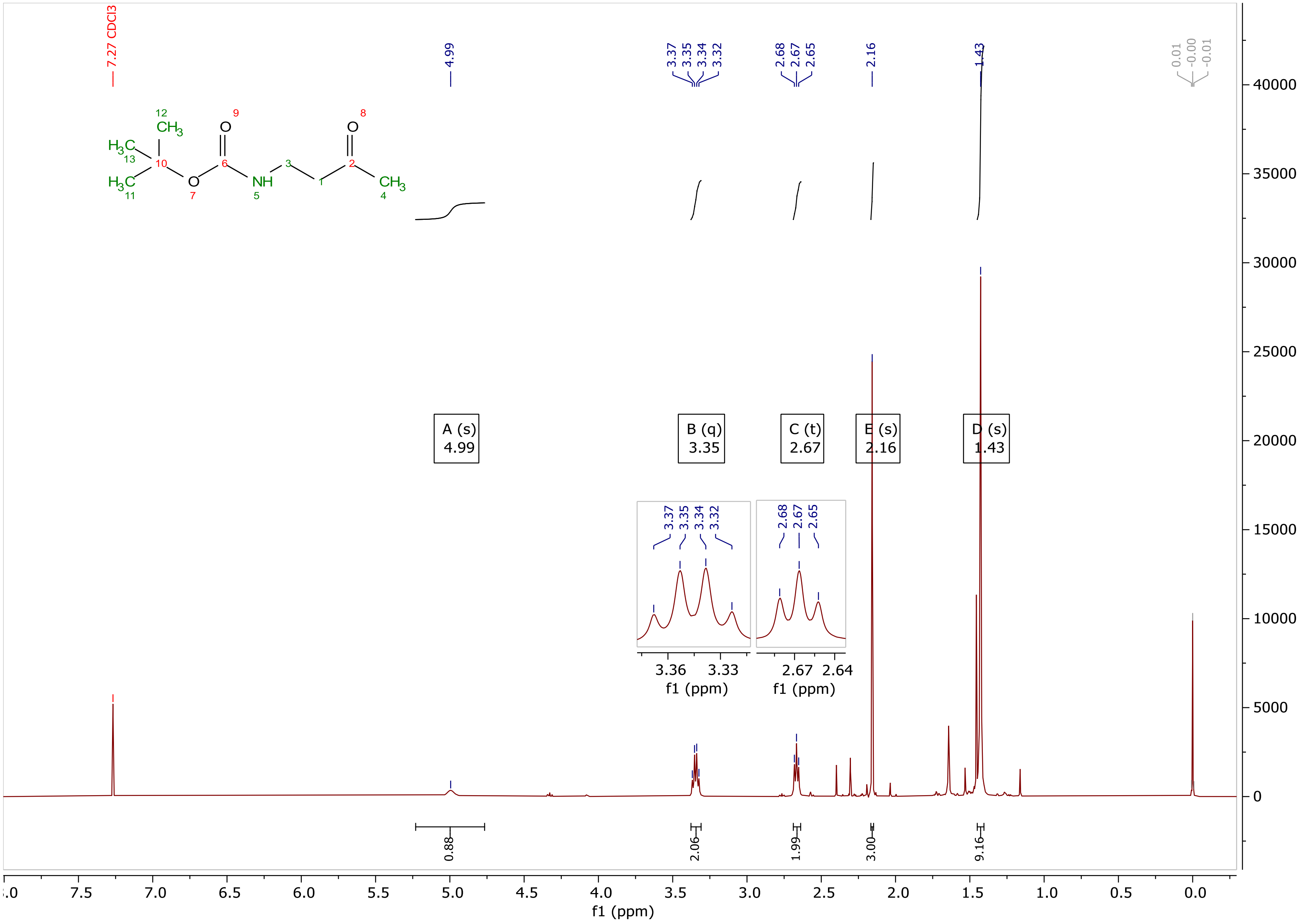
7.27 CDCl3
4.99
3.37 3.35 3.34 3.32
2.68 2.67 2.65
2.16
1.43
0.01 -0.00 -0.01
A (s) 4.99
B (q) 3.35
C (t) 2.67
E (s) 2.16
D (s) 1.43
0.88
2.06
1.99
3.00
9.16
f1 (ppm)

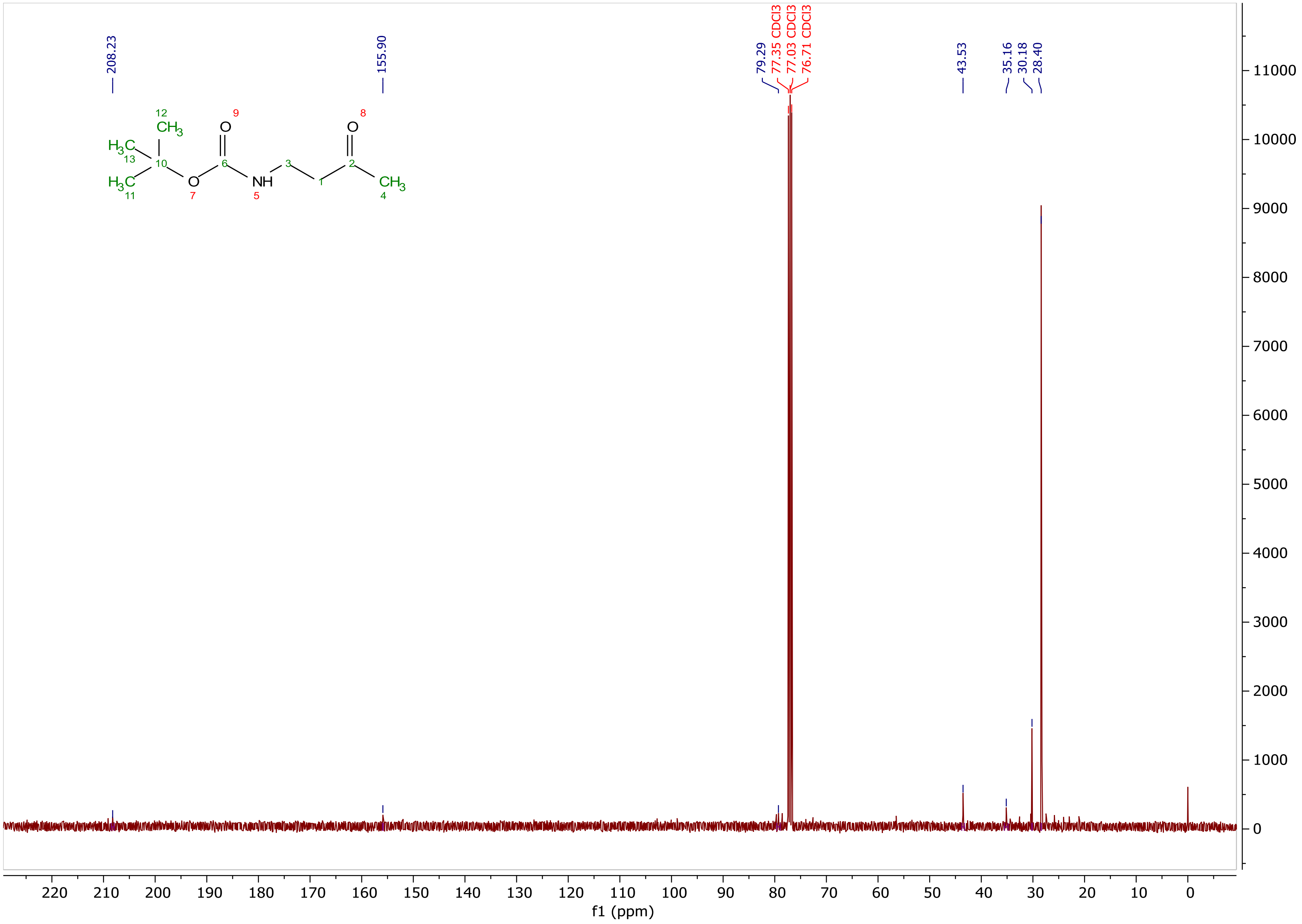
208.23
155.90
79.29
77.35 CDCl3
77.03 CDCl3
76.71 CDCl3
43.53
35.16
30.18
28.40
f1 (ppm)

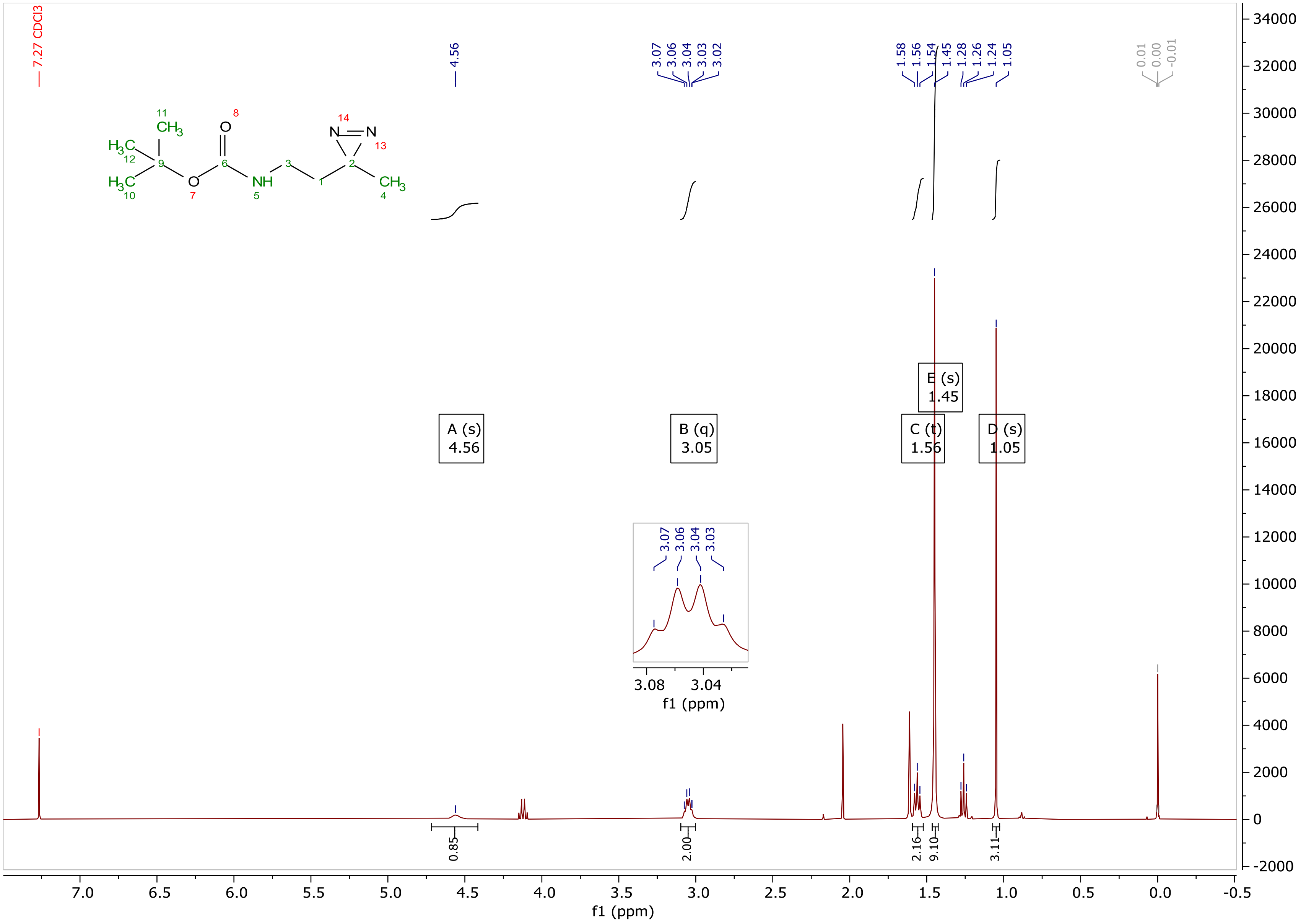
7.27 CDCl3
4.56
3.07
3.06
3.04
3.03
3.02
1.58
1.56
1.54
1.45
1.28
1.26
1.24
1.05
0.01
0.00
-0.01
A (s)
4.56
B (q)
3.05
C (t)
1.56
E (s)
1.45
D (s)
1.05
0.85
2.00
2.16
9.10
3.11
f1 (ppm)

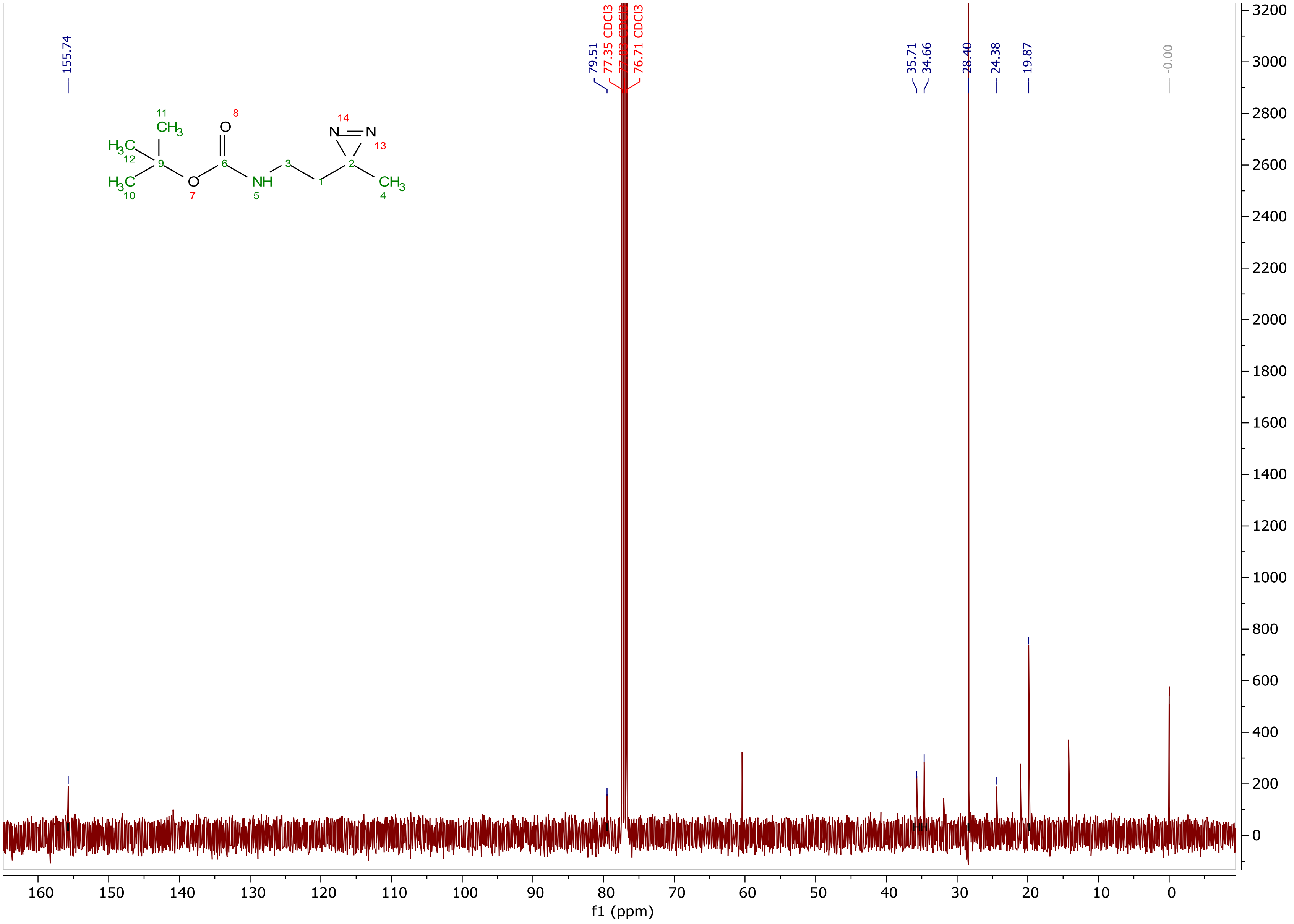

155.74
79.51
77.35 CDCl3
77.03 CDCl3
76.71 CDCl3
35.71
34.66
28.40
24.38
19.87
-0.00
11
CH3
8
O
14
N
N
13
H3C
12
9
6
3
2
H3C
10
O
7
NH
5
1
CH3
4
3200
3000
2800
2600
2400
2200
2000
1800
1600
1400
1200
1000
800
600
400
200
0
160
150
140
130
120
110
100
90
80
70
60
50
40
30
20
10
0
f1 (ppm)

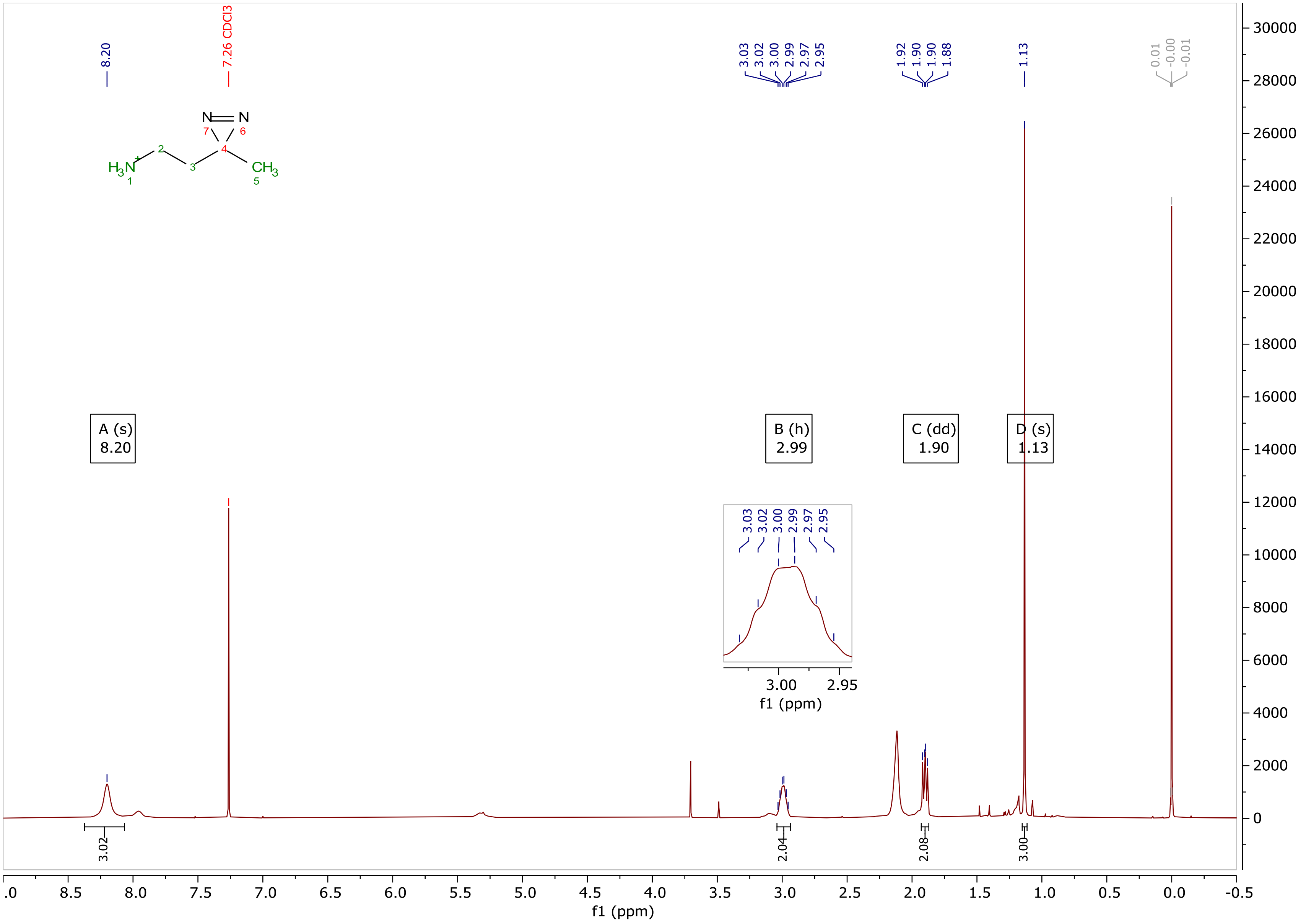

8.20
7.26 CDCl3
3.03
3.02
3.00
2.99
2.97
2.95
1.92
1.90
1.90
1.88
1.13
0.01
-0.00
-0.01
A (s)
8.20
B (h)
2.99
C (dd)
1.90
D (s)
1.13
3.02
2.04
2.08
3.00
f1 (ppm)

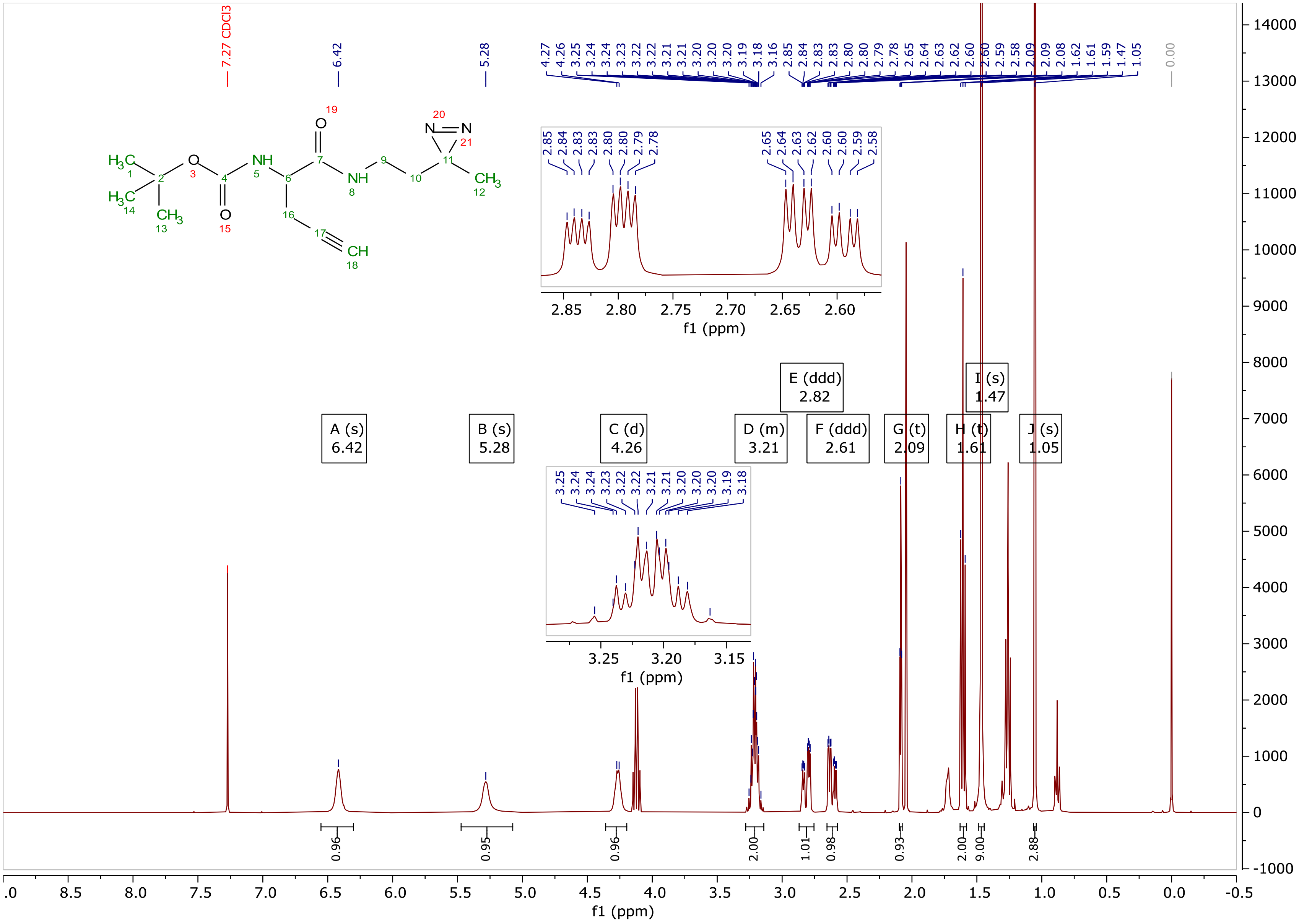
7.27 CDCl3
A (s) 6.42
B (s) 5.28
C (d) 4.26
D (m) 3.21
E (ddd) 2.82
F (ddd) 2.61
G (t) 2.09
H (t) 1.61
I (s) 1.47
J (s) 1.05
0.96
0.95
0.96
2.00
1.01
0.98
0.93
2.00
9.00
2.88
f1 (ppm)

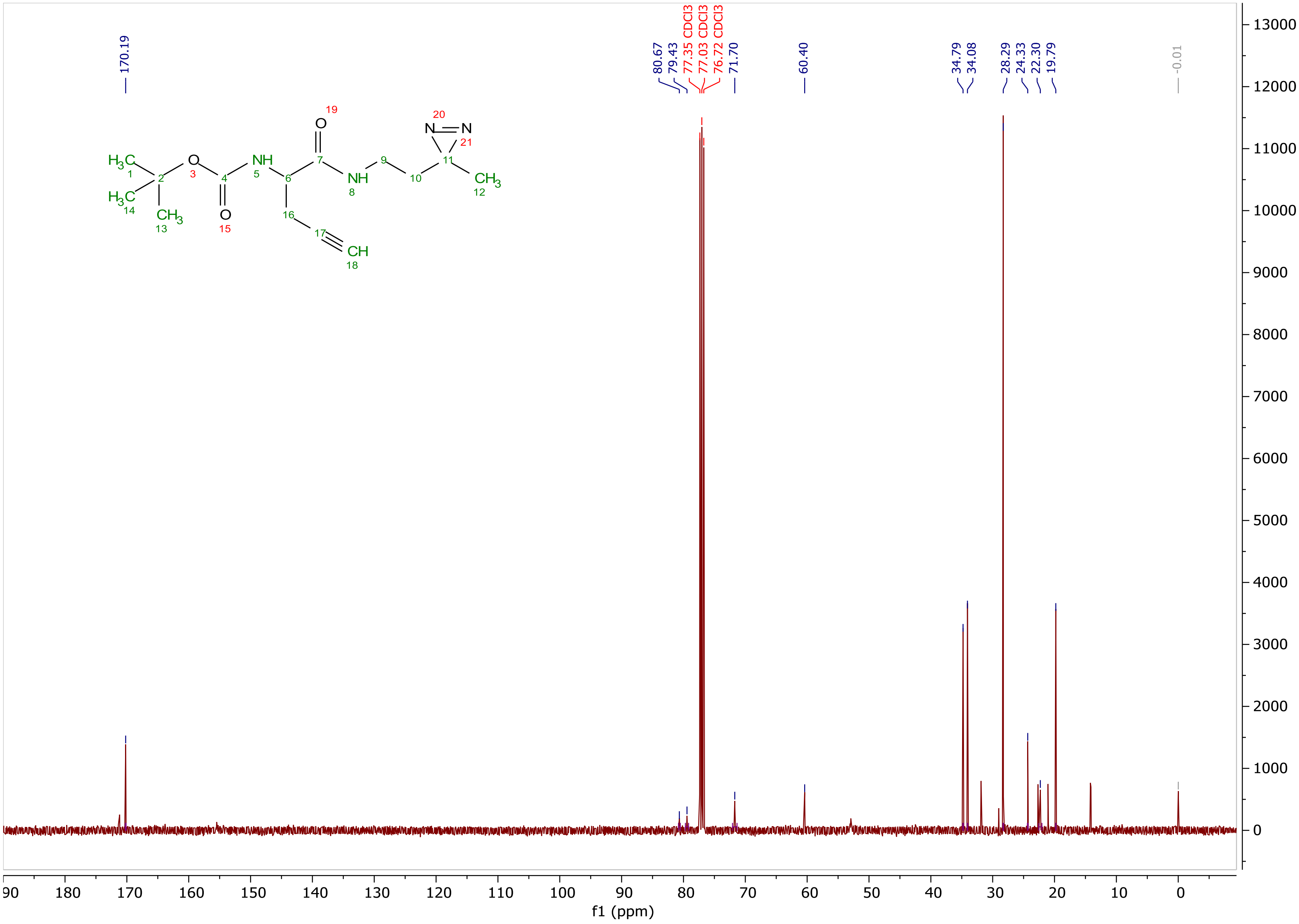
170.19
80.67
79.43
77.35 CDCl3
77.03 CDCl3
76.72 CDCl3
71.70
60.40
34.79
34.08
28.29
24.33
22.30
19.79
-0.01
f1 (ppm)

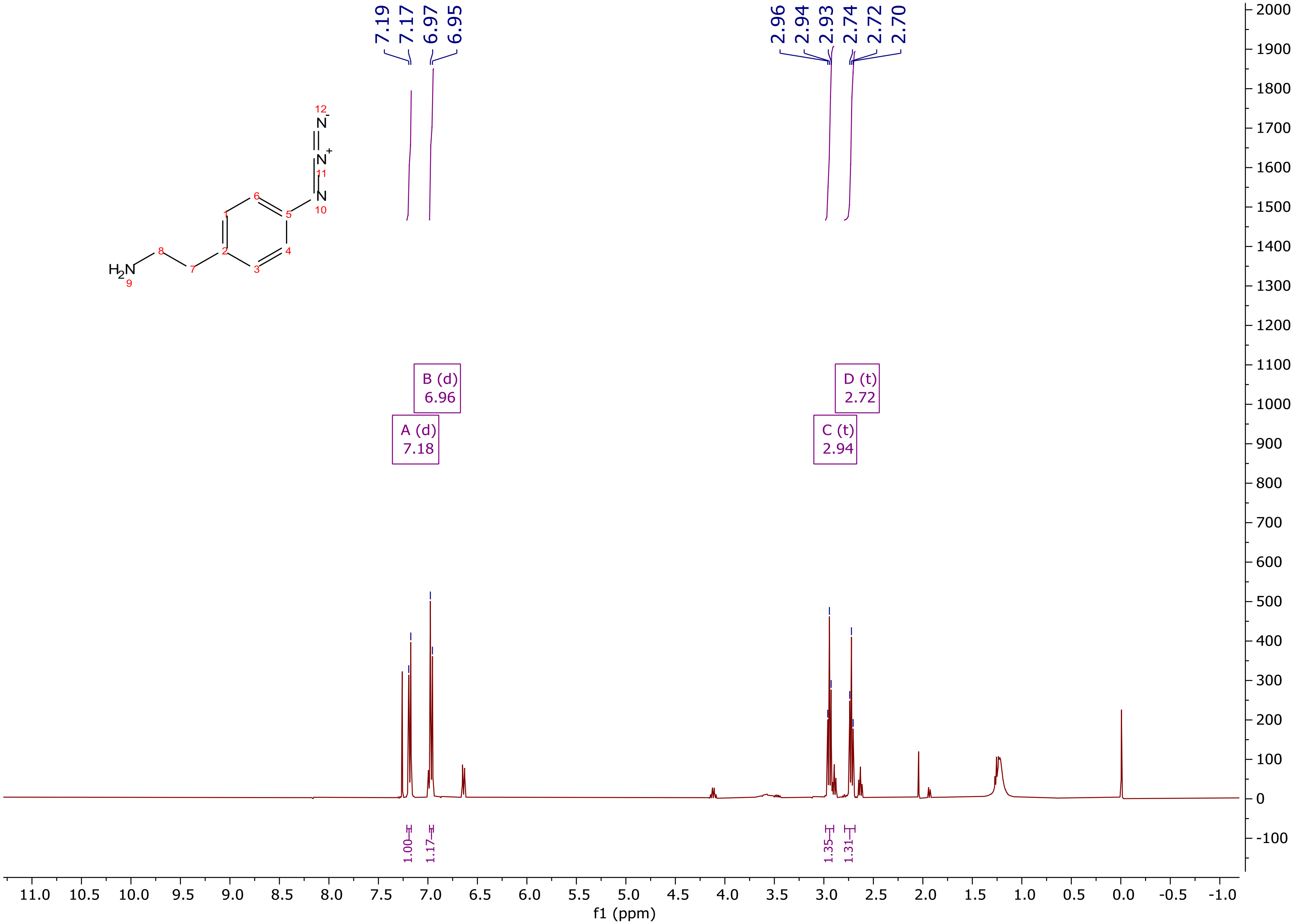
7.19
7.17
6.97
6.95
2.96
2.94
2.93
2.74
2.72
2.70
B (d)
6.96
A (d)
7.18
D (t)
2.72
C (t)
2.94
1.00
1.17
1.35
1.31
f1 (ppm)

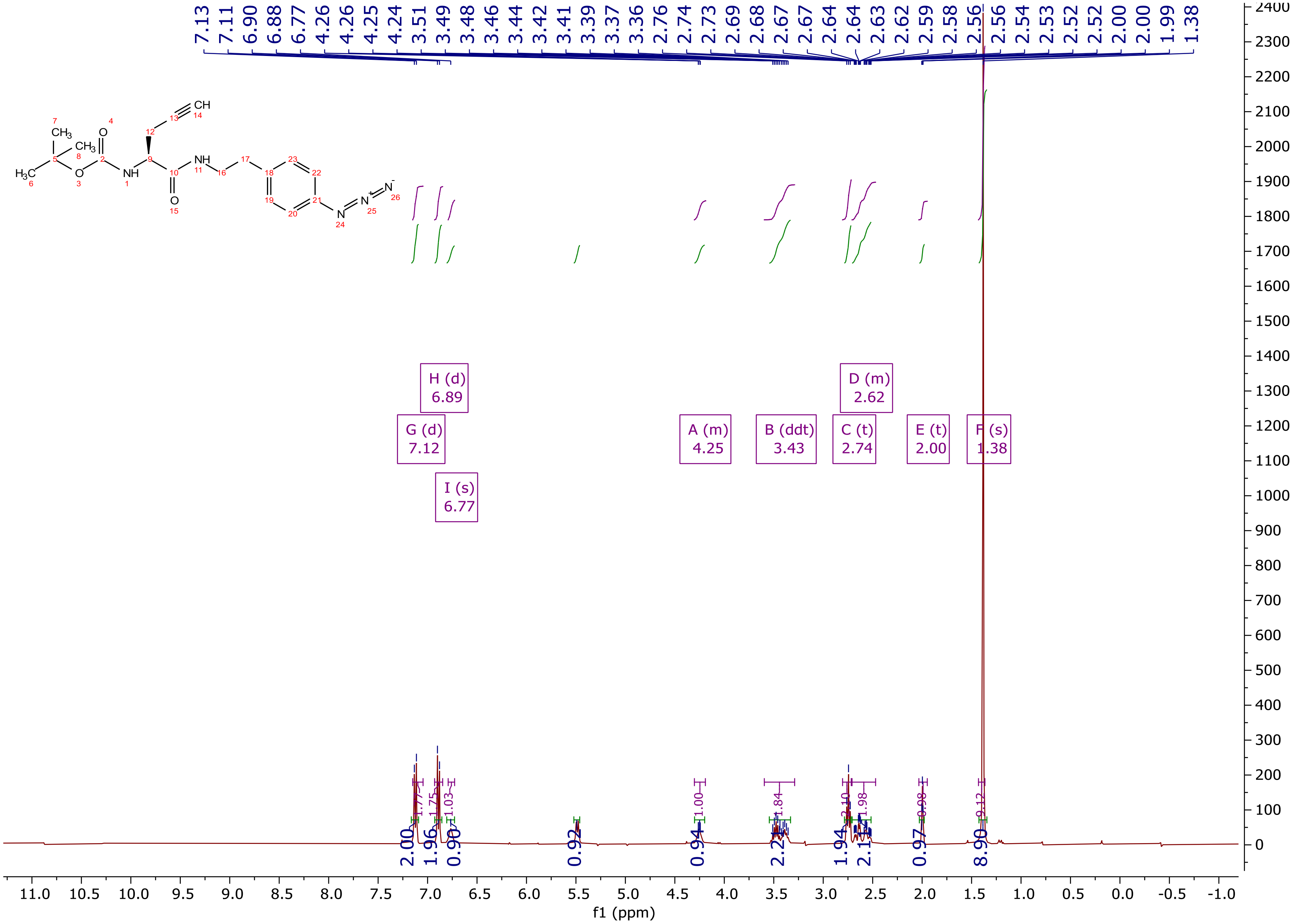
7.13 7.11 6.90 6.88 6.77 4.26 4.26 4.25 4.24 3.51 3.49 3.48 3.46 3.44 3.42 3.41 3.39 3.37 3.36 2.76 2.74 2.73 2.69 2.68 2.67 2.67 2.64 2.64 2.63 2.62 2.59 2.58 2.56 2.56 2.54 2.53 2.52 2.52 2.00 2.00 1.99 1.38
H (d) 6.89
D (m) 2.62
G (d) 7.12
A (m) 4.25
B (ddt) 3.43
C (t) 2.74
E (t) 2.00
F (s) 1.38
I (s) 6.77
2.00 1.96 0.90 0.92 0.94 2.22 1.94 2.11 0.97 8.90
f1 (ppm)

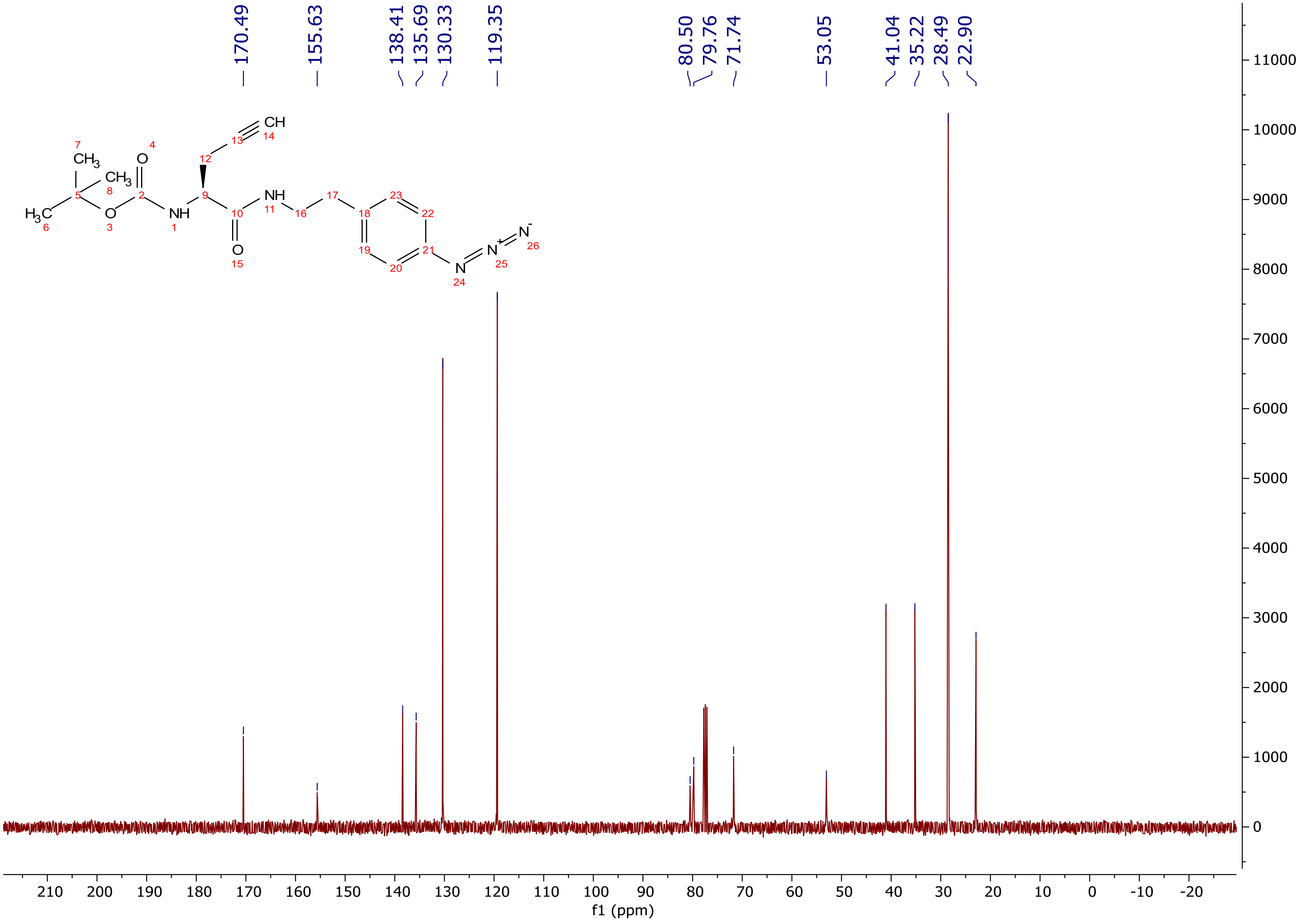

170.49
155.63
138.41
135.69
130.33
119.35
80.50
79.76
71.74
53.05
41.04
35.22
28.49
22.90
f1 (ppm)

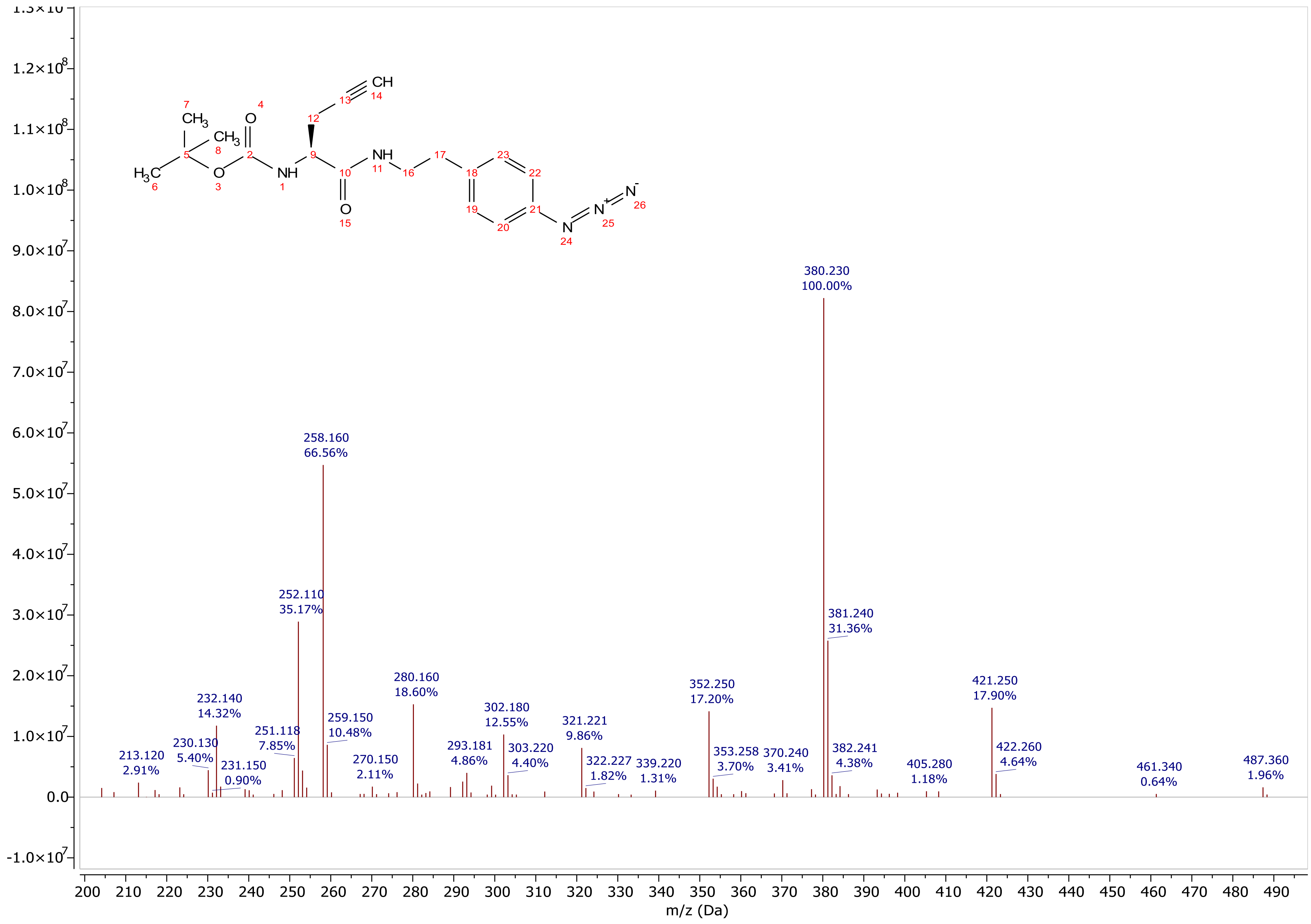

m/z (Da)
380.230
100.00%
258.160
66.56%
252.110
35.17%
381.240
31.36%
280.160
18.60%
421.250
17.90%
352.250
17.20%
232.140
14.32%
302.180
12.55%
259.150
10.48%
321.221
9.86%
251.118
7.85%
230.130
5.40%
293.181
4.86%
422.260
4.64%
303.220
4.40%
382.241
4.38%
353.258
3.70%
370.240
3.41%
213.120
2.91%
270.150
2.11%
487.360
1.96%
322.227
1.82%
339.220
1.31%
405.280
1.18%
231.150
0.90%
461.340
0.64%

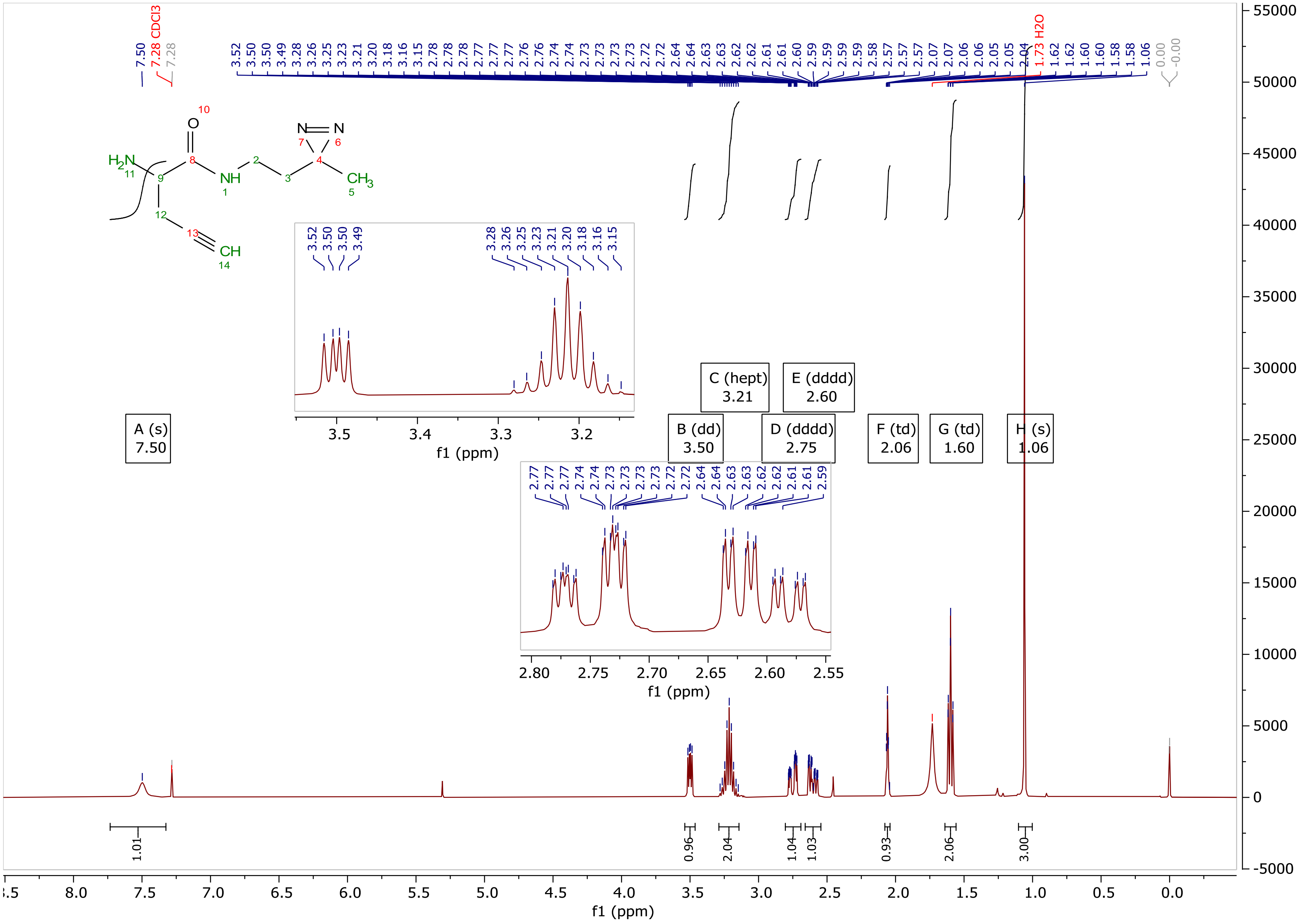

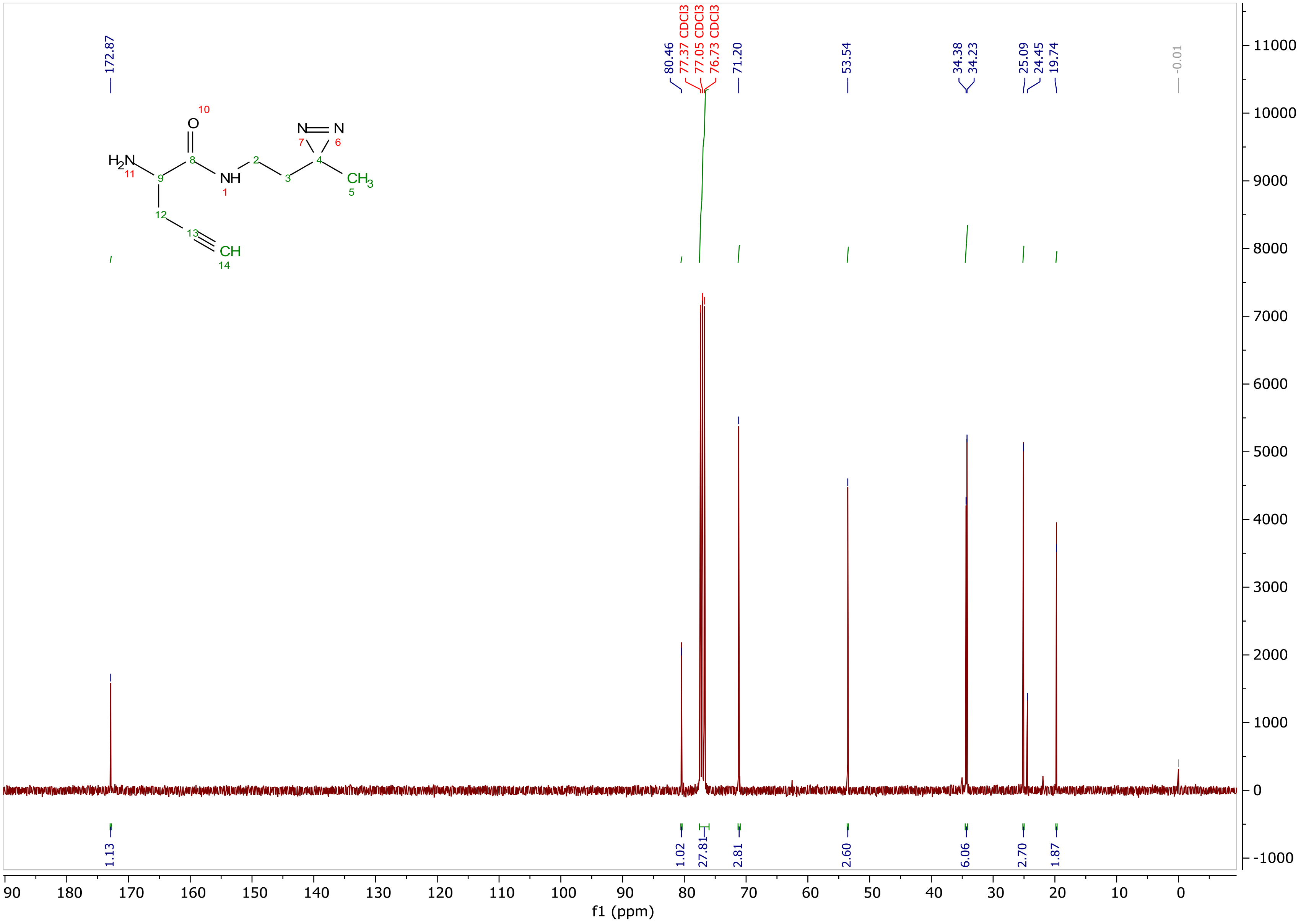

172.87
80.46
77.37 CDCl3
77.05 CDCl3
76.73 CDCl3
71.20
53.54
34.38
34.23
25.09
24.45
19.74
-0.01
1.13
1.02
27.81
2.81
2.60
6.06
2.70
1.87
f1 (ppm)

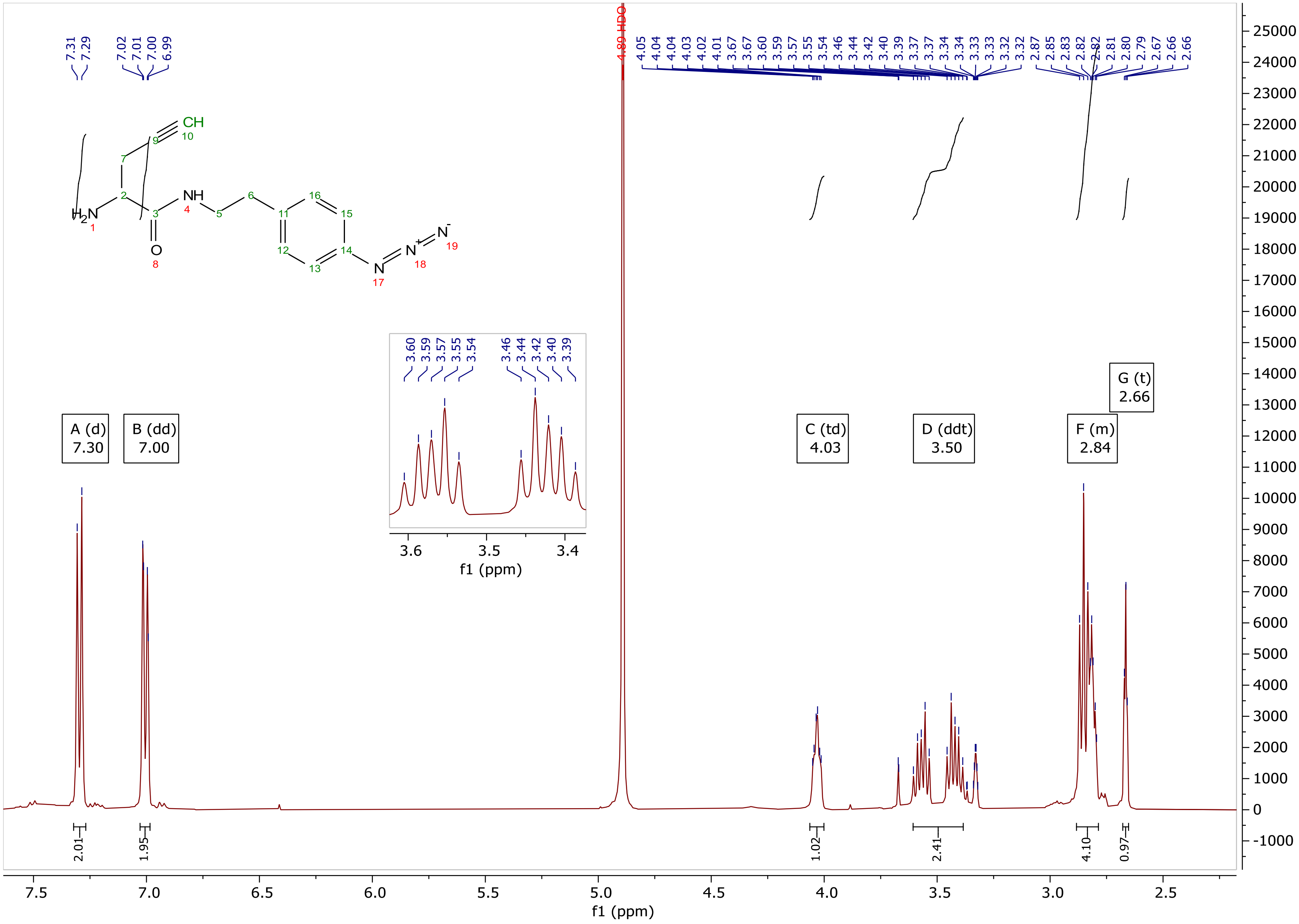

A (d)
7.30
B (dd)
7.00
C (td)
4.03
D (ddt)
3.50
F (m)
2.84
G (t)
2.66
4.89 HDO
f1 (ppm)

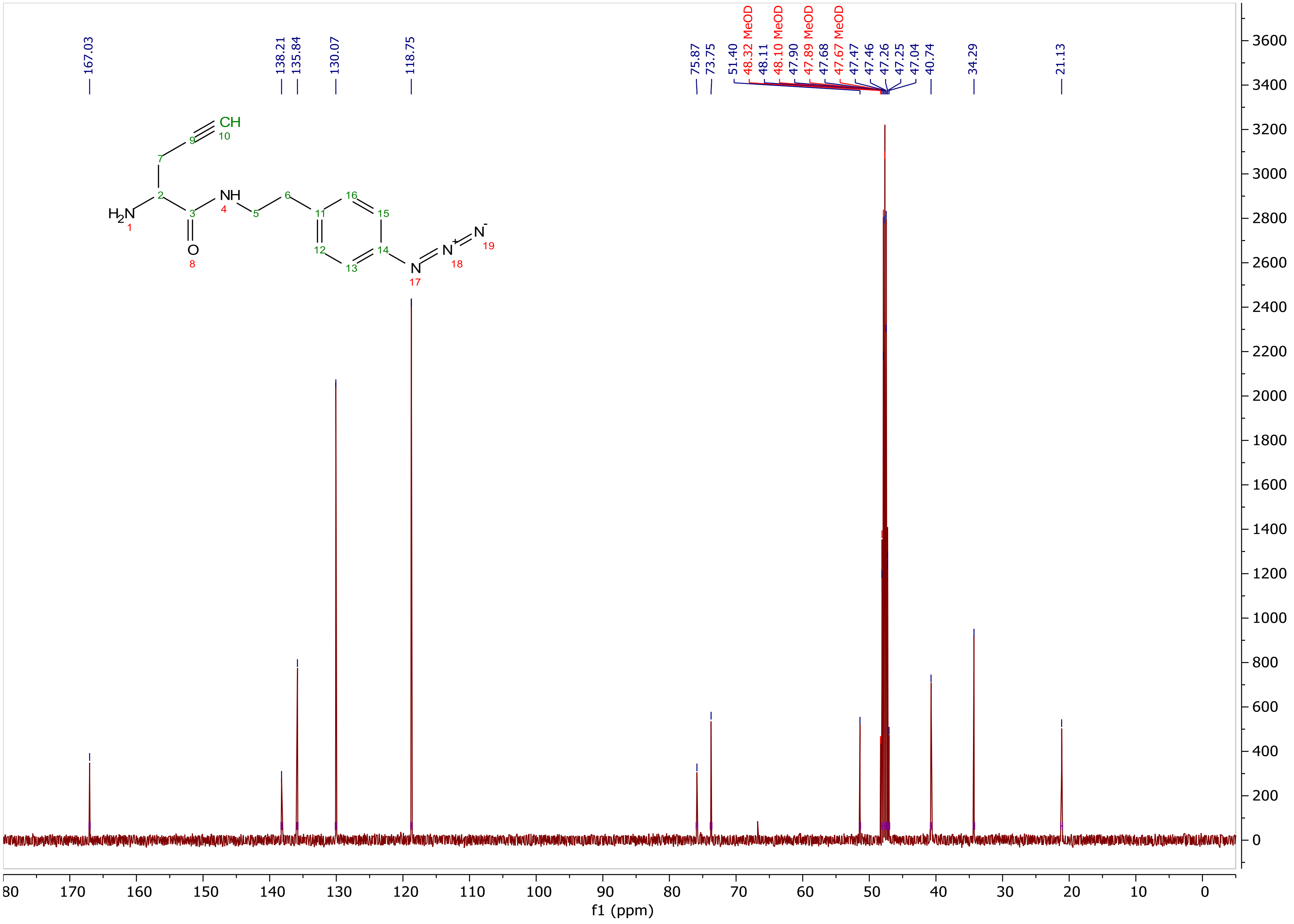
167.03
138.21
135.84
130.07
118.75
75.87
73.75
51.40
48.32 MeOD
48.11
48.10 MeOD
47.90
47.89 MeOD
47.68
47.67 MeOD
47.47
47.46
47.26
47.25
47.04
40.74
34.29
21.13
f1 (ppm)

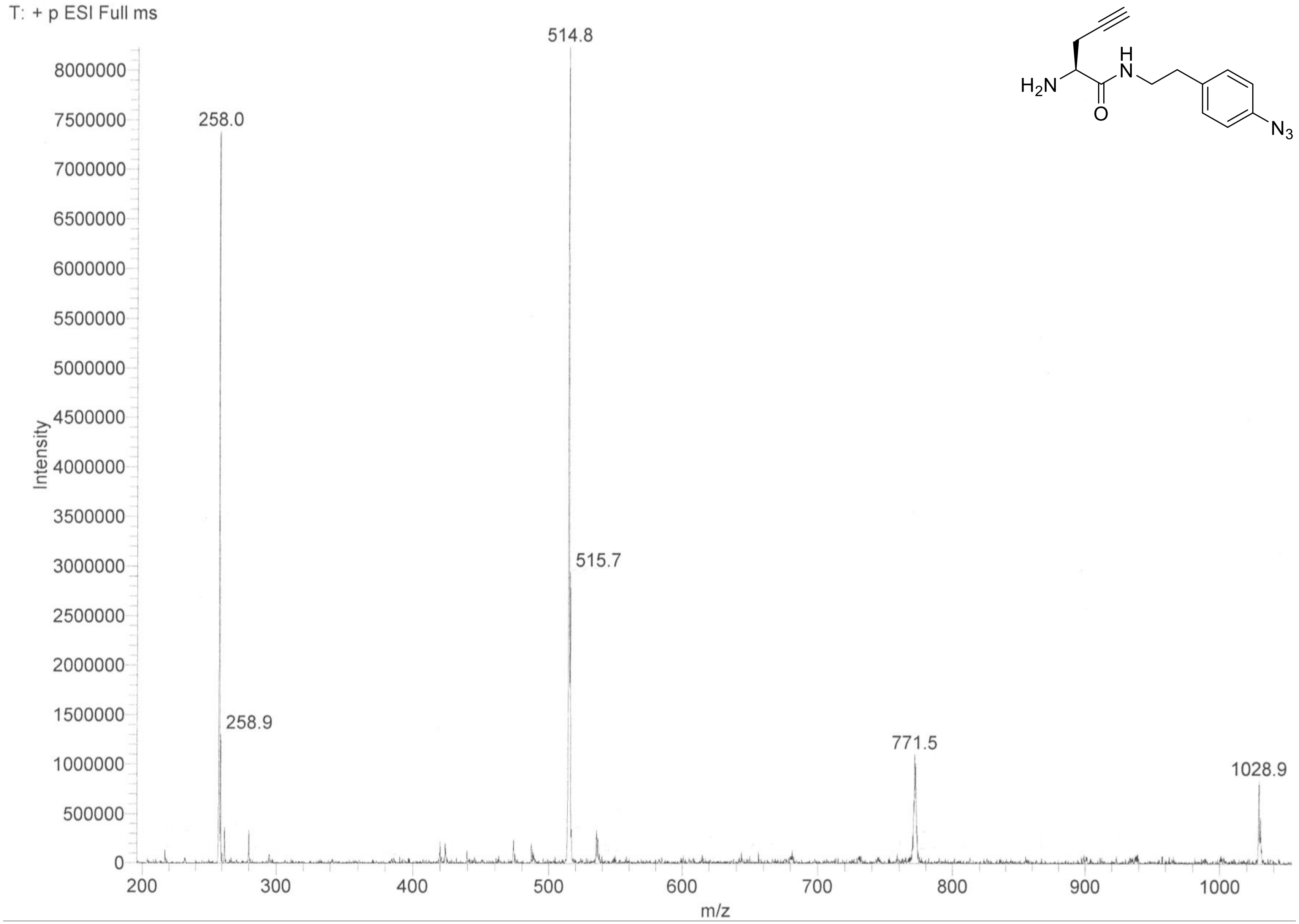
T: + p ESI Full ms
514.8
258.0
515.7
258.9
771.5
1028.9
Intensity
m/z
H2N
H
N
O
N3

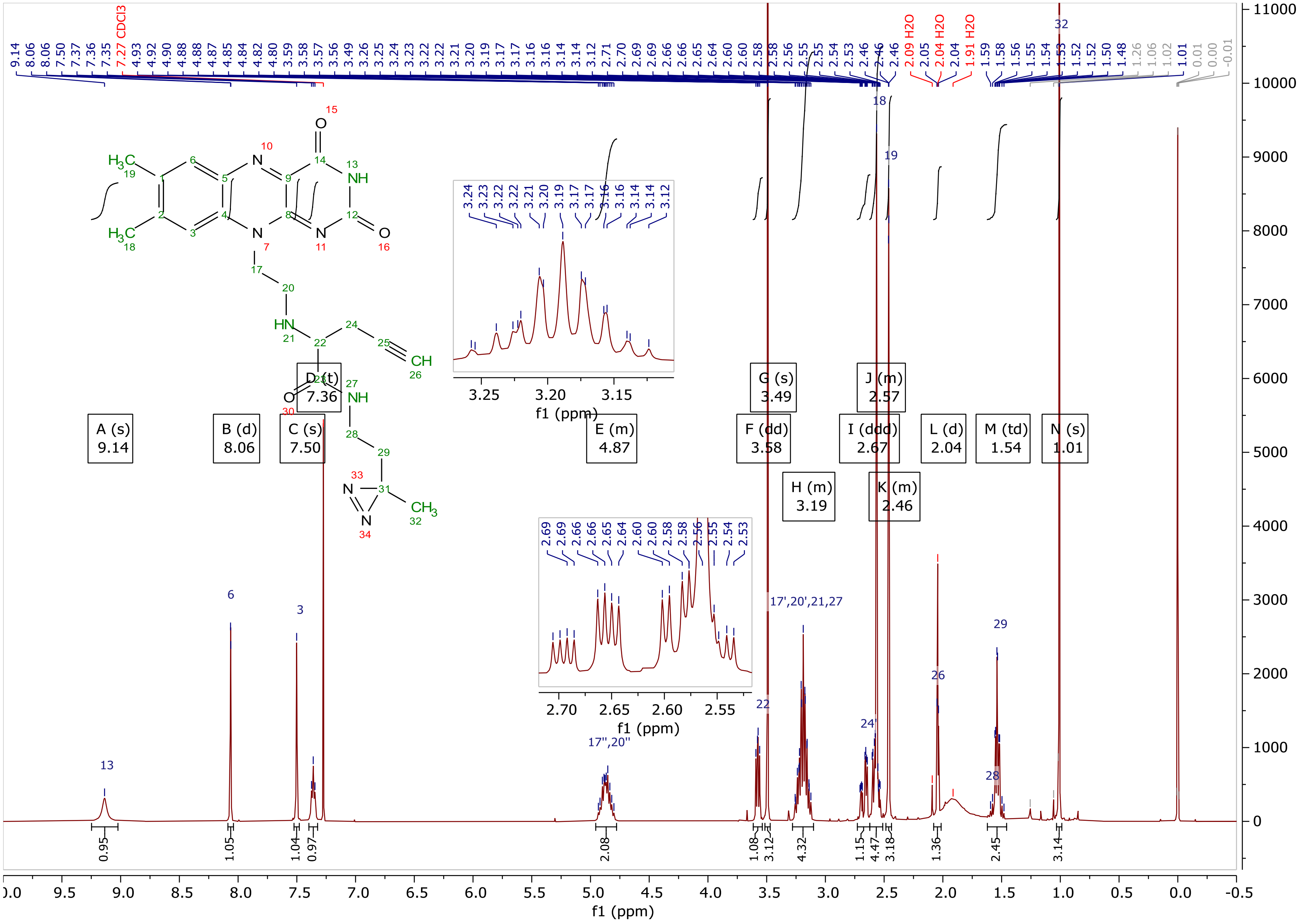

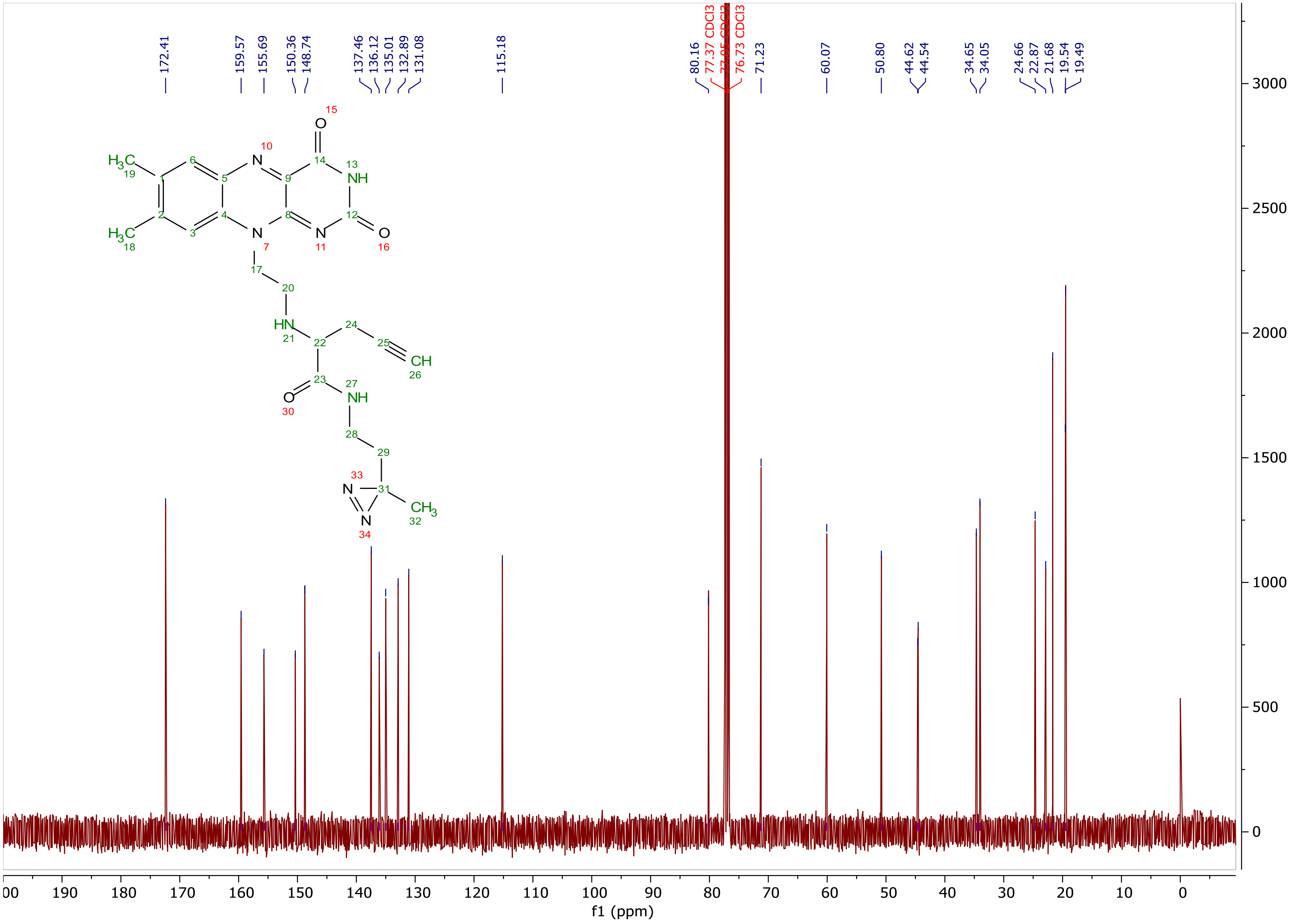

172.41
159.57
155.69
150.36
148.74
137.46
136.12
135.01
132.89
131.08
115.18
80.16
77.37 CDCl3
76.73 CDCl3
71.23
60.07
50.80
44.62
44.54
34.65
34.05
24.66
22.87
21.68
19.54
19.49
f1 (ppm)

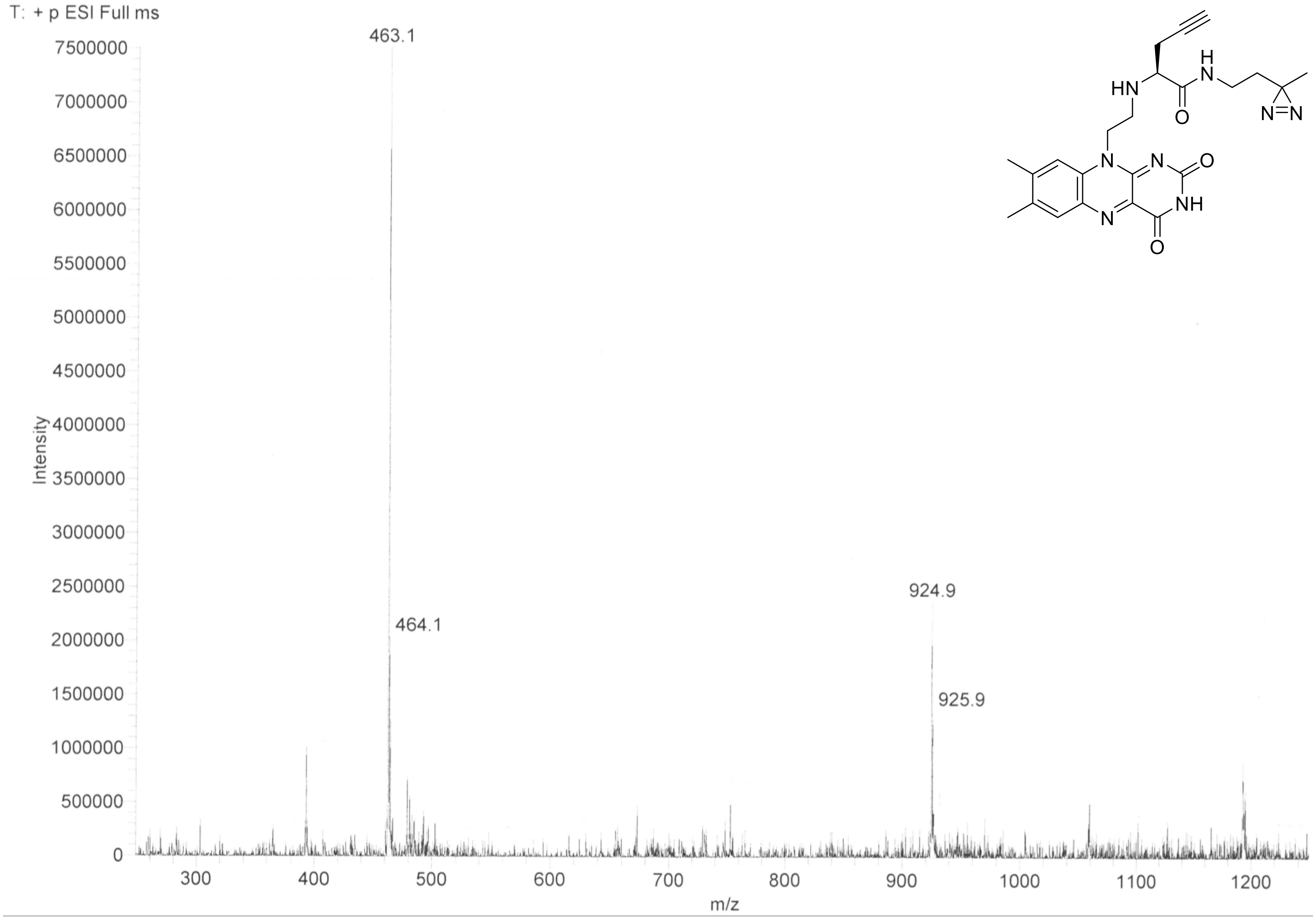

T: + p ESI Full ms
463.1
464.1
924.9
925.9
Intensity
m/z
HN
H
N
O
N
N
N
N
O
NH
O
N=N

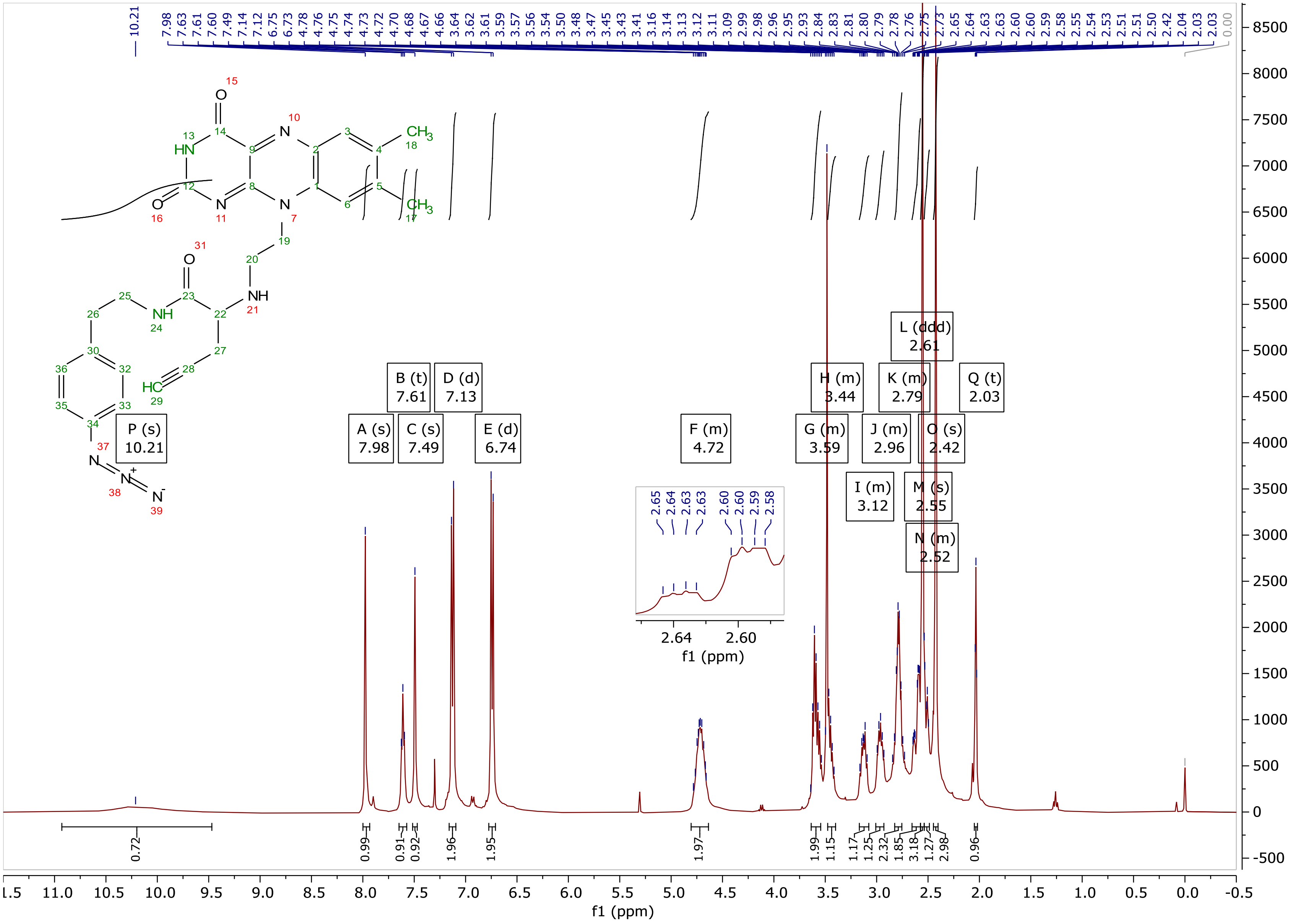

A (s) 7.98
B (t) 7.61
C (s) 7.49
D (d) 7.13
E (d) 6.74
F (m) 4.72
G (m) 3.59
H (m) 3.44
I (m) 3.12
J (m) 2.96
K (m) 2.79
L (ddd) 2.61
M (s) 2.55
N (m) 2.52
O (s) 2.42
P (s) 10.21
Q (t) 2.03
f1 (ppm)

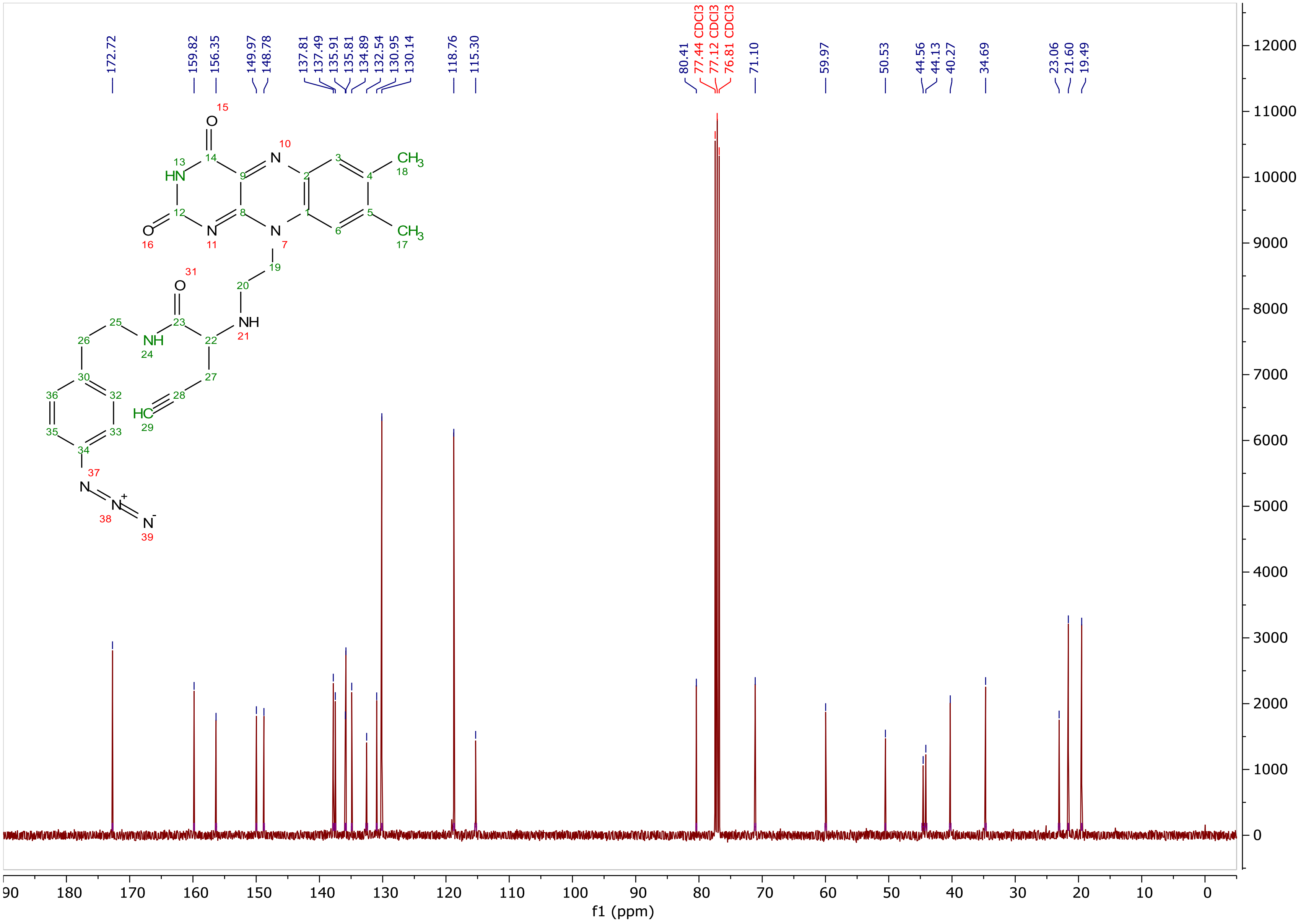
172.72
159.82
156.35
149.97
148.78
137.81
137.49
135.91
135.81
134.89
132.54
130.95
130.14
118.76
115.30
80.41
77.44 CDCl3
77.12 CDCl3
76.81 CDCl3
71.10
59.97
50.53
44.56
44.13
40.27
34.69
23.06
21.60
19.49
f1 (ppm)

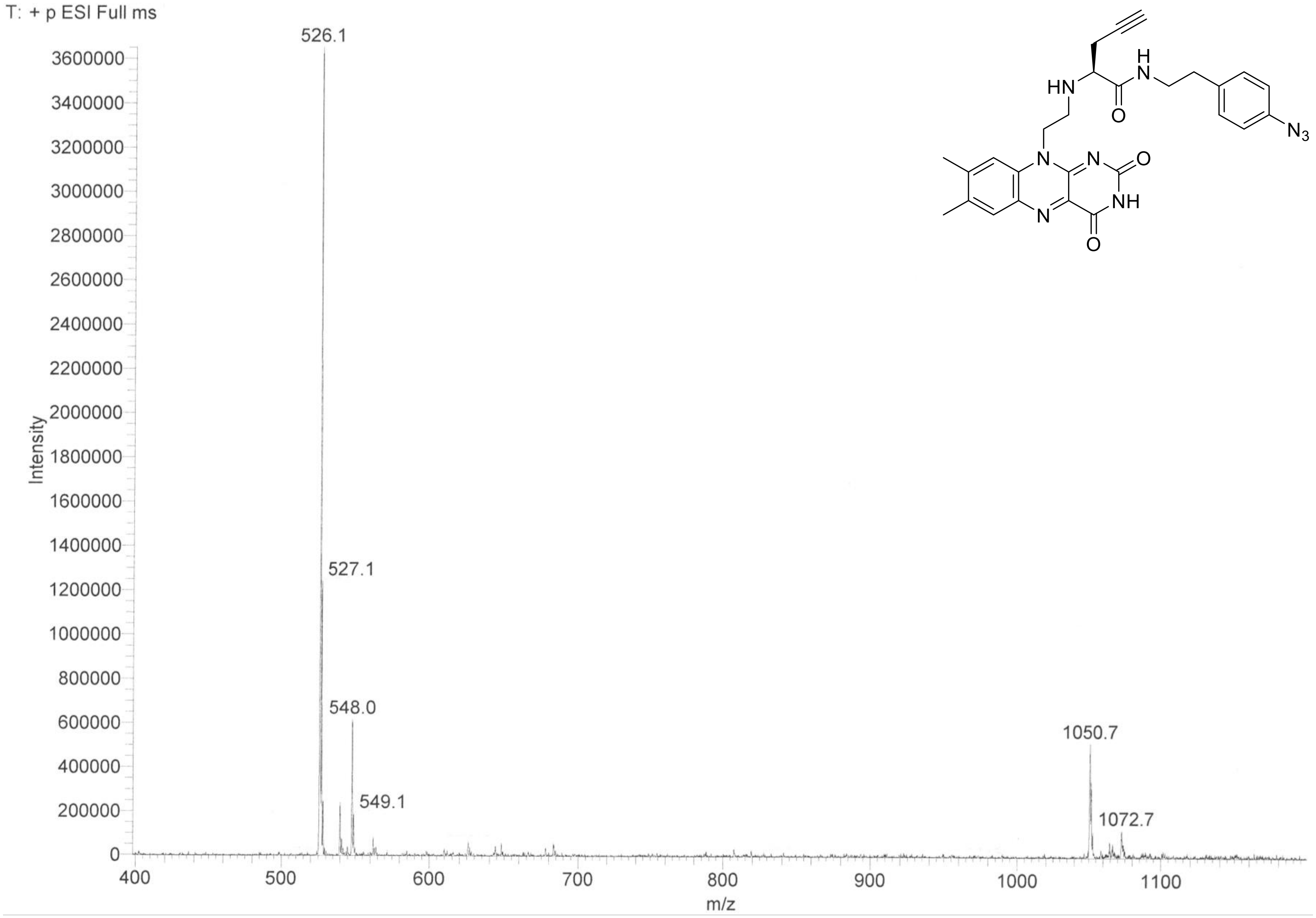

T: + p ESI Full ms
526.1
527.1
548.0
549.1
1050.7
1072.7
Intensity
m/z
HN
H
N
O
N
N
N
O
NH
O
N
N_3

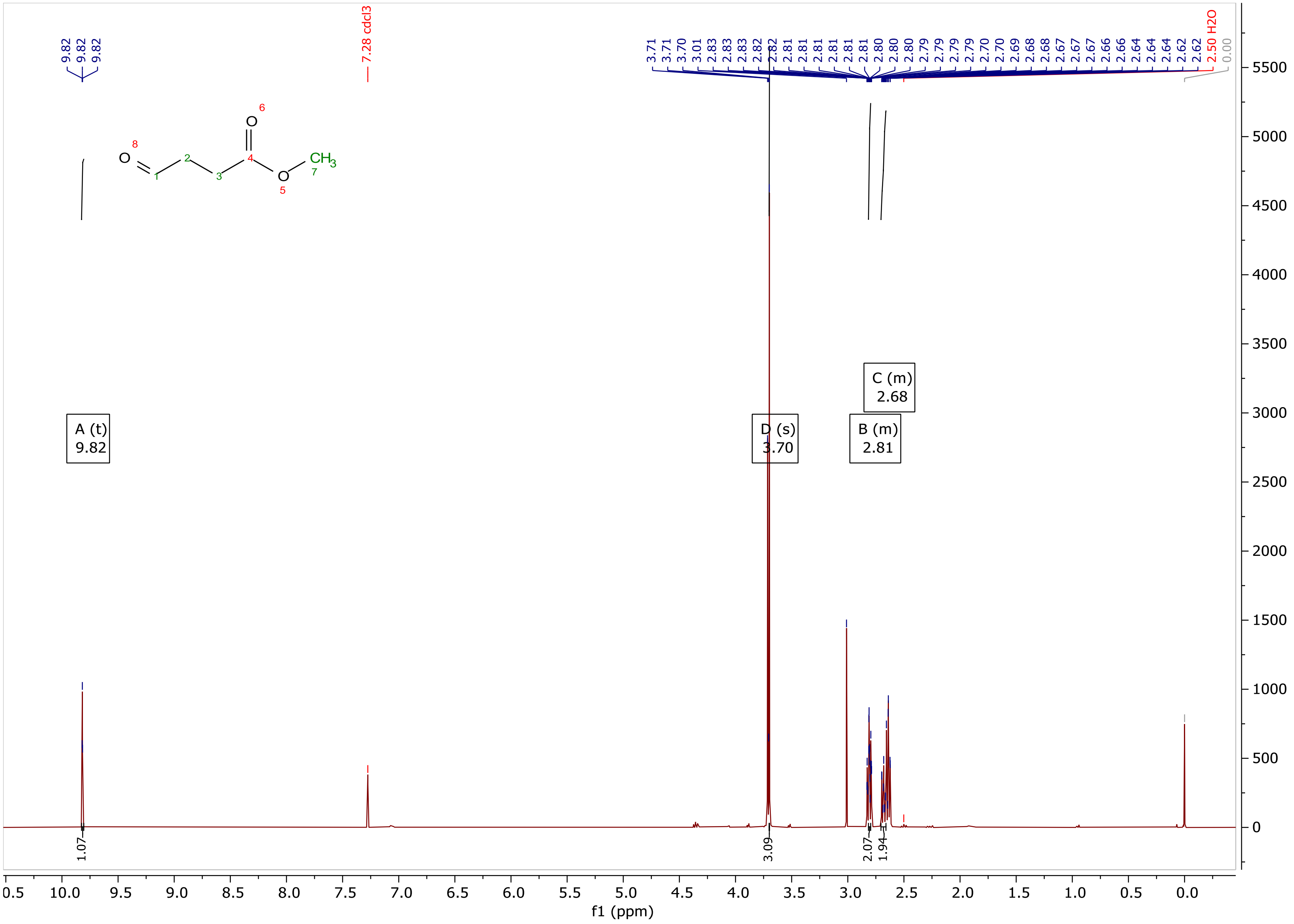

9.82
9.82
9.82
7.28 cdcl3
3.71
3.71
3.70
3.01
2.83
2.83
2.83
2.82
2.82
2.81
2.81
2.81
2.81
2.81
2.81
2.80
2.80
2.80
2.79
2.79
2.79
2.79
2.70
2.70
2.69
2.68
2.68
2.67
2.67
2.67
2.66
2.66
2.64
2.64
2.64
2.62
2.62
2.50 H2O
0.00
O
8
1
2
3
4
O
6
O
5
CH3
7
A (t)
9.82
D (s)
3.70
B (m)
2.81
C (m)
2.68
1.07
3.09
2.07
1.94
f1 (ppm)

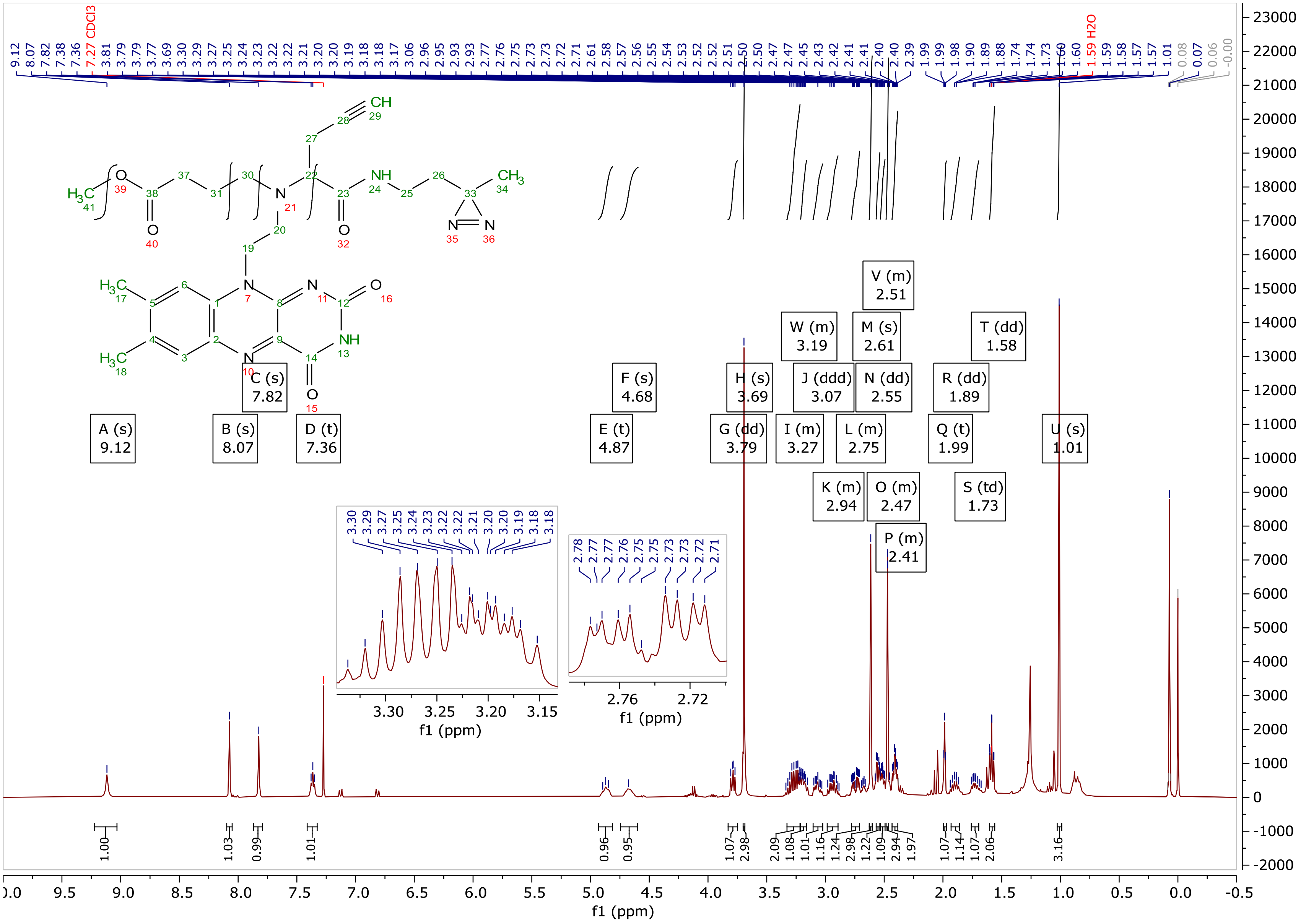

A (s) 9.12
B (s) 8.07
C (s) 7.82
D (t) 7.36
E (t) 4.87
F (s) 4.68
G (dd) 3.79
H (s) 3.69
I (m) 3.27
J (ddd) 3.07
K (m) 2.94
L (m) 2.75
M (s) 2.61
N (dd) 2.55
O (m) 2.47
P (m) 2.41
Q (t) 1.99
R (dd) 1.89
S (td) 1.73
T (dd) 1.58
U (s) 1.01
V (m) 2.51
W (m) 3.19
7.27 CDCl3
1.59 H2O
f1 (ppm)

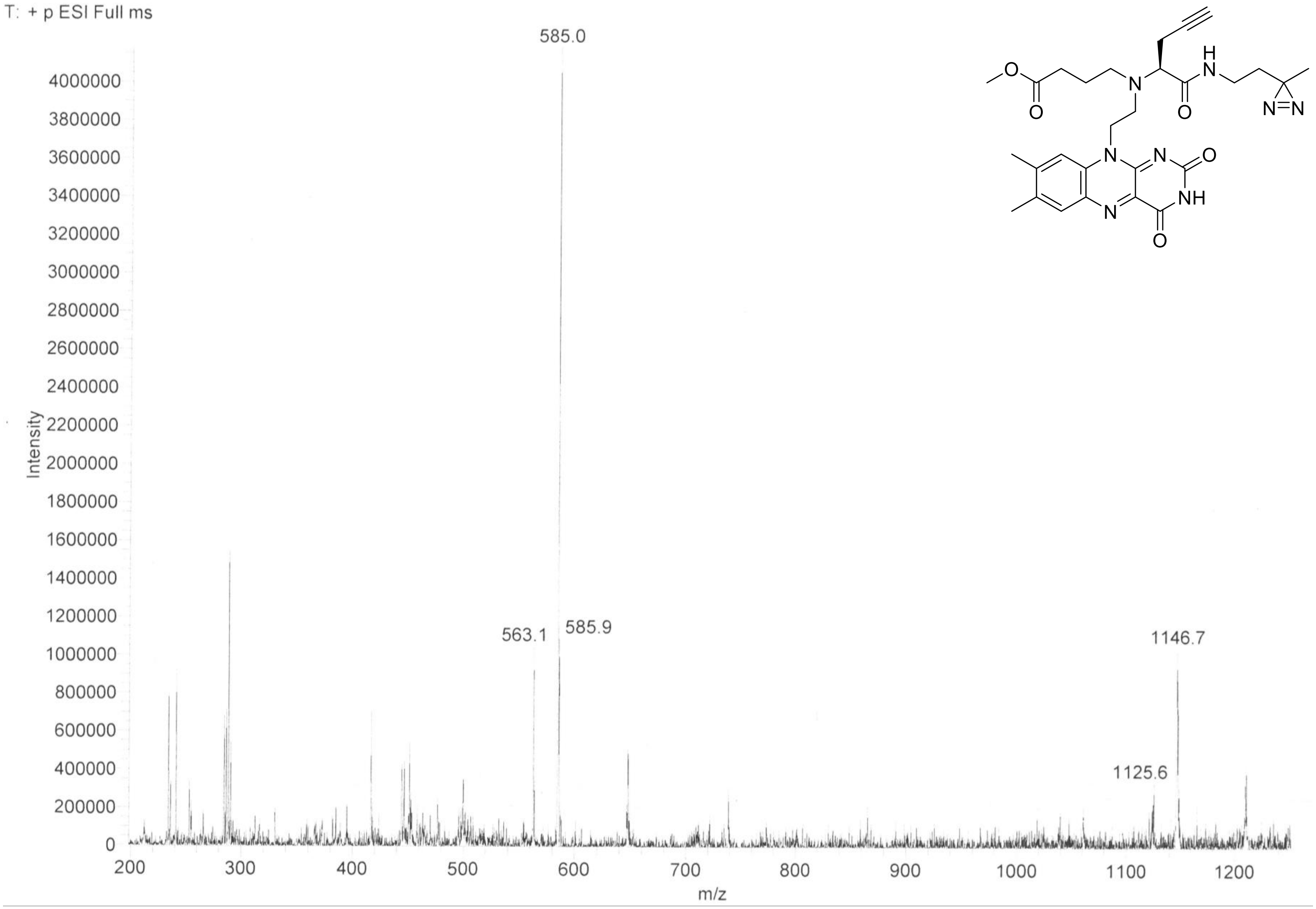
T: + p ESI Full ms
585.0
563.1
585.9
1146.7
1125.6
Intensity
m/z
O
N
H
N
O
O
N=N
N
N
O
NH
N
O

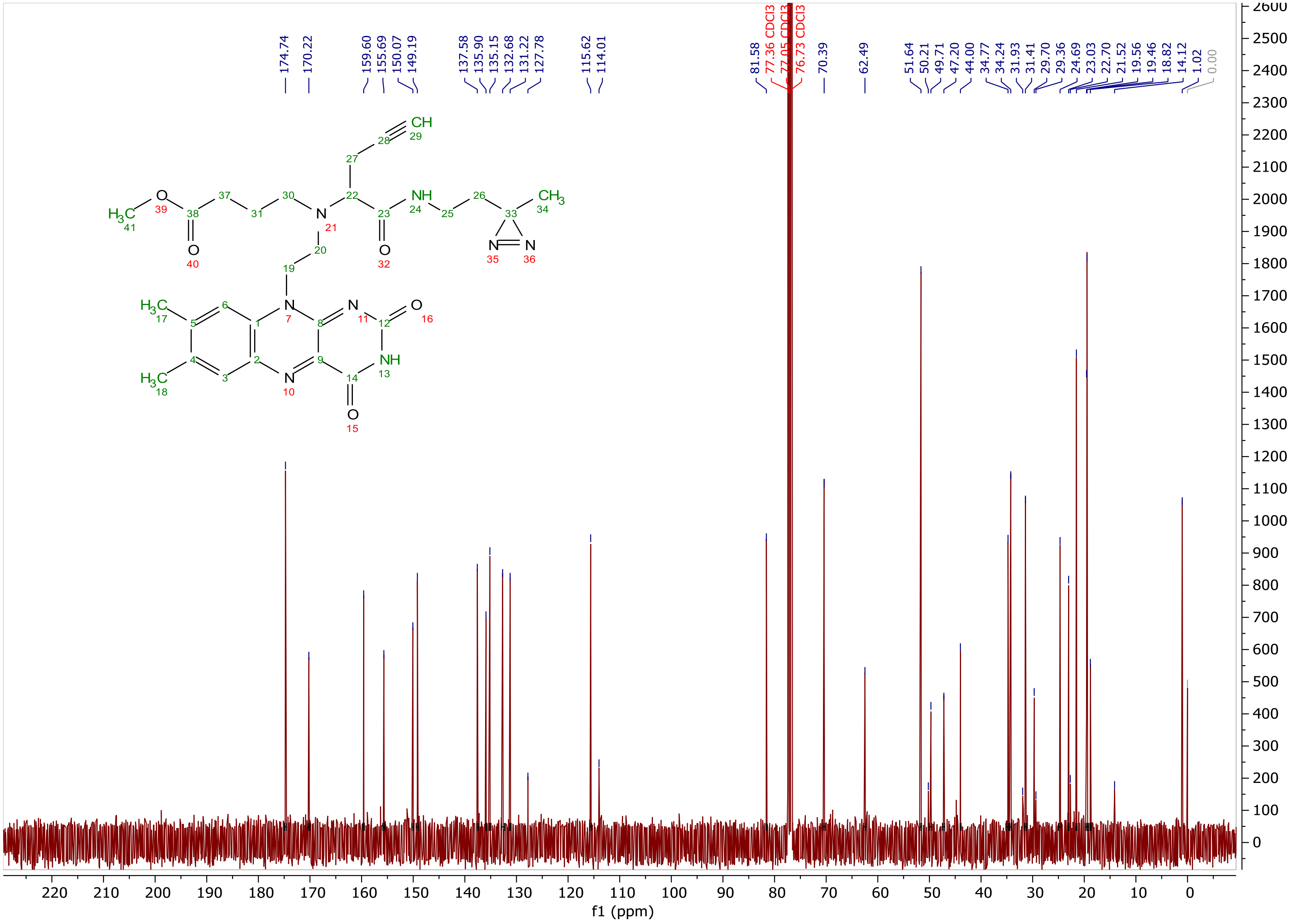
174.74
170.22
159.60
155.69
150.07
149.19
137.58
135.90
135.15
132.68
131.22
127.78
115.62
114.01
81.58
77.36 CDCl3
77.05 CDCl3
76.73 CDCl3
70.39
62.49
51.64
50.21
49.71
47.20
44.00
34.77
34.24
31.93
31.41
29.70
29.36
24.69
23.03
22.70
21.52
19.56
19.46
18.82
14.12
1.02
0.00
f1 (ppm)

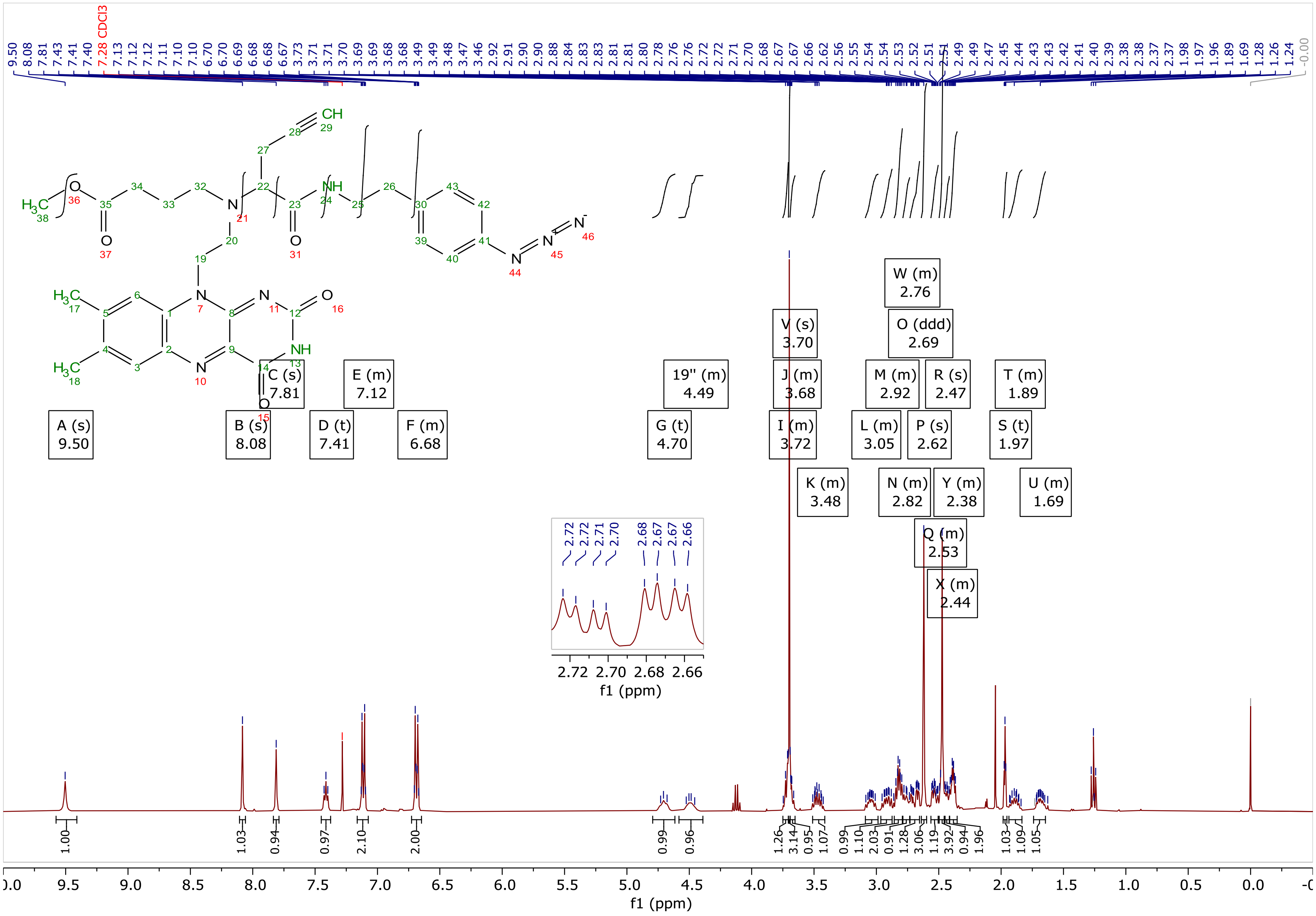
A (s) 9.50
B (s) 8.08
C (s) 7.81
D (t) 7.41
E (m) 7.12
F (m) 6.68
G (t) 4.70
19'' (m) 4.49
I (m) 3.72
J (m) 3.68
K (m) 3.48
V (s) 3.70
L (m) 3.05
M (m) 2.92
N (m) 2.82
W (m) 2.76
O (ddd) 2.69
P (s) 2.62
Q (m) 2.53
R (s) 2.47
X (m) 2.44
Y (m) 2.38
S (t) 1.97
T (m) 1.89
U (m) 1.69
CDCl3
f1 (ppm)

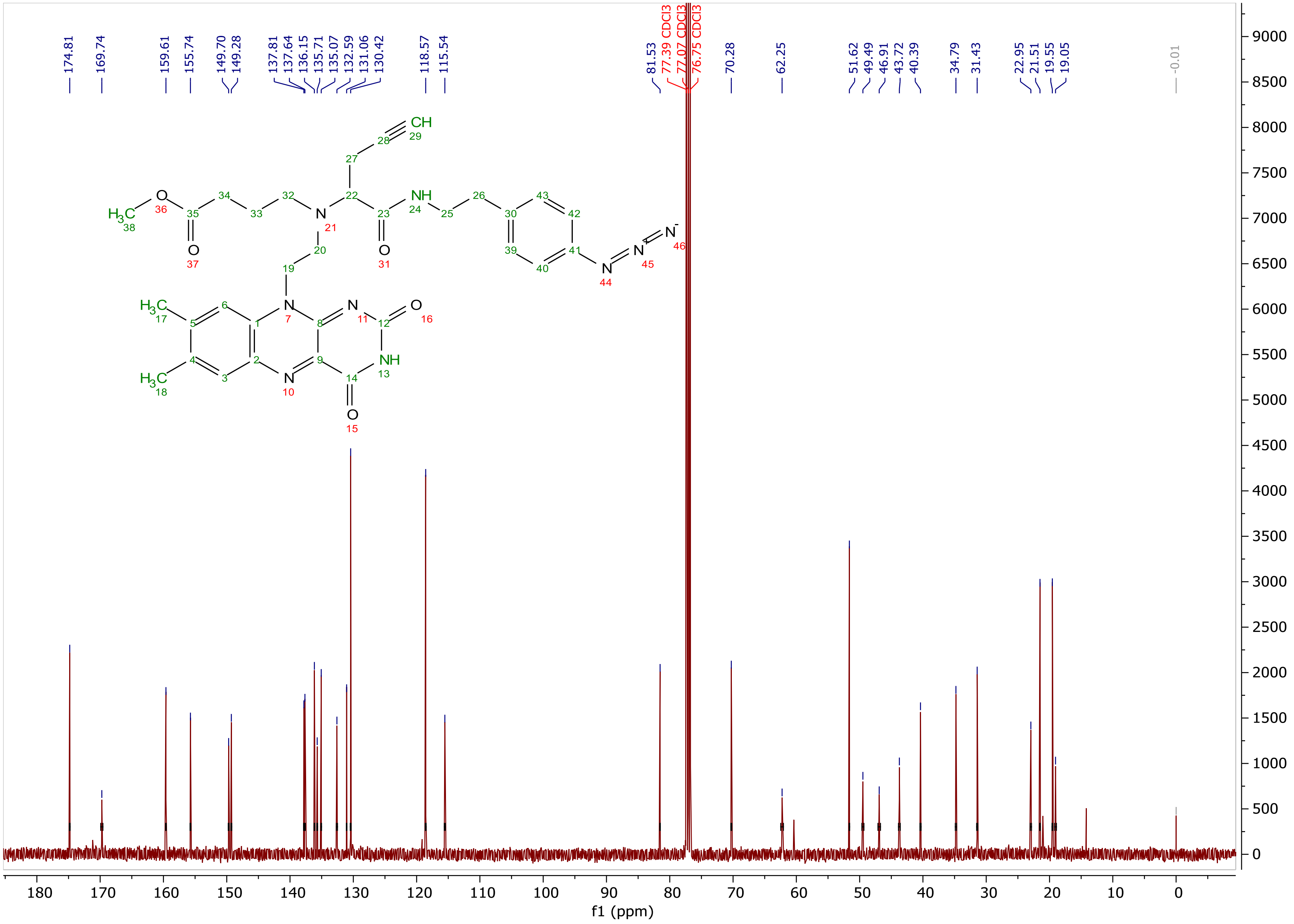

174.81
169.74
159.61
155.74
149.70
149.28
137.81
137.64
136.15
135.71
135.07
132.59
131.06
130.42
118.57
115.54
81.53
77.39 CDCl3
77.07 CDCl3
76.75 CDCl3
70.28
62.25
51.62
49.49
46.91
43.72
40.39
34.79
31.43
22.95
21.51
19.55
19.05
-0.01
f1 (ppm)

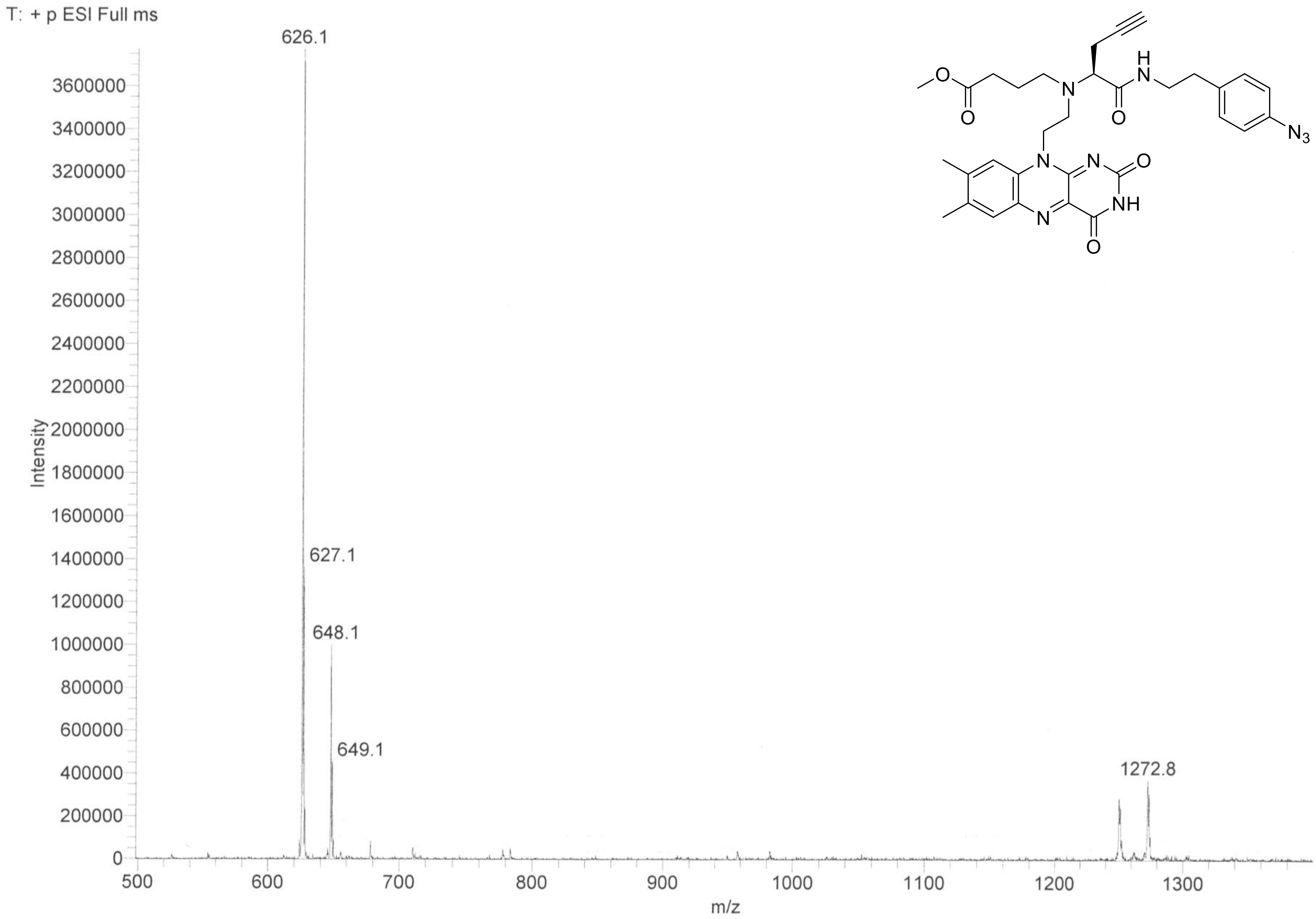
T: + p ESI Full ms
626.1
627.1
648.1
649.1
1272.8
Intensity
m/z
3600000
3400000
3200000
3000000
2800000
2600000
2400000
2200000
2000000
1800000
1600000
1400000
1200000
1000000
800000
600000
400000
200000
0
500
600
700
800
900
1000
1100
1200
1300
O
N
H
N
O
O
N
N
N
O
NH
O
N3

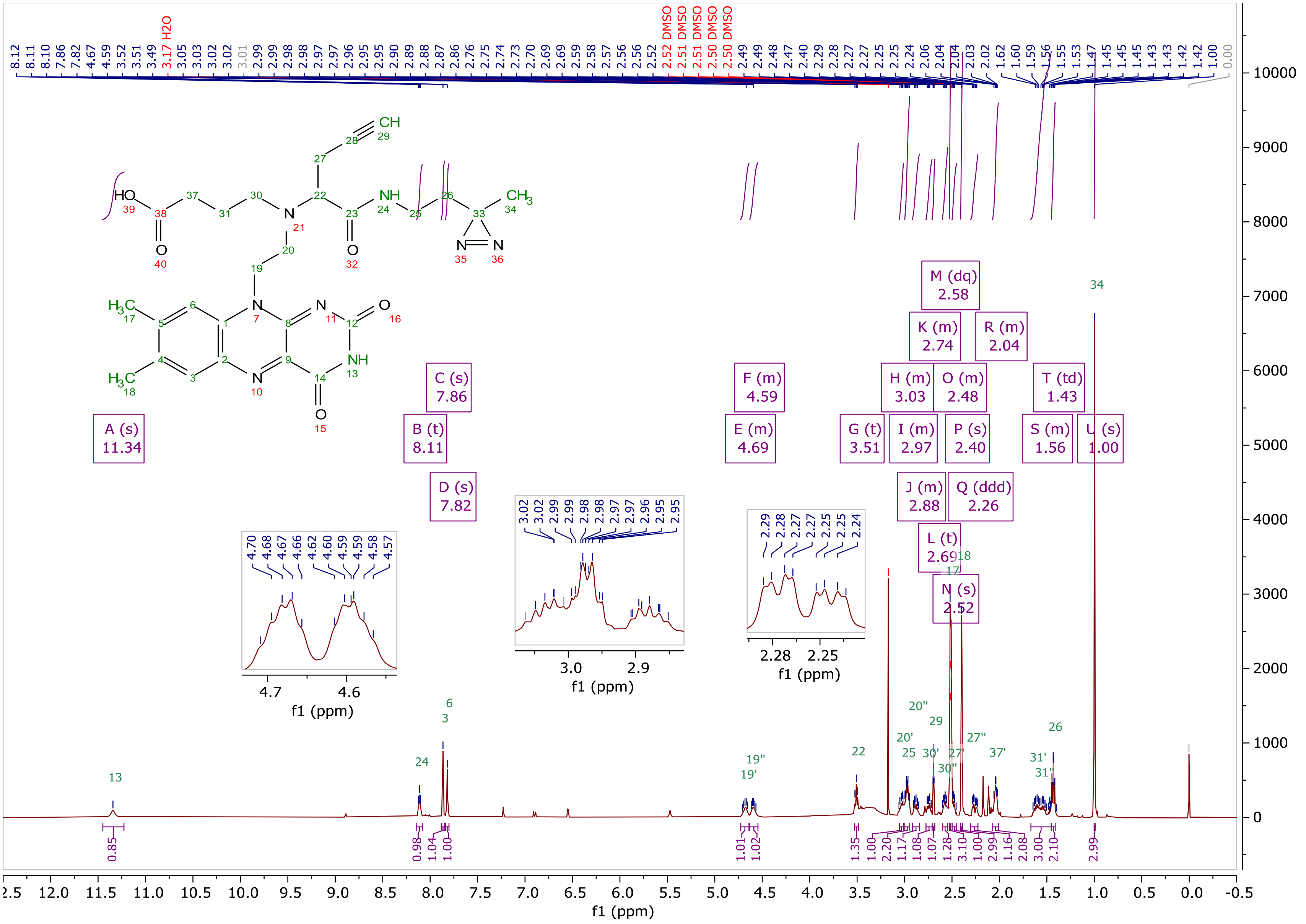
A (s)
11.34
B (t)
8.11
C (s)
7.86
D (s)
7.82
E (m)
4.69
F (m)
4.59
G (t)
3.51
H (m)
3.03
I (m)
2.97
J (m)
2.88
K (m)
2.74
L (t)
2.69
M (dq)
2.58
N (s)
2.52
O (m)
2.48
P (s)
2.40
Q (ddd)
2.26
R (m)
2.04
S (m)
1.56
T (td)
1.43
U (s)
1.00
3.17 H2O
2.52 DMSO
2.51 DMSO
2.51 DMSO
2.50 DMSO
2.50 DMSO
f1 (ppm)

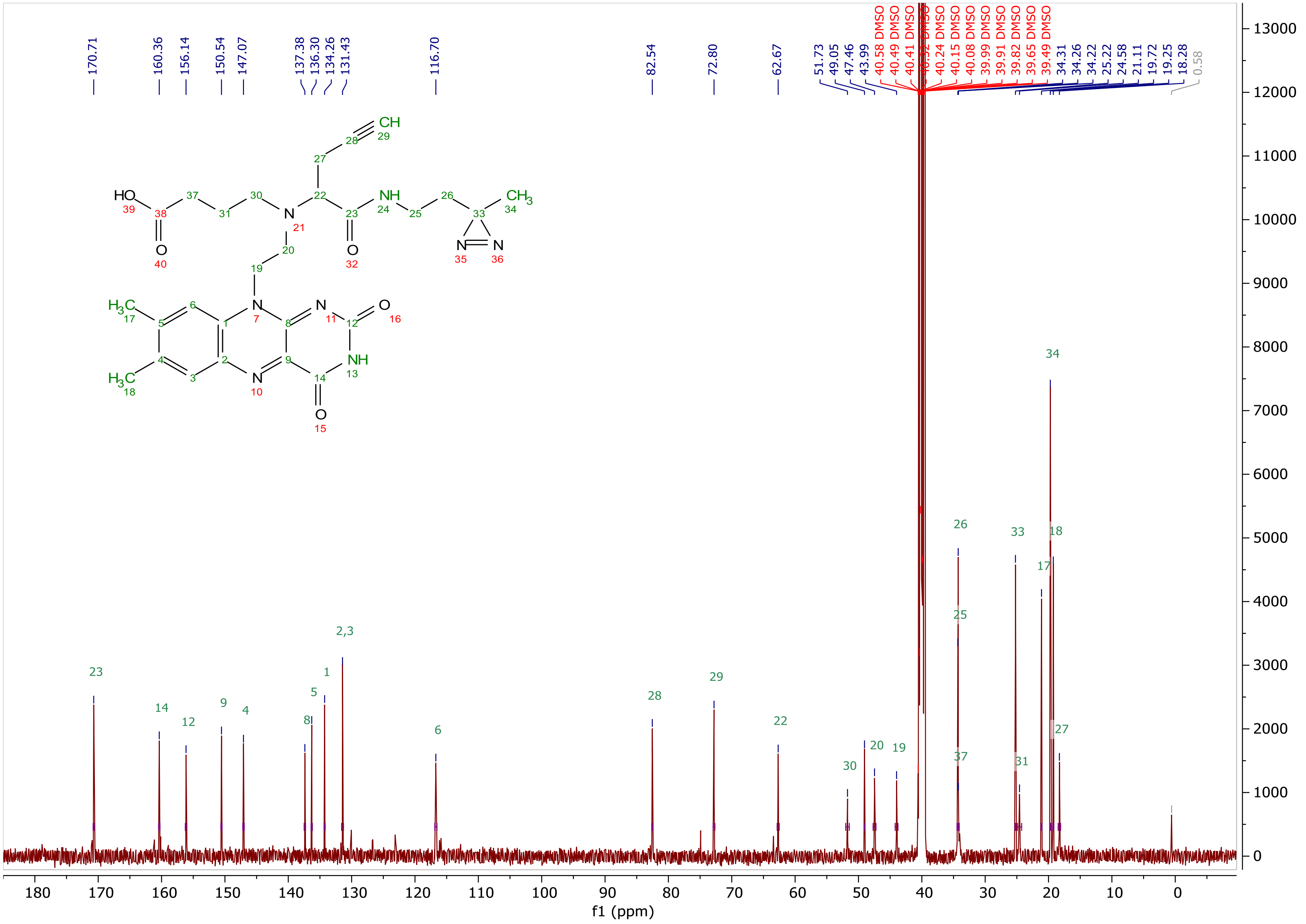

170.71
160.36
156.14
150.54
147.07
137.38
136.30
134.26
131.43
116.70
82.54
72.80
62.67
51.73
49.05
47.46
43.99
40.58 DMSO
40.49 DMSO
40.41 DMSO
40.24 DMSO
40.15 DMSO
40.08 DMSO
39.99 DMSO
39.91 DMSO
39.82 DMSO
39.65 DMSO
39.49 DMSO
34.31
34.26
34.22
25.22
24.58
21.11
19.72
19.25
18.28
0.58
HO
O
N
NH
CH
CH3
H3C
NH
O
f1 (ppm)

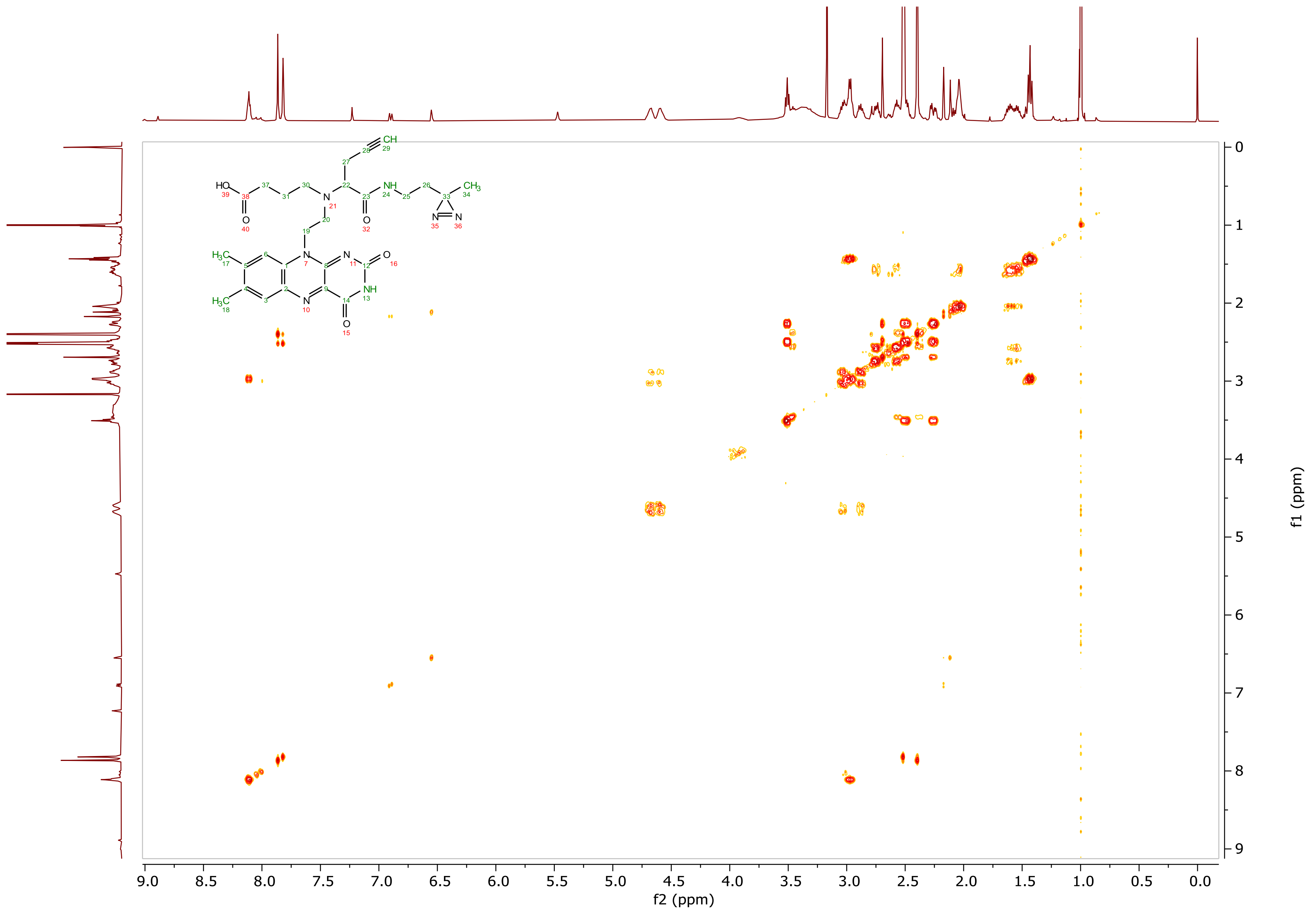
f2 (ppm)
f1 (ppm)

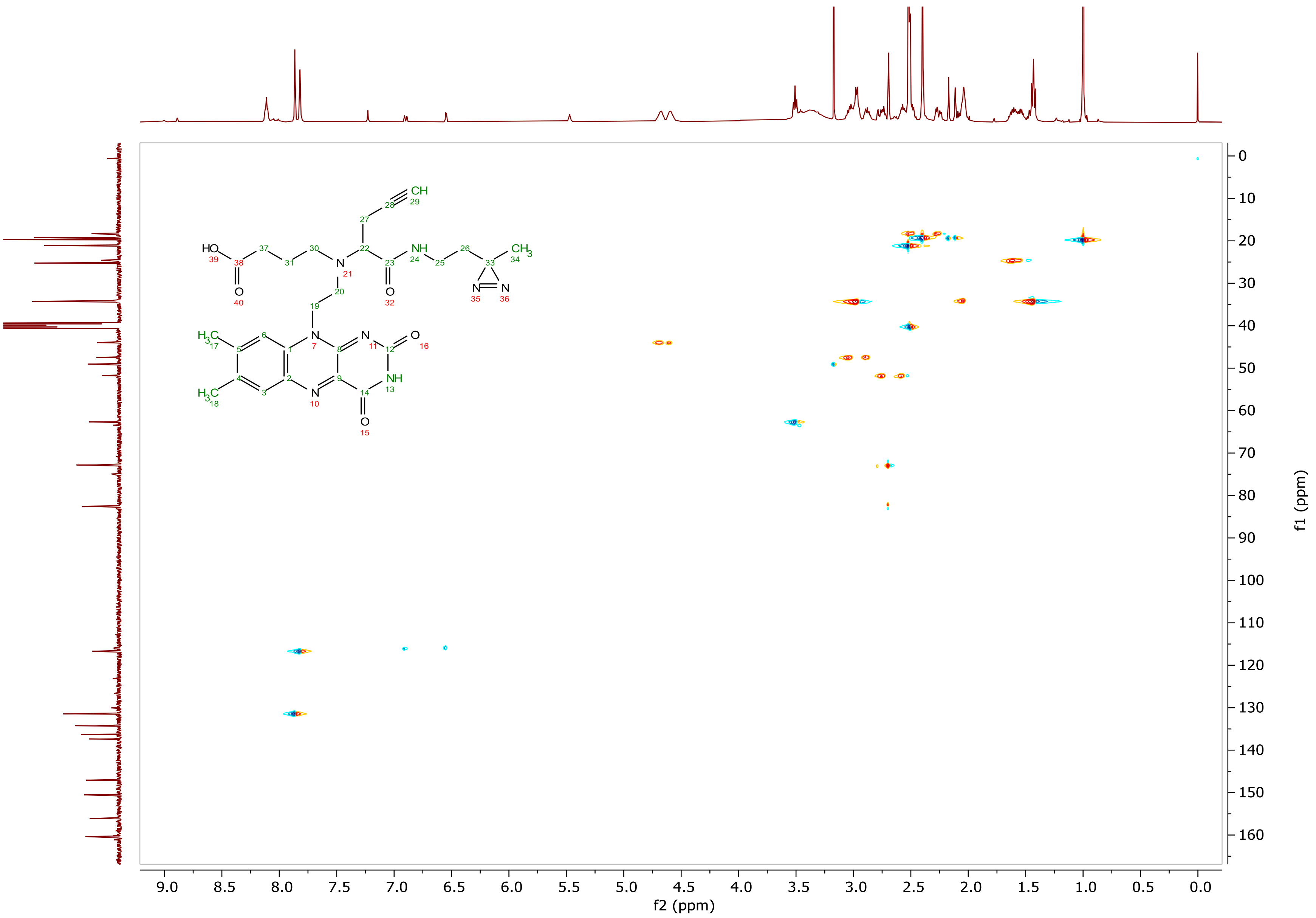
f2 (ppm)
9.0 8.5 8.0 7.5 7.0 6.5 6.0 5.5 5.0 4.5 4.0 3.5 3.0 2.5 2.0 1.5 1.0 0.5 0.0
f1 (ppm)
0 10 20 30 40 50 60 70 80 90 100 110 120 130 140 150 160

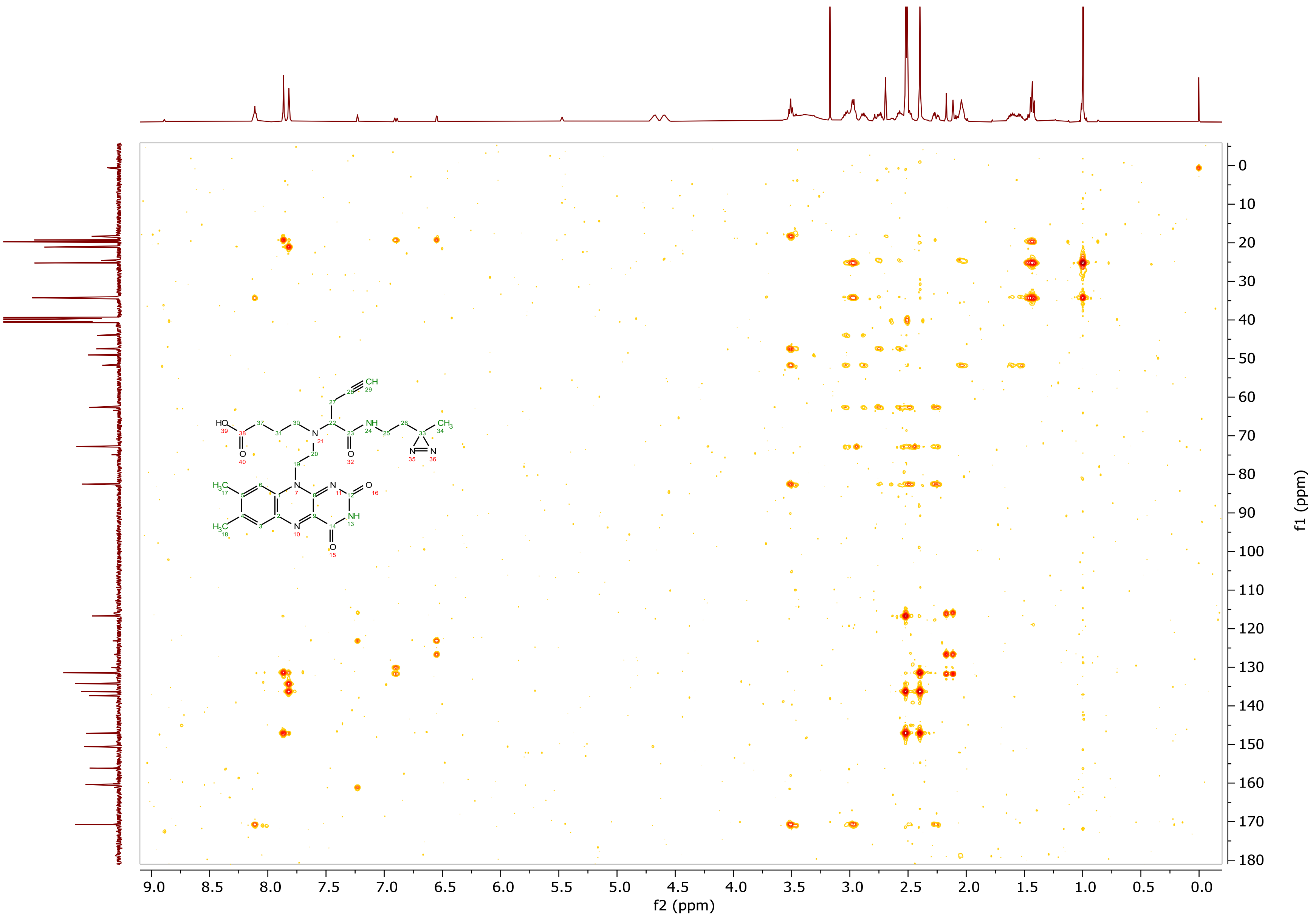

f2 (ppm)
f1 (ppm)

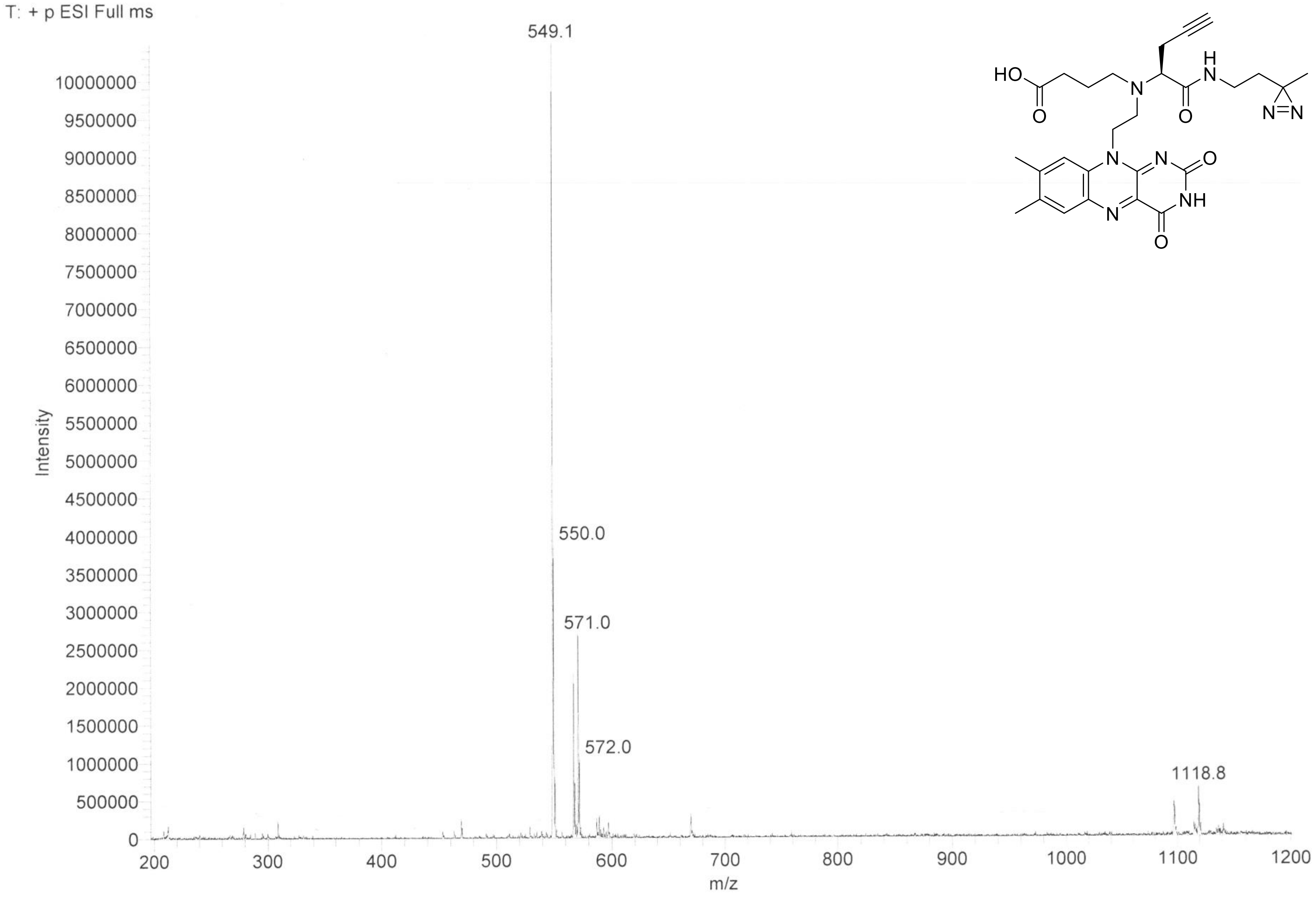
T: + p ESI Full ms
549.1
550.0
571.0
572.0
1118.8
Intensity
m/z
HO
O
N
O
H
N
N=N
N
N
O
NH
N
O

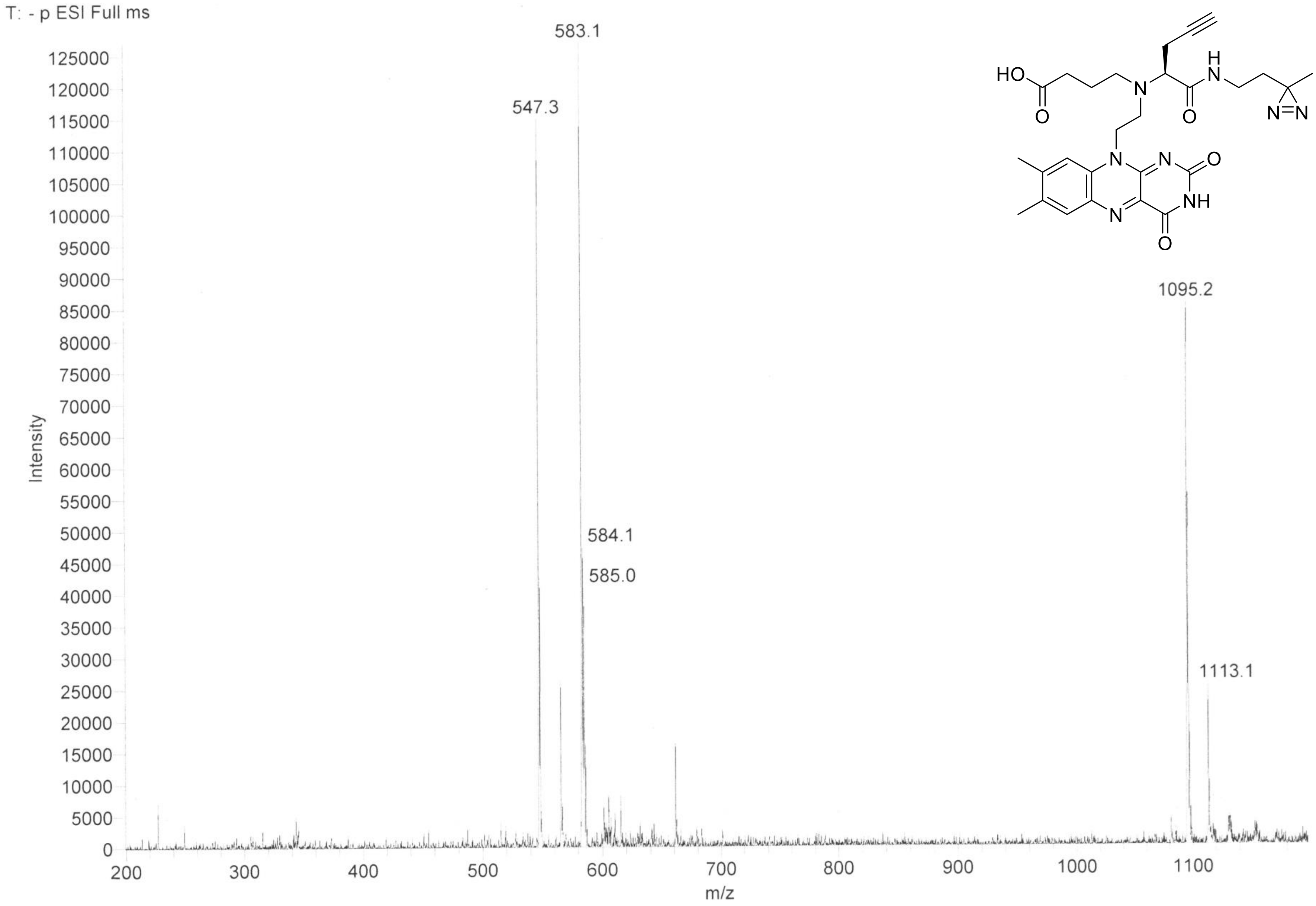
T: - p ESI Full ms
583.1
547.3
1095.2
584.1
585.0
1113.1
Intensity
m/z
HO
O
N
O
H
N
N=N
N
N
N
O
NH
O

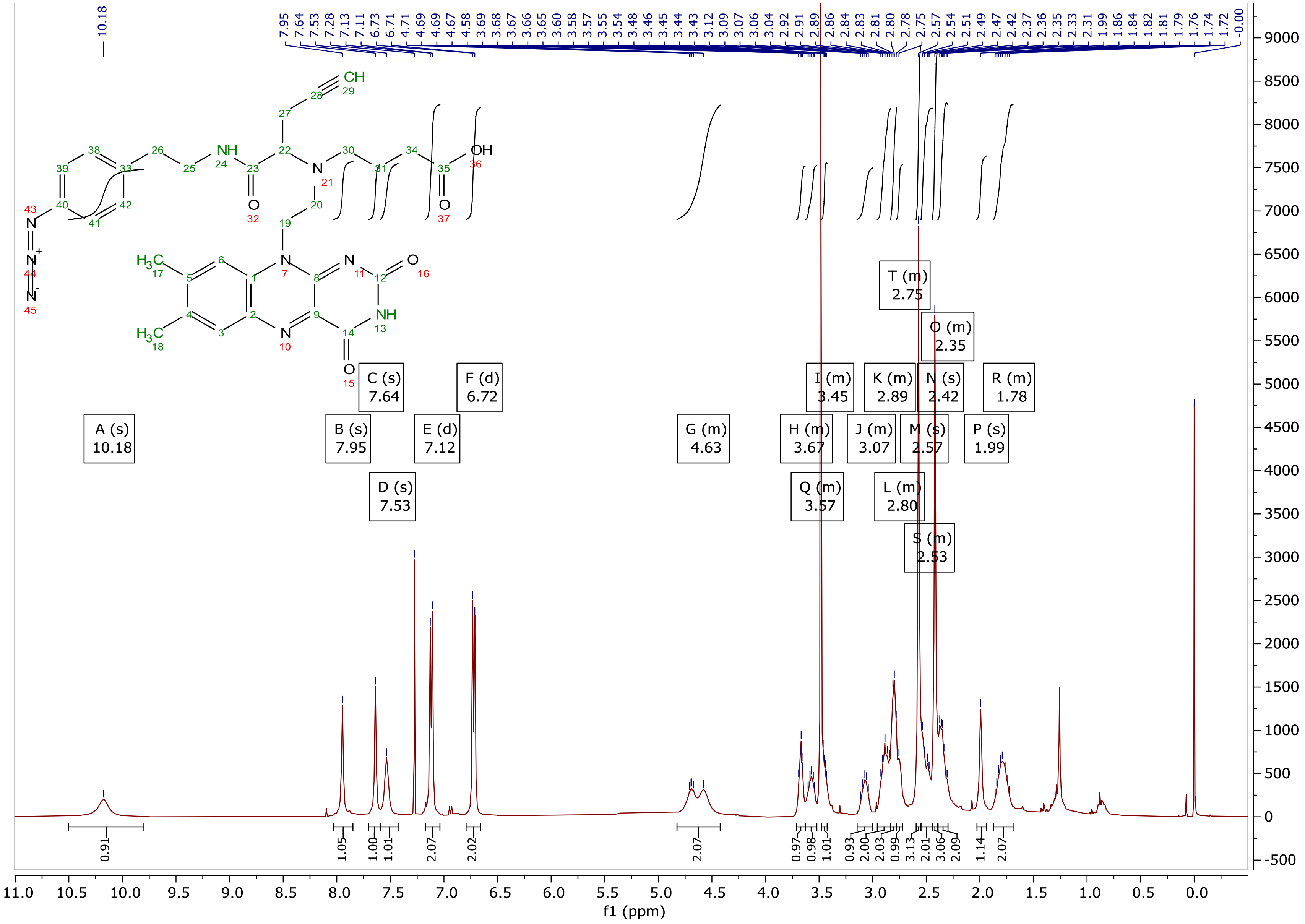
A (s) 10.18
B (s) 7.95
C (s) 7.64
D (s) 7.53
E (d) 7.12
F (d) 6.72
G (m) 4.63
H (m) 3.67
I (m) 3.45
Q (m) 3.57
J (m) 3.07
K (m) 2.89
L (m) 2.80
T (m) 2.75
M (s) 2.57
S (m) 2.53
N (s) 2.42
O (m) 2.35
P (s) 1.99
R (m) 1.78
f1 (ppm)

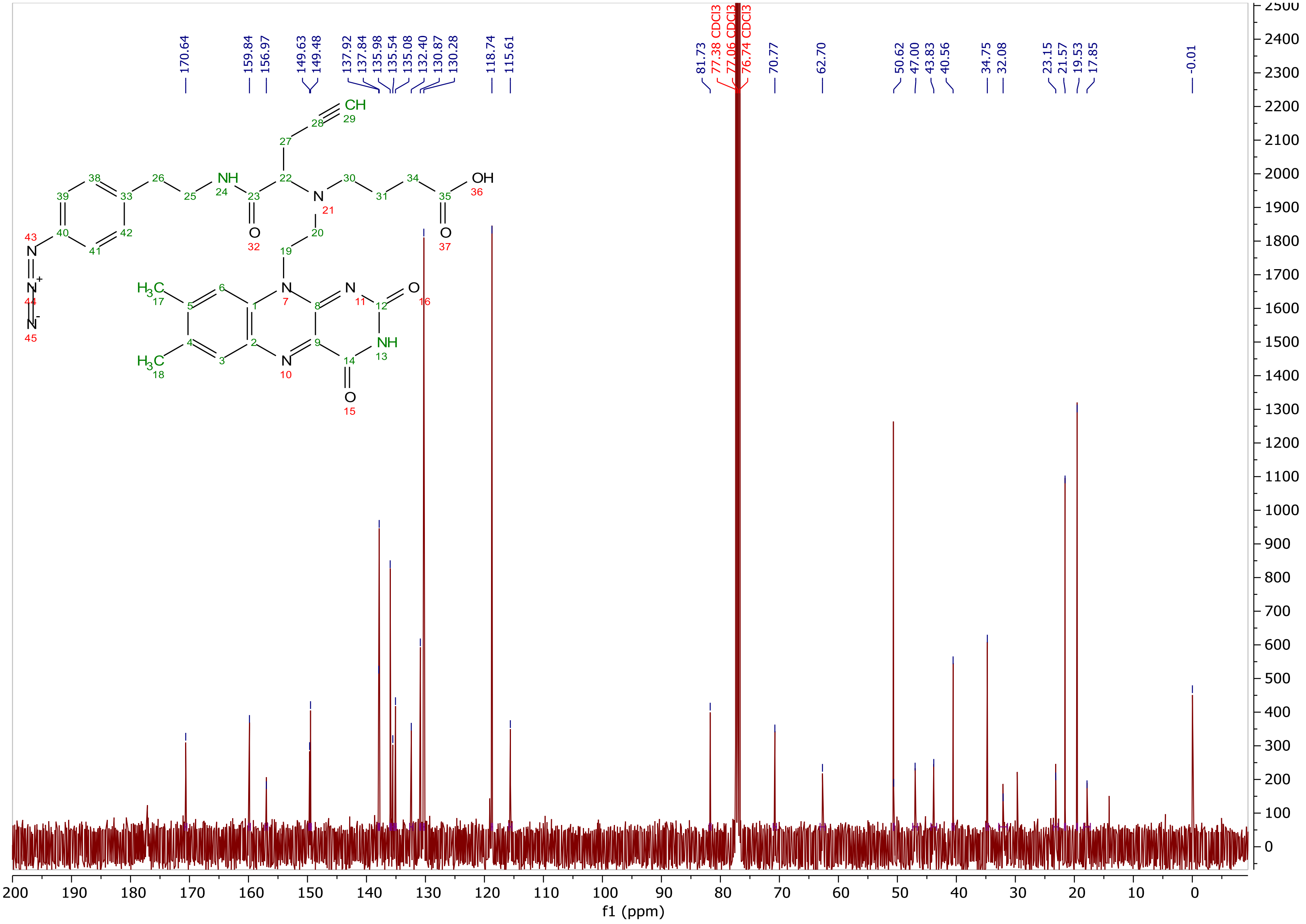
170.64
159.84
156.97
149.63
149.48
137.92
137.84
135.98
135.54
135.08
132.40
130.87
130.28
118.74
115.61
81.73
77.38 CDCl3
77.06 CDCl3
76.74 CDCl3
70.77
62.70
50.62
47.00
43.83
40.56
34.75
32.08
23.15
21.57
19.53
17.85
-0.01
f1 (ppm)

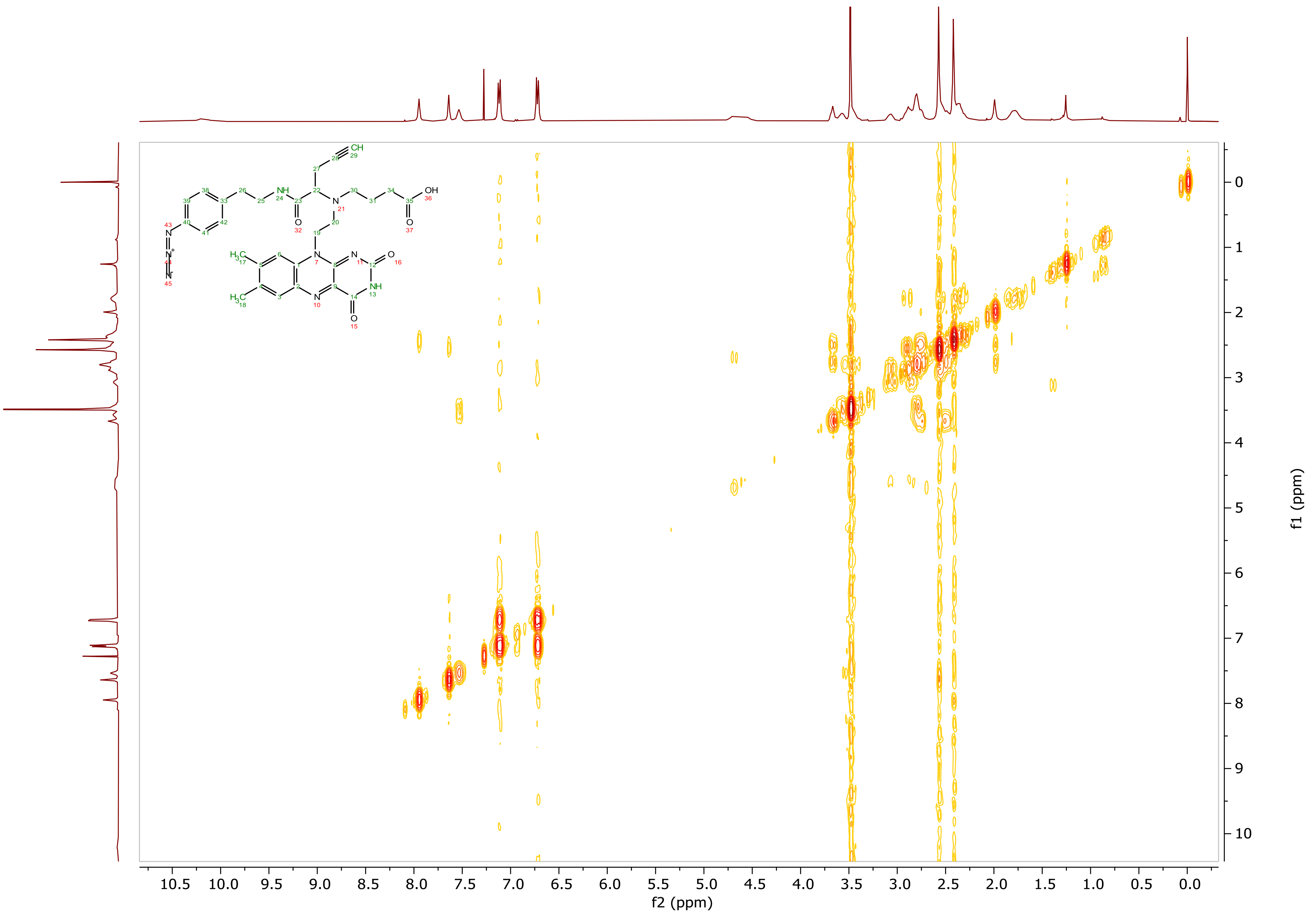
f2 (ppm)
f1 (ppm)

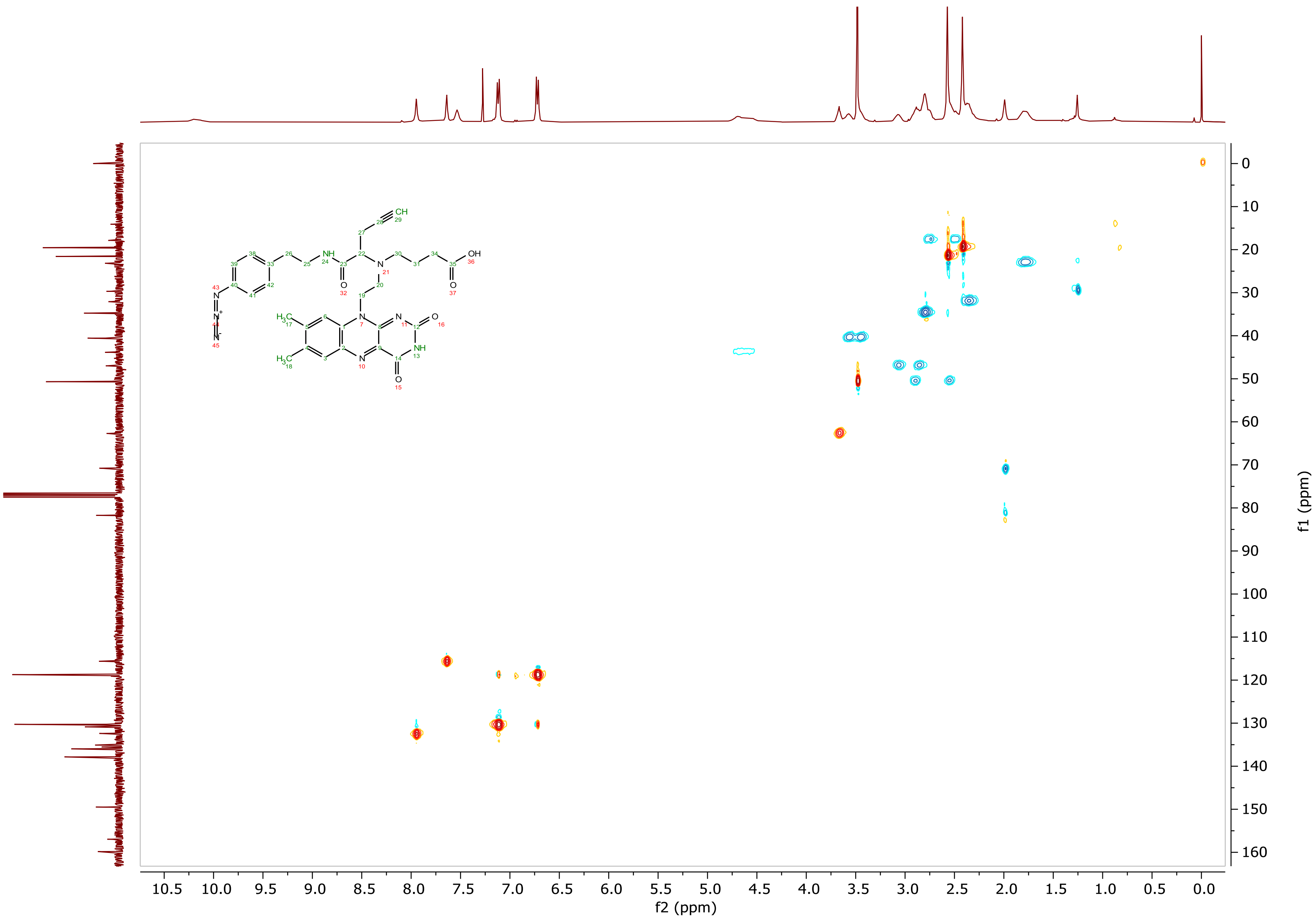
f2 (ppm)
f1 (ppm)

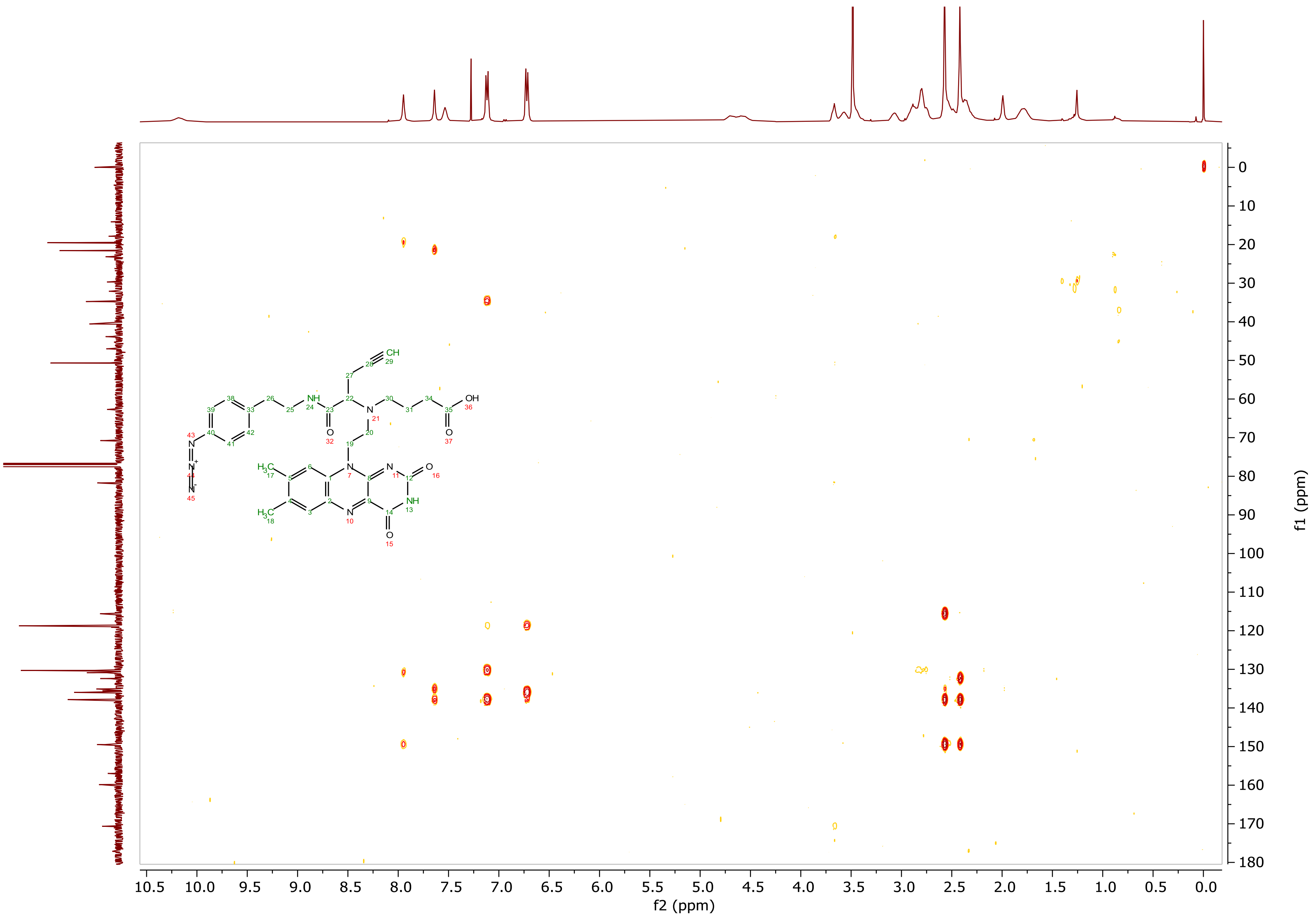
f2 (ppm)
f1 (ppm)

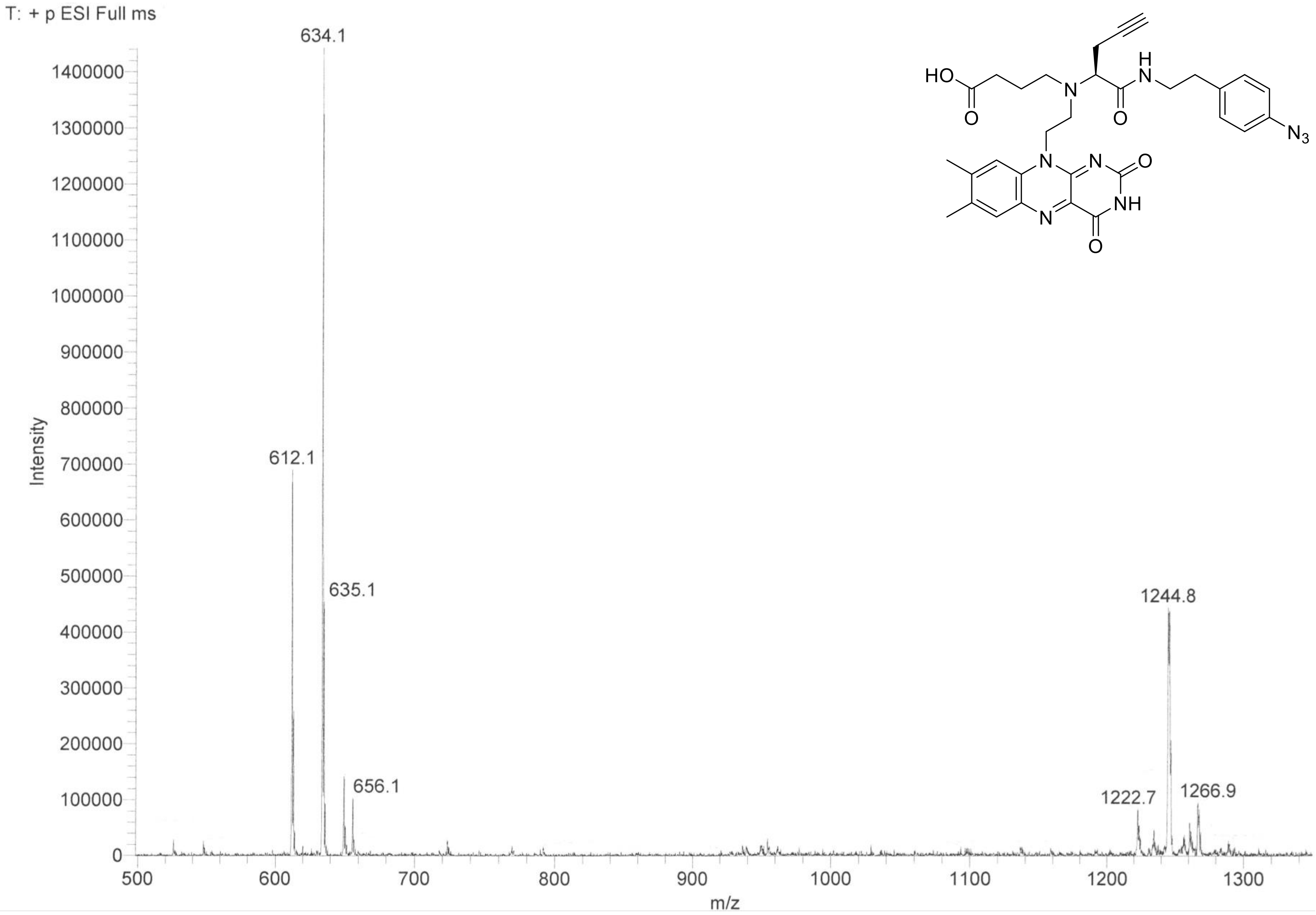

T: + p ESI Full ms
634.1
612.1
635.1
656.1
1244.8
1222.7
1266.9
Intensity
m/z
HO
O
N
O
H
N
N3
N
N
O
NH
O
N

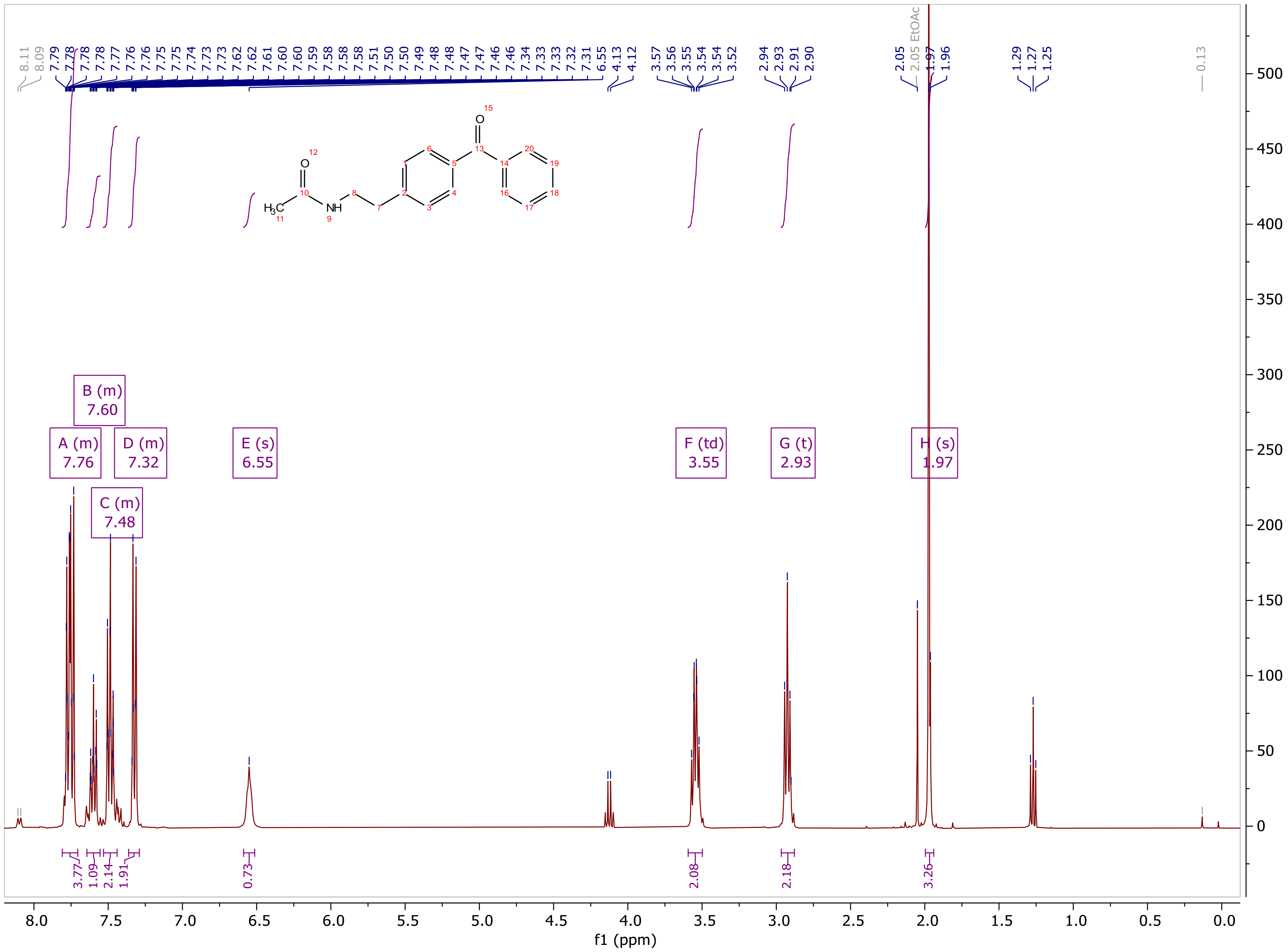
A (m) 7.76
B (m) 7.60
C (m) 7.48
D (m) 7.32
E (s) 6.55
F (td) 3.55
G (t) 2.93
H (s) 1.97
EtOAc
f1 (ppm)
3.77
1.09
2.14
1.91
0.73
2.08
2.18
3.26

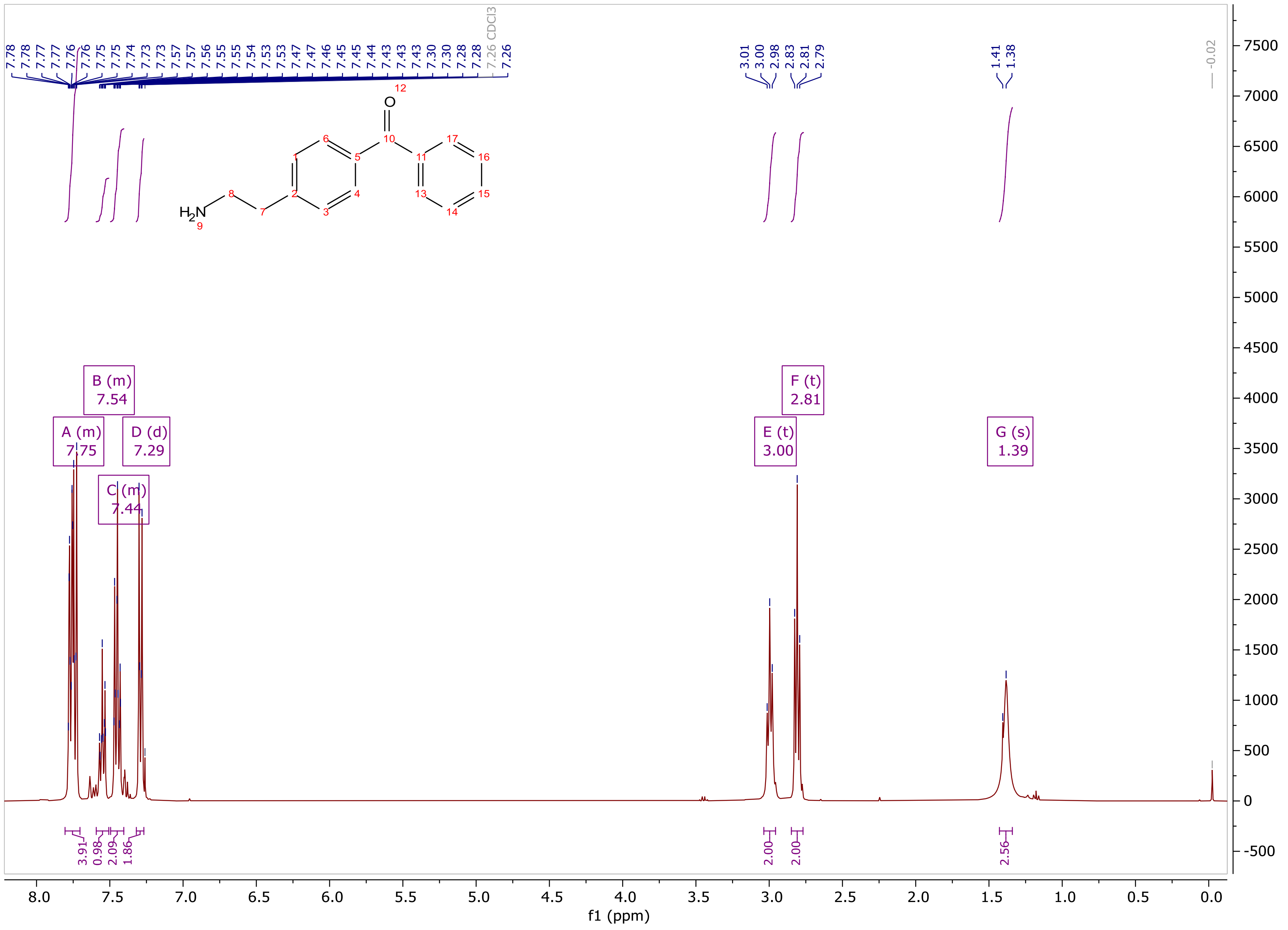
7.78 7.78 7.77 7.77 7.76 7.76 7.75 7.75 7.74 7.73 7.73 7.57 7.57 7.56 7.55 7.55 7.54 7.53 7.53 7.47 7.47 7.46 7.45 7.45 7.44 7.43 7.43 7.43 7.30 7.28 7.28 7.26 CDCl3 7.26
3.01 3.00 2.98 2.83 2.81 2.79
1.41 1.38
-0.02
A (m) 7.75
B (m) 7.54
C (m) 7.44
D (d) 7.29
E (t) 3.00
F (t) 2.81
G (s) 1.39
3.91 0.98 2.09 1.86 2.00 2.00 2.56
f1 (ppm)

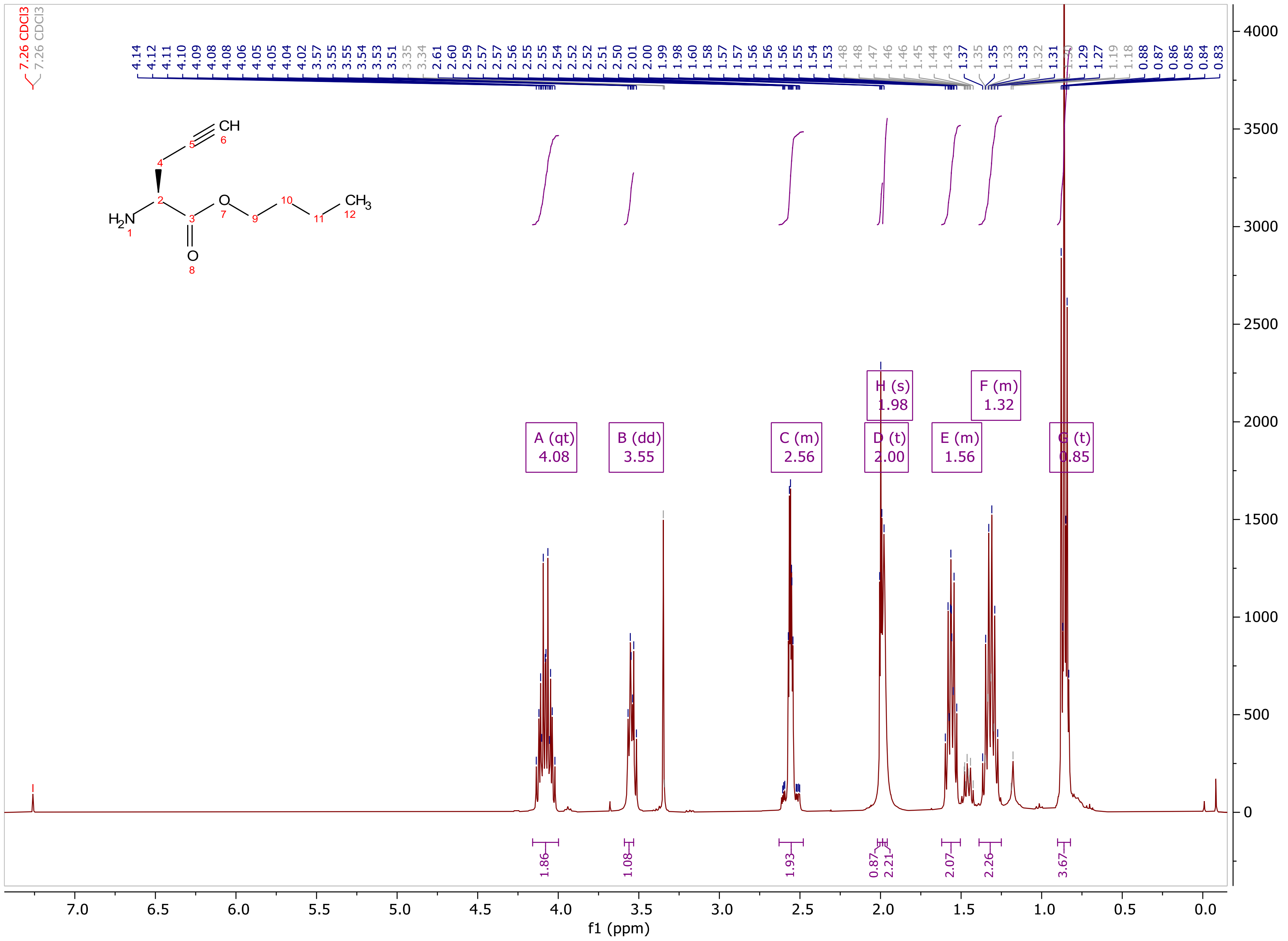

A (qt)
4.08
B (dd)
3.55
C (m)
2.56
H (s)
1.98
D (t)
2.00
E (m)
1.56
F (m)
1.32
G (t)
0.85
f1 (ppm)

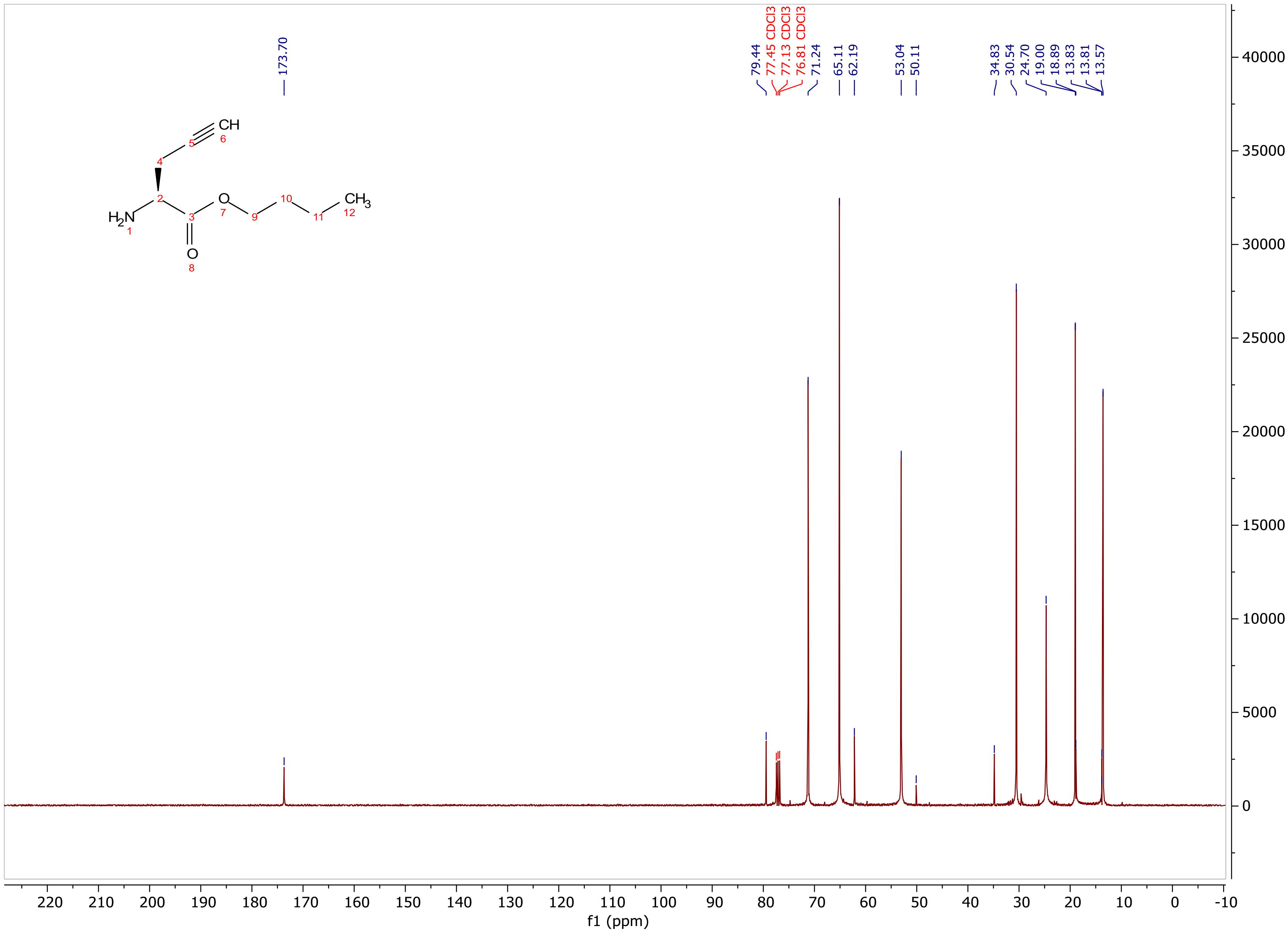
173.70
79.44
77.45 CDCl3
77.13 CDCl3
76.81 CDCl3
71.24
65.11
62.19
53.04
50.11
34.83
30.54
24.70
19.00
18.89
13.83
13.81
13.57
CH
H2N
O
CH3
O
f1 (ppm)

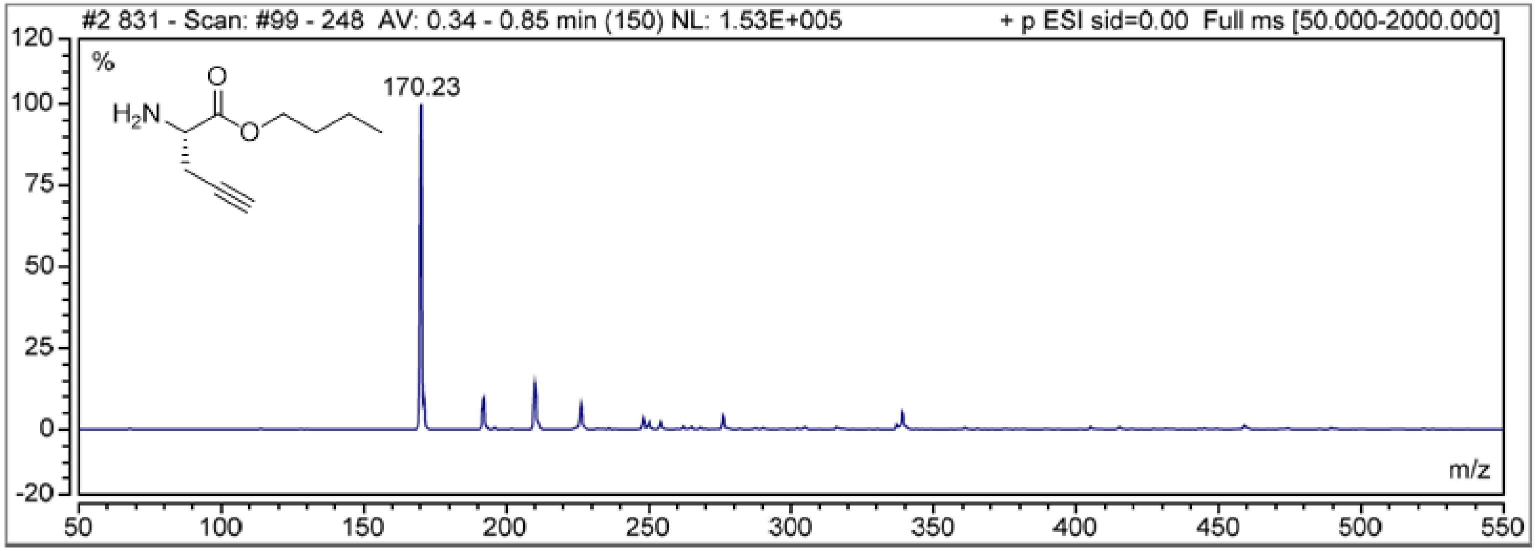

#2 831 - Scan: #99 - 248 AV: 0.34 - 0.85 min (150) NL: 1.53E+005
+ p ESI sid=0.00 Full ms [50.000-2000.000]
%
170.23
H2N
O
O
m/z
120
100
75
50
25
0
-20
50
100
150
200
250
300
350
400
450
500
550

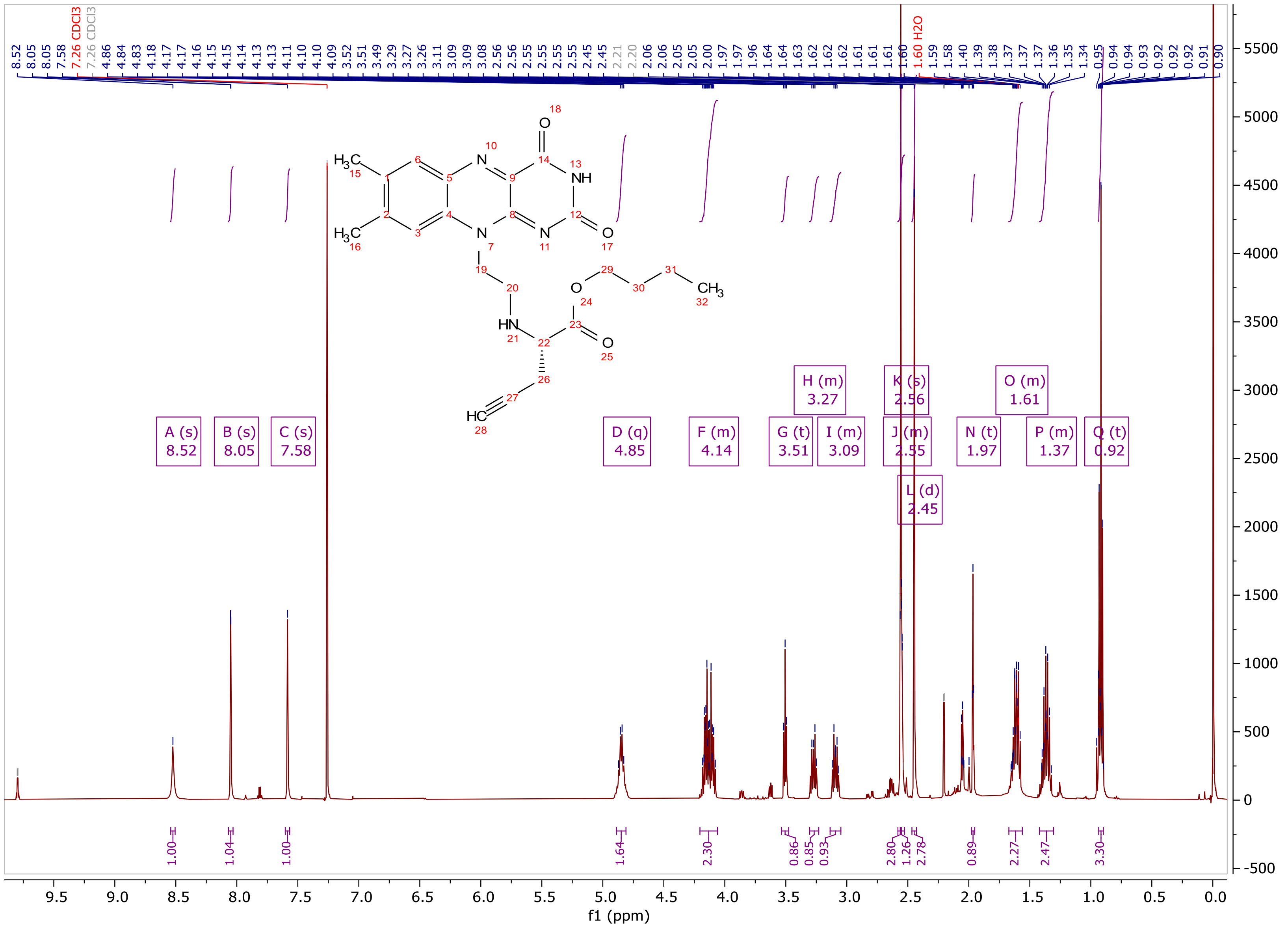

A (s) 8.52
B (s) 8.05
C (s) 7.58
D (q) 4.85
F (m) 4.14
G (t) 3.51
H (m) 3.27
I (m) 3.09
K (s) 2.56
J (m) 2.55
L (d) 2.45
N (t) 1.97
O (m) 1.61
P (m) 1.37
Q (t) 0.92
7.26 CDCl3
1.60 H2O
f1 (ppm)

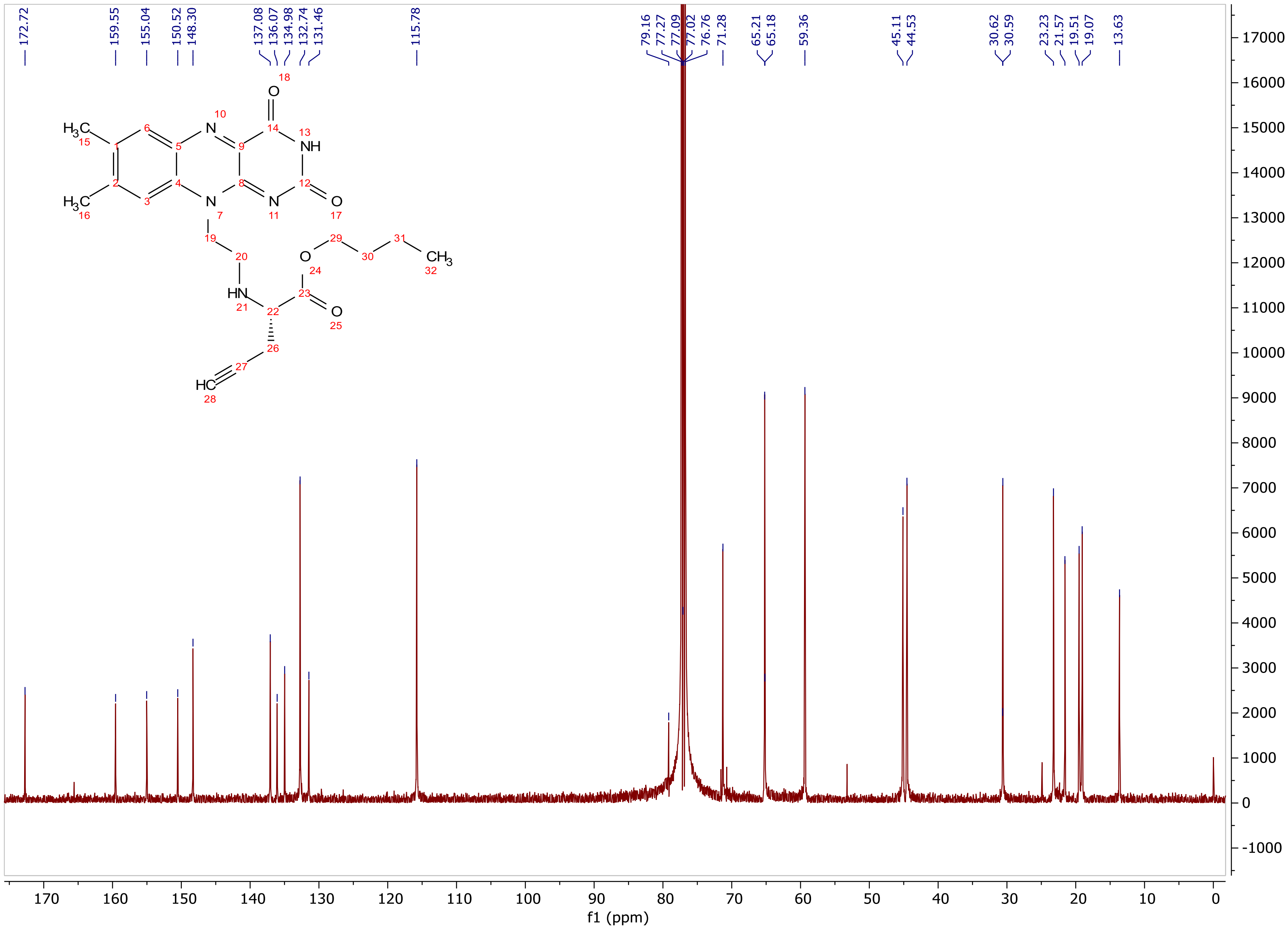
172.72
159.55
155.04
150.52
148.30
137.08
136.07
134.98
132.74
131.46
115.78
79.16
77.27
77.09
77.02
76.76
71.28
65.21
65.18
59.36
45.11
44.53
30.62
30.59
23.23
21.57
19.51
19.07
13.63
f1 (ppm)

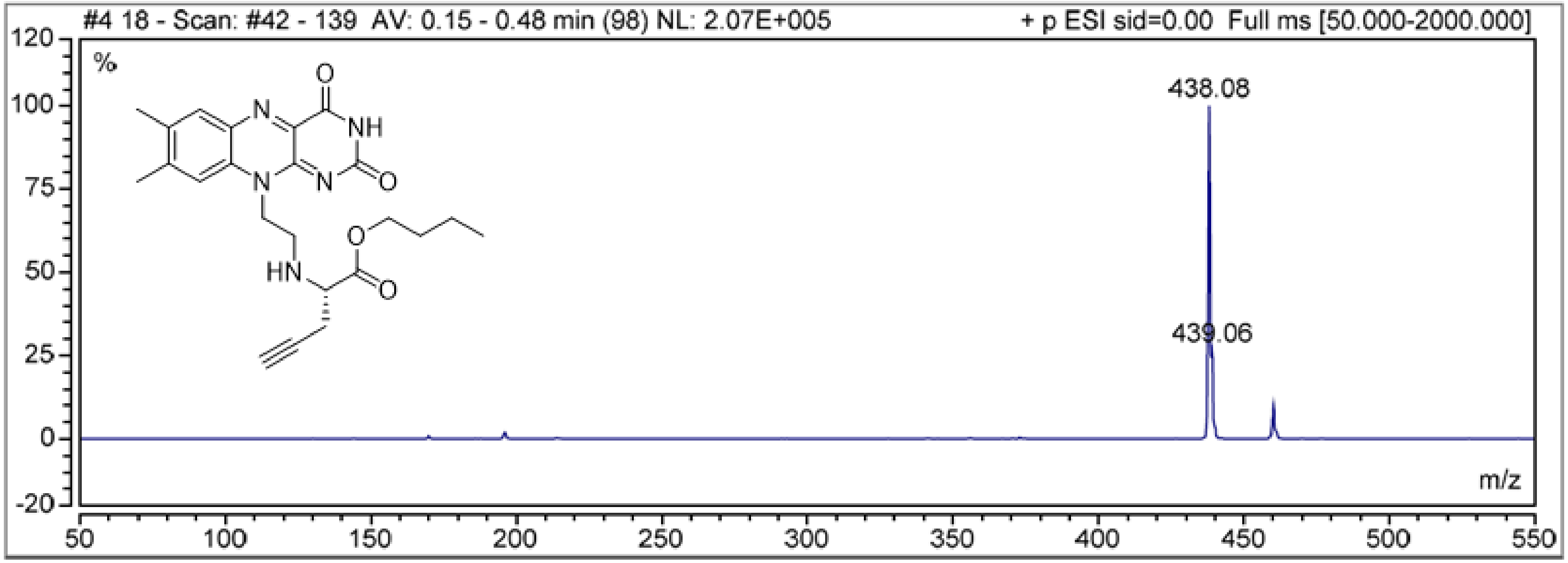
#4 18 - Scan: #42 - 139 AV: 0.15 - 0.48 min (98) NL: 2.07E+005
+ p ESI sid=0.00 Full ms [50.000-2000.000]
%
438.08
439.06
m/z
120
100
75
50
25
0
-20
50
100
150
200
250
300
350
400
450
500
550
O
N
NH
N
N
O
O
HN
O
O

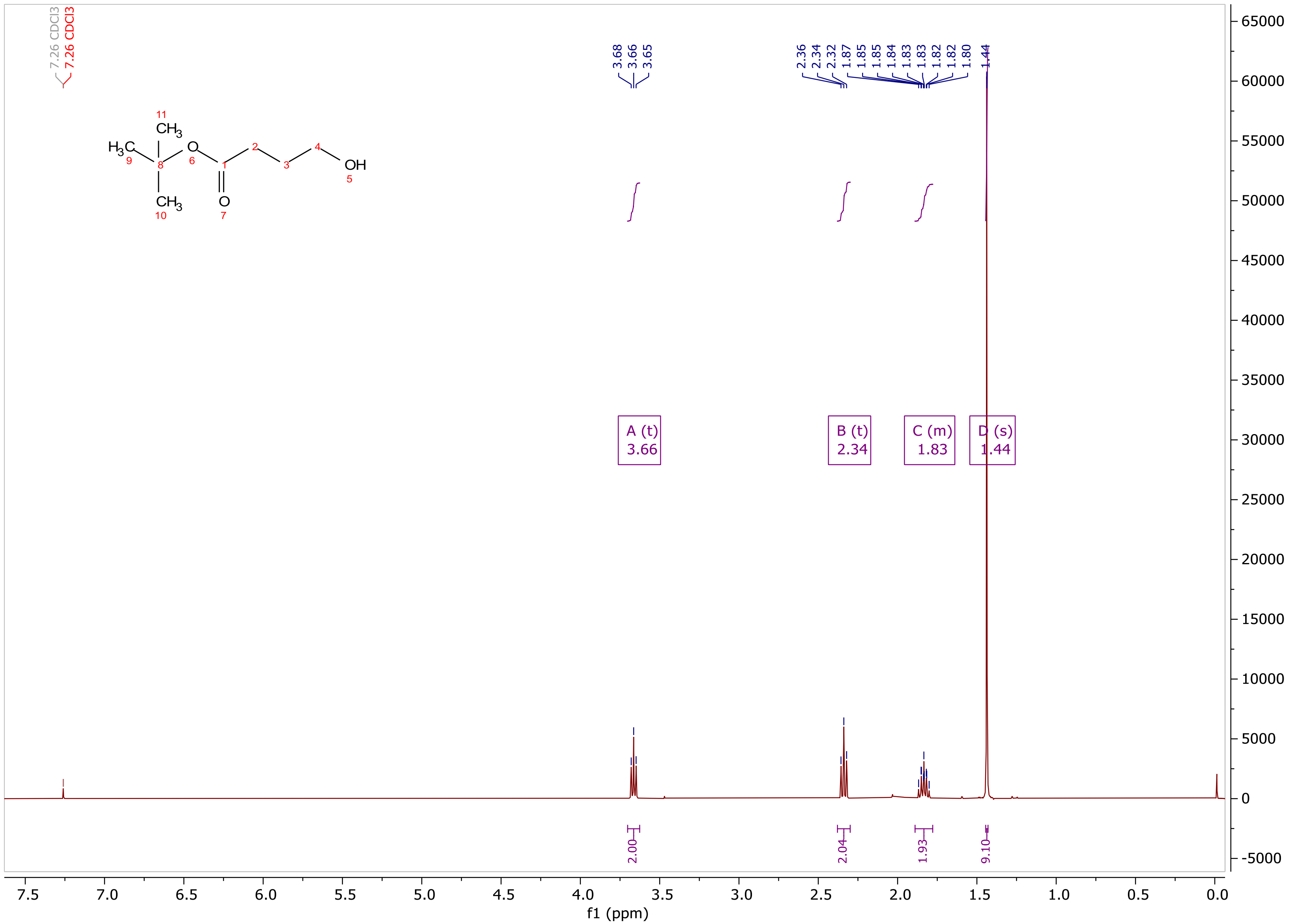

7.26 CDCl3
7.26 CDCl3
3.68
3.66
3.65
2.36
2.34
2.32
1.87
1.85
1.85
1.84
1.83
1.83
1.82
1.82
1.80
1.44
A (t)
3.66
B (t)
2.34
C (m)
1.83
D (s)
1.44
2.00
2.04
1.93
9.10
f1 (ppm)

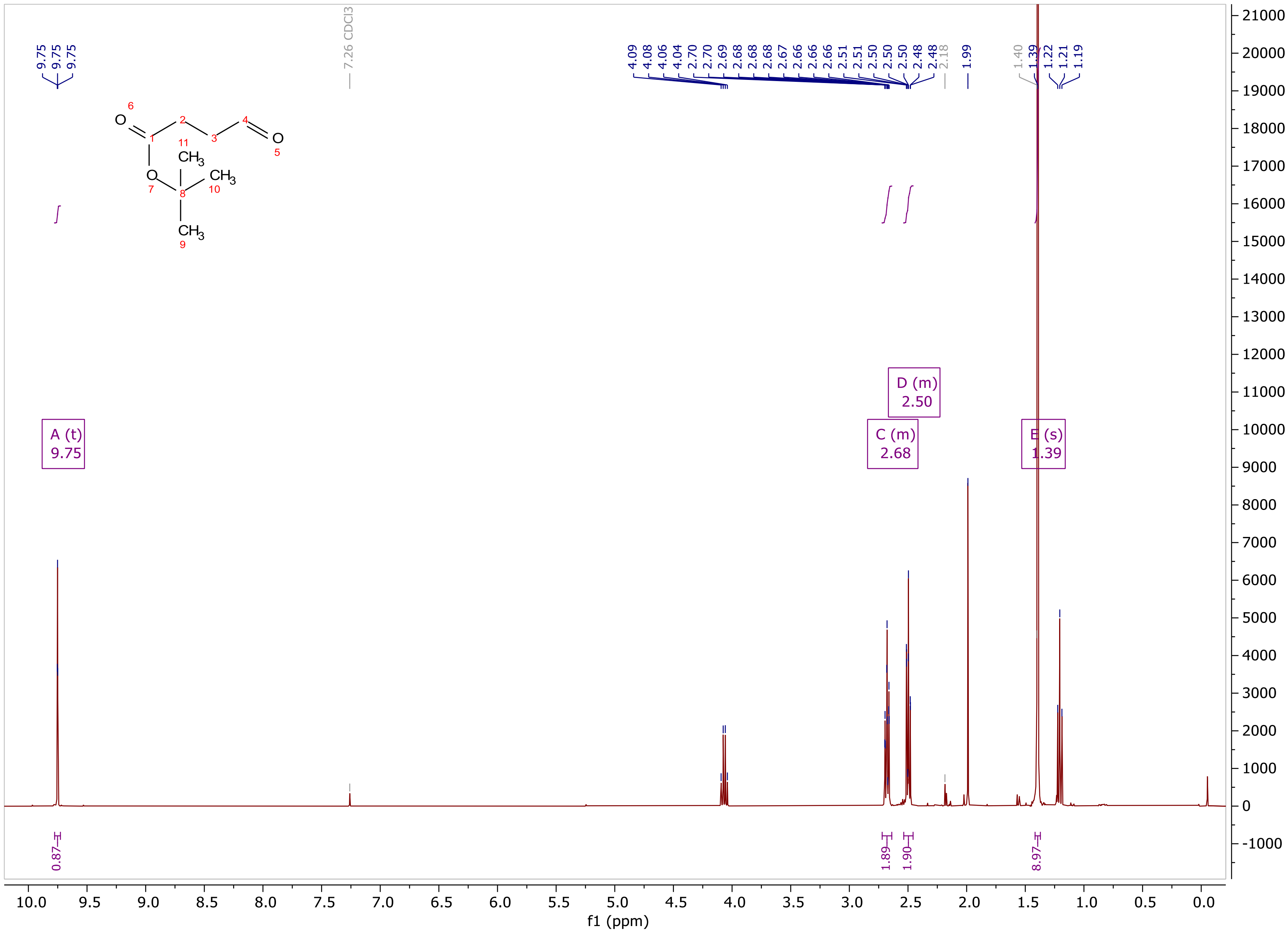
9.75
9.75
9.75
7.26 CDCl3
4.09
4.08
4.06
4.04
2.70
2.70
2.69
2.68
2.68
2.68
2.67
2.66
2.66
2.66
2.51
2.51
2.50
2.50
2.50
2.48
2.48
2.18
1.99
1.40
1.39
1.22
1.21
1.19
A (t)
9.75
C (m)
2.68
D (m)
2.50
E (s)
1.39
0.87
1.89
1.90
8.97
f1 (ppm)

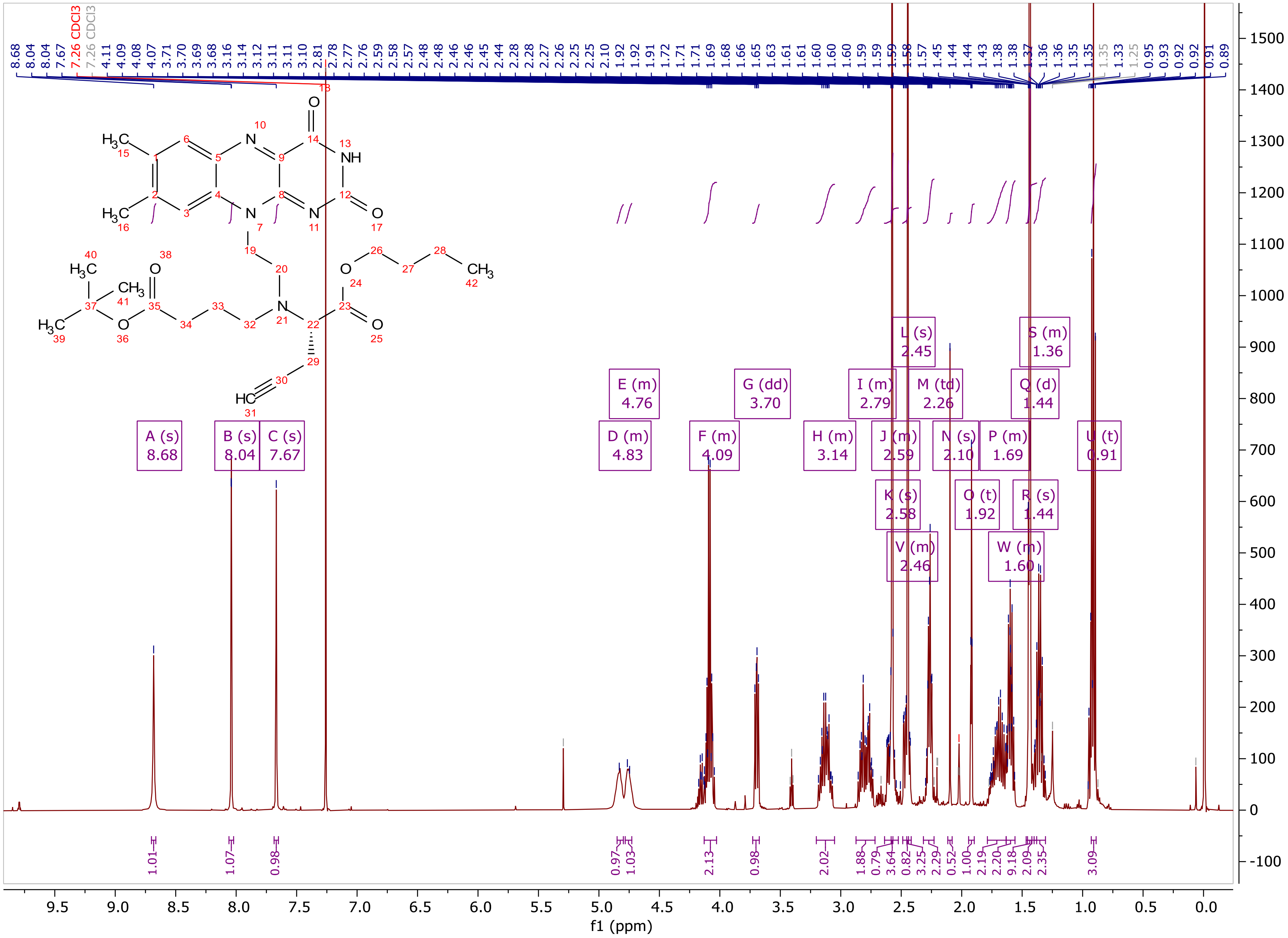
A (s) 8.68
B (s) 8.04
C (s) 7.67
D (m) 4.83
E (m) 4.76
F (m) 4.09
G (dd) 3.70
H (m) 3.14
I (m) 2.79
J (m) 2.59
K (s) 2.58
L (s) 2.45
M (td) 2.26
N (s) 2.10
O (t) 1.92
P (m) 1.69
Q (d) 1.44
R (s) 1.44
S (m) 1.36
U (t) 0.91
V (m) 2.46
W (m) 1.60
f1 (ppm)

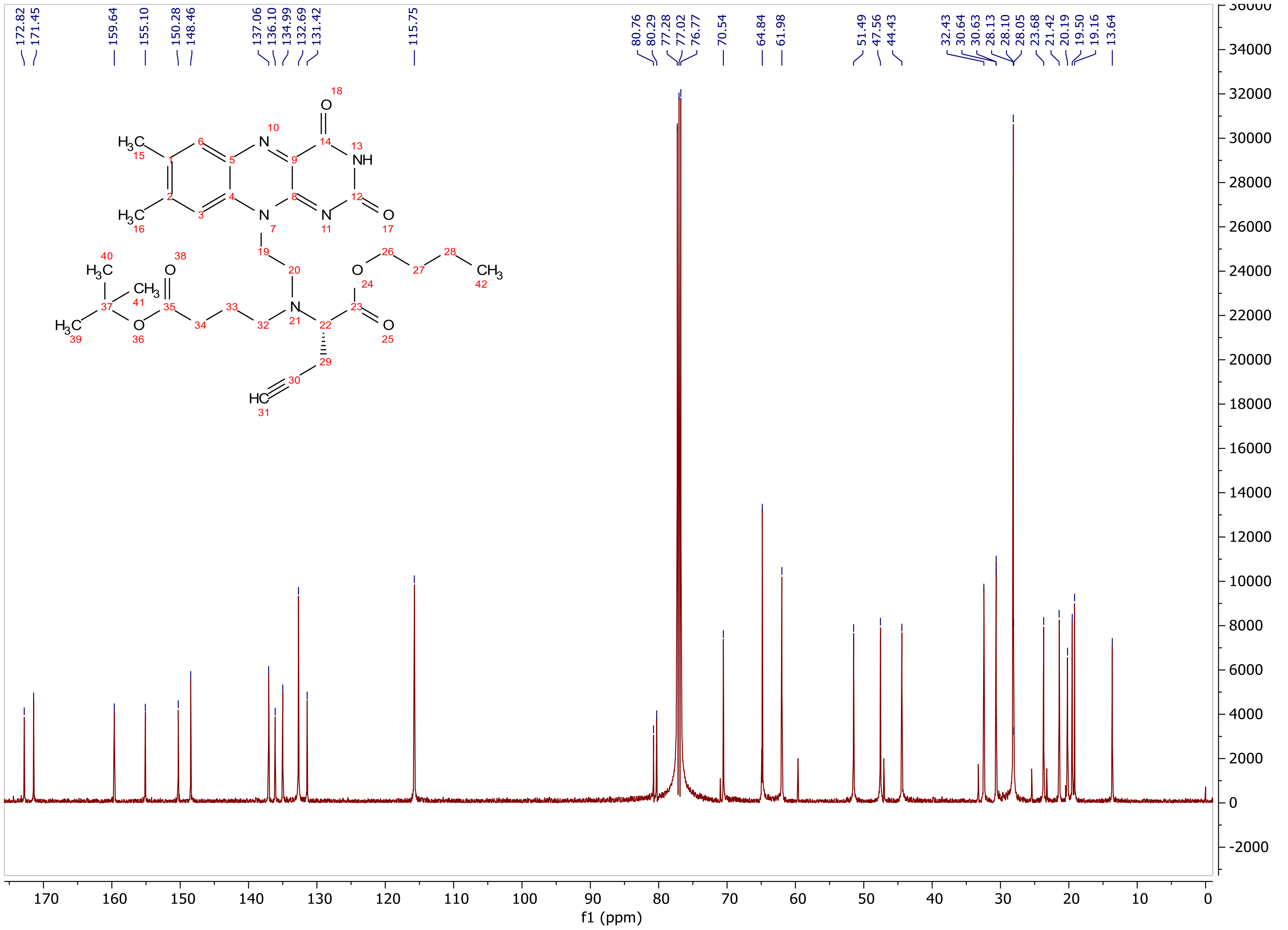
172.82
171.45
159.64
155.10
150.28
148.46
137.06
136.10
134.99
132.69
131.42
115.75
80.76
80.29
77.28
77.02
76.77
70.54
64.84
61.98
51.49
47.56
44.43
32.43
30.64
30.63
28.13
28.10
28.05
23.68
21.42
20.19
19.50
19.16
13.64
f1 (ppm)

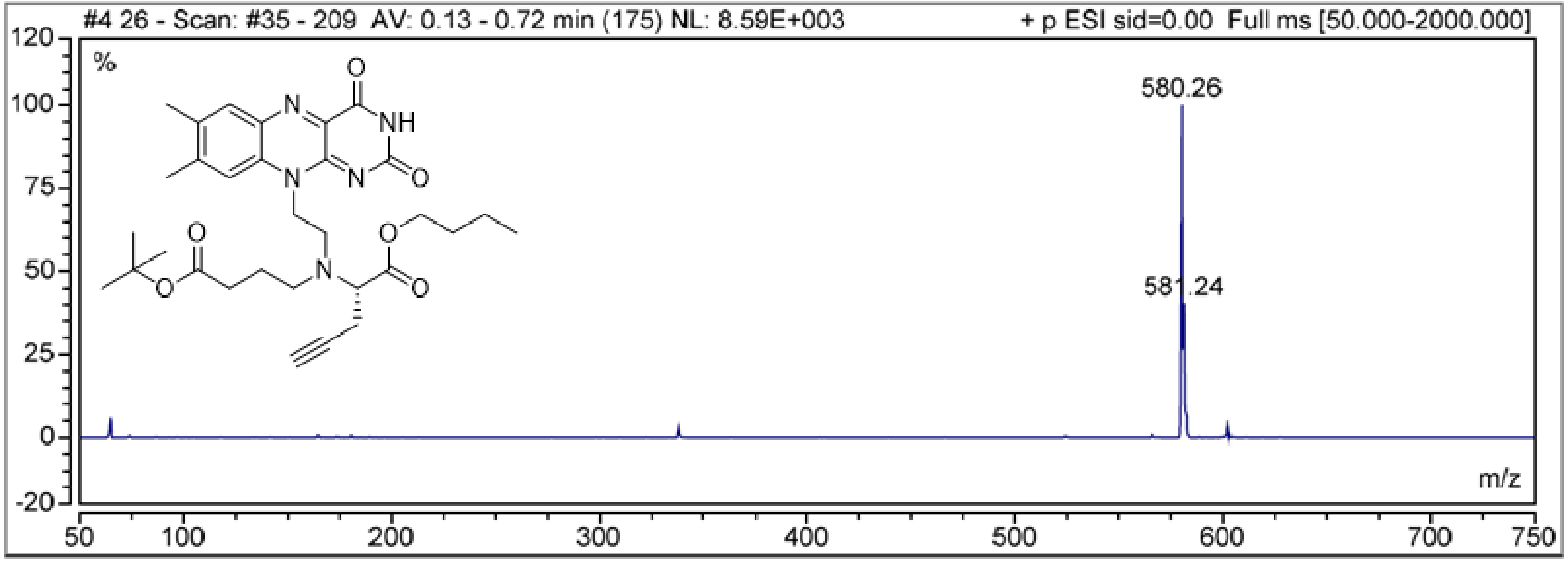
#4 26 - Scan: #35 - 209 AV: 0.13 - 0.72 min (175) NL: 8.59E+003
+ p ESI sid=0.00 Full ms [50.000-2000.000]
%
580.26
581.24
m/z
120
100
75
50
25
0
-20
50
100
200
300
400
500
600
700
750
O
N
NH
N
N
O
O
O
O
N
O
O

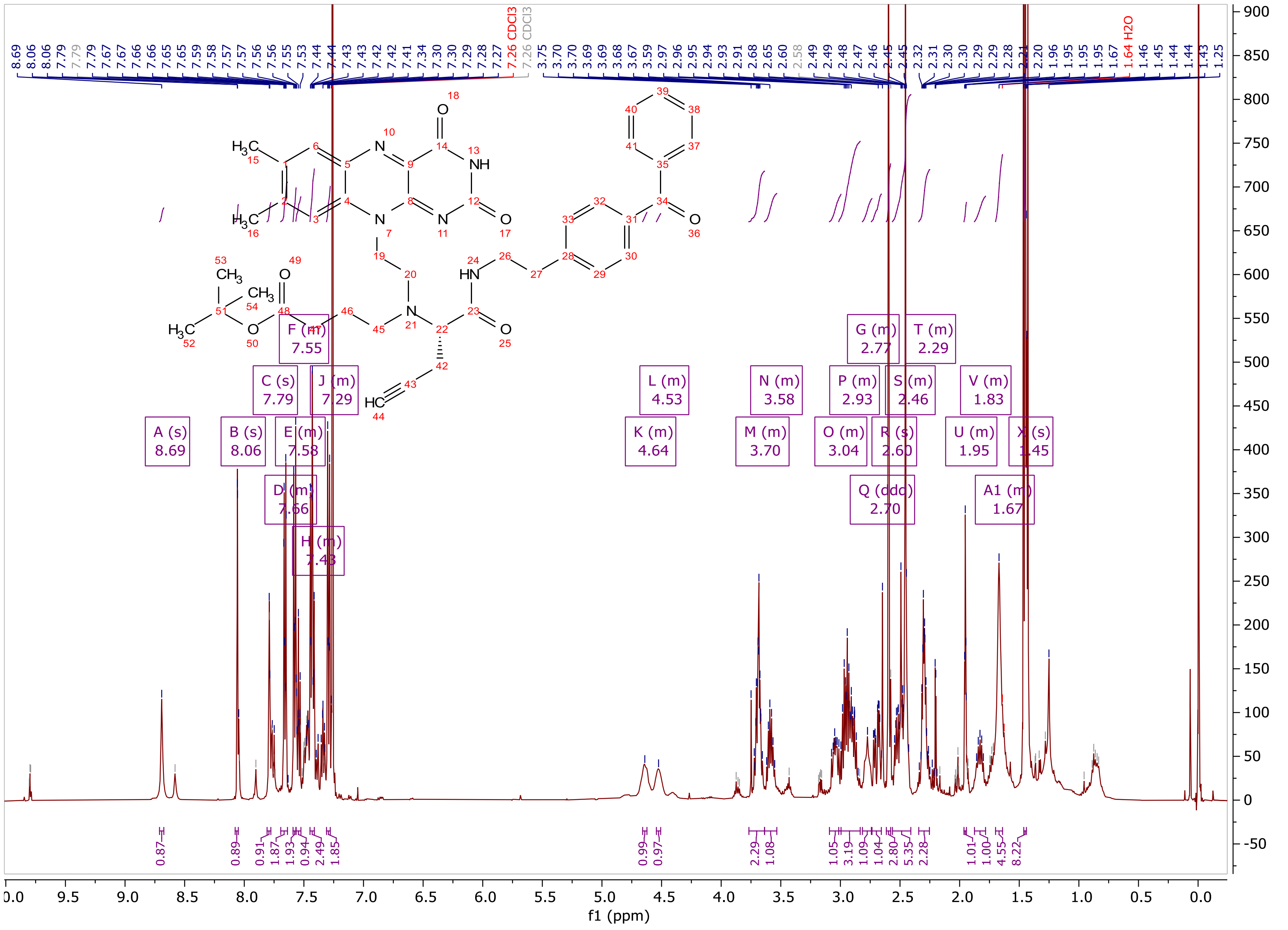

f1 (ppm)
A (s) 8.69
B (s) 8.06
C (s) 7.79
D (m) 7.66
E (m) 7.58
F (m) 7.55
H (m) 7.43
J (m) 7.29
K (m) 4.64
L (m) 4.53
M (m) 3.70
N (m) 3.58
O (m) 3.04
P (m) 2.93
G (m) 2.77
Q (ddd) 2.70
R (s) 2.60
S (m) 2.46
T (m) 2.29
U (m) 1.95
V (m) 1.83
A1 (m) 1.67
X (s) 1.45
7.26 CDCl3
1.64 H2O

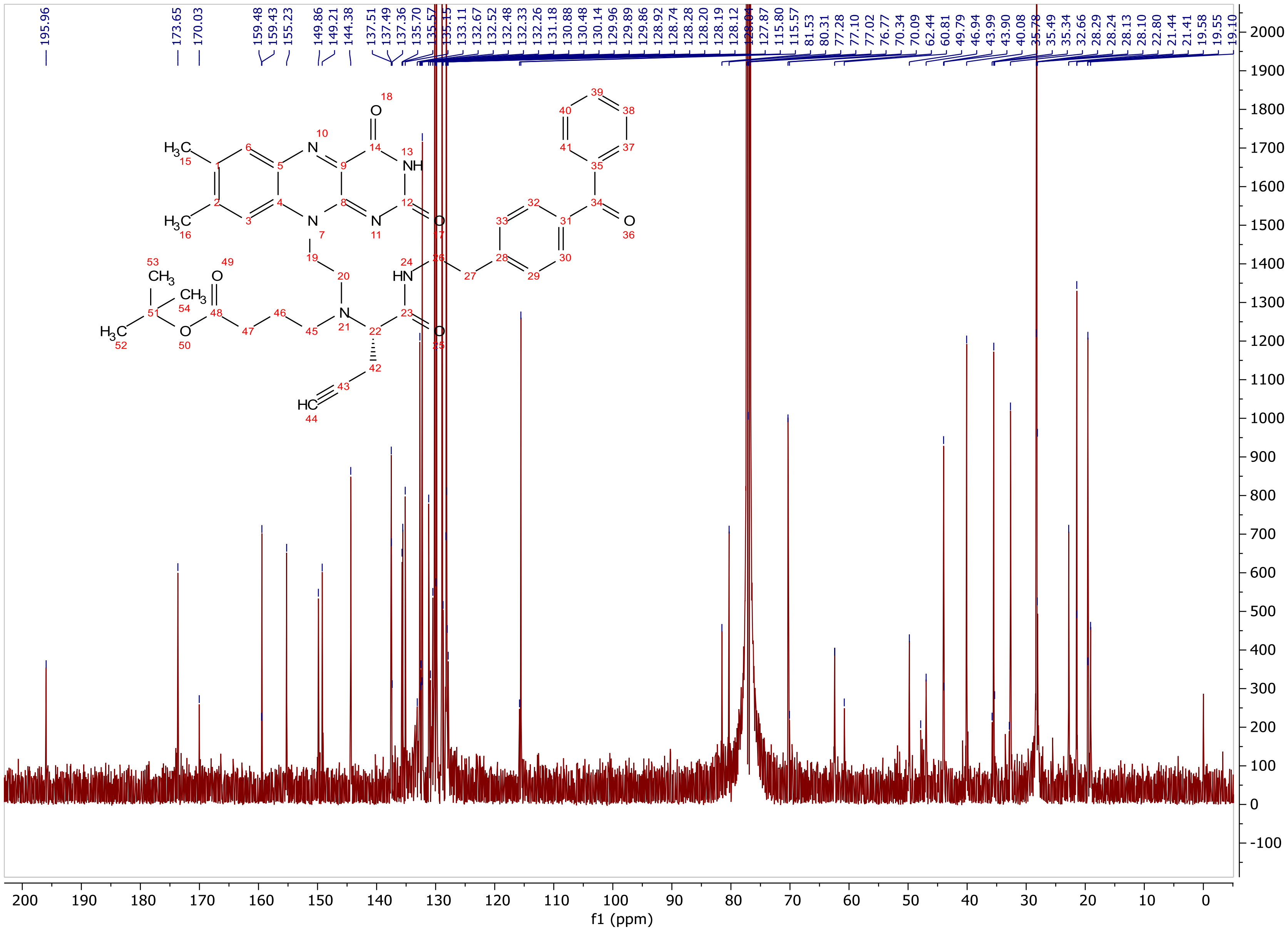
195.96
173.65
170.03
159.48
159.43
155.23
149.86
149.21
144.38
137.51
137.49
137.36
135.70
135.57
135.15
133.11
132.67
132.52
132.48
132.33
132.26
131.18
130.88
130.48
130.14
129.96
129.89
129.86
128.92
128.74
128.28
128.20
128.19
128.12
127.87
115.80
115.57
81.53
80.31
77.28
77.10
77.02
76.77
70.34
70.09
62.44
60.81
49.79
46.94
43.99
43.90
40.08
35.78
35.49
35.34
32.66
28.29
28.24
28.13
28.10
22.80
21.44
21.41
19.58
19.55
19.10
f1 (ppm)

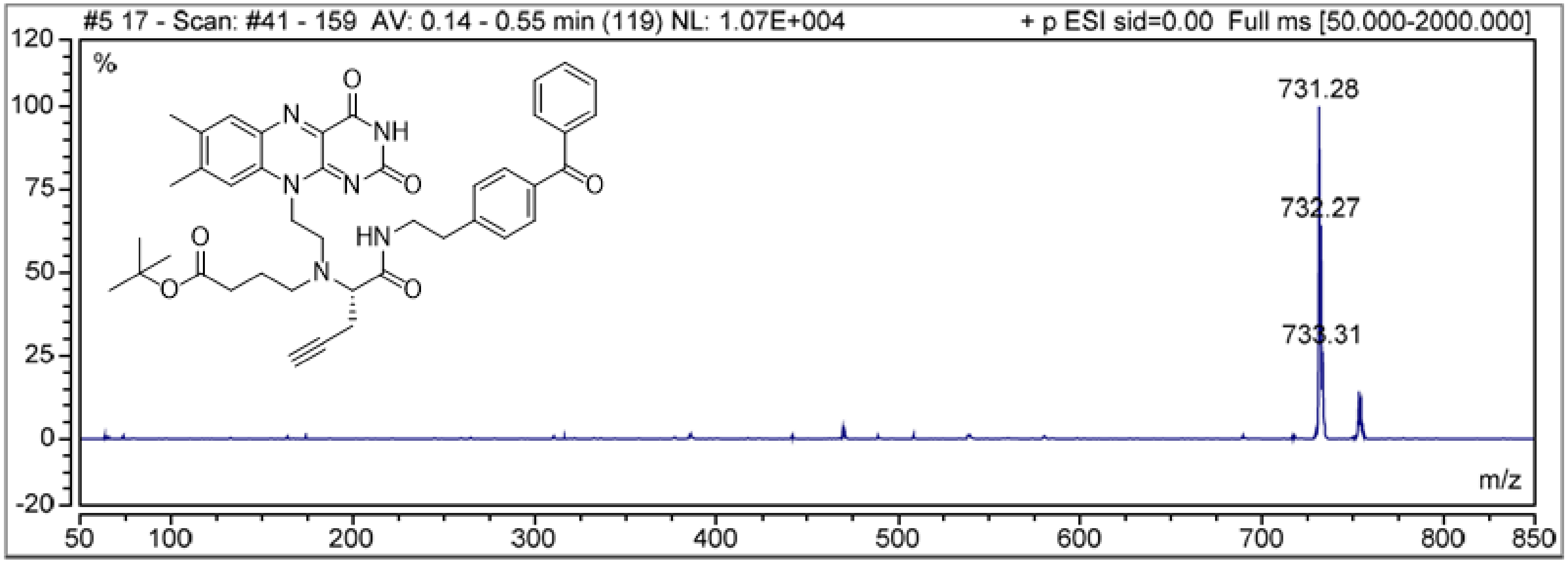

#5 17 - Scan: #41 - 159 AV: 0.14 - 0.55 min (119) NL: 1.07E+004
+ p ESI sid=0.00 Full ms [50.000-2000.000]
%
731.28
732.27
733.31
m/z
120
100
75
50
25
0
-20
50
100
200
300
400
500
600
700
800
850

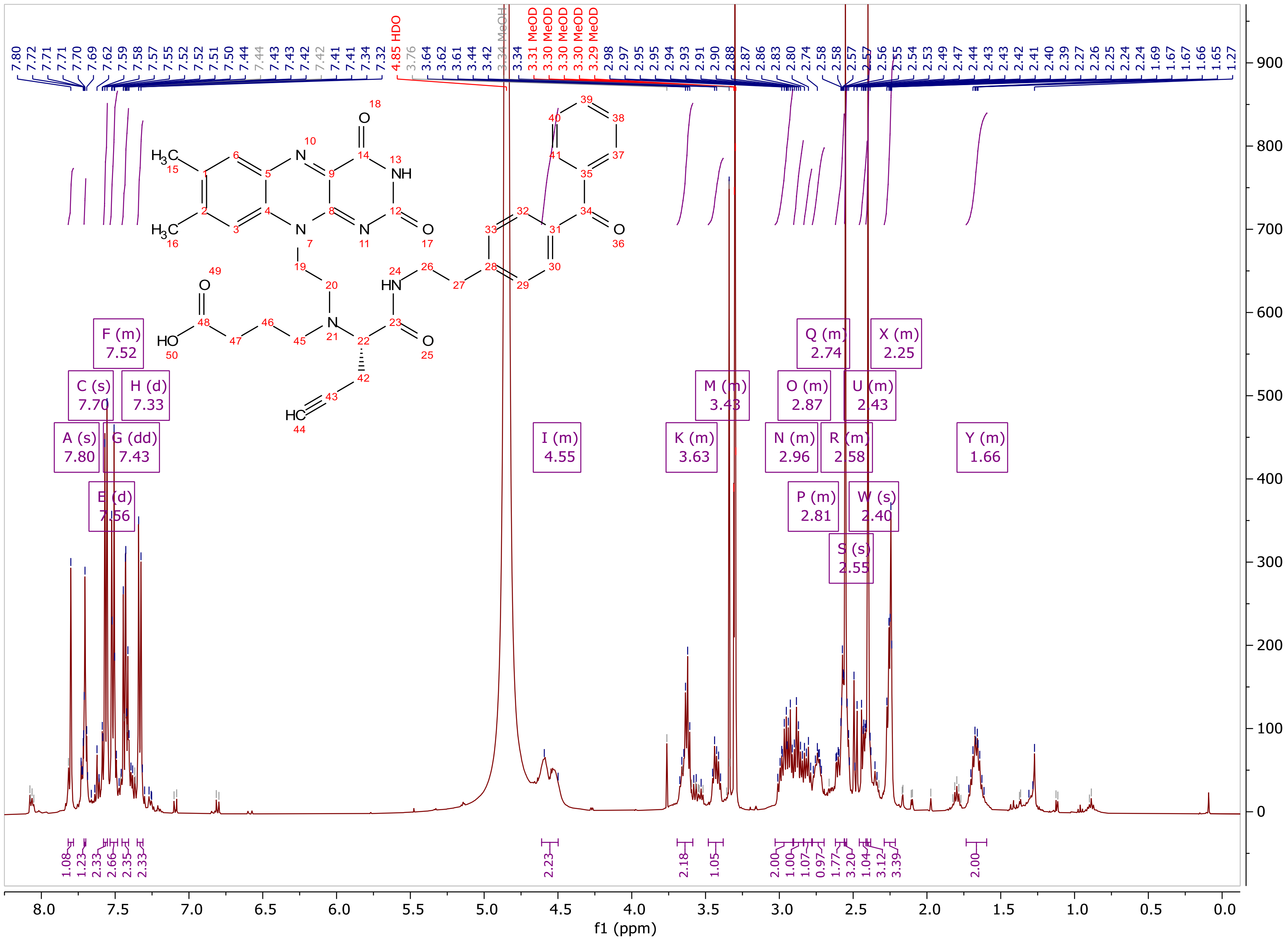
A (s)
7.80
C (s)
7.70
E (d)
7.56
F (m)
7.52
G (dd)
7.43
H (d)
7.33
I (m)
4.55
K (m)
3.63
M (m)
3.43
N (m)
2.96
O (m)
2.87
P (m)
2.81
Q (m)
2.74
R (m)
2.58
S (s)
2.55
U (m)
2.43
W (s)
2.40
X (m)
2.25
Y (m)
1.66
f1 (ppm)

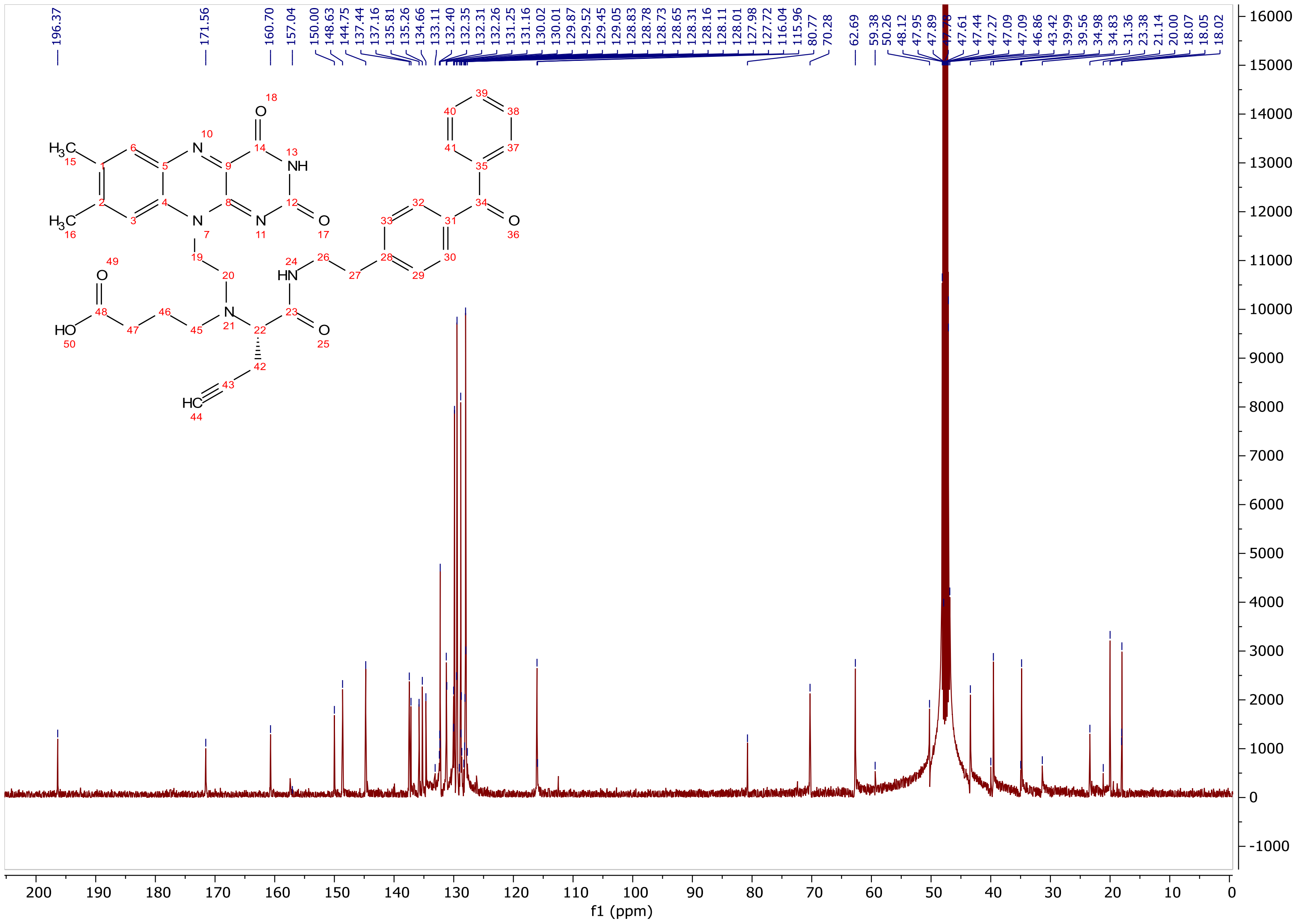

196.37
171.56
160.70
157.04
150.00
148.63
144.75
137.44
137.16
135.81
135.26
134.66
133.11
132.40
132.35
132.31
132.26
131.25
131.16
130.02
130.01
129.87
129.52
129.45
129.05
128.83
128.78
128.73
128.65
128.31
128.16
128.11
128.01
127.98
127.72
116.04
115.96
80.77
70.28
62.69
59.38
50.26
48.12
47.95
47.89
47.61
47.44
47.27
47.09
46.86
43.42
39.99
39.56
34.98
34.83
31.36
23.38
21.14
20.00
18.07
18.05
18.02
f1 (ppm)

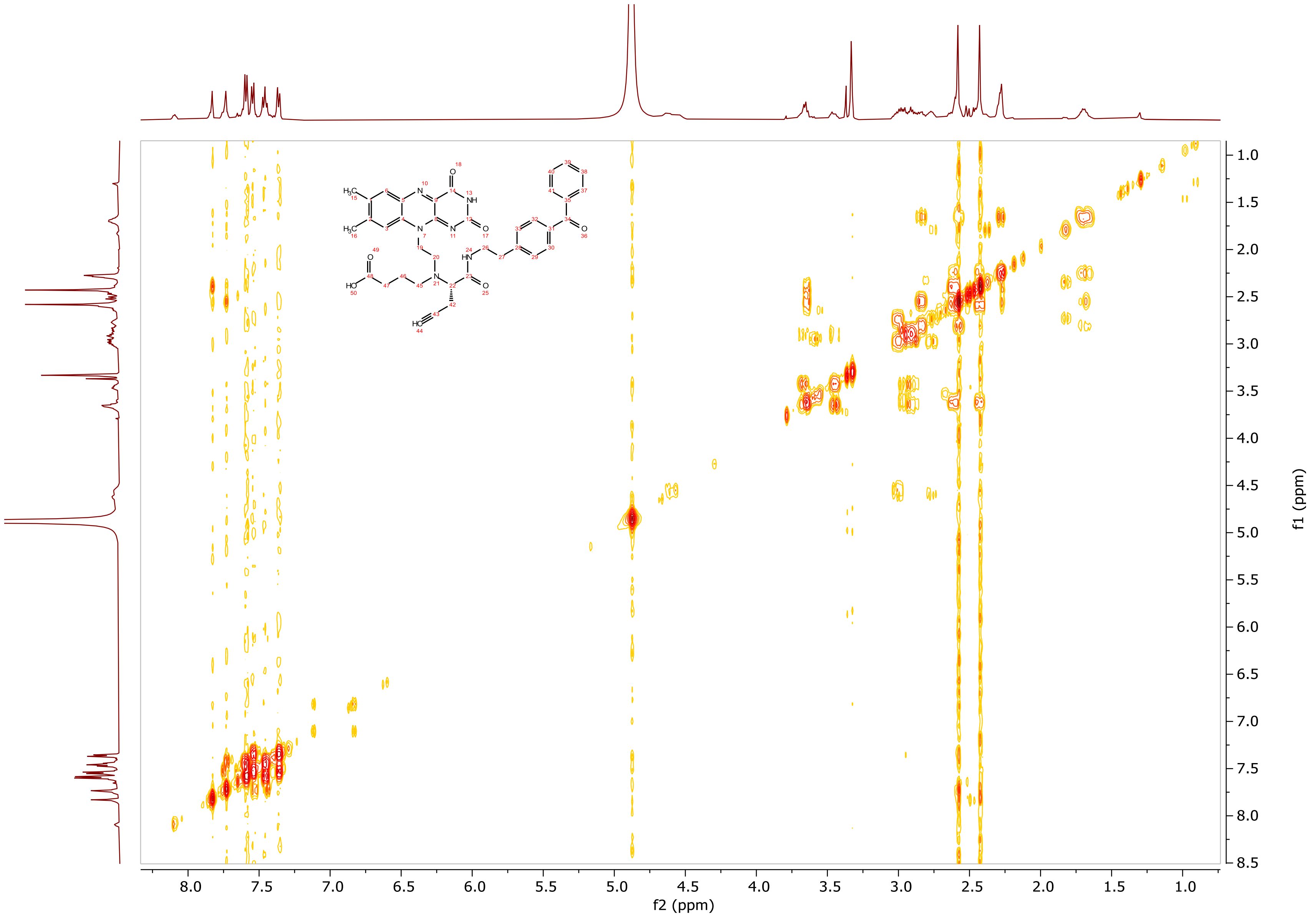
f2 (ppm)
f1 (ppm)

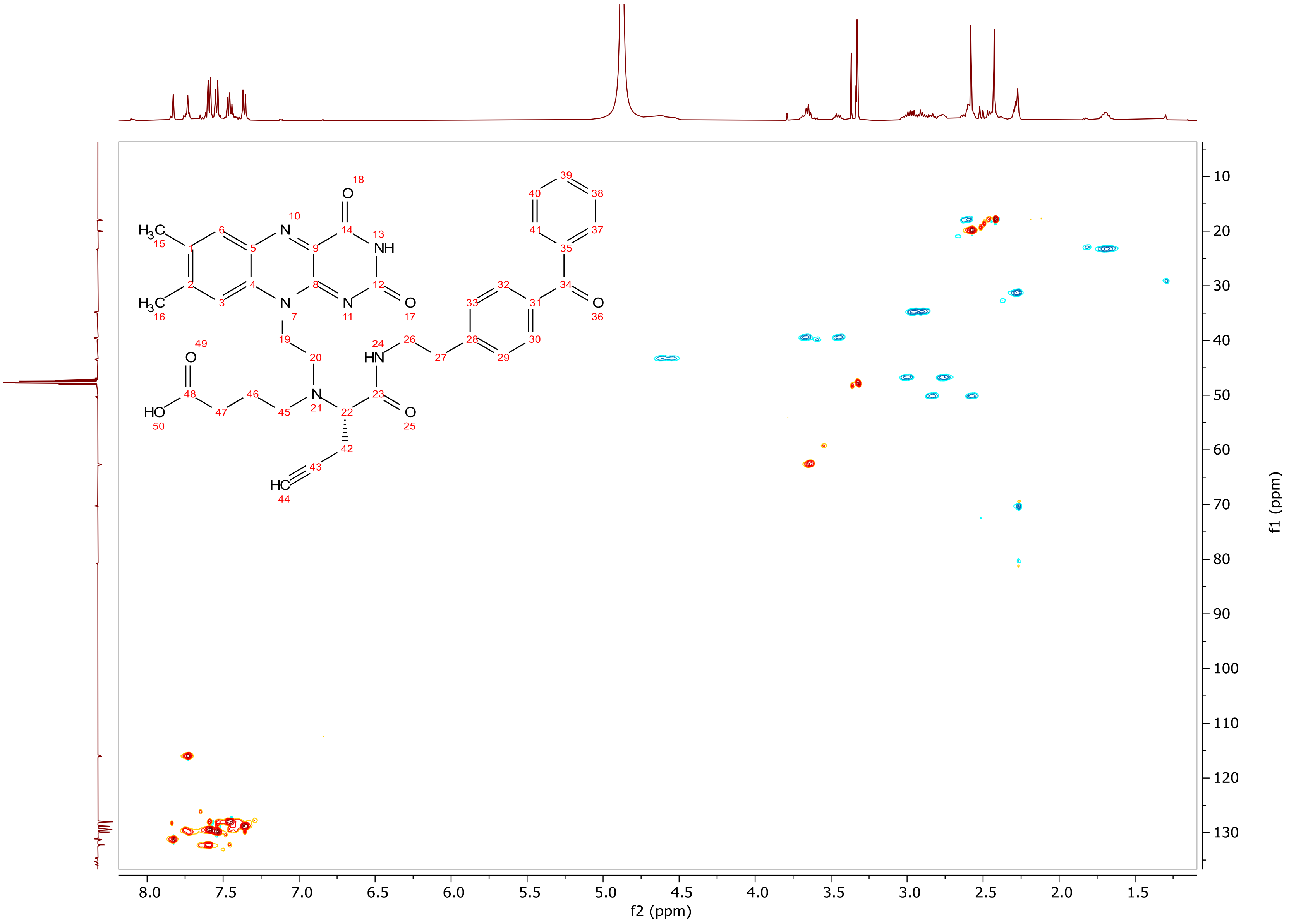

f2 (ppm)
f1 (ppm)

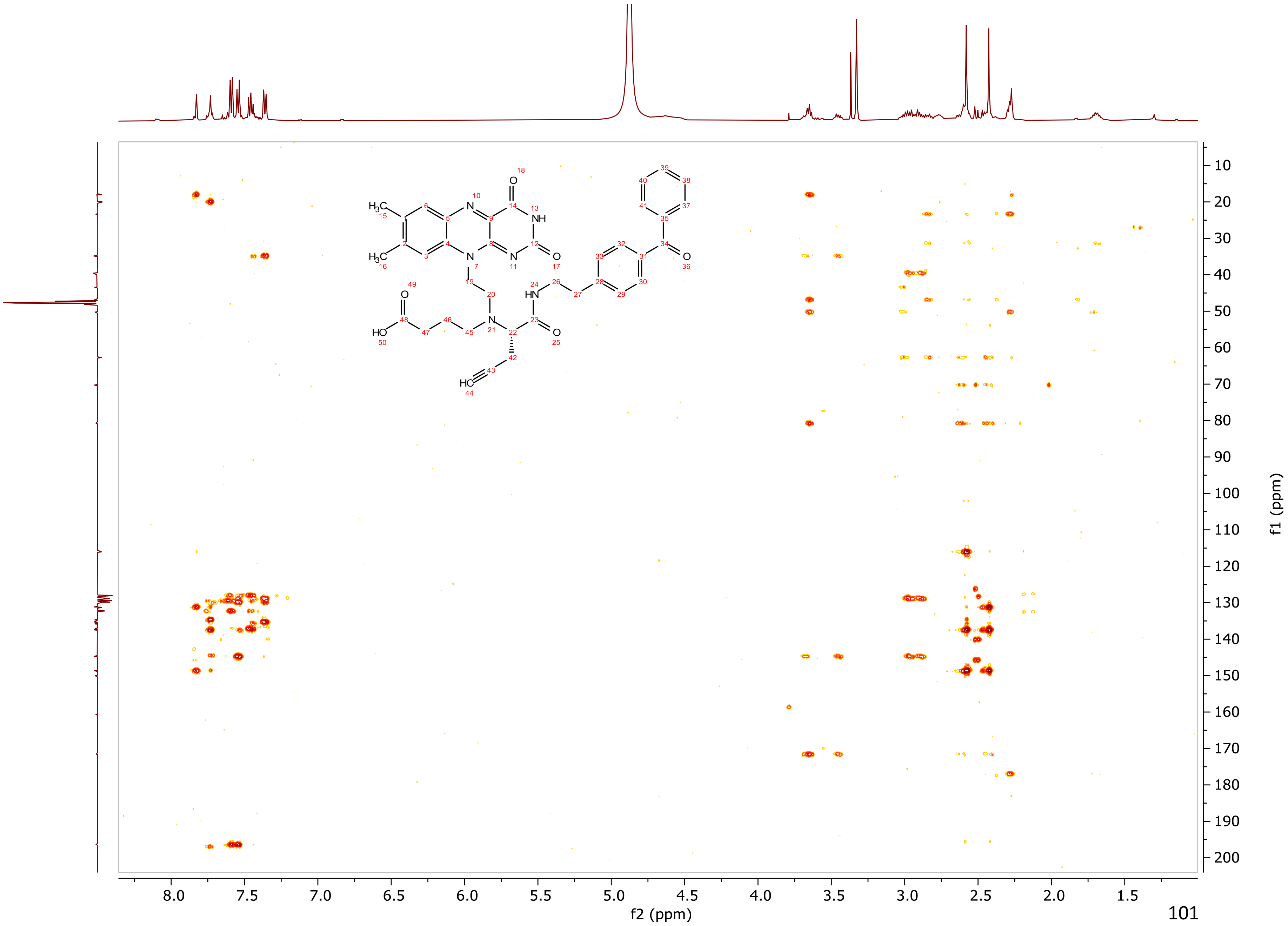
f2 (ppm)
f1 (ppm)

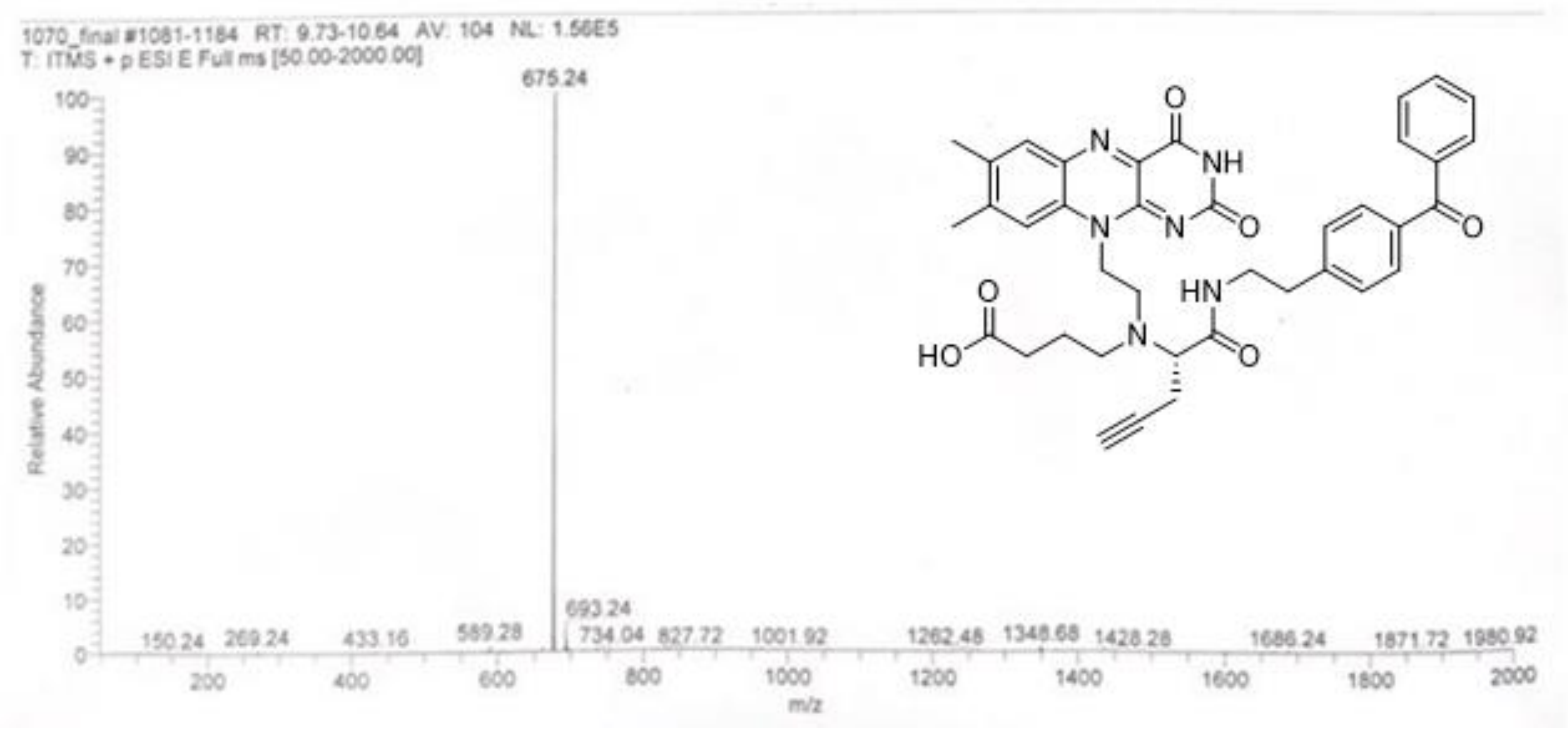
1070_final #1081-1184 RT: 9.73-10.64 AV: 104 NL: 1.56E5
T: ITMS + p ESI E Full ms [50.00-2000.00]
675.24
693.24
150.24
269.24
433.16
589.28
734.04
827.72
1001.92
1262.48
1348.68
1428.28
1686.24
1871.72
1980.92
Relative Abundance
m/z
HO
O
N
NH
HN
O

REFERENCES

(1) Wilkinson, K. A.; Merino, E. J.; Weeks, K. M. Selective 2'-Hydroxyl Acylation Analyzed by Primer Extension (SHAPE): Quantitative RNA Structure Analysis at Single Nucleotide Resolution. *Nature Protocols* **2006**, *1* (3), 1610–1616.

(2) Habibian, M.; Velema, W. A.; Kietrys, A. M.; Onishi, Y.; Kool, E. T. Polyacetate and Polycarbonate RNA: Acylating Reagents and Properties Scheme 1. Structures of RNA Acylating Reagents and Adducts. *Org. Lett* **2019**, *21*, 5413-5416.

(3) Schindelin, J.; Arganda-Carreras, I.; Frise, E.; Kaynig, V.; Longair, M.; Pietzsch, T.; Preibisch, S.; Rueden, C.; Saalfeld, S.; Schmid, B.; Tinevez, J. Y.; White, D. J.; Hartenstein, V.; Eliceiri, K.; Tomancak, P.; Cardona, A. Fiji: An Open-Source Platform for Biological-Image Analysis. *Nature Methods* **2012**, *9* (7) 676–682.

(4) Origin(Pro), 8.5.0 SR. OriginLab Corporation, Northampton, MA, USA.

(5) Serganov, A.; Huang, L.; Patel, D. J. Coenzyme Recognition and Gene Regulation by a Flavin Mononucleotide Riboswitch. *Nature* **2009**, *458* (7235), 233–237.

(6) Wilson, D. L.; Kool, E. T. Ultrafast Oxime Formation Enables Efficient Fluorescence Light-up Measurement of DNA Base Excision. *Journal of the American Chemical Society* **2019**, *141* (49), 19379–19388.

(7) Vicens, Q.; Mondragón, E.; Batey, R. T. Molecular Sensing by the Aptamer Domain of the FMN Riboswitch: A General Model for Ligand Binding by Conformational Selection. *Nucleic Acids Research* **2011**, *39* (19), 8586–8598.

(8) Vicens, Q.; Mondragón, E.; Reyes, F. E.; Coish, P.; Aristoff, P.; Berman, J.; Kaur, H.; Kells, K. W.; Wickens, P.; Wilson, J.; Gadwood, R. C.; Schostarez, H. J.; Suto, R. K.; Blount, K. F.; Batey, R. T. Structure-Activity Relationship of Flavin Analogues That Target the Flavin Mononucleotide Riboswitch. *ACS Chemical Biology* **2018**, *13* (10), 2908–2919.

(9) **Schrödinger Release 2021-4**: Maestro 12.9. Schrödinger, LLC: New York, NY 2021.

(10) Lu, C.; Wu, C.; Ghoreishi, D.; Chen, W.; Wang, L.; Damm, W.; Ross, G. A.; Dahlgren, M. K.; Russell, E.; von Bargen, C. D.; Abel, R.; Friesner, R. A.; Harder, E. D. OPLS4: Improving Force Field Accuracy on Challenging Regimes of Chemical Space. *Journal of Chemical Theory and Computation* **2021**, *17* (7), 4291–4300.

(11) Toni, L. S.; Garcia, A. M.; Jeffrey, D. A.; Jiang, X.; Stauffer, B. L.; Miyamoto, S. D.; Sucharov, C. C. Optimization of Phenol-Chloroform RNA Extraction. *MethodsX* **2018**, *5*, 599–608.

(12) Krügel, H.; Licht, A.; Biedermann, G.; Petzold, A.; Lassak, J.; Hupfer, Y.; Schlott, B.; Hertweck, C.; Platzer, M.; Brantl, S.; Saluz, H. P. Cervimycin C Resistance in Bacillus Subtilis Is Due to a Promoter Up-Mutation and Increased MRNA Stability of the Constitutive ABC-Transporter Gene BmrA. *FEMS Microbiology Letters* **2010**, *313* (2), 155–163.

(13) Pedrolli, D.; Langer, S.; Hobl, B.; Schwarz, J.; Hashimoto, M.; Mack, M. The RibB FMN Riboswitch from Escherichia Coli Operates at the Transcriptional and Translational Level and Regulates Riboflavin Biosynthesis. *FEBS Journal* **2015**, *282* (16), 3230–3242.

(14) Chang, A. Y.; Chau, V. W.; Landas, J. A.; Pang, Y. Preparation of Calcium Competent *Escherichia Coli* and Heat-Shock Transformation. *JEMI Methods* **2017**, *1*, 22-25.

(15) Howe, J. A.; Wang, H.; Fischmann, T. O.; Balibar, C. J.; Xiao, L.; Galgoci, A. M.; Malinverni, J. C.; Mayhood, T.; Villafania, A.; Nahvi, A.; Murgolo, N.; Barbieri, C. M.; Mann, P. A.; Carr, D.; Xia, E.; Zuck, P.; Riley, D.; Painter, R. E.; Walker, S. S.; Sherborne, B.; de Jesus, R.; Pan, W.; Plotkin, M. A.; Wu, J.; Rindgen, D.; Cummings, J.; Garlisi, C. G.; Zhang, R.; Sheth, P. R.; Gill, C. J.; Tang, H.; Roemer, T. Selective Small-Molecule Inhibition of an RNA Structural Element. *Nature* **2015**, *526* (7575), 672–677.

(16) Velema, W. A.; van der Berg, J. P.; Hansen, M. J.; Szymanski, W.; Driessen, A. J. M.; Feringa, B. L. Optical Control of Antibacterial Activity. *Nature Chemistry* **2013**, *5* (11), 924–928.

(17) Murahashi, S. I.; Zhang, D.; Iida, H.; Miyawaki, T.; Uenaka, M.; Murano, K.; Meguro, K. Flavin-Catalyzed Aerobic Oxidation of Sulfides and Thiols with Formic Acid/Triethylamine. *Chemical Communications* **2014**, *50* (71), 10295–10298.

(18) Chen, S.; Zhao, X.; Chen, J.; Chen, J.; Kuznetsova, L.; Wong, S. S.; Ojima, I. Mechanism-Based Tumor-Targeting Drug Delivery System. Validation of Efficient Vitamin Receptor-Mediated Endocytosis and Drug Release. *Bioconjugate Chemistry* **2010**, *21* (5), 979–987.
